# Lithium‐Catalyzed Thiol Alkylation with Tertiary and Secondary Alcohols: Synthesis of 3‐Sulfanyl‐Oxetanes as Bioisosteres

**DOI:** 10.1002/chem.201705576

**Published:** 2017-12-20

**Authors:** Rosemary A. Croft, James J. Mousseau, Chulho Choi, James A. Bull

**Affiliations:** ^1^ Department of Chemistry Imperial College London South Kensington, London SW7 2AZ UK; ^2^ Pfizer Medicine Design Eastern Point Road Groton CT 06340 USA

**Keywords:** bioisosteres, homogeneous catalysis, oxetanes, oxygen heterocycles, sulfides

## Abstract

3‐Sulfanyl‐oxetanes are presented as promising novel bioisosteric replacements for thioesters or benzyl sulfides. From oxetan‐3‐ols, a mild and inexpensive Li catalyst enables chemoselective C−OH activation and thiol alkylation. Oxetane sulfides are formed from various thiols providing novel motifs in new chemical space and specifically as bioisosteres for thioesters due to their similar shape and electronic properties. Under the same conditions, various π‐activated secondary and tertiary alcohols are also successful. Derivatization of the oxetane sulfide linker provides further novel oxetane classes and building blocks. Comparisons of key physicochemical properties of the oxetane compounds to selected carbonyl and methylene analogues indicate that these motifs are suitable for incorporation into drug discovery efforts.

Organosulfur functional groups are often present in pharmaceutical compounds, found in a quarter of the top 200 drugs (branded drugs by US retail sales in 2011).^1^, ^2^ Benzylic sulfides, sulfoxides and sulfones are particularly prevalent, such as in AstraZeneca's blockbuster antiulcerant Nexium, Figure 1.^3^ Thioester‐containing compounds have also been disclosed, but are often used as a pro‐drug due to limited metabolic stability;^4^ thioesters are 100× more reactive to amine nucleophiles than esters.^5^ However, to date there are no suitable bioisosteres for these functional groups to provide improved stability at the C‐center, limiting this design space for medicinal chemists.

**Figure 1:**
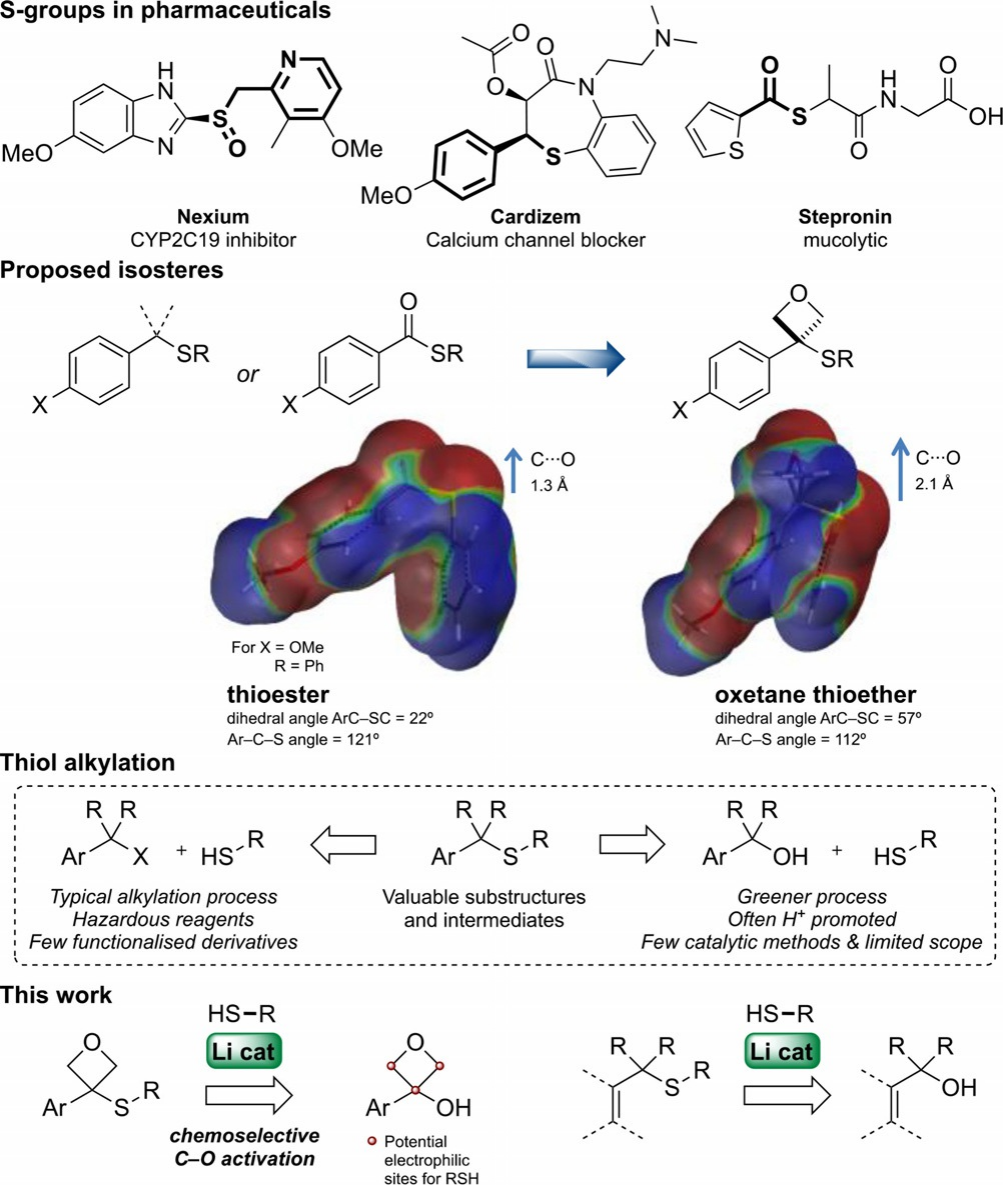
The design of oxetane sulfides as bioisosteres for thioesters and new structural motifs, via a thioalkylation strategy.

Oxetanes have emerged as valuable motifs in medicinal chemistry that can confer improved physicochemical and metabolic properties.^6^ Oxetanes can act as suitable polar replacement groups for *gem*‐dimethyl linkers and as bioisosteres for carbonyl functionality.^7^ In recent years, oxetane isosteres have been presented for amide,^8^ ketone^9^ and carboxylic acid derivatives.^10^ These developments continue to accelerate the exploration of oxetanes in medicinal chemistry.6a, 11

We were interested in the potential of 3‐sulfanyloxetane derivatives as isosteres for sulfides or thioesters (Figure 1). This little explored class of compounds would offer similar features to thioesters, based on the dipole and lone pair position of the oxetane, but without the electrophilic center. Indeed, our DFT studies indicated that while the thioester C−O bond length is calculated to be 0.8 Å shorter than the oxetanyl sulfide, their electrostatic mapping is very similar.^12^, ^13^ Additionally, these motifs may offer a protected benzylic center for sulfides or oxidized derivatives. Encouragingly, Bernardes recently reported a mono‐substituted 3‐sulfanyloxetane as part of a modified protein which was stable under incubation with blood plasma and with glutathione.^14^ However, synthetic access to these motifs remains limited,^15^, ^16^, ^17^, ^18^ particularly towards 3,3‐disubstituted examples; Ellman^19^ and Sun^20^ reported 3‐alkyl‐3‐sulfanyloxetanes through conjugate addition of a sulfide to oxetane‐Michael acceptors.

We envisaged an S_n_1 process for the formation of oxetane sulfides from 3‐aryloxetan‐3‐ols (Figure 1). We recently reported a Li‐catalyzed Friedel–Crafts reaction using oxetanols to form diaryloxetanes, invoking an oxetane carbocation.^9^ The catalytic activation of alcohols through C−O activation has become an attractive alternative to replace more toxic alkyl halides.^21^, ^22^ However, there are only infrequent examples of catalytic thiol alkylation on functionalized substrates, which often require high catalyst loadings with acidic reagents.^23^ Furthermore, ring opening of oxetanes by S‐nucleophiles under acidic conditions^24^ presents a significant chemoselectivity concern. Here we report a high yielding Li‐catalyzed alkylation of thiols with oxetanol derivatives. Preliminary data suggest that the methods described can be applied in the synthesis of targets with attractive physicochemical properties for drug discovery.

Building on our prior studies and with the above considerations in mind, we investigated the alkylation of benzylmercaptan with oxetanol **1** (Table 1).

**Table 1:** Selected optimization for the reaction of **1** with benzylmercaptan.

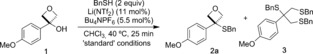			
	Change from the “standard” conditions	Yield **2 a** [%]^[a]^	Yield **3** [%]^[a]^
**1**	**none**	**81(67)**	**<5**
2	FeCl_3_ (5 mol %), instead of Li(NTf_2_)/ Bu_4_NPF_6_	18	0
3^[b]^	Ca(NTf_2_)_2_ (5 mol %), Bu_4_NPF_6_ (5 mol %) instead of Li(NTf_2_)	62	0
4	Ga(OTf)_3_ (5 mol %), instead of Li(NTf_2_)/ Bu_4_NPF_6_	21	11^[c]^
5	Bi(OTf)_3_ (5 mol %), instead of Li(NTf_2_)/ Bu_4_NPF_6_	4	0^[d]^
6	TsOH.H_2_O (5 mol %), instead of Li(NTf_2_)/ Bu_4_NPF_6_	0	0^[e]^
7	toluene, instead of CHCl_3_	61	16
8	No Li(NTf_2_)	0	0
9	No Bu_4_NPF_6_	0	0
10	3 equiv BnSH, 10 min reaction time	0	0
11	3 equiv BnSH	75	9
12	6 equiv BnSH, 6 h	0	97

[a] Yield determined by ^1^H NMR using 1,3,5‐trimethoxybenzene as internal standard. Yield of isolated product in parentheses. [b] See reference 24 for development of Ca reagents. [c] 45 % recovered **1**. [d] Trace amount of recovered **1**. [e] 63 % recovered **1**.

We optimized the reaction to form oxetane sulfide **2 a**, and to minimize ring‐opened side product **3** in which all alkyl C−O bonds had reacted. The optimized ‘standard“ conditions used 2 equivalents of benzylmercaptan and a Li catalyst in chloroform at 40 °C for 25 min. The use of the inexpensive and easily handled salt Li(NTf_2_) (11 mol %) with Bu_4_NPF_6_ (5.5 mol %) as an additive gave a 67 % isolated yield of oxetane sulfide **2 a** and minimal tri‐S‐benzylated product **3**. Catalysts such as FeCl_3_, Ca(NTf_2_)_2_ or Ga(OTf)_3_ gave lower yields compared to the standard conditions (entries 2–4). Bi(OTf)_3_ and TsOH gave no productive reaction (entries 5–6). Using toluene as solvent was also successful with slightly reduced yield and selectivity (entry 7). No reaction occurred in the absence of catalyst or additive (entries 8–9). Reducing the reaction time to 10 min gave no conversion, due to an activation period for the reaction, believed to involve solubilizing the lithium species, likely the key role of the Bu_4_NPF_6_ additive (entry 10). Increasing the reaction time and/or the equivalents of nucleophile resulted in an increased yield of ring opened **3** (entry 11). Indeed, a longer reaction time of 6 h and 6 equivalents of benzylthiol, gave **3** as a single product in a remarkable 97 % isolated yield (entry 12).^25^ This ring opening reactivity highlights the high initial selectivity achieved with the Li catalyst in forming the putative carbocationic intermediate.

Different thiols were examined under the standard conditions; benzylic thiols gave **2 a** and **2 b** in similar yields (Scheme 1). A diverse set of thiophenols gave excellent yields of sulfides **2 c**–**2 l** with the reaction insensitive to the electronic and steric nature of the substituents. 4‐Hydroxythiolphenol gave complete selectivity for the S‐alkylated product **2 j** with no Friedel–Crafts or O‐alkylated product observed. Aliphatic thiols such as tertiary 1‐adamantanethiol (**2 m**) and primary butyl‐3‐mercaptoproprionate (**2 n**) were also successful. However, NBoc‐cysteine methyl ester and 2‐(Boc‐amino)ethanethiol did not afford the corresponding 3‐sulfanyloxetanes, giving complete recovery of starting material. The comparison to butyl‐3‐mercaptoproprionate suggests that coordination of the NHBoc group to the catalyst causes deactivation. Other preinstalled electron‐rich aromatic groups were successful in stabilizing the oxetane‐carbocation intermediate. A TIPS‐protected phenol gave oxetane sulfide **8** in 66 % yield. A 3,4,5‐trimethoxybenzene group yielded oxetane sulfide **9** in a low yield (23 %) due to increased oxetane ring opening. Furan and indole substituted oxetanols gave the corresponding oxetane sulfides **10** and **11** in excellent yields of 86 % and 91 % respectively.

**Scheme 1:**
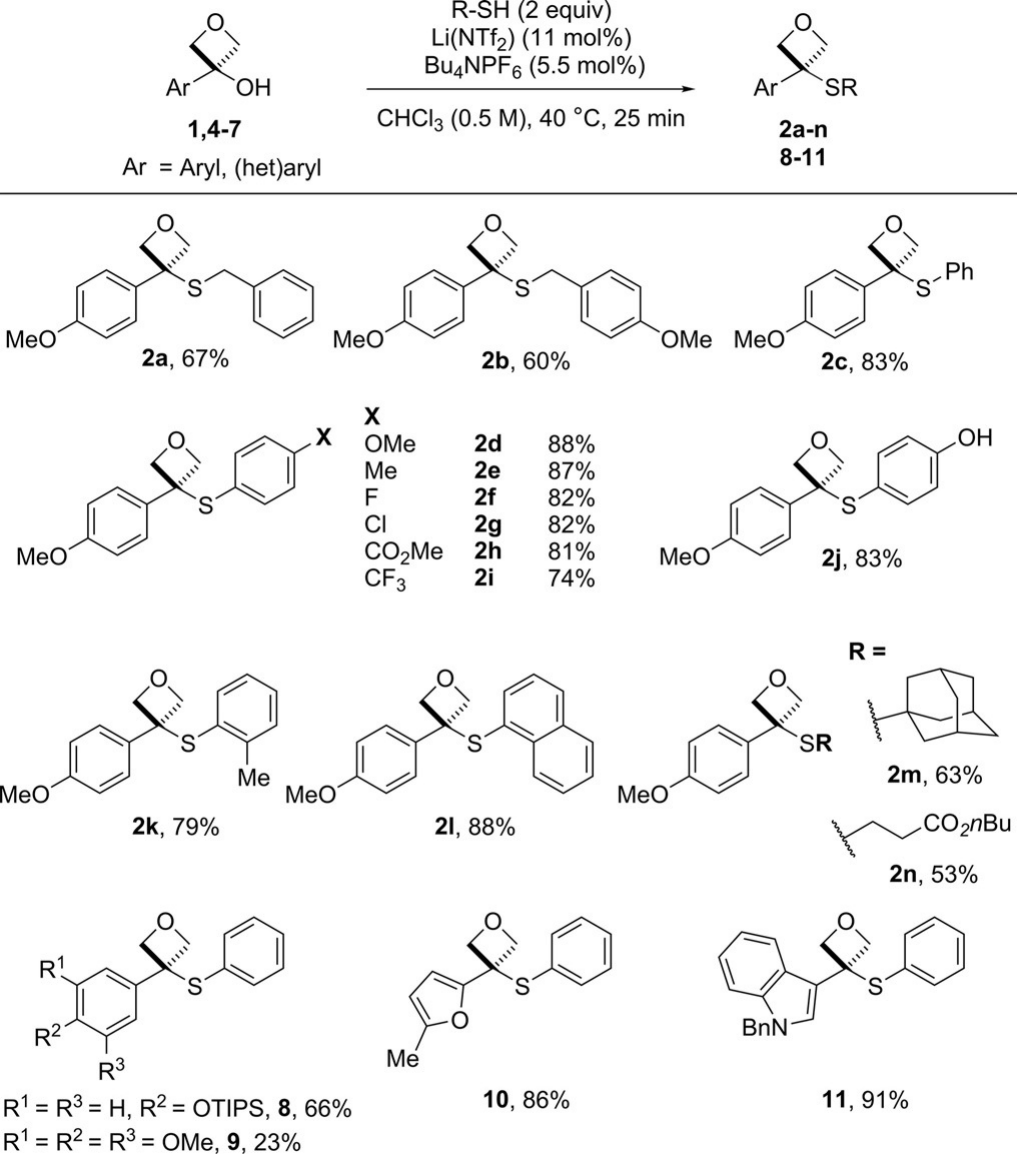
Scope of oxetanylsulfides using aryl, benzyl and alkyl thiols.

Other varied π‐activated secondary and tertiary alcohols (**12**) were explored to demonstrate the wider applicability of these reaction conditions. Excellent yields were obtained of tertiary benzylic sulfides bearing tetrahydropyran, cyclohexane and cyclobutane linkers as well as secondary and tertiary propargylic and allylic sulfides (see Supporting Information page S18, for full details; 10 examples **13 a**‐**‐j**).

Next, we explored functionalization of the oxetane sulfides. Deprotection of the TIPS group of oxetane sulfide **8**, followed by formation of the triflate **14** occurred in high yields, providing a building block for further reaction (Scheme 2 A). Biaryl **15** was formed by Suzuki–Miyaura cross‐coupling in 95 % yield.^26^ The high yield demonstrated the excellent stability of the oxetane sulfide unit to the reaction conditions. Oxidation of the oxetane sulfides with *m*CPBA formed selectively 3‐sulfinyloxetanes **16 c,d,f** or 3‐sulfonyloxetanes **17 c,d,f** as new oxetane structural classes (Scheme 2 B).

**Scheme 2:**
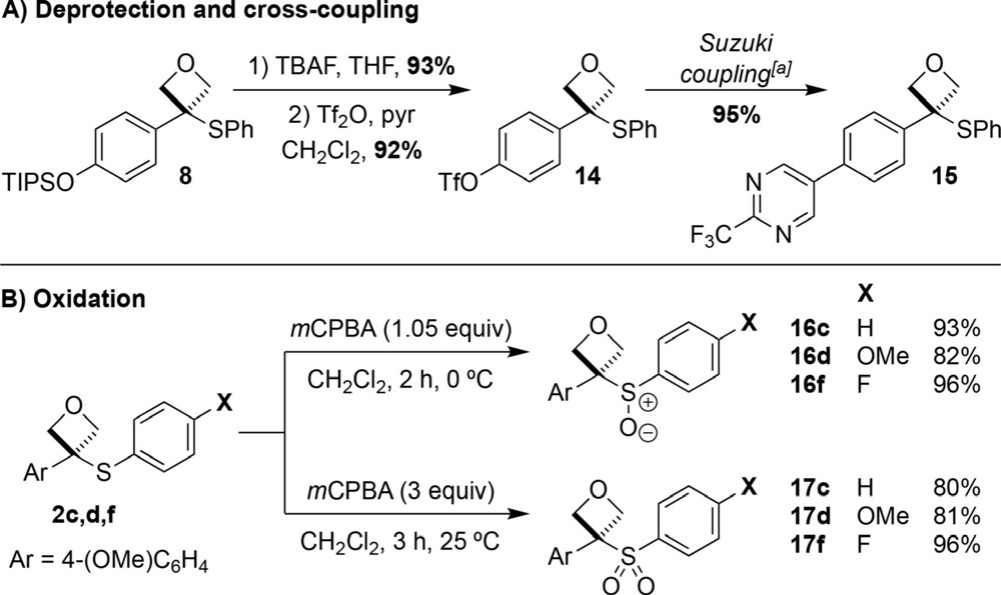
A) Cross‐coupling of an oxetane sulfide. B) Oxidation to the oxetane sulfoxide or sulfone. [a] See Supporting Information for conditions.

Oxetane–cysteine derivatives continued to make an attractive target as alkylated cysteines feature in several marketed drugs.^27^ As protected cysteine did not react with oxetanol **1** directly, an alternative route via oxetane thiol **18** was devised (Scheme 3). Typically, conversion of a tertiary alcohol to a thiol involves Lawesson's reagent or heating under acidic conditions with thiourea.^28^ From oxetanol **1**, a mild and convenient two step, one pot procedure was developed using tritylthiol under thiol alkylation conditions. In situ deprotection (trifluoroacetic acid (TFA) and triethylsilane) gave 53 % of oxetane thiol **18**.^13^ Reaction of **18** with a protected dehydroalanine derivative yielded sulfide **19** in quantitative yield. Ester hydrolysis and Boc deprotection afforded the racemic target unnatural amino acid **20**.

**Scheme 3:**
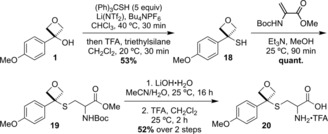
Synthesis of an unnatural oxetane containing amino acid **20**.

In the context of drug discovery, the distribution coefficient of a compound strongly affects how effectively the drug can reach its intended target, as well as efficacy and pharmacokinetic properties. Hence, Log*D* is often used by medicinal chemists in pre‐clinical drug discovery to consider the drug‐likeness of an intended target molecule. To understand the effect of oxetane sulfides on this key parameter, Log*D* was measured for compounds **21**, **2 d**, and **17 d**. Replacing the thioester functionality with the oxetane sulfide had the positive effect of lowering the Log*D* by approximately 1 Log unit (Figure 2). Furthermore, oxidizing the sulfide to the sulfone further dramatically decreased the lipophilicity of the compound, furnishing **17 d** in very favorable property space. In addition, the clearance and cell permeability of **20** and **22** were also explored. While oxetane **20** displayed slightly lower cell permeability than the corresponding methane analogue **22**, the clearance profile by human liver microsomes of both substrates was determined to be the same, indicating that inclusion of an oxetane in this substrate is not a metabolic liability. The combination of this data, in addition to the improved Log*D* suggests that previous observations of oxetanes lending improved drug property space extends to these novel oxetane sulfides.

**Figure 2:**
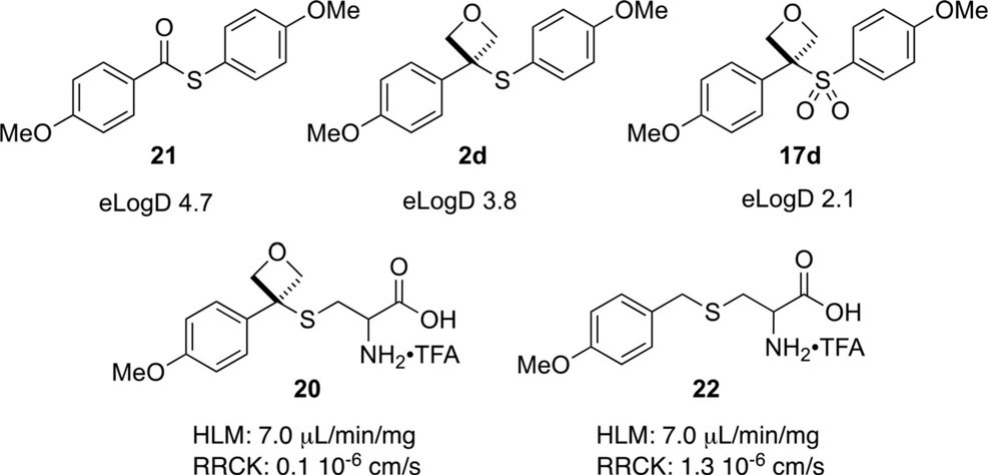
Comparison of physicochemical parameters. eLog*D*=distribution coefficient (Log*D*) measured by HPLC. HLM=Clearance in liver microsomes; RRCK=Cell membrane permeability.

In summary, 3‐sulfanyloxetanes offer promising bioisosteric replacement groups for thioesters, and new design elements for medicinal chemistry. The first Li‐catalyzed thiol alkylation with alcohol substrates is described, suitable for both oxetanol derivatives, and more generally with π‐activated secondary and tertiary alcohols. Remarkably, complete chemoselectivity can be achieved for the activation of the C−OH group of oxetanols, over competing oxetane ring opening. The use of the mild and inexpensive Li catalyst was crucial and careful control of the reaction conditions gave high yields of the oxetane sulfides. The oxetane sulfides were compatible with palladium catalyzed cross‐coupling and were converted to sulfoxide, sulfone, and thiol derivatives; themselves providing new classes of oxetane containing compounds. Measurement of key physicochemical properties: Log*D*, clearance and cell permeability, indicated that oxetane sulfides and sulfones are attractive for medicinal chemistry applications.

## Conflict of interest

The authors declare no conflict of interest.

## Supporting information

As a service to our authors and readers, this journal provides supporting information supplied by the authors. Such materials are peer reviewed and may be re‐organized for online delivery, but are not copy‐edited or typeset. Technical support issues arising from supporting information (other than missing files) should be addressed to the authors.

Supplementary
