# Supplementary material for: Lithium‐Catalyzed Thiol Alkylation with Tertiary and Secondary Alcohols: Synthesis of 3‐Sulfanyl‐Oxetanes as Bioisosteres
Source: Chemistry. 2017 Dec 20;24(4):818–21. doi: 10.1002/chem.201705576 (PMC5814735; doi:10.1002/chem.201705576)
Supplement: Supplementary file 1 — Supplementary [file CHEM-24-818-s001.pdf]

CHEMISTRY
A European Journal

Supporting Information

Lithium-Catalyzed Thiol Alkylation with Tertiary and Secondary Alcohols: Synthesis of 3-Sulfanyl-Oxetanes as Bioisosteres

Rosemary A. Croft,[a] James J. Mousseau,[b] Chulho Choi,[b] and James A. Bull*[a]

chem_201705576_sm_miscellaneous_information.pdf

Table of Contents

Table of Contents S1
Complete References **7b, 10, 11a** S2
Comparison of Thioester and Oxetane Sulfide Structural Features and Properties (DFT) S3–S10
General Experimental Considerations S7
Synthesis of Oxetanols **1, 4–7** S8–10
Synthesis of Oxetane-3-sulfides **2a–n, 8–11** S11–17
Thiol Alkylation with Secondary and Tertiary Thiols **12a–j** S18
Synthesis of Thiol Alkylation Precursors **12a–c, j** S19–20
Synthesis of Tertiary Sulfides **13a–e** S21–22
Synthesis of Secondary Sulfides **13f–j** S23–24
Derivatization of Oxetane Sulfide **8** S25
Synthesis of Oxetane-3-Sulfoxides **16c**,**d**,**f** S26
Synthesis of Oxetane-3-Sulfones **17c**,**d**,**f** S27
Optimization of 1-pot Synthesis of Oxetane-3-thiol **18** S28–29
Synthesis of Oxetane Cysteine Derivative **20** S30–31
Synthesis of Thioester **21** and Benzylic Sulfide **22** S32
References S33
^{1}H and ^{13}C NMR Spectra for Selected Compounds S34–87

References: Complete references 7b, 10, 11a are given below:

[7] b) G. Wuitschik, M. Rogers-Evans, A. Buckl, M. Bernasconi, M. Märki, T. Godel, H. Fischer, B. Wagner, I. Parrilla, F. Schuler, J. Schneider, A. Alker, W. B. Schweizer, K. Müller, E. M. Carreira, *Angew. Chem. Int. Ed.* **2008**, *47*, 4512–4515; *Angew. Chem*. **2008**, *120*, 4588–4591.

[10] P. Lassalas, K. Oukoloff, V. Makani, M. James, V. Tran, Y. Yao, L. Huang, K. Vijayendran, L. Monti, J. Q. Trojanowski, V. M.-Y. Lee, M. C. Kozlowski, A. B. Smith, K. R. Brunden, C. Ballatore, *ACS Med. Chem. Lett.* **2017**, *8*, 864.

[11] a) A. F. Stepan, K. Karki, W. S. McDonald, P. H. Dorff, J. K. Dutra, K. J. DiRico, A. Won, C. Subramanyam, I. V. Efremov, C. J. O'Donnell, C. E. Nolan, S. L. Becker, L. R. Pustilnik, B. Sneed, H. Sun, Y. Lu, A. E. Robshaw, D. Riddell, T. J. O'Sullivan, E. Sibley, S. Capetta, K. Atchison, A. J. Hallgren, E. Miller, A. Wood, R. S. Obach, *J. Med. Chem.* **2011**, *54*, 7772–7783.

Comparison of Thioester and Oxetane Sulfide Structural Features and Properties (DFT)

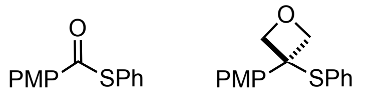

I II

Thioester I and oxetane II were modelled using DFT to compare calculated properties. Calculations were performed using Spartan '16, Version 2.0.3, Jan 13 2017, from Wavefunction. The work flow involved investigating a conformer distribution using Merck Molecular Force Field (MMFF) with maximum of 1000 conformers analyzed. The lowest energy determined conformer was then minimized through equilibrium geometry in the gas phase at DFT ωB97X-D 6-311G* level theory.

Comparison of Ar-C-S bond angles and dihedral angles ArC-SAr

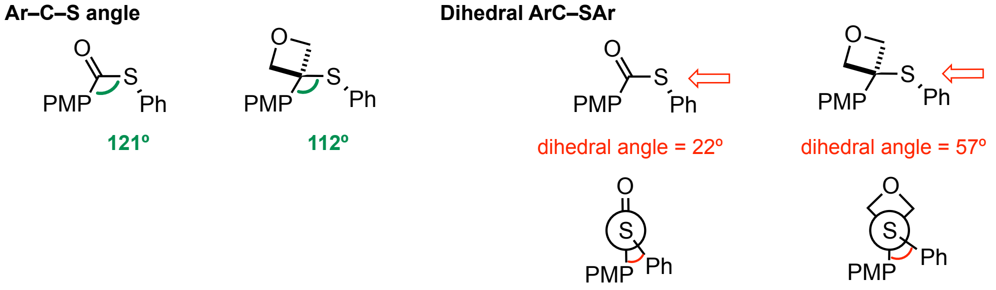

Minimized Structure of Thioester **I**

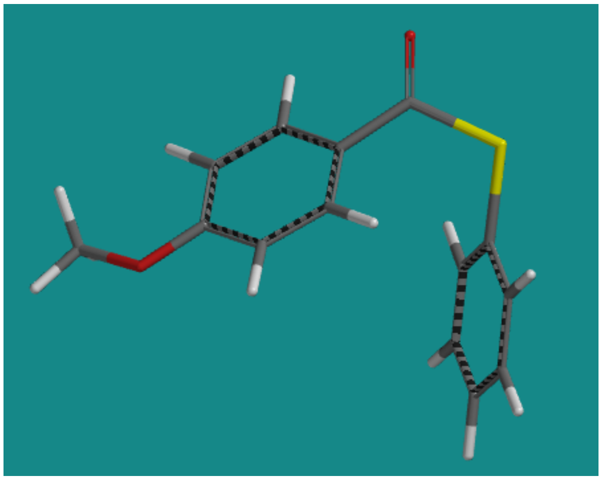

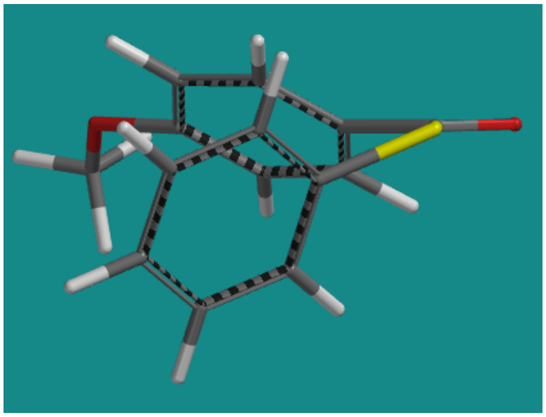

Minimized Structure of Oxetane Thioether **II**

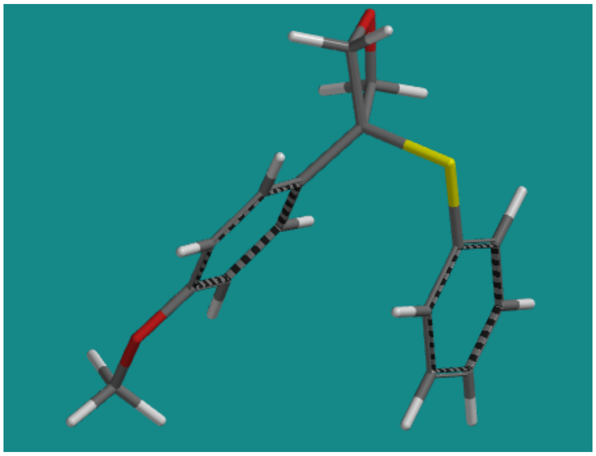

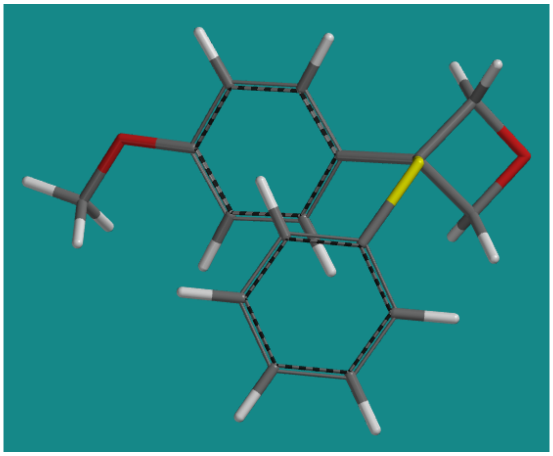

Electrostatic Mapping (-25 to 25 kJ/mol)

Thioester **I**

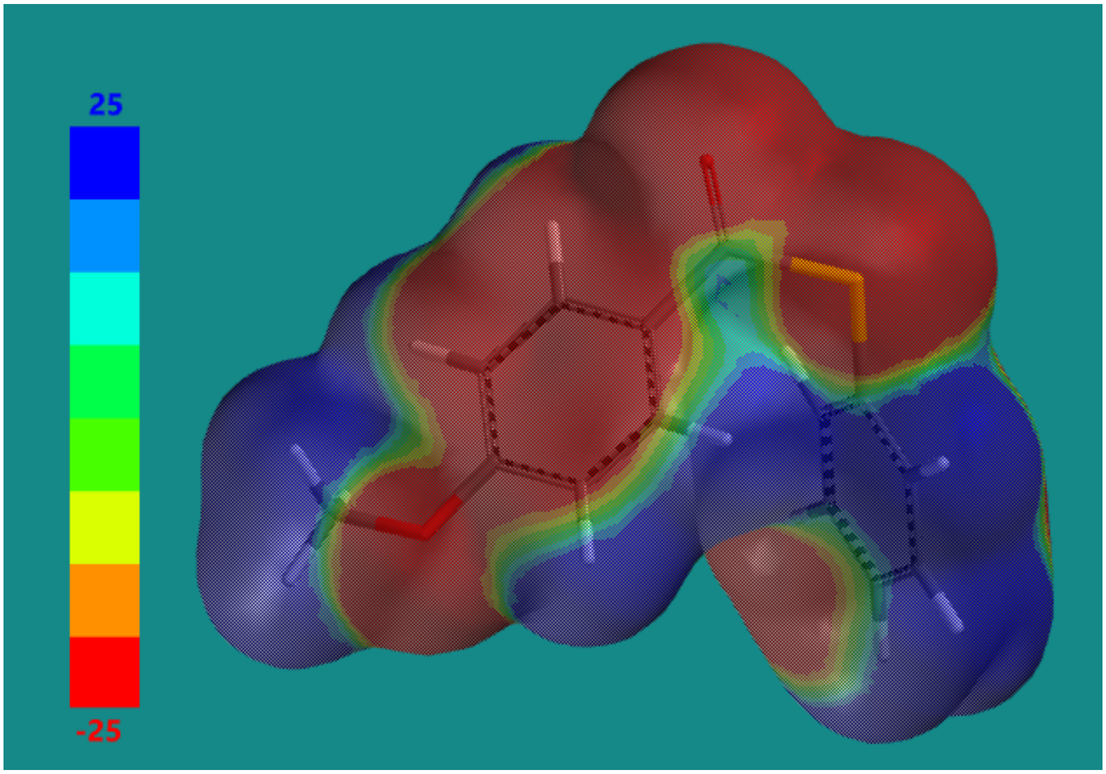

Oxetane Thioether **II**

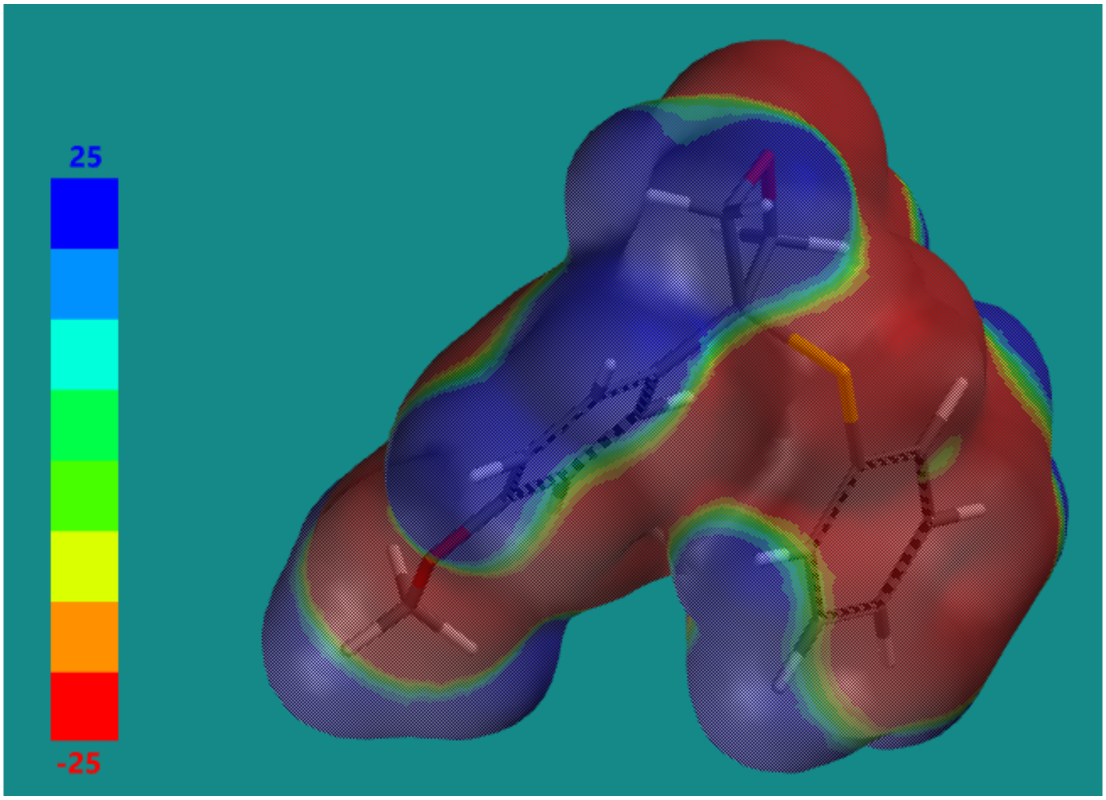

HOMO/LUMO Map:

Thioester I

LUMO = 0.4 eV

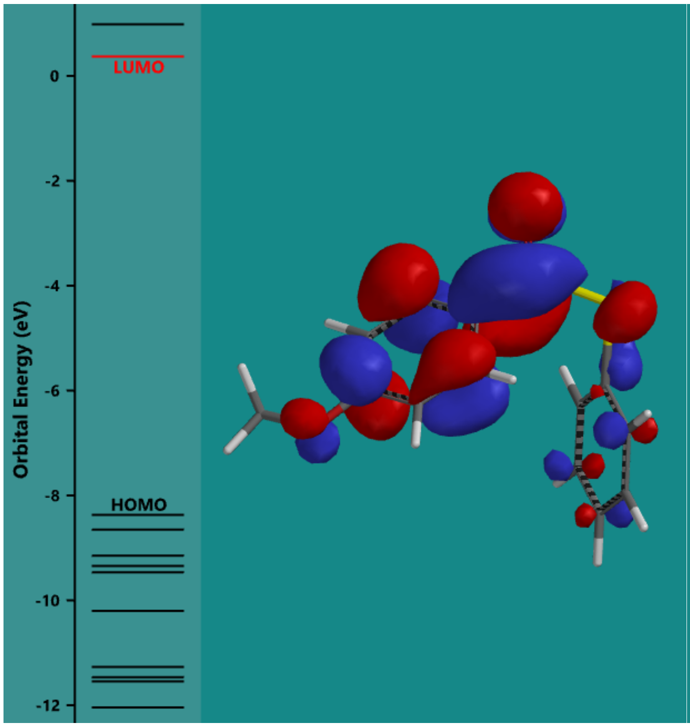

HOMO = –8.4 eV

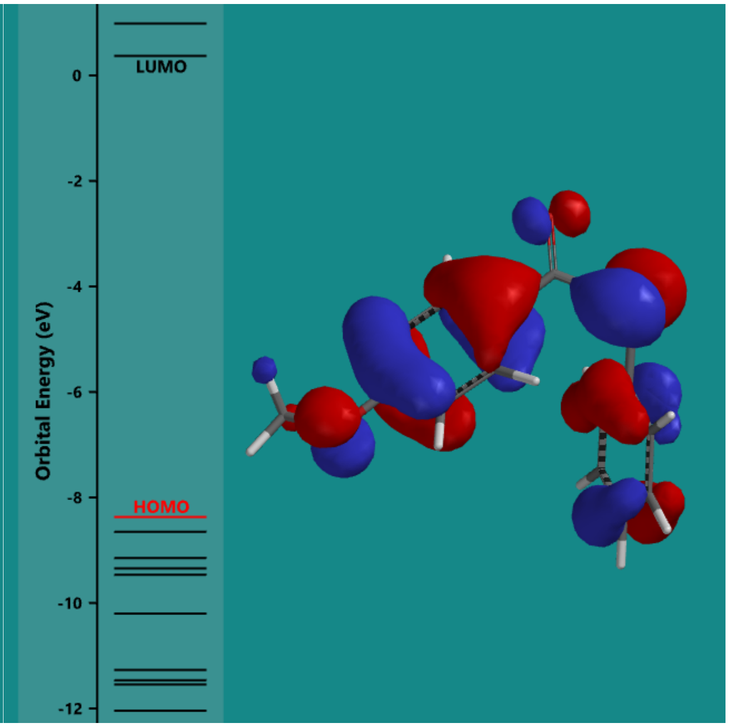

Oxetane Thioether II

LUMO = 1.0 eV

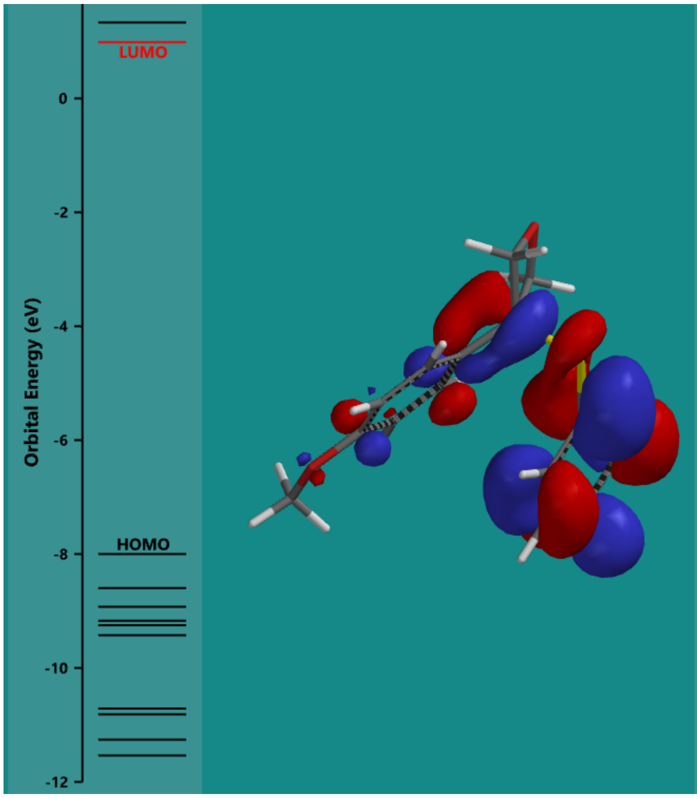

HOMO = –8.0 eV

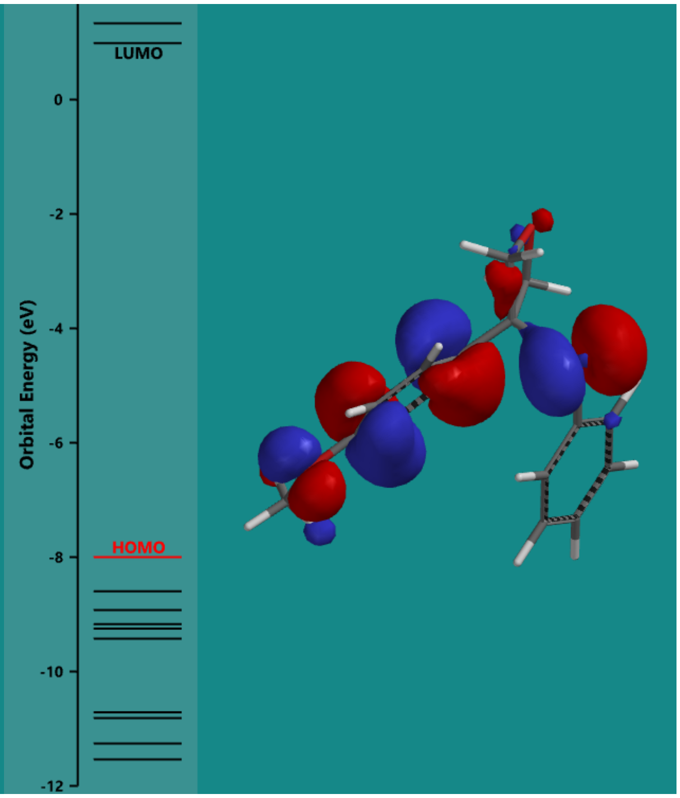

Other Calculated Property Comparisons:

	Thioester I	Oxetane Sulfide II
	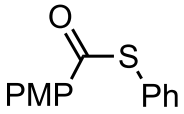	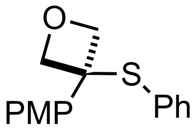
logP:	3.96	3.54
HBD:	0	0
HBA:	3	3
polarizability	59.5	62.1
volume	248.4 Å^3	281.8 Å^3
PSA	21.4 Å^2	13.3 Å^2

General Experimental Considerations

All nonaqueous reactions were run under an inert atmosphere (argon) with flame-dried glassware using standard techniques. Anhydrous solvents were obtained by filtration through drying columns (CH_3CN, THF, CH_2Cl_2, MeOH) or used directly from commercial sources ($CHCl_3$). Flash column chromatography was performed using 230-400 mesh silica with the indicated solvent system according to standard techniques. Analytical thin-layer chromatography (TLC) was performed on precoated, glass-backed silica gel plates. Visualization of the developed chromatogram was performed by UV absorbance (254 nm), aqueous potassium permanganate stain or PMA (phosphomolybdic acid). Infrared spectra (ν_{max}, FTIR ATR) were recorded in reciprocal centimeters (cm^{-1}). Nuclear magnetic resonance (NMR) spectra were recorded on 400 MHz spectrometers. Chemical shifts for ^1H NMR spectra are recorded in parts per million from tetramethylsilane with the solvent resonance as the internal standard ($CDCl_3$, δ = 7.27 ppm, CD_3OD: 3.31 ppm, $(CD_3)_2SO$: 2.50 ppm). Data is reported as follows: chemical shift [multiplicity (s = singlet, d = doublet, t = triplet, quin = quintet, sep = septet, m = multiplet and br = broad), coupling constant in Hz, integration, assignment]. ^{13}C NMR spectra were recorded with complete proton decoupling. Chemical shifts are reported in parts per million from tetramethylsilane with the solvent resonance as the internal standard ($^{13}CDCl_3$: 77.0 ppm, $^{13}CD_3OD$: 49.0 ppm, $^{13}(CD_3)_2SO$: 39.5 ppm). ^{19}F NMR spectra were recorded with complete proton decoupling. Chemical shifts are reported in parts per million referenced to the standard monofluorobenzene: −113.5 ppm. Chemical shifts are reported in parts per million. *J* values are reported in Hz. Assignments of $^1H/^{13}C$ spectra were made by the analysis of δ/J values, and COSY, HSQC, and HMBC experiments as appropriate. Melting points were obtained using Optimelt MPA100 melting point apparatus and are uncorrected.

Reagents: Commercial reagents were used as supplied or purified by standard techniques where necessary.

Synthesis of Oxetanols 1,4–7

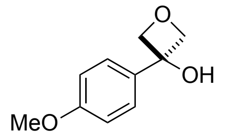

3-(4-Methoxyphenyl)oxetan-3-ol (1)[1]

n-BuLi (2.24 M in THF, 5.80 mL, 13.0 mmol) was added dropwise over 5 min to a solution of 4-bromoanisole (1.63 mL, 13.0 mmol) in THF (45 mL) at –78 °C. The reaction mixture was stirred at –78 °C for a further 10 min. Oxetan-3-one (0.65 mL, 10.0 mmol) in THF (5 mL) was added dropwise to the reaction mixture. Following a further 10 min at –78 °C the reaction mixture was warmed to rt then quenched with water (40 mL). The layers were separated and the aqueous portion extracted with diethylether (3 × 30 mL). The organic extracts were combined, washed with brine, dried over Na_2SO_4, filtered and concentrated *in vacuo*. Purification by flash column chromatography (30% EtOAc/hexane) afforded oxetanol **1** (1.58 g, 88%) as a white solid. R_f = 0.23 (50% EtOAc/hexane); mp = 52–53 °C; IR (film)/cm^{-1} 3315 (br. OH), 2995, 2950, 2882, 1607, 1581, 1511, 1461, 1439, 1301, 1236, 1179, 1150, 1107, 1053, 1029, 969, 951, 875, 840, 816; ^{1}H NMR (400 MHz, $CDCl_3$) δ 7.53–7.51 (m, 2 H, 2 × Ar-CH), 6.99–6.96 (m, 2 H, 2 × Ar-CH), 4.95 (d, *J* = 6.9 Hz, 2 H, C*H*HOC*H*H), 4.93 (d, *J* = 6.9 Hz, 2 H, CH*H*OCH*H*), 3.86 (s, 3 H, CH_3), 2.43 (s, 1 H, OH); ^{13}C NMR (101 MHz, $CDCl_3$) δ 159.3 (Ar-C_q-O), 134.5 (Ar-C_q-C_q), 125.9 (2 × Ar-CH), 114.0 (2 × Ar-CH), 85.6 (2 × CH_2), 75.7 (C_q), 55.4 (CH_3). The observed spectroscopic data for this compound was consistent with that previously reported.[1]

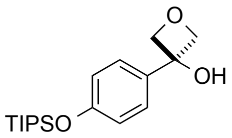

3-(4-{[Tris(propan-2-yl)silyl]oxy}phenyl)oxetan-3-ol (4)[1]

n-BuLi (2.24 M in THF, 4.46 mL, 10.0 mmol) was added dropwise over 5 min to a solution of 4-Bromophenoxytris(propan-2-yl)silane (3.29 g, 10.0 mmol) in THF (35 mL) at –78 °C. The reaction mixture was stirred at –78 °C for a further 10 min. Oxetan-3-one (0.49 mL, 7.70 mmol) in THF (2.5 mL) was added dropwise to the reaction mixture. Following a further 10 min at –78 °C the reaction mixture was warmed to rt then quenched with water (30 mL). The layers were separated and the aqueous portion extracted with diethylether (3 × 30 mL). The organic extracts were combined, washed with brine, dried over Na_2SO_4, filtered and concentrated *in vacuo*. Purification by flash column chromatography (30% EtOAc/hexane) afforded oxetanol **4** (1.96 g, 79%) as a white solid. R_f = 0.29 (30% EtOAc/hexane); mp = 63–64 °C; IR (film)/cm^{-1} 3404 (br. OH), 2945, 2868, 2852, 1605, 1513, 1461, 1421, 1251, 1171, 1104, 1076, 1009, 999, 977, 953, 908, 881, 843, 820, 738, 727, 688; ^{1}H NMR (400 MHz, $CDCl_3$) δ 7.44–7.40 (m, 2 H, 2 × Ar-CH), 6.94–6.90 (m, 2 H, 2 × Ar-CH), 4.93 (d, *J* = 7.0 Hz, 2 H, C*H*HOC*H*H), 4.90 (d, *J* = 7.0 Hz, 2 H, CH*H*OCH*H*), 2.41 (s, 1 H, OH), 1.31–1.22 (m, 3 H, 3 × CH), 1.11 (d, *J* = 7.4 Hz, 18 H, 6 × CH_3); ^{13}C NMR (101 MHz, $CDCl_3$) δ 155.8 (Ar-C_q-O), 134.7 (Ar-C_q-C_q), 125.8 (2 × Ar-CH), 119.9 (2 × Ar-CH), 85.5 (2 × CH_2), 75.7 (C_q), 17.9 (6 × CH_3), 12.6 (3 × CH). The observed spectroscopic data for this compound was consistent with that previously reported.[1]

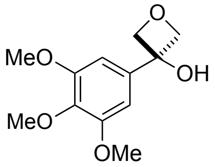

3-(3,4,5-Trimethoxyphenyl)oxetan-3-ol (5)

n-BuLi (2.40 M in THF, 1.0 mL, 2.4 mmol) was added dropwise over 5 min to a solution of 5-bromo-1,2,3-trimethoxybenzene (642 mg, 2.6 mmol) in THF (10 mL) at –78 °C. The reaction mixture was stirred at –78 °C for a further 10 min. Oxetan-3-one (0.14 mL, 2.0 mmol) was added dropwise to the reaction mixture. Following a further 5 min at –78 °C the reaction mixture was warmed to rt then quenched with water (40 mL). The layers were separated and the aqueous portion extracted with diethylether (3 × 30 mL). The organic extracts were combined, washed with brine, dried over Na_2SO_4, filtered and concentrated *in vacuo*. Purification by flash column chromatography (70% EtOAc/hexane) afforded oxetanol **5** (415 g, 86%) as a white solid. R_f = 0.23 (70% EtOAc/hexane); mp = 128–130 °C; IR (film)/cm^{-1} 3429 (br. OH), 2972, 2944, 1739, 1593, 1449, 1412, 1335, 1234, 1092, 1124, 996, 980, 850, 655; ^{1}H NMR (400 MHz, $CDCl_3$) δ 6.83 (s, 2 H, 2 × Ar-CH), 4.91 (s, 4 H, CH_2OCH_2), 3.91 (s, 6 H, 2 × OCH_3), 3.87 (s, 3 H, OCH_3), 2.58 (s, 1 H, OH); ^{13}C NMR (101 MHz, $CDCl_3$) δ 153.4 (2 × Ar-C_q-O), 138.1 (Ar-C_q-O), 137.5 (Ar-C_q-C_q), 101.7 (2 × Ar-CH), 85.7 (2 × CH_2), 75.8 (C_q), 60.8 (OCH_3), 56.2 (2 × OCH_3); FTMS (+p NSI) *m/z* calcd for $C_{12}H_{17}O_5^+$ $[M+H]^+$: 241.1071, Found: 241.1073.

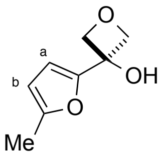

3-(5-Methylfuran-2-yl)oxetan-3-ol (6)[1]

n-BuLi (2.50 M in THF, 14.0 mL, 35.1 mmol) was added dropwise over 5 min to a solution of 2-methylfuran (3.00 g, 36.5 mmol) in THF (30 mL) at –10 °C. The reaction mixture was stirred at 0 °C for a further 1 h. Oxetan-3-one (2.04 mL, 31.9 mmol) in THF (15 mL) was added dropwise to the reaction mixture at –10 °C. Following a further 15 h at 15 °C the reaction mixture was warmed to rt then quenched with sat. aq NH_4Cl (10 mL). The layers were separated and the aqueous portion extracted with EtOAc (3 × 50 mL). The organic extracts were combined, dried over Na_2SO_4, filtered and concentrated *in vacuo*. Purification by flash column chromatography (0–50% EtOAc/Petroleum ether) afforded oxetanol **6** (2.30 g, 47%) as a colorless oil. R_f = 0.64 (50% Petroleum ether/EtOAc); IR (film)/cm^{-1} 3366 (br. OH), 2951, 2879, 1738, 1565, 1217, 1174, 1140, 1087, 1019, 970, 953, 942, 866, 784, 674; ^{1}H NMR (400 MHz, $CDCl_3$) δ 6.29 (d, *J* = 3.3 Hz, 1 H, Ar-CH_a), 6.01–5.98 (m, 1 H, Ar-CH_b), 4.93 (d, *J* = 6.5 Hz, 2 H, C*H*HOC*H*H), 4.84 (dd, *J* = 6.5, 1.3 Hz, 2 H, CH*H*OCH*H*), 2.74 (s, 1 H, OH), 2.33 (s, 3 H, CH_3); ^{13}C NMR (101 MHz, $CDCl_3$) δ 152.5 (Ar-C_q), 152.4, (Ar-C_q), 106.9 (Ar-CH), 106.4 (Ar-CH), 83.1 (CH_2OCH_2), 72.1 (C_q), 13.5 (CH_3). The observed spectroscopic data for this compound was consistent with that previously reported.[1]

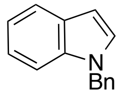

1-Benzyl-1*H*-indole[2]

Indole (1.17 g, 10.0 mmol) in DMF (10 mL) was added to a solution of sodium hydride (60% in mineral oil, 480 mg, 12.0 mmol) in DMF (10 mL) at 0 °C. The reaction mixture was stirred at 25 °C for 30 min then benzyl bromide (1.80 mL, 15.0 mmol) was added dropwise. The resulting solution was stirred at 25 °C for a further 12 h then quenched with water (20 mL). Dichloromethane (15 mL) was added and the layers separated. The organic extract was washed with water (3 × 20 mL), dried over Na_2SO_4, filtered and concentrated *in vacuo*. Purification by flash column chromatography (3% EtOAc/hexane) afforded 1-benzyl-1*H*-indole (1.90 g, 91%) as a pale yellow solid. R_f = 0.32 (3% EtOAc/hexane); mp = 39–41 °C [Lit. mp = 41 °C][3]; IR (film)/cm^{-1} 3025, 1738, 1508, 1485, 1463, 1455, 1440, 1354, 1336, 1316, 1178, 1078, 765, 740, 715, 695; ^{1}H NMR (400 MHz, $CDCl_3$) δ 7.70 (d, *J* = 7.7 Hz, 1 H, Ar-CH), 7.49–7.09 (m, 9 H, 9 × Ar-CH), 6.60 (dd, *J* = 2.9, 1.7 Hz, 1 H, $Ar_{(indole\text{-}3H)}$-CH), 5.37 (CH_2); ^{13}C NMR (101 MHz, $CDCl_3$) δ 137.5 (Ph-C_q), 136.3 ($Ar_{(indole)}$-C_q), 128.7 (2 × $Ar_{(Bn)}$-CH), 128.4 ($Ar_{(indole)}$-C_q), 128.2 ($Ar_{(indole)}$-CH), 127.6 (Ph-CH), 126.7 (2 × $Ar_{(Bn)}$-CH), 121.7 ($Ar_{(indole)}$-CH), 120.9 ($Ar_{(indole)}$-CH), 119.6 ($Ar_{(indole)}$-CH), 109.7 ($Ar_{(indole)}$-CH), 101.7 ($Ar_{(indole)}$-CH), 50.1 (Ph-CH_2). The observed spectroscopic data for this compound was consistent with that previously reported.[4]

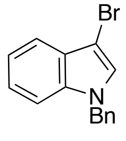

1-Benzyl-3-bromo-1*H*-indole[5]

1,3-Dibromo-5,5-dimethylhydantoin (786 mg, 2.75 mmol) was added portionwise over 10 min to a solution of 1-benzyl-1*H*-indole (1.04 g, 5.00 mmol) in 1,4-dioxane (17 mL) at 10 °C. The resulting solution was stirred for 5 min and then sat. aq. $NaHCO_3$ (30 mL) was added followed by EtOAc (15 mL). The layers were separated and the aqueous portion was extracted with EtOAc (3 × 15 mL). The organic extract was dried over Na_2SO_4, filtered and concentrated *in vacuo*. Purification by flash column chromatography (100% hexane) afforded 1-benzyl-3-bromo-1*H*-indole (877 mg, 61%) as a pink oil. R_f = 0.27 (30% EtOAc/hexane); IR (film)/cm^{-1} 3253, 2963, 1739, 1609, 1500, 1464, 1382, 1270, 1252, 1230, 1125, 1062, 1015, 978, 939, 823, 739, 724, 694; ^{1}H NMR (400 MHz, $CDCl_3$) δ 7.62 (d, *J* = 6.9 Hz, 1 H, Ar-CH), 7.48–7.02 (m, 9 H, 9 × Ar-CH), 5.31 (s, 2 H, CH_2); ^{13}C NMR (101 MHz, $CDCl_3$) δ 136.7 (Ph-C_q), 135.9 ($Ar_{(indole)}$-C_q), 128.9 (2 × $Ar_{(Bn)}$-CH), 127.8 (Ph-CH), 127.5 ($Ar_{(indole)}$-C_q), 127.0 ($Ar_{(indole)}$-CH), 126.9 (2 × $Ar_{(Bn)}$-CH), 122.8 ($Ar_{(indole)}$-CH), 120.3 ($Ar_{(indole)}$-CH), 119.4 ($Ar_{(indole)}$-CH), 109.9 ($Ar_{(indole)}$-CH), 90.2 ($Ar_{(indole)}$-C_q-Br), 50.1 ($PhCH_2$). The observed spectroscopic data for this compound was consistent with that previously reported.[6]

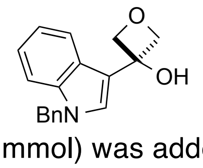

3-(1-Benzyl-1H-indol-3-yl)oxetan-3-ol (7)[1]

n-BuLi (2.45 M in THF, 0.97 mL, 2.40 mmol) was added dropwise over 5 min to a solution of 1-benzyl-3-bromo-1*H*-indole (859 mg, 3.00 mmol) in THF (15 mL) at –78 °C. The reaction mixture was stirred at –78 °C for a further 5 min. Oxetan-3-one (0.13 mL, 2.00 mmol) was added dropwise to the reaction mixture. Following a further 30 min at –78 °C the reaction mixture

was warmed to rt then quenched with water (15 mL). The layers were separated and the aqueous portion extracted with diethyl ether (3 × 30 mL). The organic extracts were combined, washed with brine (30 mL), dried over Na_2SO_4, filtered and concentrated *in vacuo*. Purification by flash column chromatography (40% EtOAc/hexane) afforded oxetanol **7** (504 mg, 90%) as a beige solid. R_f = 0.19 (40% EtOAc/hexane); mp = 118–120 °C; IR (film)/cm^{-1} 3278 (br. OH), 2986, 2876, 1739, 1553, 1496, 1467, 1451, 1243, 1198, 1179, 1140, 1087, 1071, 956, 863, 830, 742, 695; ^{1}H NMR (400 MHz, $CDCl_3$) δ 7.80 (dt, *J* = 7.9, 1.0 Hz, 1 H, Ar-CH), 7.36–7.14 (m, 9 H, 9 × Ar-CH), 5.34 (s, 2 H, CH_2Ph), 5.09 (d, *J* = 6.9 Hz, 2 H, C*H*HOC*H*H), 5.03 (d, *J* = 6.9 Hz, 2 H, CH*H*OCH*H*), 2.49 (s, 1 H, OH); ^{13}C NMR (101 MHz, $CDCl_3$) δ 137.4 (Ph-C_q), 136.9 ($Ar_{(indole)}$-C_q), 128.9 (2 × Ar-CH), 127.8 (Ar-CH), 126.9 (2 × Ar-CH), 125.5 ($Ar_{(indole)}$-C_q), 125.2 (Ar-CH), 122.5 (Ar-CH), 120.0 (Ar-CH), 119.9 (Ar-CH), 117.1 (Ar-C_q-C_q), 110.1 (Ar-CH), 84.7 (CH_2OCH_2), 73.1 (C_q), 50.1 ($PhCH_2$). The observed spectroscopic data for this compound was consistent with that previously reported.[1]

Synthesis of Oxetane-3-sulfides 2a–m, 8–11

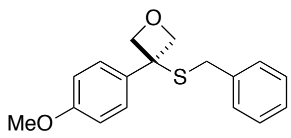

3-(Benzylthio)-3-(4-methoxyphenyl)oxetane (2a)

Lithium bis(trifluoromethanesulfonimide) (31.6 mg, 0.11 mmol) and tetrabutylammonium hexafluorophosphate (21.3 mg, 0.055 mmol) were added to a solution of oxetanol **1** (180 mg, 1.0 mmol) and benzylmercaptan (0.23 mL, 2.0 mmol) in chloroform (2.0 mL). The reaction mixture was stirred at 40 °C for 25 min then quenched with sat. aq. $NaHCO_3$ (10 mL). The layers were separated and the aqueous portion extracted with dichloromethane (3 × 5 mL). The organic extracts were combined then dried over Na_2SO_4, filtered and concentrated *in vacuo*. Purification by flash column chromatography (15% Et_2O/pentane) afforded oxetane **2a** (191 mg, 67%) as a colorless oil. R_f = 0.17 (15% Et_2O/pentane); IR (film)/cm^{-1} 2948, 2870, 1739, 1610, 1511, 1495, 1453, 1301, 1244, 1179, 1026, 982, 829, 697; ^{1}H NMR (400 MHz, $CDCl_3$) δ 7.34–7.23 (m, 5 H, 5 × Ph-CH), 7.22–7.13 (m, 2 H, 2 × $Ar_{(p\text{-OMe})}$-CH), 7.00–6.87 (m, 2 H, 2 × $Ar_{(p\text{-OMe})}$-CH), 5.09 (d, *J* = 6.4 Hz, 2 H, C*H*HOC*H*H), 4.80 (d, *J* = 6.4 Hz, 2 H, CH*H*OCH*H*), 3.85 (s, 3 H, OCH_3), 3.61 (s, 2 H, CH_2Ph); ^{13}C NMR (101 MHz, $CDCl_3$) δ 158.7 (Ar-C_q-OMe), 137.6 (Ph-C_q), 134.3 (Ar-C_q-C_q), 129.0 (2 × Ph-CH), 128.5 (2 × Ph-CH), 127.7 (2 × $Ar_{(p\text{-OMe})}$-CH), 127.1 (Ph-CH), 113.9 (2 × $Ar_{(p\text{-OMe})}$-CH), 82.9 (CH_2OCH_2), 55.3 (OCH_3), 52.6 (C_q), 35.1 (CH_2Ph); HRMS (TOF-ASAP+) *m/z* calcd for $C_{17}H_{17}SO^+$ $[M+H-H_2O]^+$: 269.1000, Found: 269.1004.

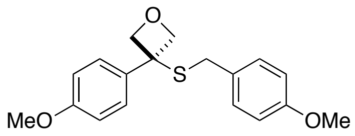

3-((4-Methoxybenzyl)thio)-3-(4-methoxyphenyl)oxetane (2b)

Lithium bis(trifluoromethanesulfonimide) (47.4 mg, 0.165 mmol) and tetrabutylammonium hexafluorophosphate (32.0 mg, 0.0825 mmol) were added to a solution of oxetanol **1** (270 mg, 1.5 mmol) and (4-methoxyphenyl)methanethiol (0.42 mL, 3.0 mmol) in chloroform (3.0 mL). The reaction mixture was stirred at 40 °C for 25 min then quenched with sat. aq. $NaHCO_3$ (10 mL). The layers were separated and the aqueous portion extracted with dichloromethane (3 × 5 mL). The organic extracts were combined then dried over Na_2SO_4, filtered and concentrated *in vacuo*. Purification by flash column chromatography (20% pentane/CH_2Cl_2) afforded oxetane **2b** (190 mg, 60%) as a colorless oil. R_f = 0.31 (20% pentane/CH_2Cl_2); IR (film)/cm^{-1} 2870, 2950, 2835, 1739, 1609, 1509, 1241, 1175, 1027, 982, 827; ^{1}H NMR (400 MHz, $CDCl_3$) δ 7.20–7.14 (m, 4 H, 4 × Ar-CH), 6.96–6.90 (m, 2 H, 2 × Ar-CH), 6.85–6.80 (m, 2 H, 2 × Ar-CH), 5.09 (d, *J* = 6.4 Hz, 2 H, C*H*HOC*H*H), 4.81 (d, *J* = 6.4 Hz, 2 H, CH*H*OCH*H*), 3.85 (s, 3 H, OCH_3), 3.79 (s, 3 H, OCH_3), 3.56 (s, 2 H, CH_2Ph); ^{13}C NMR (101 MHz, $CDCl_3$) δ 158.64 (Ar-C_q-OMe), 158.55 (Ar-C_q-OMe), 134.3 (Ar-C_q-C_q), 130.0 (2 × Ar-CH), 129.3 (Ar-C_q-CH_2), 127.7 (2 × Ar-CH), 113.9 (4 × Ar-CH), 82.9 (CH_2OCH_2), 55.3 (OCH_3), 55.2 (OCH_3), 52.5 (C_q), 34.4 (SCH_2); HRMS (TOF-ASAP+) *m/z* calcd for $C_{18}H_{19}SO_2^+$ $[M+H-H_2O]^+$: 299.1106, Found: 299.1108.

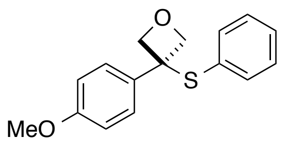

3-(4-Methoxyphenyl)-3-(phenylsulfanyl)oxetane (2c)

Lithium bis(trifluoromethanesulfonimide) (47.4 mg, 0.165 mmol) and tetrabutylammonium hexafluorophosphate (32.0 mg, 0.0825 mmol) were added to a solution of oxetanol **1** (270 mg, 1.5 mmol) and thiophenol (0.31 mL, 3.0 mmol) in chloroform (3.0 mL). The reaction mixture was stirred at 40 °C for 25 min then quenched with sat. aq. $NaHCO_3$ (10 mL). The layers were separated and the aqueous portion extracted with dichloromethane (3 × 5 mL). The organic extracts were combined, washed with aq. NaOH (1 M, 15 mL) then dried over Na_2SO_4, filtered and concentrated *in vacuo*. Purification by flash column chromatography (15% Et_2O/pentane) afforded oxetane **2c** (340 mg, 83%) as a white solid. R_f = 0.15 (15% Et_2O/pentane); mp = 97–99 °C; IR (film)/cm^{-1} 2959, 1739, 1609, 1513, 1252, 1175, 1022, 982, 818, 756, 695; ^{1}H NMR (400 MHz, $CDCl_3$) δ 7.32–7.19 (m, 5 H, 5 × Ph-CH), 6.98–6.93 (m, 2 H, 2 × $Ar_{(p\text{-OMe})}$-CH), 6.84–6.79 (m, 2 H, 2 × $Ar_{(p\text{-OMe})}$-CH), 5.13 (d, *J* = 6.4 Hz, 2 H, C*H*HOC*H*H), 4.96 (d, *J* = 6.4 Hz, 2 H, CH*H*OCH*H*), 3.81 (s, 3 H, OCH_3); ^{13}C NMR (101 MHz, $CDCl_3$) δ 158.6 (Ar-C_q-OMe), 135.2 (2 × Ph-CH), 134.7 (Ar-C_q-C_q), 132.1 (Ar-C_q-S), 128.7 (3 × Ph-CH), 127.7 (2 × $Ar_{(p\text{-OMe})}$-CH), 113.6 (2 × $Ar_{(p\text{-OMe})}$-CH), 82.6 (CH_2OCH_2), 55.3 (OCH_3), 54.8 (C_q); HRMS (TOP-ASAP+) *m/z* calcd for $C_{16}H_{15}SO^+$ $[M+H-H_2O]^+$: 255.0844, Found: 255.0845.

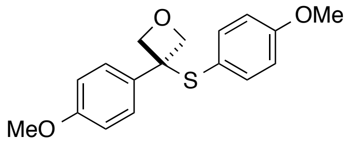

3-(4-Methoxyphenyl)-3-[(4-methoxyphenyl)sulfanyl]oxetane (2d)

Lithium bis(trifluoromethanesulfonimide) (47.4 mg, 0.165 mmol) and tetrabutylammonium hexafluorophosphate (32.0 mg, 0.0825 mmol) were added to a solution of oxetanol **1** (270 mg, 1.5 mmol) and 4-methoxythiophenol (0.37 mL, 3.0 mmol) in chloroform (3.0 mL). The reaction mixture was stirred at 40 °C for 25 min then quenched with sat. aq. $NaHCO_3$ (10 mL). The layers were separated and the aqueous portion extracted with dichloromethane (3 × 5 mL). The organic extracts were combined, washed with aq. NaOH (1 M, 15 mL) then dried over Na_2SO_4, filtered and concentrated *in vacuo*. Purification by flash column chromatography (25% Et_2O/pentane) afforded oxetane **2d** (400 mg, 88%) as a white solid. R_f = 0.18 (25% Et_2O/pentane); mp = 113–116 °C; IR (film)/cm^{-1} 2956, 2882, 2837, 1740, 1611, 1585, 1511, 1492, 1456, 1440, 1298, 1284, 1237, 1171, 1021, 976, 823, 810; ^{1}H NMR (400 MHz, $CDCl_3$) δ 7.20–7.16 (m, 2 H, 2 × Ar-CH), 6.91–6.90 (m, 2 H, 2 × Ar-CH), 6.87–6.76 (m, 4 H, 4 × Ar-CH), 5.11 (d, *J* = 6.5 Hz, 2 H, C*H*HOC*H*H), 4.94 (d, *J* = 6.5 Hz, 2 H, CH*H*OCH*H*), 3.81 (s, 3 H, OCH_3), 3.80 (s, 3 H, OCH_3); ^{13}C NMR (101 MHz, $CDCl_3$) δ 160.6 ($Ar_{(p\text{-S})}$-C_q-OMe) 158.4 (Ar-C_q-OMe), 137.9 (2 × $Ar_{(p\text{-OMe})}$-CH), 135.0 (Ar-C_q-C_q), 127.6 (2 × $Ar_{(p\text{-OMe})}$-CH), 122.5 (Ar-C_q-S), 114.2 (2 × $Ar_{(p\text{-OMe})}$-CH), 113.5 (2 × $Ar_{(p\text{-OMe})}$-CH), 82.2 (CH_2OCH_2), 55.3 (2 × OCH_3), 54.9 (C_q); HRMS (TOF-ASAP+) *m/z* calcd for $C_{17}H_{17}SO_2^+$ $[M+H-H_2O]^+$: 285.0949, Found: 285.0952.

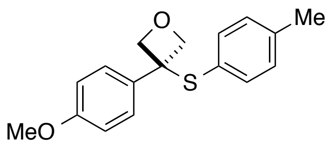

3-(4-Methoxyphenyl)-3-[(4-methylphenyl)sulfanyl]oxetane (2e)

Lithium bis(trifluoromethanesulfonimide) (31.6 mg, 0.11 mmol) and tetrabutylammonium hexafluorophosphate (21.3 mg, 0.055 mmol) were added to a solution of oxetanol **1** (180 mg, 1.0 mmol) and 4-methylthiophenol (248 mg, 2.0 mmol) in chloroform (2.0 mL). The reaction mixture was stirred at 40 °C for 25 min then quenched with sat. aq. $NaHCO_3$ (10 mL). The layers were separated and the aqueous portion extracted with dichloromethane (3 × 5 mL). The organic extracts were combined, washed with aq. NaOH (1 M, 15 mL) then dried over Na_2SO_4, filtered and concentrated *in vacuo*. Purification by flash column chromatography (15% Et_2O/pentane) afforded oxetane **2e** (250 mg, 87%) as a white solid. R_f = 0.21 (15% Et_2O/pentane); mp = 98–100 °C; IR (film)/cm^{-1} 2947, 2877, 1615, 1515, 1454, 1308, 1258, 1175, 1025, 978, 814; ^{1}H NMR (400 MHz, $CDCl_3$) δ 7.14–7.11 (m, 2 H, 2 × $Ar_{(p\text{-Me})}$-CH), 7.07–7.04 (m, 2 H, 2 × $Ar_{(p\text{-Me})}$-CH), 6.98–6.92 (m, 2 H, 2 × $Ar_{(p\text{-OMe})}$-CH), 6.84–6.81 (m, 2 H, 2 × $Ar_{(p\text{-OMe})}$-CH), 5.11 (d, *J* = 6.4 Hz, 2 H, C*H*HOC*H*H), 4.96 (d, *J* = 6.4 Hz, 2 H, CH*H*OCH*H*), 3.82 (s, 3 H, OCH_3), 2.33 (CH_3); ^{13}C NMR (101 MHz, $CDCl_3$) δ 158.5 (Ar-C_q-OMe), 139.1 (Ar-C_q-CH_3), 135.7 (2 × $Ar_{(p\text{-Me})}$-CH), 135.0 (Ar-C_q-C_q), 129.6 (2 × $Ar_{(p\text{-Me})}$-CH), 128.4 (Ar-C_q-S), 127.7 (2 × $Ar_{(p\text{-OMe})}$-CH), 113.6 (2 × $Ar_{(p\text{-OMe})}$-CH), 82.5 (CH_2OCH_2), 55.3 (OCH_3), 54.8 (C_q), 21.2 (CH_3); HRMS (TOF-ASAP+) *m/z* calcd for $C_{17}H_{17}SO_2^+$ $[M+H-H_2O]^+$: 269.1000, Found: 269.1000.

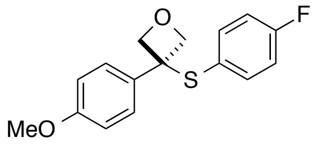

3-[(4-Fluorophenyl)sulfanyl]-3-(4-methoxyphenyl)oxetane (2f)

Lithium bis(trifluoromethanesulfonimide) (47.4 mg, 0.165 mmol) and tetrabutylammonium hexafluorophosphate (32.0 mg, 0.0825 mmol) were added to a solution of oxetanol **1** (270 mg, 1.5 mmol) and 4-fluorothiophenol (0.32 mL, 3.0 mmol) in chloroform (3.0 mL). The reaction mixture was stirred at 40 °C for 25 min then quenched with sat. aq. $NaHCO_3$ (10 mL). The layers were separated and the aqueous portion extracted with dichloromethane (3 × 5 mL). The organic extracts were combined, washed with aq. NaOH (1 M, 15 mL) then dried over Na_2SO_4, filtered and concentrated *in vacuo*. Purification by flash column chromatography (15% Et_2O/pentane) afforded oxetane **2f** (358 mg, 82%) as a white solid. R_f = 0.25 (15% Et_2O/pentane); mp = 69–71 °C; IR (film)/cm^{-1} 2959, 2882, 1740, 1611, 1588, 1512, 1484, 1459, 1305, 1245, 1223, 1177, 1026, 983, 825; ^{1}H NMR (400 MHz, $CDCl_3$) δ 7.20–7.14 (m, 2 H, 2 × $Ar_{(p\text{-F})}$-CH), 6.97–6.89 (m, 2 H, 2 × $Ar_{(p\text{-F})}$-CH), 6.88–6.85 (m, 2 H, 2 × $Ar_{(p\text{-OMe})}$-CH), 6.84–6.80 (m, 2 H, 2 × $Ar_{(p\text{-OMe})}$-CH), 5.13 (d, *J* = 6.5 Hz, 2 H, C*H*HOC*H*H), 4.94 (d, *J* = 6.5 Hz, 2 H, CH*H*OCH*H*), 3.81 (s, 3 H, OCH_3); ^{13}C NMR (101 MHz, $CDCl_3$) δ 163.5 (d, *J* = 253.8 Hz, Ar-C_q-F), 158.6 (Ar-C_q-OMe), 137.9 (d, *J* = 8.4 Hz, 2 × $Ar_{(p\text{-F})}$-CH), 134.5 (Ar-C_q-C_q), 127.6 (2 × $Ar_{(p\text{-OMe})}$-CH), 127.1 (d, *J* = 3.4 Hz, Ar-C_q-S), 115.9 (d, *J* = 21.7 Hz, 2 × $Ar_{(p\text{-F})}$-CH), 113.6 (2 × $Ar_{(p\text{-OMe})}$-CH), 82.4 (CH_2OCH_2), 55.3 (OCH_3), 55.1 (C_q); ^{19}F NMR (377 MHz, $CDCl_3$) δ –111.7; HRMS (TOF-ASAP+) *m/z* calcd for $C_{16}H_{15}SFO^+$ $[M+H-H_2O]^+$: 273.0749, Found: 273.0751.

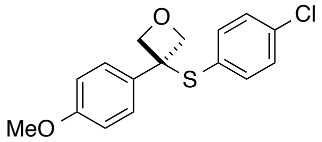

3-[(4-Chlorophenyl)sulfanyl]-3-(4-methoxyphenyl)oxetane (2g)

Lithium bis(trifluoromethanesulfonimide) (47.4 mg, 0.165 mmol) and tetrabutylammonium hexafluorophosphate (32.0 mg, 0.0825 mmol) were added to a solution of oxetanol **1** (270 mg, 1.5 mmol) and 4-chlorothiophenol (434 mg, 3.0 mmol) in chloroform (3.0 mL). The reaction mixture was stirred at 40 °C for 25 min then quenched with sat. aq. $NaHCO_3$ (10 mL). The layers were separated and the aqueous portion extracted with dichloromethane (3 × 5 mL). The organic extracts were combined, washed with aq. NaOH (1 M, 15 mL) then dried over Na_2SO_4, filtered and concentrated *in vacuo*. Purification by flash column chromatography (15% Et_2O/pentane) afforded oxetane **2g** (378 mg, 82%) as a white solid. R_f = 0.21 (15% Et_2O/pentane); mp = 103–105 °C; IR (film)/cm^{-1} 2947, 2878, 1739, 1514, 1454, 1366, 1230, 1217, 1176, 1092, 1024, 1013, 974, 823; ^{1}H NMR (400 MHz, $CDCl_3$) δ 7.23–7.18 (m, 2 H, 2 × $Ar_{(p\text{-Cl})}$-CH), 7.12–7.09 (m, 2 H, 2 × $Ar_{(p\text{-Cl})}$-CH), 6.96–6.90 (m, 2 H, 2 × $Ar_{(p\text{-OMe})}$-CH), 6.85–6.80 (m, 2 H, 2 × $Ar_{(p\text{-OMe})}$-CH), 5.13 (d, *J* = 6.5 Hz, 2 H, C*H*HOC*H*H), 4.94 (d, *J* = 6.5 Hz, 2 H, CH*H*OCH*H*), 3.82 (s, 3 H, OCH_3); ^{13}C NMR (101 MHz, $CDCl_3$) δ 158.7 (Ar-C_q-OMe), 136.5 (2 × $Ar_{(p\text{-Cl})}$-CH), 135.3 (Ar-C_q-Cl), 134.3 (Ar-C_q-C_q), 130.5 (Ar-C_q-S), 128.9 (2 × $Ar_{(p\text{-Cl})}$-CH), 127.7 (2 × $Ar_{(p\text{-OMe})}$-CH), 113.7 (2 × $Ar_{(p\text{-OMe})}$-CH), 82.5 (CH_2OCH_2), 55.3 (OCH_3), 55.1 (C_q); HRMS (TOF-ASAP+) *m/z* calcd for $C_{16}H_{14}SOCl^+$ $[M+H-H_2O]^+$: 289.0454, Found: 289.0451.

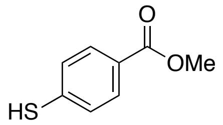

Methyl-4-mercaptobenzoate (S1)

Concentrated H_2SO_4 (2 drops) was added to a stirred solution of 4-mercaptobenzoic acid (771 mg, 5.0 mmol) in MeOH (4.0 mL). The reaction mixture was stirred for 6 h at reflux then cooled to rt and concentration *in vacuo*. The residue was taken up in EtOAc (15 mL) and washed with water (2 × 15 mL) then sat. aq. $NaHCO_3$ (15 mL). The organic portions were combined, dried over Na_2SO_4 and concentrated *in vacuo*. Purification by flash column chromatography (60% Et_2O/pentane) afforded thiol **S1** (553 mg, 66%) as a pale yellow solid. R_f = 0.42 (60% Et_2O/pentane); mp = 53–55 °C; IR (film)/cm^{-1} 2960, 2553, 1717 (C=O), 1595, 1433, 1278, 1179, 1111, 1014, 952, 842, 829, 757, 690; ^{1}H NMR (400 MHz, $CDCl_3$) δ 7.92–7.86 (m, 2 H, 2 × Ar-CH), 7.32–7.25 (m, 2 H, 2 × Ar-CH), 3.90 (s, 3 H, CH_3), 3.61 (s, 1 H, SH); ^{13}C NMR (101 MHz, $CDCl_3$) δ 166.6 (C(O)OMe), 138.3 (Ar-C_q-C(O)OMe), 130.2 (2 × Ar-CH), 128.1 (2 × Ar-CH), 127.1 (Ar-C_q-SH), 52.1 (CH_3). The observed spectroscopic data for this compound was consistent with that previously reported.[7]

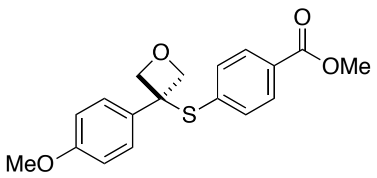

Methyl 4-((3-(4-methoxyphenyl)oxetan-3-yl)thio)benzoate (2h)

Lithium bis(trifluoromethanesulfonimide) (31.6 mg, 0.11 mmol) and tetrabutylammonium hexafluorophosphate (21.3 mg, 0.055 mmol) were added to a solution of oxetanol **1** (180.2 mg, 1.0 mmol) and methyl-4-mercaptobenzoate (336.4 mg, 2.0 mmol) in chloroform (2.0 mL). The reaction mixture was stirred at 40 °C for 25 min then quenched with sat. aq. $NaHCO_3$ (10 mL). The layers were separated and the aqueous portion extracted with dichloromethane (3 × 5 mL). The organic extracts were combined, washed with aq. NaOH (1 M, 15 mL) then dried over Na_2SO_4, filtered and concentrated *in vacuo*. Purification by flash column chromatography (31:65:4 Et_2O/pentane/Et_3N) afforded oxetane **2h** (267 mg, 81%) as a pale yellow solid. R_f = 0.30 (31:65:4 Et_2O/pentane/Et_3N); mp = 106–108 °C; IR (film)/cm^{-1} 2951, 1719 (C=O), 1615, 1516, 1425, 1300, 1259, 1175, 1115, 1017, 980, 817, 764, 697; ^{1}H NMR (400 MHz, $CDCl_3$) δ 7.89–7.85 (m, 2 H, 2 × $Ar_{(p\text{-CO2Me})}$-CH), 7.19–7.09 (m, 4 H, 2 × $Ar_{(p\text{-CO2Me})}$-CH + 2 × $Ar_{(p\text{-OMe})}$-CH), 6.87–6.81 (m, 2 H, 2 × $Ar_{(p\text{-OMe})}$-CH), 5.16 (d, *J* = 6.5 Hz, 2 H, C*H*HOC*H*H), 4.99 (d, *J* = 6.5 Hz, 2 H, CH*H*OCH*H*), 3.90 (C(O)OCH_3), 3.81 (s, 3 H, ArOCH_3); ^{13}C NMR (101 MHz, $CDCl_3$) δ 166.5 (C(O)OMe),158.8 (Ar-C_q-OMe), 139.0 (Ar-C_q-C(O)OMe), 133.8 (Ar-C_q-C_q), 132.4 (2 × $Ar_{(p\text{-CO2Me})}$-CH), 129.7 (2 × $Ar_{(p\text{-CO2Me})}$-CH), 129.3 (Ar-C_q-S), 127.6 (2 × $Ar_{(p\text{-OMe})}$-CH), 113.8 (2 × $Ar_{(p\text{-OMe})}$-CH), 83.1 (CH_2OCH_2), 55.3 (OCH_3), 54.7 (C_q), 52.2 (C(O)OCH_3); HRMS (TOF-ASAP+) *m/z* calcd for $C_{18}H_{17}SO_3^+$ $[M+H-H_2O]^+$: 313.0898, Found: 313.0894.

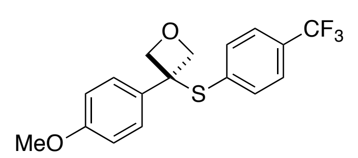

3-(4-Methoxyphenyl)-3-((4-(trifluoromethyl)phenyl)thio)oxetane (2i)

Lithium bis(trifluoromethanesulfonimide) (47.4 mg, 0.165 mmol) and tetrabutylammonium hexafluorophosphate (32.0 mg, 0.0825 mmol) were added to a solution of oxetanol **1** (270 mg, 1.5 mmol) and 4-(trifluoromethyl)thiophenol (0.41 mL, 3.0 mmol) in chloroform (3.0 mL). The reaction mixture was stirred at 40 °C for 25 min then quenched with sat. aq. $NaHCO_3$ (10 mL). The layers were separated and the aqueous portion extracted with dichloromethane (3 × 5 mL). The organic extracts were combined, washed with aq. NaOH (1 M, 15 mL) then dried over Na_2SO_4, filtered and concentrated *in vacuo*. Purification by flash column chromatography (20% Et_2O/pentane) afforded oxetane **2i** (378 mg, 74%) as a colorless oil. R_f = 0.26 (20% Et_2O/pentane); IR (film)/cm^{-1} 2953, 2874, 1739, 1607, 1512, 1321, 1245, 1164, 1120, 1100, 1088, 1062, 827; ^{1}H NMR (400 MHz, $CDCl_3$) δ 7.48–7.44 (m, 2 H, 2 × $Ar_{(p\text{-}CF3)}$-CH), 7.24–7.20 (m, 2 H, 2 × $Ar_{(p\text{-}CF3)}$-CH), 7.11–7.05 (m, 2 H, 2 × $Ar_{(p\text{-}OMe)}$-CH), 6.87–6.82 (m, 2 H, 2 × $Ar_{(p\text{-}OMe)}$-CH), 5.17 (d, *J* = 6.6 Hz, 2 H, C*H*HOC*H*H), 4.97 (d, *J* = 6.6 Hz, 2 H, CH*H*OCH*H*), 3.81 (s, 3 H, OCH_3); ^{13}C NMR (101 MHz, $CDCl_3$) δ 158.8 (Ar-C_q-OMe), 137.5 (Ar-C_q-C_q),133.7 (Ar-C_q-S), 133.4 (2 × $Ar_{(p\text{-}CF3)}$-CH), 130.0 (q, $J_{C\text{-}F}$ = 32.9 Hz, Ar-C_q-CF_3), 127.6 (2 × $Ar_{(p\text{-}OMe)}$-CH), 125.5 (q, $J_{C\text{-}F}$ = 3.9 Hz, 2 × $Ar_{(p\text{-}CF3)}$-CH), 123.9 (q, $J_{C\text{-}F}$ = 271.7 Hz, CF_3), 113.8 (2 × $Ar_{(p\text{-}OMe)}$-CH), 82.9 (CH_2OCH_2), 55.3 (OCH_3), 54.8 (C_q); ^{19}F NMR (377 MHz, $CDCl_3$) δ –62.7; HRMS (TOF-ASAP+) *m/z* calcd for $C_{17}H_{14}SOF_3^+$ $[M+H-H_2O]^+$: 323.0717, Found: 323.0725.

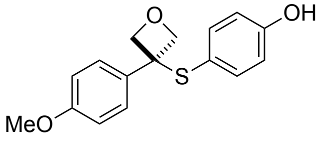

3-(4-Methoxyphenyl)-3-((4-methoxyphenyl)thio)oxetane (2j)

Lithium bis(trifluoromethanesulfonimide) (15.8 mg, 0.055 mmol) and tetrabutylammonium hexafluorophosphate (10.6 mg, 0.0275 mmol) were added to a solution of oxetanol **1** (90 mg, 0.5 mmol) and 4-hydroxythiophenol (126 mg, 1.0 mmol) in chloroform (1.0 mL). The reaction mixture was stirred at 40 °C for 25 min then quenched with sat. aq. $NaHCO_3$ (10 mL). The layers were separated and the aqueous portion extracted with EtOAc (5 × 10 mL). The organic extracts were combined, dried over Na_2SO_4, filtered and concentrated *in vacuo* affording oxetane **2j** (90 mg, 62%) as a white solid. R_f = 0.22 (40% EtOAc/hexane); mp = 226–228 °C; IR (film)/cm^{-1} 3221 (br. OH), 2944, 1611, 1597, 1575, 1512, 1444, 1267, 1246, 1216, 1179, 1164, 1024, 968, 929, 833, 818, 773; ^{1}H NMR (400 MHz, $(CD_3)_2SO$) δ 9.82 (s, 1 H, OH), 7.15–7.10 (m, 2 H, 2 × Ar-CH), 7.00–6.95 (m, 2 H, 2 × Ar-CH), 6.89–6.84 (m, 2 H, 2 × Ar-CH), 6.73–6.68 (m, 2 H, 2 × Ar-CH), 4.98 (d, *J* = 6.6 Hz, 2 H, C*H*HOC*H*H), 4.82 (d, *J* = 6.6 Hz, 2 H, CH*H*OCH*H*), 3.75 (s, 3 H, OCH_3); ^{13}C NMR (101 MHz, $(CD_3)_2SO$) δ 159.2 (Ar-C_q-OH) 158.5 (Ar-C_q-OMe), 138.3 (2 × Ar-CH), 135.0 ($Ar_{(p\text{-}OMe)}$-C_q-C_q), 128.2 (2 × Ar-CH), 120.5 (Ar-C_q-S), 116.4 (2 × Ar-CH), 114.0 (2 × Ar-CH), 81.6 (CH_2OCH_2), 55.6 (OCH_3), 54.9 (C_q); HRMS (TOF-ASAP+) *m/z* calcd for $C_{16}H_{17}SO_3^+$ $[M+H]^+$: 289.0898, Found: 289.0896.

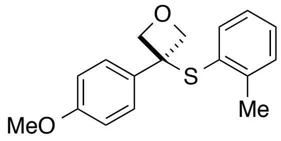

3-(4-Methoxyphenyl)-3-[(2-methylphenyl)sulfanyl]oxetane (2k)

Lithium bis(trifluoromethanesulfonimide) (47.4 mg, 0.165 mmol) and tetrabutylammonium hexafluorophosphate (32.0 mg, 0.0825 mmol) were added to a solution of oxetanol **1** (270 mg, 1.5 mmol) and 2-methylthiophenol (0.35 mL, 3.0 mmol) in chloroform (3.0 mL). The reaction mixture was stirred at 40 °C for 25 min then quenched with sat. aq. $NaHCO_3$ (10 mL). The layers were separated and the aqueous portion extracted with dichloromethane (3 × 5 mL). The organic extracts were combined, washed with aq. NaOH (1 M, 15 mL) then dried over Na_2SO_4, filtered and concentrated *in vacuo*. Purification by flash column chromatography (15% Et_2O/pentane) afforded oxetane **2k** (340 mg, 79%) as a white solid. R_f = 0.28 (15% Et_2O/pentane); mp = 90–91 °C; IR (film)/cm^{-1} 2952, 1739, 1509, 1455, 1366, 1230, 1217, 1177, 1022, 977, 804, 777; ^{1}H NMR (400 MHz, $CDCl_3$) δ 7.22–7.15 (m, 3 H, 3 × $Ar_{(o\text{-}Me)}$-CH), 7.09–7.05 (m, 1 H, $Ar_{(o\text{-}Me)}$-CH), 7.04–6.99 (m, 2 H, 2 × $Ar_{(p\text{-}OMe)}$-CH), 6.85–6.79 (m, 2 H, 2 × $Ar_{(p\text{-}OMe)}$-CH), 5.14 (d, *J* = 6.4 Hz, 2 H, C*H*HOC*H*H), 5.04 (d, *J* = 6.4 Hz, 2 H, CH*H*OCH*H*), 3.82 (s, 3 H, OCH_3), 2.16 (CH_3); ^{13}C NMR (101 MHz, $CDCl_3$) δ 158.6 (Ar-C_q-OMe), 141.9 (Ar-C_q-CH_3), 134.8 ($Ar_{(o\text{-}Me)}$-CH), 134.7 (Ar-C_q-C_q), 131.8 (Ar-C_q-S), 130.4 ($Ar_{(o\text{-}Me)}$-CH), 128.4 ($Ar_{(o\text{-}Me)}$-CH), 127.5 (2 × $Ar_{(p\text{-}OMe)}$-CH), 126.2 ($Ar_{(o\text{-}Me)}$-CH), 113.6 (2 × $Ar_{(p\text{-}OMe)}$-CH), 83.1 (CH_2OCH_2), 55.3 (OCH_3), 54.8 (C_q), 20.7 (CH_3); HRMS (TOF-ASAP+) *m/z* calcd for $C_{17}H_{18}SO^+$ $[M+H-H_2O]^+$: 269.1000, Found: 269.0998.

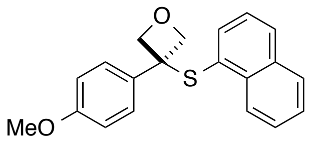

3-(4-Methoxyphenyl)-3-(naphthalen-1-ylsulfanyl)oxetane (2l)

Lithium bis(trifluoromethanesulfonimide) (47.4 mg, 0.165 mmol) and tetrabutylammonium hexafluorophosphate (32.0 mg, 0.0825 mmol) were added to a solution of oxetanol **1** (270 mg, 1.5 mmol) and 1-napthalenethiol (0.42 mL, 3.0 mmol) in chloroform (3.0 mL). The reaction mixture was stirred at 40 °C for 25 min then quenched with sat. aq. $NaHCO_3$ (10 mL). The layers were separated and the aqueous portion extracted with dichloromethane (3 × 5 mL). The organic extracts were combined, washed with aq. NaOH (1 M, 15 mL) then dried over Na_2SO_4, filtered and concentrated *in vacuo*. Purification by flash column chromatography (25% Et_2O/pentane) afforded oxetane **2l** (424 mg, 88%) as a colorless oil. R_f = 0.35 (25% Et_2O/pentane); IR (film)/cm^{-1} 2951, 2870, 1739, 1610, 1511, 1299, 1244, 1179, 1026, 983, 827, 799, 771, 729; ^{1}H NMR (400 MHz, $CDCl_3$) δ 8.42–8.39 (m, 1 H, $Ar_{(napthalene)}$-CH), 7.85–7.80 (m, 2 H, 2 × $Ar_{(napthalene)}$-CH), 7.51–7.37 (m, 3 H, 3 × $Ar_{(napthalene)}$-CH), 7.36–7.30 (m, 1 H, $Ar_{(napthalene)}$-CH), 6.93–6.87 (m, 2 H, 2 × $Ar_{(p\text{-OMe})}$-CH), 6.75–6.70 (m, 2 H, 2 × $Ar_{(p\text{-OMe})}$-CH), 5.11 (d, *J* = 6.4 Hz, 2 H, C*H*HOC*H*H), 5.04 (d, *J* = 6.4 Hz, 2 H, CH*H*OCH*H*), 3.76 (s, 3 H, OCH_3); ^{13}C NMR (101 MHz, $CDCl_3$) δ 158.6 (Ar-C_q-OMe), 135.6 ($Ar_{(napthalene)}$-C_q), 135.1 ($Ar_{(napthalene)}$-CH), 134.6 ($Ar_{(p\text{-OMe})}$-C_q-C_q), 134.0 ($Ar_{(napthalene)}$-C_q), 129.9 ($Ar_{(napthalene)}$-CH), 129.7 (Ar-C_q-S), 128.3 ($Ar_{(napthalene)}$-CH), 127.6 (2 × $Ar_{(p\text{-OMe})}$-CH), 126.6 ($Ar_{(napthalene)}$-CH), 126.1 ($Ar_{(napthalene)}$-CH), 125.9 ($Ar_{(napthalene)}$-CH), 125.3 ($Ar_{(napthalene)}$-CH), 113.6 (2 × $Ar_{(p\text{-OMe})}$-CH), 82.6 (CH_2OCH_2), 55.7 (C_q), 55.3 (OCH_3); HRMS (TOP-ASAP+) *m/z* calcd for $C_{20}H_{17}SO^+$ $[M+H-H_2O]^+$: 305.1000, Found: 305.1007.

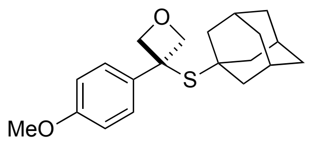

3-(((3s,5s,7s)-Adamantan-1-yl)thio)-3-(4-methoxyphenyl)oxetane (2m)

Lithium bis(trifluoromethanesulfonimide) (15.8 mg, 0.055 mmol) and tetrabutylammonium hexafluorophosphate (10.7 mg, 0.0275 mmol) were added to a solution of oxetanol **1** (90.1 mg, 0.5 mmol) and 1-adamantane thiol (168.3 mg, 2.0 mmol) in chloroform (1.0 mL). The reaction mixture was stirred at 40 °C for 25 min then quenched with sat. aq. $NaHCO_3$ (10 mL). The layers were separated and the aqueous portion extracted with dichloromethane (3 × 5 mL). The organic extracts were combined then dried over Na_2SO_4, filtered and concentrated *in vacuo*. Purification by flash column chromatography (10% Et_2O/pentane) afforded oxetane **2m** (104 mg, 63%) as a white solid; R_f = 0.40 (10% Et_2O/pentane); mp = 126–128 °C; IR (film)/cm^{-1} 2914, 2850, 1607, 1509, 1455, 1248, 1175, 1040, 1024, 986, 830, 815; ^{1}H NMR (400 MHz, $CDCl_3$) δ 7.35–7.29 (m, 2 H, 2 × Ar-CH), 6.92–6.86 (m, 2 H, 2 × Ar-CH), 5.14 (d, *J* = 6.1 Hz, 2 H, C*H*HOC*H*H), 4.96 (d, *J* = 6.1 Hz, 2 H, CH*H*OCH*H*), 3.84 (s, 3 H, OCH_3), 1.92–1.81 (m, 3 H, 3 × CH), 1.64–1.45 (m, 12 H, 6 × CH_2); ^{13}C NMR (101 MHz, $CDCl_3$) δ 158.4 (Ar-C_q-OMe), 136.7 (Ar-C_q-C_q), 128.0 (2 × Ar-CH), 113.6 (2 × Ar-CH), 84.6 (CH_2OCH_2), 55.3 (OCH_3), 51.7 ($C_{q(oxetane)}$), 48.7 ($C_{q(adamantane)}$), 43.7 (3 × CH_2), 36.1 (3 × CH_2), 29.6 (3 × CH); HRMS (TOF-ASAP+) *m/z* calcd for $C_{20}H_{26}SO_2^+$ $[M+H]^+$: 331.1732, Found: 331.1724.

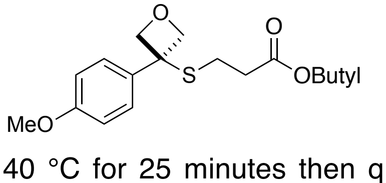

Butyl 3-((3-(4-methoxyphenyl)oxetan-3-yl)thio)propanoate (2n)

Lithium bis(trifluoromethanesulfonimide) (79.0 mg, 0.275 mmol) and tetrabutylammonium hexafluorophosphate (53.3 mg, 0.138 mmol) were added to a solution of oxetanol **1** (451 mg, 2.5 mmol) and butyl 3-mercaptopropionate (0.81 mL, 5.0 mmol) in chloroform (5 mL). The reaction mixture was stirred at 40 °C for 25 minutes then quenched with sat. aq. $NaHCO_3$ (10 mL). The layers were separated and the aqueous portion extracted with dichloromethane (3 × 5 mL). The organic extracts were combined then dried over Na_2SO_4, filtered and concentrated *in vacuo*. Purification by flash column chromatography (40% Et_2O/pentane) afforded oxetane **2n** (473 mg, 53%) as a colourless oil; R_f = 0.33 (40% Et_2O/pentane); IR (film)/cm^{-1} 2957, 2872, 1731 (C=O), 1512, 1244, 1178, 1027, 985, 830; ^{1}H NMR (400 MHz, $CDCl_3$) δ 7.16–7.12 (m, 2 H, 2 × Ar-CH), 6.93–6.88 (m, 2 H, 2 × Ar-CH), 5.19 (d, *J* = 6.4 Hz, 2 H, C*H*HOC*H*H), 4.90 (d, *J* = 6.4 Hz, 2 H, CH*H*OCH*H*), 4.08 (t, *J* = 6.7 Hz, 2 H, CO_2CH_2), 3.82 (s, 3 H, $ArOCH_3$), 2.66 (t, *J* = 7.4 Hz, 2 H, SCH_2), 2.41 (t, *J* = 7.4 Hz, 2 H, $CH_2C(O)$), 1.67–1.53 (m, 2 H, $CH_2CH_2CH_3$), 1.44–1.29 (m, 2 H, $CH_2CH_2CH_3$), 0.93 (t, J = 7.4 Hz, CH_2CH_3); ^{13}C NMR (101 MHz, $CDCl_3$) δ 171.7 (C=O), 158.6 (Ar-C_q-OMe), 134.3 (Ar-C_q-C_q), 127.5 (2 × Ar-CH), 114.0 (2 × Ar-CH), 83.2 (CH_2OCH_2), 64.6 (CO_2CH_2), 55.3 (Ar-OCH_3), 52.1 (C_q), 34.3

(*C*H_2C(O)), 30.6 (CH_2*C*H_2CH_3), 25.3 (SCH_2), 19.1 (*C*$H_2CH_2CH_3$), 13.7 (CH_2*C*H_3); HRMS (TOF-ASAP+) *m/z* calcd for $C_{17}H_{28}NSO_4^+$ $[M+NH_4]^+$: 342.1737, Found: 342.1733.

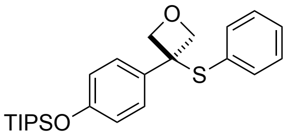

Triisopropyl(4-(3-(phenylthio)oxetan-3-yl)phenoxy)silane (8)

Lithium bis(trifluoromethanesulfonimide) (54.0 mg, 0.188 mmol) and tetrabutylammonium hexafluorophosphate (36.4 mg, 0.094 mmol) were added to a solution of oxetanol **4** (551 mg, 1.7 mmol) and thiophenol (0.35 mL, 3.4 mmol) in chloroform (3.4 mL). The reaction mixture was stirred at 40 °C for 25 min then quenched with sat. aq. $NaHCO_3$ (10 mL). The layers were separated and the aqueous portion extracted with dichloromethane (3 × 5 mL). The organic extracts were combined, washed with aq. NaOH (1 M, 15 mL) then dried over Na_2SO_4, filtered and concentrated *in vacuo*. Purification by flash column chromatography (10% Et_2O/pentane) afforded oxetane **8** (470 mg, 66%) as a colorless oil. R_f = 0.22 (10% Et_2O/pentane); IR (film)/cm^{-1} 2944, 2866, 1606, 1510, 1462, 1262, 988, 909, 882, 835, 748, 689; ^1H NMR (400 MHz, $CDCl_3$) δ 7.33–7.27 (m, 1 H, Ph-CH), 7.23–7.15 (m, 4 H, 4 × Ph-CH), 6.85–6.75 (m, 4 H, 4 × $Ar_{(p\text{-OTIPS})}$-CH), 5.14 (d, *J* = 6.4 Hz, 2 H, C*H*HOC*H*H), 4.97 (d, *J* = 6.4 Hz, 2 H, CH*H*OCH*H*), 1.31–1.20 (s, 3 H, C*H*($CH_3)_2$), 1.11 (d, *J* = 7.3 Hz, 18 H, C*H*($CH_3)_2$); ^{13}C NMR (101 MHz, $CDCl_3$) δ 155.1 (Ar-C_q-OTIPS), 135.5 (2 × Ph-CH), 135.1 (Ar-*C_q*-C_q), 132.0 (Ph-C_q-S), 128.8 (Ph-CH), 128.7 (2 × Ph-CH), 127.6 (2 × $Ar_{(p\text{-OTIPS})}$-CH), 119.6 (2 × $Ar_{(p\text{-OTIPS})}$-CH), 82.7 (CH_2OCH_2), 55.0 (C_q), 17.9 (CH(*C*$H_3)_2$), 12.7 (*C*H($CH_3)_2$); HRMS (TOP-ASAP+) *m/z* calcd for $C_{24}H_{35}O_2SSi^+$ $[M+H]^+$: 415.2127, Found: 415.2126.

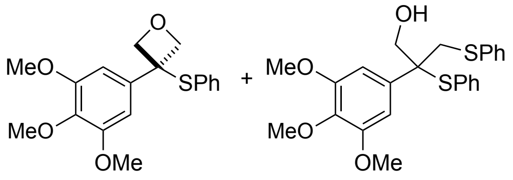

3-(Phenylthio)-3-(3,4,5-trimethoxyphenyl)oxetane (9) and 2,3-bis(phenylthio)-2-(3,4,5-trimethoxyphenyl)propan-1-ol (S2)

Lithium bis(trifluoromethanesulfonimide) (15.6 mg, 0.055 mmol) and tetrabutylammonium hexafluorophosphate (10.7 mg, 0.0275 mmol) were added to a solution of oxetanol **5** (120 mg, 0.5 mmol) and thiophenol (0.10 mL, 1.0 mmol) in chloroform (1.0 mL). The reaction mixture was stirred at 40 °C for 25 min then quenched with sat. aq. $NaHCO_3$ (10 mL). The layers were separated and the aqueous portion extracted with dichloromethane (3 × 5 mL). The organic extracts were combined, washed with aq. NaOH (1 M, 15 mL) then dried over Na_2SO_4, filtered and concentrated *in vacuo*. Purification by flash column chromatography (25% EtOAc/hexane) afforded oxetane **9** (39 mg, 23%) as a yellow oil followed by alcohol **S2** (54 mg, 25%).

Oxetane **9**: R_f = 0.16 (20% EtOAc/hexane); IR (film)/cm^{-1} 2940, 1588, 1509, 1456, 1414, 1349, 1243, 1127, 1006, 986, 752; ^1H NMR (400 MHz, $CDCl_3$) δ 7.35–7.30 (m, 1 H, Ph-CH), 7.27–7.19 (m, 4 H, 4 × Ph-CH), 6.12–6.07 (m, 2 H, 2 × Ar-CH), 5.14 (d, *J* = 6.4 Hz, 2 H, C*H*HOC*H*H), 4.97 (d, *J* = 6.4 Hz, 2 H, CH*H*OCH*H*), 3.83 (s, 3 H, OCH_3), 3.72 (s, 6 H, 2 × OCH_3); ^{13}C NMR (101 MHz, $CDCl_3$) δ 152.9 (2 × Ar-C_q-O), 138.2 (Ar-C_q-O), 137.0 (Ar-*C_q*-C_q), 135.6 (2 × Ph-CH), 131.8 (Ar-C_q-S), 129.0 (Ph-CH), 128.7 (2 × Ph-CH), 103.7 (2 × Ar-CH), 82.5 (CH_2OCH_2), 60.9 (OCH_3), 56.1 (2 × OCH_3), 55.5 (C_q); HRMS (TOP-ASAP+) *m/z* calcd for $C_{18}H_{21}SO_4^+$ $[M+H]^+$: 333.1161, Found: 333.1160.

Alcohol **S2**: R_f = 0.16 (20% EtOAc/hexane); IR (film)/cm^{-1} 3493 (br. OH), 2935, 1588, 1511, 1464, 1327, 1439, 1415, 1128, 749, 693; ^1H NMR (400 MHz, $CDCl_3$) δ 7.38–7.14 (m, 10 H, 10 × Ph-CH), 6.59–6.52 (m, 2 H, 2 × Ar-CH), 4.10 (d, *J* = 11.6 Hz, 1 H, C*H*HOH), 4.04 (d, *J* = 11.6 Hz, 1 H, CH*H*OH), 3.85 (s, 3 H, OCH_3), 3.74 (s, 6 H, 2 × OCH_3), 3.67 (d, *J* = 13.1 Hz, 1 H, C*H*HSPh), 3.57 (d, *J* = 13.1 Hz, 1 H, CH*H*SPh), 2.42 (s, 1 H, OH); ^{13}C NMR (101 MHz, $CDCl_3$) δ 152.7 (2 × Ar-C_q-O), 137.5 (Ar-C_q-C_q), 136.7 (2 × Ph-CH), 136.5 (Ar-*C_q*-O), 134.5 (Ar-C_q-S), 130.5 (2 × Ph-CH), 129.7 (Ar-C_q-S), 129.4 (Ph-CH), 128.8 (4 × Ph-CH), 126.6 (Ph-CH), 105.5 (2 × Ar-CH), 63.9 (CH_2OH), 61.5 (C_q), 60.8 (OCH_3), 56.1 (2 × OCH_3), 42.3 (CH_2SPh); HRMS (+ p NSI) *m/z* calcd for $C_{24}H_{26}S_2O_4Na^+$ $[M+Na]^+$: 465.1165, Found: 465.1159.

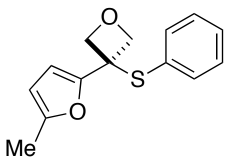

2-Methyl-5-(3-(phenylthio)oxetan-3-yl)furan (10)

Lithium bis(trifluoromethanesulfonimide) (47.4 mg, 0.165 mmol) and tetrabutylammonium hexafluorophosphate (32.0 mg, 0.0825 mmol) were added to a solution of oxetanol **6** (231 mg, 1.5 mmol) and thiophenol (0.31 mL, 3.0 mmol) in chloroform (3.0 mL). The reaction mixture was stirred at 40 °C for 25 min then quenched with sat. aq. $NaHCO_3$ (10 mL). The layers were separated and the aqueous portion extracted with dichloromethane (3 × 5 mL). The organic extracts were combined, washed with aq. NaOH (1 M, 15 mL) then dried over Na_2SO_4, filtered and concentrated *in vacuo*. Purification by flash column chromatography (5% Et_2O/pentane) afforded oxetane **10** (316 mg, 86%) as a colorless oil. R_f = 0.19 (5% Et_2O/pentane); IR (film)/cm^{-1} 2950, 2875, 1739, 1476, 1439, 1366, 1350, 1217, 1021, 985, 906, 783, 748, 691; ^1H NMR (400 MHz, $CDCl_3$) δ 7.30–7.21 (m, 5 H, 5 × Ph-CH), 6.01 (d, *J* = 3.1 Hz, 1 H, $Ar_{(furan)}$-CH), 5.90–5.87 (m, 1 H, $Ar_{(furan)}$-CH), 5.05 (d, *J* = 6.3 Hz, 2 H, C*H*HOC*H*H), 4.87 (d, *J* = 6.3 Hz, 2 H, CH*H*OCH*H*), 2.31 (d, *J* = 1.0 Hz, 3 H, CH_3); ^{13}C NMR (101 MHz, $CDCl_3$) δ 152.3 ($Ar_{(furan)}$-C_q-CH_3), 151.7 (Ar-C_q-C_q), 133.8 (2 × Ph-CH), 132.0 (Ar-C_q-S), 128.8 (2 × Ph-CH), 128.3 (Ph-CH), 109.0 ($Ar_{(furan)}$-CH), 106.4 ($Ar_{(furan)}$-CH), 81.2 (CH_2OCH_2), 49.8 (C_q), 13.6 (CH_3); HRMS (TOF-ASAP+) *m/z* calcd for $C_{14}H_{13}SO^+$ $[M+H-H_2O]^+$: 229.0687, Found: 229.0691.

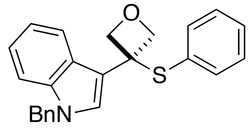

1-Benzyl-3-(3-(phenylthio)oxetan-3-yl)-1*H*-indole (11)

Lithium bis(trifluoromethanesulfonimide) (18.9 mg, 0.07 mmol) and tetrabutylammonium hexafluorophosphate (12.8 mg, 0.03 mmol) were added to a solution of oxetanol **7** (168 mg, 0.6 mmol) and thiophenol (0.12 mL, 1.2 mmol) in chloroform (1.2 mL). The reaction mixture was stirred at 40 °C for 25 min then quenched with sat. aq. $NaHCO_3$ (10 mL). The layers were separated and the aqueous portion extracted with dichloromethane (3 × 5 mL). The organic extracts were combined, washed with aq. NaOH (1 M, 15 mL) then dried over Na_2SO_4, filtered and concentrated *in vacuo*. Purification by flash column chromatography (20% Et_2O/pentane) afforded oxetane **11** (203 mg, 91%) as a colorless oil. R_f = 0.17 (20% Et_2O/pentane); IR (film)/cm^{-1} 2971, 2866, 1739, 1468, 1455, 1438, 1366, 1229, 1217, 983, 741, 695; ^1H NMR (400 MHz, $CDCl_3$) δ 7.75–7.71 (m, 1 H, $Ar_{(indole)}$-CH), 7.31–7.14 (m, 11 H, 11 × Ar-CH), 7.03–6.98 (m, 2 H, 2 × Ar-CH), 6.78 (s, 1 H, $Ar_{(indole)}$-CH), 5.25 (d, *J* = 6.3 Hz, 2 H, C*H*HOC*H*H), 5.23 (s, 2 H, CH_2), 5.13 (d, *J* = 6.3 Hz, 2 H, CH*H*OCH*H*); ^{13}C NMR (101 MHz, $CDCl_3$) δ 137.1 (Ar-C_q), 137.0 (Ar-C_q), 134.3 (2 × Ar-CH), 133.0 (Ar-C_q-S), 128.7 (2 × Ar-CH), 128.5 (2 × Ar-CH), 128.1 (Ar-CH) 127.6 (Ar-CH), 126.71 ($Ar_{(indole)}$-CH), 126.65 (2 × Ar-CH), 125.6 ($Ar_{(indole)}$-C_q), 122.3 (Ar-CH), 120.4 ($Ar_{(indole)}$-CH), 119.6 ($Ar_{(indole)}$-CH), 116.3 ($Ar_{(indole)}$-C_q), 110.0 ($Ar_{(indole)}$-CH), 83.3 (CH_2OCH_2), 50.9 (C_q), 49.9 (CH_2); HRMS (TOF-ASAP+) *m/z* calcd for $C_{24}H_{22}NSO^+$ $[M+H]^+$: 372.1422, Found: 372.1420.

Thiol alkylation with Secondary and Tertiary Alcohols, 12a–j

A varied set of π-activated secondary and tertiary alcohols were explored to demonstrate the wider applicability of these reaction conditions (scheme S1).

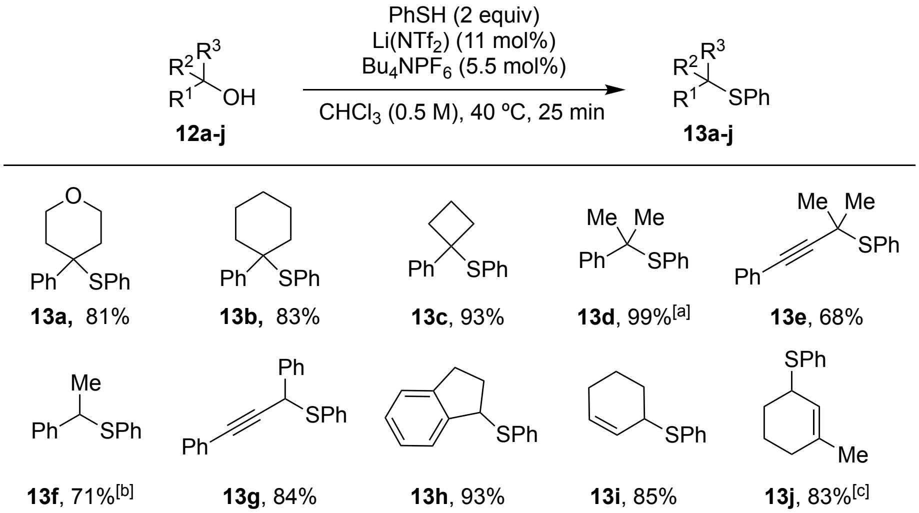

Scheme S1. Thiol alkylation with secondary and tertiary alcohols. [a] 93% yield when heptane used as solvent instead of $CHCl_3$. [b] 5 equivalents of benzenethiol. [c] 5:1 mixture, 3-methyl:1-methyl

Tertiary benzylic alcohols with tetrahydropyran (**12a**), cyclohexane (**12b**) and cyclobutane (**12c**) linkers all afforded the corresponding sulfides **13a–c** in excellent yields between 81% and 93%. Using 2-phenylpropan-2-ol gave product **13d** in 99% yield under the standard conditions, or 93% using heptane as the solvent. Tertiary propargylic alcohol **13e** afforded the corresponding sulfide **13e** in a 68% yield. Secondary alcohols with benzylic, indane, propargylic and vinylic structures all afforded the sulfides **13f–i** in high yields. For the synthesis of sulfide **13f** the equivalents of the nucleophile were increased from 2 to 5 to prevent the formation of a dimeric ether side product. Unsymmetrical tertiary allylic alcohol **12j** gave predominantly the secondary sulfide product **13j** due to attack at the least hindered position of the carbocationic intermediate.

Synthesis of Thiol Alkylation Precursors 12a–c, j

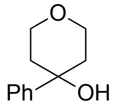

4-Phenyloxan-4-ol (12a)

n-BuLi (2.4 M in THF, 2.7 mL, 6.5 mmol) was added dropwise over 5 min to a solution of bromobenzene (0.69 mL, 6.5 mmol) in THF (20 mL) at –78 °C. The reaction mixture was stirred at –78 °C for a further 10 min. Oxan-4-one (0.46 mL, 5 mmol) was added dropwise as a solution in THF (5 mL) to the reaction mixture. Following a further 10 minutes at –78 °C the reaction mixture was warmed to rt and water (20 mL) was added. The layers were separated and the aqueous portion extracted with diethylether (3 × 30 mL). The organic extracts were combined, washed with brine (20 mL), dried over Na_2SO_4, filtered and concentrated *in vacuo*. Purification by flash column chromatography (50% EtOAc/hexane) afforded 4-phenyloxan-4-ol **12a** (780 mg, 87%) as a white solid. R_f = 0.35 (50% EtOAc/hexane); mp = 99–100 °C (Lit. mp = 100.0–100.5 °C)[8]; IR (film)/ cm^{-1} 3375 (br. OH), 3026, 2970, 2956, 2923, 2889, 1739, 1491, 1446, 1408, 1366, 1310, 1218, 1140, 1126, 1089, 1071, 1039, 1024, 1016, 1007, 965, 916, 834, 660, 759, 693; ^{1}H NMR (400 MHz, $CDCl_3$) δ 7.54–7.47 (m, 2 H, 2 × Ph-CH), 7.47–7.35 (m, 2 H, 2 × Ph-CH), 7.35–7.27 (m, 1 H, Ph-CH), 4.04–3.85 (m, 4 H, 2 × CH_2O), 2.21 (ddd, *J* = 13.6, 11.9, 5.4 Hz, 2 H, 2 × C*H*HC_q), 1.77–1.65 (m, 2 H, 2 × C*H*HC_q); ^{13}C NMR (101 MHz, $CDCl_3$) δ 148.0 (Ph-C_q), 128.5 (2 × Ph-CH), 127.2 (Ph-CH), 124.4 (2 × Ph-CH), 70.7 (C_q), 63.9 (2 × CH_2O), 38.8 (2 × C_q*C*H_2). The spectroscopic data for this compound is consistent with the literature.[8]

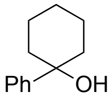

1-Phenylcyclohexan-1-ol (12b)

n-BuLi (2.3 M in THF, 2.8 mL, 6.5 mmol) was added dropwise over 5 min to a solution of bromobenzene (0.69 mL, 6.5 mmol) in THF (20 mL) at –78 °C. The reaction mixture was stirred at –78 °C for a further 10 min. Cyclohexanone (0.52 mL, 5 mmol) was added dropwise as a solution in THF (5 mL) to the reaction mixture. Following a further 10 minutes at –78 °C the reaction mixture was warmed to rt and water (20 mL) was added. The layers were separated and the aqueous portion extracted with diethylether (3 × 30 mL). The organic extracts were combined, washed with brine (20 mL), dried over Na_2SO_4, filtered and concentrated *in vacuo*. Purification by flash column chromatography (20% Et_2O/pentane) afforded 1-phenylcyclohexan-1-ol **12b** (735 mg, 83%) as a white solid. R_f = 0.26 (20% Et_2O/pentane); mp = 62–64 °C (Lit. mp = 61 °C)[9]; IR (film)/ cm^{-1} 3321 (br. OH), 3062, 3033, 2923, 2854, 1494, 1444, 1381, 1351, 1259, 1210, 1176, 1146, 1135, 1032, 1015, 974, 897, 849, 831, 754, 694; ^{1}H NMR (400 MHz, $CDCl_3$) δ 7.61–7.44 (m, 2 H, 2 × Ph-CH), 7.43–7.30 (m, 2 H, 2 × Ph-CH), 7.30–7.18 (m, 1 H, Ph-CH), 1.93–1.71 (m, 7 H, 2 × C_qCH_2 + C*H*HC*H*HC*H*H), 1.71–1.61 (m, 2 H, 2 × CH*H*CH_2CH*H*), 1.58 (s, 1 H, OH), 1.39–1.23 (m, 1 H, 1 × CH_2CH*H*CH_2); ^{13}C NMR (101 MHz, $CDCl_3$) δ 149.4 (Ph-C_q), 128.2 (2 × Ph-CH), 126.7 (Ph-CH), 124.6 (2 × Ph-CH), 73.1 (C_q), 38.8 (*C*H_2C_q*C*H_2), 25.5 (*C*H_2CH_2*C*H_2), 22.2 (CH_2*C*H_2CH_2). The spectroscopic data for this compound is consistent with the literature.[10]

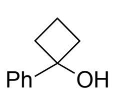

1-Phenylcyclobutan-1-ol (12c)

n-BuLi (2.4 M in THF, 2.7 mL, 6.5 mmol) was added dropwise over 5 min to a solution of bromobenzene (0.69 mL, 6.5 mmol) in THF (20 mL) at –78 °C. The reaction mixture was stirred at –78 °C for a further 10 min. Cyclobutanone (0.32 mL, 5.0 mmol) was added dropwise as a solution in THF (5 mL) to the reaction mixture. Following a further 10 minutes at –78 °C the reaction mixture was warmed to rt and water (20 mL) was added. The layers were separated and the aqueous portion extracted with diethylether (3 x 30 mL). The organic extracts were combined, washed with brine (20 mL), dried over $MgSO_4$, filtered and concentrated *in vacuo*. Purification by flash column chromatography (30% EtOAc/hexane) afforded 1-phenylcyclobutan-1-ol **12c** (574 mg, 78%) as a white solid. R_f = 0.3 (25% EtOAc/hexane); mp = 40 °C (lit. mp = 40–41 °C)[11]; IR (film)/ cm^{-1} 3303 (br. OH), 2989, 2940, 2869, 1495, 1446, 1423, 1246, 1185, 1132, 1110, 1074, 1026, 959, 913, 825, 760, 697, ^{1}H NMR (400 MHz, $CDCl_3$) δ 7.59–7.47 (m, 2 H, 2 × Ph-CH), 7.46–7.35 (m, 2 H, 2 × Ph-CH), 7.35–7.22 (m, 1 H, Ph-CH), 2.67–2.50 (m, 2 H, 2 × C_qC*H*H), 2.50–2.28 (m, 2 H, 2 × C_qCH*H*), 2.11–1.98 (m, 1 H, C_qCH_2C*H*H), 1.93 (br. s, 1 H, OH), 1.71 (dtt, *J* = 11.3, 8.8, 7.5 Hz, 1 H, C_qCH_2CH*H*); ^{13}C NMR (101 MHz, $CDCl_3$) δ 146.2 (Ph-C_q), 128.4 (2 × Ph-CH), 127.2 (Ph-CH), 125.0 (2 × Ph-CH), 36.8 (2 × C_q*C*H_2), 13.0 (C_qCH_2*C*H_2). The spectroscopic data for this compound is consistent with

the literature.[12] Quaternary carbon peak obscured by $CDCl_3$ peak in ^{13}C NMR but presence confirmed by HMBC.

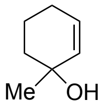

1-Methylcyclohex-2-en-1-ol (12j)

MeLi (1.6 M, 2.9 mL, 4.5 mmol) was added dropwise to 2-cyclohexen-1-one (0.29 mL, 3 mmol) in THF (5 mL) at 78 °C. The reaction mixture was stirred at –78 °C for 10 min then warmed to to 0 °C and stirred for a further 3 h. The reaction mixture was quenched with sat. aq. NH_4Cl (15 mL). The aqueous portion was extracted with EtOAc (3 × 50 mL). The organic portions were combined, dried over Na_2SO_4, filtered and concentrated *in vacuo*. Purification by flash column chromatography (40% Et_2O/pentane) afforded oxetane **12j** (337 mg, 54%) as a colorless oil; R_f = 0.23 (40% Et_2O/pentane); IR (film)/cm^{-1} 3366 (br. OH), 2933, 1368, 1180, 1122, 1101, 1017, 997, 963, 905, 728; ^1H NMR (400 MHz, $CDCl_3$) δ 5.75 (ddd, J = 10.0, 4.2, 3.1 Hz, 1 H, $C_qCH{=}C\mathit{H}$), 5.68–5.58 (m, 1 H, $C_qC\mathit{H}{=}CH$), 2.11–1.99 (m, 1 H, $CH{=}CHC\mathit{H}H$), 1.99–1.85 (m, 1 H, $CH{=}CHCH\mathit{H}$), 1.81–1.56 (m, 5 H, $C_qC\mathit{H_2}C\mathit{H_2}$ + OH), 1.29 (s, 3 H, CH_3); ^{13}C NMR (101 MHz, $CDCl_3$) δ 133.7 ($C_q\mathit{C}H{=}CH$), 129.0 ($C_qCH{=}\mathit{C}H$), 67.9 (C_q), 37.9 ($C_q\mathit{C}H_2$), 29.3 (CH_3), 25.0 ($CH{=}CH\mathit{C}H_2$), 19.5 ($CH_2\mathit{C}H_2CH_2$). The observed spectroscopic data for this compound was consistent with that previously reported.[13]

Synthesis of Tertiary Sulfides 13a–e

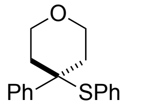

4-Phenyl-4-(phenylthio)tetrahydro-2*H*-pyran (13a)

Lithium bis(trifluoromethanesulfonimide) (47.4 mg, 0.165 mmol) and tetrabutylammonium hexafluorophosphate (32.0 mg, 0.0825 mmol) were added to a solution of 4-phenyltetrahydro-2*H*-pyran-4-ol **12a** (267 mg, 1.5 mmol) and thiophenol (0.31 mL, 3.0 mmol) in chloroform (3.0 mL). The reaction mixture was stirred at 40 °C for 25 min then quenched with sat. aq. $NaHCO_3$ (10 mL). The layers were separated and the aqueous portion extracted with dichloromethane (3 × 5 mL). The organic extracts were combined, washed with aq. NaOH (1 M, 15 mL) then dried over Na_2SO_4, filtered and concentrated *in vacuo*. Purification by flash column chromatography (5% EtOAc/hexane) afforded sulfide **13a** (328 mg, 81%) as a white solid. R_f = 0.18 (5% EtOAc/hexane); mp = 66–68 °C; IR (film)/cm^{-1} 2955, 2909, 2861, 1245, 1106, 1027, 1019, 749, 694, 655; ^{1}H NMR (400 MHz, $CDCl_3$) δ 7.33–7.10 (m, 8 H, 8 × Ph-CH), 7.02–6.93 (m, 2 H, 2 × Ph-CH), 4.12 (ddd, *J* = 11.5, 8.6, 2.9 Hz, 2 H, C*H*HOC*H*H), 3.76 (ddd, *J* = 11.5, 5.6, 3.7 Hz, 2 H, CH*H*OCH*H*), 2.27 (ddd, *J* = 12.4, 8.6, 3.7 Hz, 2 H, C*H*HC$_q$C*H*H), 2.22–2.11 (m, 2 H, CH*H*C$_q$CH*H*); ^{13}C NMR (101 MHz, $CDCl_3$) δ 144.5 (Ph-*C$_q$*-C$_q$), 137.1 (2 × Ph-CH), 130.9 (Ph-C$_q$-S), 128.8 (Ph-CH), 128.2 (2 × Ph-CH), 128.0 (2 × Ph-CH), 126.7 (Ph-CH), 126.6 (2 × Ph-CH), 64.2 (CH_2OCH_2), 52.6 (C$_q$), 35.9 (*C*H$_2$C$_q$*C*H$_2$); HRMS (TOF-ASAP+) *m/z* calcd for $C_{16}H_{21}NO_5S^+$ $[M+H]^+$: 340.1219, Found: 340.1210.

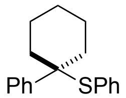

Phenyl(1-phenylcyclohexyl)sulfane (13b)

Lithium bis(trifluoromethanesulfonimide) (47.4 mg, 0.165 mmol) and tetrabutylammonium hexafluorophosphate (32.0 mg, 0.0825 mmol) were added to a solution of 1-phenylcyclohexan-1-ol **12b** (264 mg, 1.5 mmol) and thiophenol (0.31 mL, 3.0 mmol) in chloroform (3.0 mL). The reaction mixture was stirred at 40 °C for 25 min then quenched with sat. aq. $NaHCO_3$ (10 mL). The layers were separated and the aqueous portion extracted with dichloromethane (3 × 5 mL). The organic extracts were combined, washed with aq. NaOH (1 M, 15 mL) then dried over Na_2SO_4, filtered and concentrated *in vacuo*. Purification by flash column chromatography (2% EtOAc/hexane) afforded sulfide **13b** (403 mg, 83%) as a white solid. R_f = 0.28 (2% EtOAc/hexane); mp = 65–67 °C; IR (film)/cm^{-1} 3062, 2928, 2853, 1443, 745, 704, 691; ^{1}H NMR (400 MHz, $CDCl_3$) δ 7.30–7.18 (m, 6 H, 6 × Ph-CH), 7.18–7.11 (m, 2 H, 2 × Ph-CH), 7.04–6.99 (m, 2 H, 2 × Ph-CH), 2.30–2.19 (m, 2 H, C*H*HC$_q$C*H*H), 2.11–2.01 (m, 2 H, CH*H*C$_q$CH*H*), 1.92–1.80 (m, 2 H, CH$_2$C*H*HCH$_2$C*H*HCH$_2$) 1.59–1.39 (m, 4 H, CH$_2$CH*H*CH$_2$CH*H*CH$_2$ + CH$_2$CH$_2$C*H$_2$*CH$_2$CH$_2$); ^{13}C NMR (101 MHz, $CDCl_3$) δ 144.4 (Ph-*C$_q$*-C$_q$), 137.0 (2 × Ph-CH), 131.7 (Ph-C$_q$-S), 128.4 (Ph-CH), 128.0 (2 × Ph-CH), 127.8 (2 × Ph-CH), 127.0 (2 × Ph-CH), 126.2 (Ph-CH), 55.3 (C$_q$), 36.4 (*C*H$_2$CH$_2$CH$_2$CH$_2$*C*H$_2$), 25.9.0 (CH$_2$CH$_2$*C*H$_2$CH$_2$CH$_2$), 22.9 CH$_2$*C*H$_2$CH$_2$*C*H$_2$CH$_2$); HRMS (TOF-ASAP+) *m/z* calcd for $C_{16}H_{21}NO_5S^+$ $[M+H]^+$: 340.1219, Found: 340.1210.

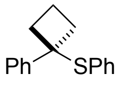

Phenyl(1-phenylcyclobutyl)sulfane (13c)

Lithium bis(trifluoromethanesulfonimide) (47.4 mg, 0.165 mmol) and tetrabutylammonium hexafluorophosphate (32.0 mg, 0.0825 mmol) were added to a solution of 1-phenylcyclobutan-1-ol **12c** (222 mg, 1.5 mmol) and thiophenol (0.31 mL, 3.0 mmol) in chloroform (3.0 mL). The reaction mixture was stirred at 40 °C for 25 min then quenched with sat. aq. $NaHCO_3$ (10 mL). The layers were separated and the aqueous portion extracted with dichloromethane (3 × 5 mL). The organic extracts were combined, washed with aq. NaOH (1 M, 15 mL) then dried over Na_2SO_4, filtered and concentrated *in vacuo*. Purification by flash column chromatography (2% EtOAc/hexane) afforded sulfide **13c** (337 mg, 93%) as a white solid. R_f = 0.21 (2% EtOAc/hexane); mp = 46–48 °C; IR (film)/cm^{-1} 2978, 2930, 1473, 1445, 1436, 774, 752, 692, 655; ^{1}H NMR (400 MHz, $CDCl_3$) δ 7.34–7.10 (m, 8 H, 8 × Ph-CH), 7.05–6.99 (m, 2 H, 2 × Ph-CH), 2.71–2.60 (m, 2 H, C*H*HC$_q$C*H*H), 2.57-2.35 (m, 3 H, CH*H*C$_q$CH*H* + CH$_2$C*H*HCH$_2$), 1.98–1.86 (m, 1 H, CH$_2$CH*H*CH$_2$); ^{13}C NMR (101 MHz, $CDCl_3$) δ 147.3 (Ph-*C$_q$*-C$_q$), 135.9 (2 × Ph-CH), 133.2 (Ph-C$_q$-S), 128.3 (Ph-CH), 128.2 (2 × Ph-CH), 127.7 (2 × Ph-CH), 126.1 (2 × Ph-CH), 126.0 (Ph-CH), 56.5 (C$_q$), 34.5 (*C*H$_2$C$_q$*C*H$_2$), 16.4 (CH$_2$*C*H$_2$CH$_2$); HRMS (EI) *m/z* calcd for $C_{16}H_{16}S^+$ $[M]^+$: 240.0967, Found 240.0965.

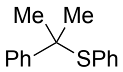

Phenyl(2-phenylpropan-2-yl)sulfane (13d)

Lithium bis(trifluoromethanesulfonimide) (47.4 mg, 0.165 mmol) and tetrabutylammonium hexafluorophosphate (32.0 mg, 0.0825 mmol) were added to a solution of 2-Phenylpropan-2-ol (210 μL, 1.5 mmol) and thiophenol (0.31 mL, 3.0 mmol) in chloroform (3.0 mL). The reaction mixture was stirred at 40 °C for 25 min then quenched with sat. aq. $NaHCO_3$ (10 mL). The layers were separated and the aqueous portion extracted with dichloromethane (3 × 5 mL). The organic extracts were combined, washed with aq. NaOH (1 M, 15 mL) then dried over Na_2SO_4, filtered and concentrated *in vacuo*. Purification by flash column chromatography (5% EtOAc/hexane) afforded sulfide **13d** (340 mg, 99%) as a colorless oil. R_f = 0.34 (5% EtOAc/hexane); IR (film)/cm^{-1} 2955, 2909, 2861, 1245, 1106, 1027, 1019, 749, 694, 655; ^{1}H NMR (400 MHz, $CDCl_3$) δ 7.47–7.42 (m, 2 H, 2 × Ph-CH), 7.34–7.15 (m, 8 H, 8 × Ph-CH), 1.72 (s, 6 H, 2 × CH_3); ^{13}C NMR (101 MHz, $CDCl_3$) δ 146.3 (Ph-C_q-C_q), 136.5 (2 × Ph-CH), 132.8 (Ph-C_q-S), 128.5 (Ph-CH), 128.2 (2 × Ph-CH), 127.9 (2 × Ph-CH), 126.53 (2 × Ph-CH), 126.50 (Ph-CH), 51.0 (C_q), 29.7 (2 × CH_3). The observed spectroscopic data for this compound was consistent with that previously reported.[14]

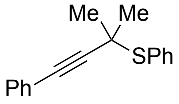

(2-Methyl-4-phenylbut-3-yn-2-yl)(phenyl)sulfane (13e)

Lithium bis(trifluoromethanesulfonimide) (47.4 mg, 0.165 mmol) and tetrabutylammonium hexafluorophosphate (32.0 mg, 0.0825 mmol) were added to a solution of 2-methyl-4-phenylbut-3-yn-2-ol (240 mg, 1.5 mmol) and thiophenol (0.31 mL, 3.0 mmol) in chloroform (3.0 mL). The reaction mixture was stirred at 40 °C for 25 min then quenched with sat. aq. $NaHCO_3$ (10 mL). The layers were separated and the aqueous portion extracted with dichloromethane (3 × 5 mL). The organic extracts were combined, washed with aq. NaOH (1 M, 15 mL) then dried over Na_2SO_4, filtered and concentrated *in vacuo*. Purification by flash column chromatography (10% dichloromethane/pentane) afforded sulfide **13e** (259 mg, 68%) as a colorless oil. R_f = 0.35 (10% dichloromethane/pentane); IR (film)/cm^{-1} 2971, 1739, 1490, 1474, 1439, 1360, 1121, 750, 705, 690; ^{1}H NMR (400 MHz, $CDCl_3$) δ 7.73–7.69 (m, 2 H, 2 × Ph-CH), 7.44–7.28 (m, 8 H, 8 × Ph-CH), 1.65 (6 H, s, 2 × CH_3); ^{13}C NMR (101 MHz, $CDCl_3$) δ 136.9 (2 × Ph-CH), 132.5 (Ph-C_q-C_q), 131.5 (2 × Ph-CH), 129.1 (Ph-CH), 128.4 (2 × Ph-CH), 128.2 (2 × Ph-CH), 127.9 (Ph-CH), 123.2 (Ph-C_q-S), 93.9 ($C_{(alkyne)}C(CH_3)_2$), 83.3 (Ph$C_{(alkyne)}$), 42.5 (C_q), 30.5 (2 × CH_3). HRMS (TOF-ASAP+) *m/z* calcd for $C_{17}H_{17}S^+$ $[M+H]^+$: 253.1051, Found: 253.1053.

Synthesis of Secondary Sulfides 13f–j

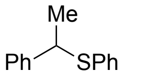

Phenyl(1-phenylethyl)sulfane (13f)

Lithium bis(trifluoromethanesulfonimide) (47.4 mg, 0.165 mmol) and tetrabutylammonium hexafluorophosphate (32.0 mg, 0.0825 mmol) were added to a solution of 1-phenylethan-1-ol (181 μL, 1.5 mmol) and thiophenol (0.31 mL, 3.0 mmol) in chloroform (3.0 mL). The reaction mixture was stirred at 40 °C for 25 min then quenched with sat. aq. $NaHCO_3$ (10 mL). The layers were separated and the aqueous portion extracted with dichloromethane (3 × 5 mL). The organic extracts were combined, washed with aq. NaOH (1 M, 15 mL) then dried over Na_2SO_4, filtered and concentrated *in vacuo*. Purification by flash column chromatography (10% dichloromethane/pentane) afforded sulfide **13f** (227 mg, 71%) as a colorless oil. R_f = 0.25 (10% dichloromethane/pentane); IR (film)/cm^{-1} 3059, 3028, 2924, 1739, 1492, 1480, 1451, 1438, 1372, 1217, 1025, 763, 739, 691; ^{1}H NMR (400 MHz, $CDCl_3$) δ 7.37–7.18 (m, 10 H, 10 × Ph-CH), 4.37 (q, *J* = 7.0 Hz, 1 H, CH), 1.66 (s, 3 H, CH_3); ^{13}C NMR (101 MHz, $CDCl_3$) δ 143.2 (Ph-C_q-C_q), 135.1 (Ph-C_q-S), 132.5 (2 × Ph-CH), 128.6 (2 × Ph-CH), 128.4 (2 × Ph-CH), 127.2 (2 × Ph-CH), 127.1 (2 × Ph-CH), 48.0 (CH), 22.3 (CH_3). The observed spectroscopic data for this compound was consistent with that previously reported.[15]

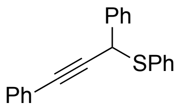

(1,3-Diphenylprop-2-yn-1-yl)(phenyl)sulfane (13g)

Lithium bis(trifluoromethanesulfonimide) (47.4 mg, 0.165 mmol) and tetrabutylammonium hexafluorophosphate (32.0 mg, 0.0825 mmol) were added to a solution of 1,3-diphenylprop-2-yn-1-ol (90% pure, 316 μL, 1.5 mmol) and thiophenol (0.31 mL, 3.0 mmol) in chloroform (3.0 mL). The reaction mixture was stirred at 40 °C for 25 min then quenched with sat. aq. $NaHCO_3$ (10 mL). The layers were separated and the aqueous portion extracted with dichloromethane (3 × 5 mL). The organic extracts were combined, washed with aq. NaOH (1 M, 15 mL) then dried over Na_2SO_4, filtered and concentrated *in vacuo*. Purification by flash column chromatography (2% Et_2O/pentane) afforded sulfide **13g** (403 mg, 84%) as a yellow oil. R_f = 0.19 (2% Et_2O/pentane); IR (film)/cm^{-1} 3057, 2030, 1739, 1490, 1439, 1025, 753, 689; ^{1}H NMR (400 MHz, $CDCl_3$) δ 7.51–7.45 (m, 4 H, 4 × Ph-CH), 7.43–7.38 (m, 2 H, 2 × Ph-CH), 7.37–7.27 (m, 9 H, 9 × Ph-CH), 5.24 (1 H, s, CH); ^{13}C NMR (101 MHz, $CDCl_3$) δ 138.0 (Ph-C_q-S), 134.5 (2 × Ph-CH), 133.4 (Ph-C_q), 131.6 (2 × Ph-CH), 128.7 (2 × Ph-CH), 128.5 (2 × Ph-CH), 128.4 (Ph-CH), 128.3 (Ph-CH), 128.2 (2 × Ph-CH), 127.8 (2 × Ph-CH), 122.9 (Ph-CH), 87.3 ($C_{(alkyne)}$CH), 86.9 (Ph$C_{(alkyne)}$), 44.3 (CH). The observed spectroscopic data for this compound was consistent with that previously reported.[16]

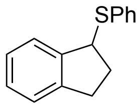

(2,3-Dihydro-1*H*-inden-1-yl)(phenyl)sulfane (13h)

Lithium bis(trifluoromethanesulfonimide) (47.4 mg, 0.165 mmol) and tetrabutylammonium hexafluorophosphate (32.0 mg, 0.0825 mmol) were added to a solution of 2,3-dihydro-1*H*-inden-1-ol (201.3 mg, 1.5 mmol) and thiophenol (0.31 mL, 3.0 mmol) in chloroform (3.0 mL). The reaction mixture was stirred at 40 °C for 25 min then quenched with sat. aq. $NaHCO_3$ (10 mL). The layers were separated and the aqueous portion extracted with dichloromethane (3 × 5 mL). The organic extracts were combined, washed with aq. NaOH (1 M, 15 mL) then dried over Na_2SO_4, filtered and concentrated *in vacuo*. Purification by flash column chromatography (2% Et_2O/pentane) afforded sulfide **13h** (340 mg, 93%) as a white solid. R_f = 0.25 (2% Et_2O/pentane); mp = 29–31 °C; IR (film)/cm^{-1} 3023, 2935, 2849, 1737, 1581, 1568, 1476, 1458, 1436, 1021, 756, 735, 699, 688; ^{1}H NMR (400 MHz, $CDCl_3$) δ 7.46–7.37 (m, 2 H, 2 × Ar-CH), 7.37–7.14 (m, 7 H, 7 × Ar-CH), 4.80 (dd, *J* = 7.3, 4.0 Hz, 1 H, CHSPh), 3.06 (dt, *J* = 15.7, 7.8 Hz, 1 H, Ar-C*H*H), 2.89 (ddd, *J* = 15.7, 8.4, 4.3 Hz, 1 H, Ar-CH*H*), 2.57 (ddt, *J* = 13.4, 8.4, 7.3 Hz, 1 H, CH*H*CH), 2.24 (ddt, *J* = 13.4, 8.4, 4.0 Hz, 1 H, CH*H*CH); ^{13}C NMR (101 MHz, $CDCl_3$) δ 143.8 (Ar-C_q-CH_2), 142.8 (Ar-C_q-CH), 136.0 (Ph-C_q-S), 131.3 (2 × Ph-CH), 128.8 (2 × Ph-CH), 127.7 (Ph-CH), 126.7 (Ar-CH), 126.5 (Ar-CH), 124.9 (Ar-CH), 124.7 (Ar-CH), 51.9 (CH), 33.6 (CH_2CH), 30.8 (Ar-CH_2). The observed spectroscopic data for this compound was consistent with that previously reported.[17]

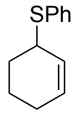

Cyclohex-2-en-1-yl(phenyl)sulfane (13i)

Lithium bis(trifluoromethanesulfonimide) (47.4 mg, 0.165 mmol) and tetrabutylammonium hexafluorophosphate (32.0 mg, 0.0825 mmol) were added to a solution of cyclohex-2-en-1-ol (147 μL, 1.5 mmol) and thiophenol (0.31 mL, 3.0 mmol) in chloroform (3.0 mL). The reaction mixture was stirred at 40 °C for 25 min then quenched with sat. aq. $NaHCO_3$ (10 mL). The layers were separated and the aqueous portion extracted with dichloromethane (3 × 5 mL). The organic extracts were combined, washed with aq. NaOH (1 M, 15 mL) then dried over Na_2SO_4, filtered and concentrated *in vacuo*. Purification by flash column chromatography (1% Et_2O/pentane) afforded sulfide **13i** (285 mg, 85%) as a colorless oil. R_f = 0.39 (1% Et_2O/pentane); IR (film)/cm^{-1} 3026, 2935, 1739, 1479, 1438, 1204, 1089, 870, 723, 690, 736; ^1H NMR (400 MHz, $CDCl_3$) δ 7.47–7.42 (m, 2 H, 2 × Ph-CH), 7.35–7.28 (m, 2 H, 2 × Ph-CH), 7.27–7.21 (m, 1 H, Ph-CH), 5.91–5.84 (m, 1 H, CHCH=C*H*), 5.84–5.75 (m, 1 H, CHC*H*=CH), 3.96–3.82 (m, 1 H, C*H*CH=CH), 2.16–2.02 (m, 2 H, CH=CHCH_2), 2.02–1.86 (m, 2 H, CHC*H*HCH_2 + CHCH_2C*H*H), 1.86–1.74 (m, 1 H, CHCH*H*CH_2), 1.71–1.55 (m, 1 H, CHCH_2CH*H*); ^{13}C NMR (101 MHz, $CDCl_3$) δ 135.8 (Ph-C_q-S), 131.2 (2 × Ph-CH), 130.4 (*C*H=CH), 128.8 (2 × Ph-CH), 126.8 (CH=*C*H), 126.5 (Ph-CH), 43.8 (*C*HCH=CH), 28.7 (CH=CH*C*H_2), 24.9 (CH*C*H_2CH_2), 19.4 (CHCH_2*C*H_2). The observed spectroscopic data for this compound was consistent with that previously reported.[18]

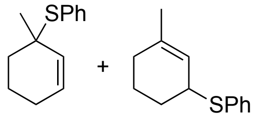

(1-Methylcyclohex-2-en-1-yl)(phenyl)sulfane (S3) and (3-methylcyclohex-2-en-1-yl)(phenyl)sulfane (13j)

Lithium bis(trifluoromethanesulfonimide) (32 mg, 0.11 mmol) and tetrabutylammonium hexafluorophosphate (21 mg, 0.055 mmol) were added to a solution of alcohol **12j** (112 mg, 1.0 mmol) and benzenethiol (0.21 mL, 2.0 mmol) in chloroform (2.0 mL). The reaction mixture was stirred at 40 °C for 25 min then quenched with sat. aq. $NaHCO_3$ (10 mL). The layers were separated and the aqueous portion extracted with dichloromethane (3 × 5 mL). The organic extracts were combined, dried over Na_2SO_4, filtered and concentrated *in vacuo*. Purification by flash column chromatography (2% EtOAc/hexane) afforded a mixture of sulfide **S3** (minor) and sulfide **13j** (major) (169 mg, 83%) in a 1:5 ratio as a colorless oil. R_f = 0.28 (2% EtOAc/hexane); IR (film)/cm^{-1} 2930, 1739, 1479, 1438, 873, 749, 736, 690; ^1H NMR (400 MHz, $CDCl_3$) δ 7.43–7.39 (m, 2 H, 2 × Ph-CH), 7.32–7.26 (m, 2 H, 2 × Ph-CH), 7.25–7.19 (m, 1 H, Ph-CH), 5.52 (dq, *J* = 3.3, 1.6 Hz, 1 H, C_q=CH), 3.90 (dtt, *J* = 5.4, 3.3, 1.6 Hz, 1 H, CHSPh), 2.00–1.84 (m, 4 H, C_qCH_2 + CH_2C*H*HCH_2 + C*H*HCH), 1.78–1.73 (m, 1 H, CH_2CH*H*CH_2), 1.70 (s, 3 H, CH_3), 1.65–1.59 (m, 1 H, CH*H*CH); ^{13}C NMR (101 MHz, $CDCl_3$) δ 138.3 (CH=C_qCH_3), 136.3 (Ph-C_q-S), 131.0 (2 × Ph-CH), 128.8 (2 × Ph-CH), 126.4 (Ph-CH), 121.0 (*C*H=CCH_3), 44.5 (CHSPh), 30.0 (C_q(CH_3)*C*H_2), 28.4 (CH_2*C*H_2CH_2), 23.9 (CH_3), 19.6 (*C*H_2CH); HRMS (CI^+) *m/z* calcd for $C_{13}H_{15}S^+$ $[M-2H+H]^+$: 203.0889, Found: 203.0893.

Derivatization of Oxetane Sulfide 8

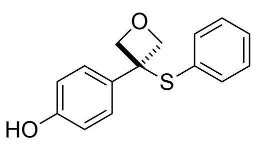

4-(3-(Phenylthio)oxetan-3-yl)phenol (S4)

TBAF (1 M in THF, 1.5 mL, 1.5 mmol) was added dropwise to a solution of oxetane sulfide **8** (415 mg, 1.0 mmol) in THF (3.3 mL) at 0 °C. The reaction mixture was stirred at 25 °C for 3 h then quenched with sat. aq. NH_4Cl (15 mL). EtOAc (3 × 15 mL) was added and the layers separated. The organic extracts were combined, dried over Na_2SO_4, filtered and concentrated *in vacuo*. Purification by flash column chromatography (50% Et_2O/pentane) afforded oxetane **S4** (239 mg, 93%) as a white solid. R_f = 0.27 (50% Et_2O/pentane); mp = 169–171 °C; IR (film)/cm^{-1} 3269 (br. OH), 2940, 2879, 1515, 1438, 1209, 1170, 1108, 965, 825, 748, 691; ^{1}H NMR (400 MHz, $CDCl_3$) δ 7.34–7.29 (m, 1 H, Ph-CH), 7.27–7.18 (m, 4 H, 4 × Ph-CH), 6.93–6.87 (m, 2 H, 2 × $Ar_{(p\text{-OH})}$-CH), 6.77–6.71 (m, 2 H, 2 × $Ar_{(p\text{-OH})}$-CH), 5.13 (d, *J* = 6.4 Hz, 2 H, C*H*HOC*H*H), 4.98 (d, *J* = 6.4 Hz, 2 H, CH*H*OCH*H*), 4.93 (s, 1 H, OH); ^{13}C NMR (101 MHz, $CDCl_3$) δ 154.6 (Ar-C_q-OH), 135.3 (2 × Ph-CH), 134.9 (Ar-C_q-C_q), 132.0 (Ph-C_q-S), 128.77 (Ph-CH), 128.75 (2 × Ph-CH), 127.9 (2 × $Ar_{(p\text{-OH})}$-CH), 115.1 (2 × $Ar_{(p\text{-OH})}$-CH), 82.7 (CH_2OCH_2), 54.8 (C_q); HRMS (TOP-ASAP+) *m/z* calcd for $C_{15}H_{15}SO_2^+$ [M+H]$^+$: 259.0793, Found: 259.0793.

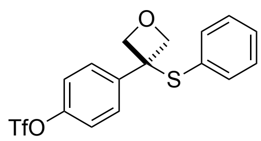

4-(3-(Phenylthio)oxetan-3-yl)phenyl trifluoromethanesulfonate (14)

Pyridine (0.13 mL, 1.6 mmol) then triflic anhydride (0.16 mL, 0.96 mmol) were added to a solution of oxetane **S4** (207 mg, 0.8 mmol) in dichloromethane (1.6 mL). The reaction mixture was stirred at 25 °C for 3 h then water (15 mL) was added followed by dichloromethane (10 mL). The layers were separated and the aqueous portion was extracted with dichloromethane (2 × 10 mL). The organic extracts were combined, dried over Na_2SO_4, filtered and concentrated *in vacuo*. Purification by flash column chromatography (20% Et_2O/pentane) afforded oxetane **14** (287 mg, 92%) as a white solid. R_f = 0.42 (30% Et_2O/pentane); mp = 58–60 °C; IR (film)/cm^{-1} 2957, 2884, 1501, 1419, 1249, 1229, 1214, 1202, 1140, 1130, 987, 884, 840, 829, 749, 693; ^{1}H NMR (400 MHz, ($CDCl_3$) δ 7.36–7.30 (m, 1 H, Ph-CH), 7.26–7.20 (m, 2 H, 2 × Ph-CH), 7.19–7.11 (m, 4 H, 2 × Ph-CH + 2 × $Ar_{(p\text{-OTf})}$-CH), 7.03–6.99 (m, 2 H, 2 × $Ar_{(p\text{-OTf})}$-CH), 5.14 (d, *J* = 6.6 Hz, 2 H, C*H*HOC*H*H), 5.02 (d, *J* = 6.6 Hz, 2 H, CH*H*OCH*H*); ^{13}C NMR (101 MHz, $CDCl_3$) δ 148.2 (Ar-C_q-C_q), 143.3 (Ar-C_q-OTf), 135.5 (2 × Ph-CH), 131.0 (Ph-C_q-S), 129.3 (Ph-CH), 129.0 (2 × Ar-CH), 128.3 (2 × Ar-CH), 121.1 (2 × Ar-CH), 118.7 (q, J_{CF} = 320.0 Hz, CF_3), 82.2 (CH_2OCH_2), 54.6 (C_q); ^{19}F NMR (377 MHz, $CDCl_3$) δ –72.7; HRMS (TOP-ASAP+) *m/z* calcd for $C_{16}H_{14}F_3O_4S_2^+$ [M+H]$^+$: 391.0286, Found: 391.0283.

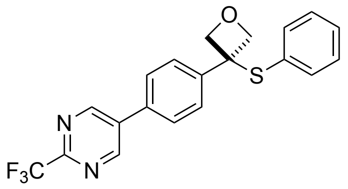

5-(4-(3-(Phenylthio)oxetan-3-yl)phenyl)-2-(trifluoromethyl)pyrimidine (15)

Oxetane **14** (98 mg, 0.25 mmol), 5-(4,4,5,5-tetramethyl-1,3,2-dioxaborolan-2-yl)-2-(trifluoromethyl)pyrimidine (91 mg, 0.33 mmol), $Pd(OAc)_2$ (2.8 mg, 0.0125 mmol), SPhos (10.2 mg, 0.025 mmol) and K_3PO_4 (106 mg, 0.5 mmol) were added to a reaction vial. The reaction vial was evacuated and then refilled with nitrogen (× 3). Degassed dioxane/water (4:1, 2.5 mL) was added *via* syringe. The reaction mixture was stirred at 65 °C for 18 h then cooled to rt. Diethylether (15 mL) was added and the crude mixture was filtered through celite® and then concentrated *in vacuo*. Purification by flash column chromatography (30% Et_2O/pentane) afforded oxetane **15** (92 mg, 95%) as a white solid. R_f = 0.15 (30% Et_2O/pentane); mp = 110–112 °C; IR (film)/cm^{-1} 3053, 2950, 2865, 1357, 1185, 1138, 1118, 986, 829, 748, 692; ^{1}H NMR (400 MHz, ($CDCl_3$) δ 9.10 (s, 2 H, 2 × $Ar_{(pyridyl)}$-CH), 7.59–7.54 (m, 2 H, 2 × Ar-CH), 7.38–7.31 (m, 1 H, Ph-CH), 7.30–7.19 (m, 6 H, 6 × Ar-CH), 5.19 (d, *J* = 6.5 Hz, 2 H, C*H*HOC*H*H), 5.07 (d, *J* = 6.5 Hz, 2 H, CH*H*OCH*H*); ^{13}C NMR (101 MHz, $CDCl_3$) δ 155.6 (2 × $Ar_{(pyridyl)}$-CH), 155.4 (q, J_{CF} = 36.8 Hz, $Ar_{(pyridyl)}$-C_q-CF_3), 144.7 (Ar-C_q-C_q), 135.4 (Ar-C_q-C_q), 135.2 (2 × Ph-CH), 131.0 (Ph-C_q-S), 131.4 (Ar-C_q-C_q), 129.1 (Ph-CH), 128.9 (2 × Ph-CH), 127.8 (2 × Ar-CH), 127.1 (2 × Ar-CH), 119.6 (q, J_{CF} = 275.2 Hz, CF_3), 82.2 (CH_2OCH_2), 54.7 (C_q); ^{19}F NMR (377 MHz, $CDCl_3$) δ –70.1; FTMS (+ p NSI) m/z calcd for $C_{20}H_{16}F_3OSN_2^+$ [M+H]$^+$: 389.0930, Found: 389.0931.

Synthesis of Oxetane-3-sulfoxides 16c,d,f

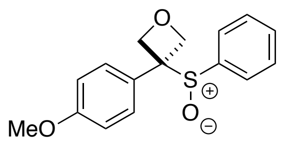

3-(Benzenesulfinyl)-3-(4-methoxyphenyl)oxetane (16c)

m-CPBA (45.3 mg, 0.26 mmol) was added in 3 portions over 30 minutes to a solution of 3-(4-methoxyphenyl)-3-(phenylsulfanyl)oxetane **2c** (68.1 mg, 0.25 mmol) in dichloromethane (2.5 mL). The reaction mixture was stirred at 0 °C for 2.5 h then 3 M $KOH_{(aq)}$ (5 mL) added and the phases separated. Dichloromethane (5 × 5 mL) was added and the layers separated. The organic extracts were combined, dried over Na_2SO_4, filtered and concentrated *in vacuo*. Purification by flash column chromatography (70% EtOAc/pentane) afforded sulfoxide **16c** (67 mg, 93%) as a white solid; R_f = 0.21 (70% EtOAc/pentane); mp = 85–87 °C; IR (film)/cm^{-1}; ^{1}H NMR (400 MHz, $CDCl_3$) δ 7.46–7.40 (m, 1 H, 1 × Ph-CH), 7.33–7.27 (m, 2 H, 2 × Ph-CH), 7.05–6.99 (m, 2 H, 2 × Ph-CH), 6.83–6.78 (m, 2 H, 2 × $Ar_{(p\text{-OMe})}$-CH), 6.61–6.55 (m, 2 H, 2 × $Ar_{(p\text{-OMe})}$-CH), 5.37 (d, *J* = 7.1 Hz, 1 H, C*H*HOCH$_2$), 5.33 (d, *J* = 7.1 Hz, 1 H, CH*H*OCH$_2$), 5.00 (d, *J* = 7.1 Hz, 1 H, CH$_2$OC*H*H), 4.87 (d, *J* = 7.1 Hz, 1 H, CH$_2$OCH*H*), 3.82 (s, 3 H, OCH_3); ^{13}C NMR (101 MHz, $CDCl_3$) δ 159.7 (Ar-C_q-OMe), 138.7 (Ph-C_q-SO), 131.5 (Ph-CH), 129.0 (2 × $Ar_{(p\text{-OMe})}$-CH), 128.2 (2 × Ph-CH), 126.2 (Ar-C_q-C_q), 125.4 (2 × Ph-CH), 113.7 (2 × $Ar_{(p\text{-OMe})}$-CH), 73.5 (CH_2OCH_2), 67.3 (C_q), 55.3 (OCH_3); HRMS (TOF-ASAP+) *m/z* calcd for $C_{16}H_{17}SO_3^+$ $[M+H]^+$: 289.0898, Found: 289.0895.

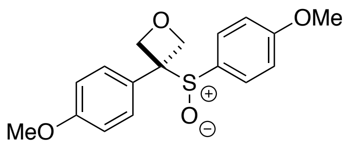

3-(4-Methoxybenzenesulfinyl)-3-(4-methoxyphenyl)oxetane (16d)

m-CPBA (90.6 mg, 0.525 mmol) was added in 3 portions over 30 minutes to a solution of 3-(4-methoxyphenyl)-3-[(4-methoxyphenyl)sulfanyl]oxetane **2d** (151.2 mg, 0.5 mmol) in dichloromethane (5.0 mL). The reaction mixture was stirred at 0 °C for 2.5 h then 3 M $KOH_{(aq)}$ (10 mL) added and the phases separated. Dichloromethane (5 × 10 mL) was added and the layers separated. The organic extracts were combined, dried over Na_2SO_4, filtered and concentrated *in vacuo*. Purification by flash column chromatography (70% EtOAc/pentane) afforded sulfoxide **16d** (131 mg, 82%) as a white solid; R_f = 0.07 (70% Et_2O/pentane); mp = 130–132 °C; IR (film)/cm^{-1}; ^{1}H NMR (400 MHz, $CDCl_3$) δ 6.98–6.92 (m, 2 H, 2 × Ar-CH), 6.85–6.78 (m, 4 H, 4 × Ar-CH), 6.62–6.57 (m, 2 H, 2 × Ar-CH), 5.36 (d, *J* = 7.0 Hz, 1 H, C*H*HOCH$_2$), 5.28 (d, *J* = 7.0 Hz, 1 H, CH*H*OCH$_2$), 4.99 (d, *J* = 7.0 Hz, 1 H, CH$_2$OC*H*H), 4.88 (d, *J* = 7.0 Hz, 1 H, CH$_2$OCH*H*), 3.83 (s, 3 H, OCH_3), 3.81 (s, 3 H, OCH_3); ^{13}C NMR (101 MHz, $CDCl_3$) δ 162.3 (Ar-C_q-OMe), 159.6 (Ar-C_q-OMe), 129.4 (Ar-C_q-SO), 129.0 (2 × Ar-CH), 127.2 (2 × Ar-CH), 126.3 (Ar-C_q-C_q), 113.8 (2 × Ar-CH), 113.6 (2 × Ar-CH), 73.7 (CH_2OCH_2), 67.2 (C_q), 55.44 (OCH_3), 55.35 (OCH_3); HRMS (TOF-ASAP+) *m/z* calcd for $C_{17}H_{19}SO_4^+$ $[M+H]^+$: 319.1004, Found: 319.0998.

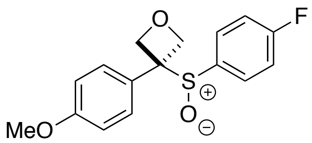

3-(4-Fluorobenzenesulfinyl)-3-(4-methoxyphenyl)oxetane (16f)

m-CPBA (90.6 mg, 0.525 mmol) was added in 3 portions over 30 minutes to a solution of 3-[(4-fluorophenyl)sulfanyl]-3-(4-methoxyphenyl)oxetane **2f** (145.2 mg, 0.5 mmol) in dichloromethane (5.0 mL). The reaction mixture was stirred at 0 °C for 2.5 h then 3 M $KOH_{(aq)}$ (10 mL) added and the phases separated. Dichloromethane (5 × 10 mL) was added and the layers separated. The organic extracts were combined, dried over Na_2SO_4, filtered and concentrated *in vacuo*. Purification by flash column chromatography (70% EtOAc/pentane) afforded sulfoxide **16f** (131 mg, 82%) as a white solid; R_f = 0.12 (70% Et_2O/pentane); mp = 95–97 °C; IR (film)/cm^{-1} 2938, 2875, 1609, 1586, 1513, 1488, 1253, 1223, 1179, 1160, 1042 (strong, SO stretch), 1023, 994, 838, 825, 813, 800; ^{1}H NMR (400 MHz, $CDCl_3$) δ 7.04–6.99 (m, 4 H, 4 × $Ar_{(p\text{-F})}$-CH), 6.87–6.82 (m, 2 H, 2 × $Ar_{(p\text{-OMe})}$-CH), 6.64–6.58 (m, 2 H, 2 × $Ar_{(p\text{-OMe})}$-CH), 5.35 (d, *J* = 7.2 Hz, 1 H, C*H*HOCH$_2$), 5.31 (d, *J* = 7.2 Hz, 1 H, CH*H*OCH$_2$), 5.01 (d, *J* = 7.2 Hz, 1 H, CH$_2$OC*H*H), 4.90 (d, *J* = 7.2 Hz, 1 H, CH$_2$OCH*H*), 3.84 (s, 3 H, OCH_3); ^{13}C NMR (101 MHz, $CDCl_3$) δ 164.7 (d, *J* = 252.5 Hz, Ar-C_q-F), 159.8 (Ar-C_q-OMe), 134.2 (d, *J* = 2.8 Hz, Ar-C_q-SO), 129.0 (2 × $Ar_{(p\text{-OMe})}$-CH), 127.7 (d, J = 8.9 Hz, 2 × $Ar_{(p\text{-F})}$-CH), 125.9 (Ar-C_q-C_q), 115.6 (d, *J* = 22.6 Hz, 2 × $Ar_{(p\text{-F})}$-CH), 113.8 (2 × $Ar_{(p\text{-OMe})}$-CH), 73.3 (CH_2OCH_2), 67.4 (C_q), 55.4 (OCH_3); ^{19}F NMR (377 MHz, $CDCl_3$) δ –107.5; HRMS (TOF-ASAP+) *m/z* calcd for $C_{16}H_{16}SO_3F^+$ $[M+H]^+$: 307.0804, Found: 307.0796.

Synthesis of Oxetane-3-sulfones 17c,d,f

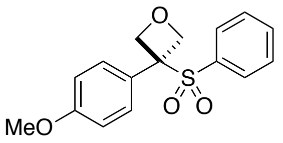

3-(4-Methoxyphenyl)-3-(phenylsulfonyl)oxetane (17c)

m-CPBA (129.4 mg, 0.75 mmol) was added to a solution of 3-(4-methoxyphenyl)-3-(phenylsulfanyl)oxetane **2c** (68.1 mg, 0.25 mmol) in dichloromethane (12.5 mL). The reaction mixture was stirred at 25 °C for 3 h then 3 M $KOH_{(aq)}$ (5 mL) added and the phases separated. Dichloromethane (4 × 5 mL) was added and the layers separated. The organic extracts were combined, dried over Na_2SO_4, filtered and concentrated *in vacuo*. Purification by flash column chromatography (55% Et_2O/pentane) afforded sulfone **17c** (61 mg, 80%) as a white solid; R_f = 0.19 (55% Et_2O/pentane); mp = 145–148 °C; IR (film)/cm^{-1} 2962, 1719, 1615; 1516, 1447, 1303 (S-O), 1265, 1173, 1146 (S-O), 1019, 980, 928, 766, 724, 692; ^1H NMR (400 MHz, $CDCl_3$) δ 7.63–7.57 (m, 1 H, Ph-CH), 7.46–7.38 (m, 4 H, 4 × Ph-CH), 6.81–6.74 (m, 4 H, 4 × Ar-CH), 5.20 (d, *J* = 7.0 Hz, 2 H, C*H*HOC*H*H), 5.04 (d, *J* = 7.0 Hz, 2 H, CH*H*OCH*H*), 3.82 (s, 3 H, OCH_3); ^{13}C NMR (101 MHz, $CDCl_3$) δ 159.9 (Ar-C_q-OMe), 134.8 (Ph-C_q-SO_2), 134.0 (Ph-CH), 129.73 (2 × Ar-CH), 129.68 (2 × Ar-CH), 128.7 (2 × Ar-CH), 126.5 (Ar-C_q-C_q), 113.7 (2 × $Ar_{(p\text{-OMe})}$-CH), 76.6 (CH_2OCH_2), 69.2 (C_q), 55.3 (OCH_3); HRMS (TOF-ASAP+) *m/z* calcd for $C_{16}H_{20}NSO_4^+$ $[M+NH_4]^+$: 322.1113, Found: 322.1108.

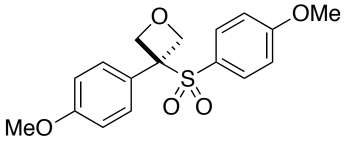

3-(4-Methoxyphenyl)-3-((4-methoxyphenyl)sulfonyl)oxetane (17d)

m-CPBA (129.4 mg, 0.75 mmol) was added to a solution of 3-(4-methoxyphenyl)-3-[(4-methoxyphenyl)sulfanyl]oxetane **2d** (75.6 mg, 0.25 mmol) in dichloromethane (12.5 mL). The reaction mixture was stirred at 25 °C for 3 h then 3 M $KOH_{(aq)}$ (5 mL) added and the phases separated. Dichloromethane (4 × 5 mL) was added and the layers separated. The organic extracts were combined, dried over Na_2SO_4, filtered and concentrated *in vacuo*. Purification by flash column chromatography (70% Et_2O/pentane) afforded sulfone **17d** (68 mg, 81%) as a white solid; R_f = 0.11 (55% Et_2O/pentane); mp = 158–160 °C; IR (film)/cm^{-1} 2961, 1720, 1514, 1297 (S-O), 1257, 1138 (S-O), 1111, 1025, 825, 800, 725, 660; ^1H NMR (400 MHz, $CDCl_3$) δ 7.37–7.33 (m, 2 H, 2 × Ar-CH), 6.87–6.83 (m, 2 H, 2 × Ar-CH), 6.83–6.77 (m, 4 H, 4 × Ar-CH), 5.46 (d, *J* = 6.9 Hz, 2 H, C*H*HOC*H*H), 5.02 (d, *J* = 6.9 Hz, 2 H, CH*H*OCH*H*), 3.86 (s, 3 H, OCH_3), 3.82 (s, 3 H, OCH_3); ^{13}C NMR (101 MHz, $CDCl_3$) δ 164.1 ($Ar_{(p\text{-S})}$-C_q-OMe), 159.8 (Ar-C_q-OMe), 131.9 (2 × Ar-CH), 129.7 (2 × Ar-CH), 126.8 (Ar-C_q-C_q), 126.2 (Ar-C_q-SO_2), 113.9 (2 × Ar-CH), 113.6 (2 × Ar-CH), 76.7 (CH_2OCH_2), 69.1 (C_q), 55.7 (OCH_3), 55.4 (OCH_3); HRMS (TOF-ASAP+) *m/z* calcd for $C_{17}H_{22}NSO_5^+$ $[M+NH_4]^+$: 352.1219, Found: 352.1215.

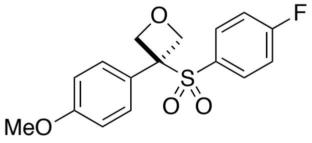

3-((4-Fluorophenyl)sulfonyl)-3-(4-methoxyphenyl)oxetane (17f)

m-CPBA (129.4 mg, 0.75 mmol) was added to a solution of 3-[(4-fluorophenyl)sulfanyl]-3-(4-methoxyphenyl)oxetane **2f** (75.6 mg, 0.25 mmol) in dichloromethane (12.5 mL). The reaction mixture was stirred at 25 °C for 3 h then 3 M $KOH_{(aq)}$ (5 mL) added and the phases separated. Dichloromethane (4 × 5 mL) was added and the layers separated. The organic extracts were combined, dried over Na_2SO_4, filtered and concentrated *in vacuo*. Purification by flash column chromatography (70% Et_2O/pentane) afforded sulfone **17f** (68 mg, 81%) as a white solid; R_f = 0.11 (55% Et_2O/pentane); mp = 129–130 °C; IR (film)/cm^{-1} 2953, 1740, 1588, 1291 (S-O), 1251, 1227, 1146 (S-O), 832, 722, 664; ^1H NMR (400 MHz, $CDCl_3$) δ 7.47–7.40 (m, 2 H, 2 × $Ar_{(p\text{-F})}$-CH), 7.11–7.04 (m, 2 H, 2 × $Ar_{(p\text{-F})}$-CH), 6.83–6.76 (m, 4 H, 4 × $Ar_{(p\text{-OMe})}$-CH), 5.47 (d, *J* = 7.0 Hz, 2 H, C*H*HOC*H*H), 5.04 (d, *J* = 7.0 Hz, 2 H, CH*H*OCH*H*), 3.82 (s, 3 H, OCH_3); ^{13}C NMR (101 MHz, $CDCl_3$) δ 166.1 (d, *J* = 257.6 Hz, Ar-C_q-F), 160.0 (Ar-C_q-OMe), 132.5 (d, *J* = 9.6 Hz, 2 × $Ar_{(p\text{-F})}$-CH), 130.8 (d, *J* = 3.4 Hz, Ar-C_q-SO_2), 129.7 (2 × $Ar_{(p\text{-OMe})}$-CH), 126.3 (Ar-C_q-C_q), 116.2 (d, *J* = 22.6 Hz, 2 × $Ar_{(p\text{-F})}$-CH), 113.8 (2 × $Ar_{(p\text{-OMe})}$-CH), 76.6 (CH_2OCH_2), 69.3 (C_q), 55.4 (OCH_3); ^{19}F NMR (377 MHz, $CDCl_3$) δ –102.4; HRMS (TOF-ASAP+) *m/z* calcd for $C_{16}H_{19}NSFO_4^+$ $[M+NH_4]^+$: 340.1019, Found: 340.1019.

Optimization of a 1-pot Synthesis of Oxetane-3-thiol 18

Table S1. Optimization using triphenylmethanethiol

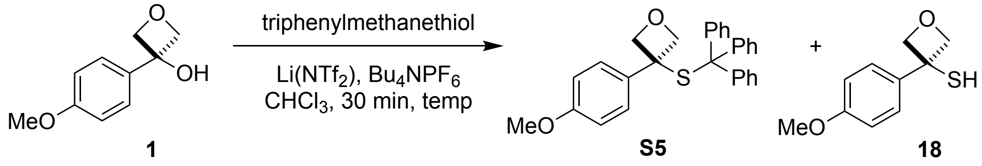

Entry	Equiv of Nucl	Cat/additive loading (mol%)	Temp/ °C	Concentration / M	Yield of thioether S5 / %	Yield of thiol 18/ %	Recovered SM 1/ %	Total 18+S5/ %
1	2	11/5.5	25	0.25	6	0	73	6
2	2	11/5.5	25	0.5	39	11	21	50
3	2	11/5.5	25	1.0	4	0	76	4
4	3	11/5.5	25	0.5	29	10	21	39
5	2	11/5.5	40	0.25	32	8	0	40
6	2	11/5.5	40	0.5	29	13	0	42
7	2	11/5.5	40	1.0	33	8	0	41
8	3	11/5.5	40	0.5	36	11	0	47
**9**	**5**	**11/5.5**	**40**	**0.5**	**42 (50)**	**11 (9)**	**0**	**53 (59)**
10	2	20/10	40	0.5	30	7	0	37
11	2	11/5.5	60	0.25	28	10	0	38
12	2	11/5.5	60	0.5	40	5	0	45
13	2	11/5.5	60	1.0	23	1	0	24
14	3	11/5.5	60	0.5	15	4	0	19

[a]*Insitu yield calculated by* [1]*H NMR using 1,3,5-trimethoxybenzene as an internal standard. Isolated yields in parentheses.*

Different temperatures were explored for the reaction between oxetanol **1** and triphenylmethanethiol. (see table S1). Although some reactivity was observed at 25 °C, the reaction was capricious at this temperature proving sensitive to changes in concentration and equivalents of nucleophile and lab conditions. The optimal temperature was found to be 40 °C although product, albeit in lower yields, was also observed at 60 °C. Changing the concentration of the reaction had little effect on the reaction at 40 °C. Increasing the equivalents of nucleophile from 2 to 5 gave an improved combined yield of product. Further increasing the catalyst loading gave no improvement in yield.

Taking these optimised conditions, deprotection of the trityl group in the same reaction pot was explored (table S2).

Table S2. Optimisation of a 2 step, 1 pot procedure to access oxetane thiol 18

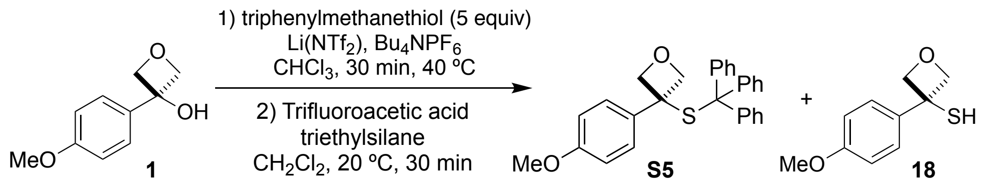

Entry	Equiv of TFA	Equiv of TES	Yield of thiol ether S5 / %	Yield of thiol 18 / %
1	6	1.2	2	51
2	6	2	0	(53)

[a]*Insitu yield calculated by* [1]*H NMR using 1,3,5-trimethoxybenzene as an internal standard. Isolated yields in parentheses.*

For the deprotection step, different equivalents of trifluoroacetic acid (TFA) and triethylsilane (TES) were explored. 6 Equivalents of TFA and 1.2 equivalents of TES gave a 51% yield of desired oxetane thiol **18** and 2% of the trityl protection thioether **S5**. The products proved inseparable by flash column chromatography and so conditions enabling the reaction to reach completion were sought. Increasing the equivalents of TES to 2 led to a 53% isolated yield of the desired oxetane thiol **18** with no intermediate observed.

Synthesis of Oxetane Cysteine Derivative 20

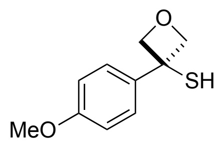

3-(4-Methoxyphenyl)oxetane-3-thiol (18)

Lithium bis(trifluoromethanesulfonimide) (15.8 mg, 0.055 mmol) and tetrabutylammonium hexafluorophosphate (10.6 mg, 0.028 mmol) were added to a solution of oxetanol **1** (90.1 mg, 0.50 mmol) and triphenylmethanethiol (691 mg, 2.5 mmol) in chloroform (1.0 mL). The reaction mixture was stirred at 40 °C for 30 minutes then cooled to 20 °C. Dichloromethane (1.5 mL), triethylsilane (0.16 mL, 1.0 mmol) then trifluroacetic acid (0.23 mL, 3.0 mmol) were added dropwise. The reaction mixture was stirred at 20 °C for 30 min then quenched with sat. aq. $NaHCO_3$ (20 mL). Dichloromethane (3 × 10 mL) was added and the layers separated following each addition. The organic extracts were combined then dried over Na_2SO_4, filtered and concentrated *in vacuo*. Purification by flash column chromatography (15% Et_2O/pentane) afforded thiol **18** (52 mg, 53%) as a white solid; R_f = 0.21 (15% Et_2O/pentane); mp = 59–61 °C; IR (film)/cm^{-1} 2966, 2884, 2544, 1610, 1513, 1458, 1297, 1245, 1179, 1024, 974, 949, 840, 818, 799; ^1H NMR (400 MHz, $CDCl_3$) δ 7.25–7.20 (m, 2 H, 2 × Ar-CH), 6.94–6.89 (m, 2 H, 2 × Ar-CH), 5.25 (d, *J* = 6.4 Hz, 2 H, C*H*HOC*H*H), 4.95 (d, *J* = 6.4 Hz, 2 H, CH*H*OCH*H*), 3.83 (s, 3 H, OCH_3), 2.81 (s, 1 H, SH); ^{13}C NMR (101 MHz, $CDCl_3$) δ 158.8 (Ar-C_q-OMe), 135.9 (Ar-C_q-C_q), 126.7 (2 × Ar-CH), 114.1 (2 × Ar-CH), 86.7 (CH_2OCH_2), 55.3 (OCH_3), 49.2 (C_q); HRMS (TOF ASAP+) *m/z* calcd for $C_{10}H_{11}SO^+$ $[M-H_2O+H]^+$: 179.0531, Found: 179.0535.

Intermediate 3-(4-methoxyphenyl)-3-(tritylthio)oxetane **S5**: mp = 134–136 °C; IR (film)/cm^{-1} 2930, 1509, 1488, 1443, 1305, 1242, 1178, 1029, 983, 773, 745, 706, 698, 676; ^1H NMR (400 MHz, $CDCl_3$) δ 7.44–7.38 (m, 6 H, 6 × Ph-CH), 7.26–7.12 (m, 9 H, 9 × Ph-CH), 7.00–6.94 (m, 2 H, 2 × $Ar_{(p\text{-OMe})}$-CH), 6.77–6.72 (m, 2 H, 2 × $Ar_{(p\text{-OMe})}$-CH), 4.64 (d, *J* = 6.4 Hz, 2 H, CH*H*OCH*H*), 4.43 (d, *J* = 6.4 Hz, 2 H, C*H*HOC*H*H), 3.79 (s, 3 H, OCH_3); ^{13}C NMR (101 MHz, $CDCl_3$) δ 158.4 (Ar-C_q-OMe), 144.1 (3 × Ph-C_q), 136.0 ($Ar_{(p\text{-OMe})}$-C_q-C_q), 129.7 (6 × Ph-CH), 128.0 (2 × $Ar_{(p\text{-OMe})}$-CH), 127.7 (6 × Ph-CH), 126.6 (3 × Ph-CH), 113.6 (2 × $Ar_{(p\text{-OMe})}$-CH), 82.1 (CH_2OCH_2), 68.8 (S-C_q-C_q) 55.4 (OCH_3), 54.7 ($C_{q(oxetane)}$); HRMS (TOF-ASAP+) *m/z* calcd for $C_{29}H_{27}SO_2^+$ $[M+H]^+$: 439.1732, Found: 439.1729.

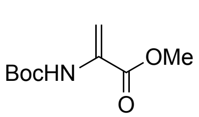

Methyl 2-((*tert*-butoxycarbonyl)amino)acrylate

MsCl (0.77 mL, 10 mmol) was added dropwise to a solution of methyl (*tert*-butoxycarbonyl)serinate (1.01 g, 5 mmol), triethylamine (2.8 mL, 20 mmol) in dichloromethane (30 mL). The reaction mixture was stirred at 25 °C for 3 h then quenched with sat. aq. $NaHCO_3$ (20 mL). Dichloromethane (3 × 15 mL) was added and the layers separated. The organic extracts were combined, dried over Na_2SO_4, filtered and concentrated *in vacuo*. Purification by flash column chromatography (15% Et_2O/pentane) afforded acrylate (950 mg, 94%) as a pale yellow oil. R_f = 0.49 (15% Et_2O/pentane); IR (film)/cm^{-1} 3424, 2980, 1714 (C=O), 1508, 1440, 1368, 1238, 1202, 1323, 1154, 1065, 884; ^1H NMR (400 MHz, $CDCl_3$) δ 7.02 (s, 1 H, NH), 6.16 (s, 1 H, CH*H*), 5.73 (d, *J* = 1.5 Hz, 1 H, C*H*H), 3.84 (s, 3 H, OCH_3), 1.49 (s, 9 H, C(C*H*$_3$)$_3$); ^{13}C NMR (101 MHz, $CDCl_3$) δ 164.4 (*C*(O)OMe), 152.5 (*C*(O)C(CH_3)$_3$), 131.3 (*C*=CH_2), 105.2 (C=*C*H_2), 80.7 (*C*(CH_3)$_3$), 52.9 (OCH_3), 28.2 (C(*C*H_3)$_3$). The observed spectroscopic data for this compound was consistent with that previously reported.[19]

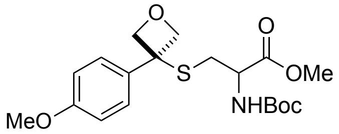

Methyl *N*-(*tert*-butoxycarbonyl)-*S*-(3-(4-methoxyphenyl)oxetan-3-yl)cysteinate (19)

Methyl 2-((*tert*-butoxycarbonyl)amino)acrylate (121 mg, 0.6 mmol) was added to a stirring solution of thiol **18** (98 mg, 0.5 mmol) and triethylamine (209 µL, 1.5 mmol) in methanol (2.0 mL). The reaction mixture was stirred at 25 °C for 90 minutes then quenched with sat. aq. NH_4Cl (10 mL). The aqueous portion was extracted with EtOAc (3 × 15 mL). The organic portions were combined, washed with water (25 mL), dried over Na_2SO_4, filtered and concentrated *in vacuo*. Purification by flash column chromatography (50% Et_2O/pentane) afforded oxetane **19** (190 mg, 100%) as a colourless oil; R_f = 0.22 (50% Et_2O/pentane); IR (film)/cm^{-1} 2953, 1745 (C=O), 1709 (C=O), 1513, 1246, 1162, 909, 729; ^1H NMR (400 MHz, $CDCl_3$) δ 7.17–7.11 (m, 2 H, 2 × Ar-CH), 6.94–6.89 (m, 2 H, 2 × Ar-CH), 5.21–5.16 (m, 3 H, C*H*HOC*H*H + NH), 4.88 (dd, *J* = 6.4, 4.2 Hz, 2 H, CH*H*OCH*H*), 4.51-

–4.41 (m, 1 H, SCH_2CH), 3.84 (s, 3 H, $ArOCH_3$), 3.74 (s, 3 H, $C(O)OCH_3$), 2.85 (dd, *J* = 13.2, 5.0 Hz, 1 H, SC*H*H), 2.80 (dd, *J* = 13.2, 5.6 Hz, 1 H, SCH*H*), 1.46 (s, 9 H, $C(CH_3)_3$); ^{13}C NMR (101 MHz, $CDCl_3$) δ 176.6 ($C(O)OC(CH_3)_3$), 171.2 (*C*(O)OMe), 158.8 (Ar-C_q-OMe), 133.8 (Ar-C_q-C_q), 127.6 (2 × Ar-CH), 114.0 (2 × Ar-CH), 83.1 (CH_2OCH_2), 80.3 ($C(CH_3)_3$), 55.3 ($ArOCH_3$), 53.0 (SCH_2CH), 52.6 ($C(O)OCH_3$), 52.2 (C_q), 32.7 (SCH_2), 28.3 ($C(CH_3)_3$); FTMS (+p NSI) *m/z* calcd for $C_{19}H_{31}N_2O_6S^+$ $[M+NH_4]^+$: 415.1897, Found: 415.1898.

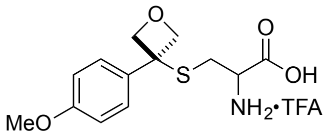

S-(3-(4-Methoxyphenyl)oxetan-3-yl)cysteine trifluoroacetate salt (20)

Methyl *N*-(*tert*-butoxycarbonyl)-*S*-(3-(4-methoxyphenyl)oxetan-3-yl)cysteinate **19** (99 mg, 0.25 mmol) and lithium hydroxide monohydrate (12 mg, 0.275 mmol) in MeCN (1.25 mL) and water (1.25 mL) was stirred at 25 °C for 16 h. Water (10 mL) was added and the aqueous layer washed with EtOAc (2 × 10 mL). The aqueous layer was acidified to pH 2 with 4 M HCl and the product was extracted with EtOAc (3 × 10 mL). The organic extracts were combined then dried over Na_2SO_4, filtered and concentrated *in vacuo*. The crude product was then dissolved in dichloromethane (1 mL) and trifluoracetic acid (0.19 mL, 2.5 mmol) was added. The reaction mixture was stirred at 25 °C for 2 h then concentrated *in vacuo* to afford oxetane **20** (52 mg, 52%) as a beige solid. IR (film)/cm^{-1} 2953 (br. OH), 1737 (C=O), 1667, 1610, 1513, 1247, 1178, 1134, 830, 796, 721; ^1H NMR (400 MHz, CD_3OD) δ 7.26–7.20 (m, 2 H, 2 × Ar-CH), 6.99–6.96 (m, 2 H, 2 × Ar-CH), 5.26 (d, *J* = 6.8 Hz, 1 H, C*H*HOCH$_2$), 5.18 (d, *J* = 6.8 Hz, 1 H, CH$_2$OC*H*H), 4.91 (dd, *J* = 6.7, 2.1 Hz, 2 H, CH*H*OCH*H*), 3.82 (s, 3 H, OCH_3), 3.74 (dd, *J* = 7.8, 4.3 Hz, 1 H, SCH_2CH), 3.05 (dd, *J* = 14.3, 4.4 Hz, 1 H, SC*H*H), 2.91 (dd, *J* = 14.3, 7.9 Hz, 1 H, SCH*H*); ^{13}C NMR (101 MHz, CD_3OD) δ 168.7 (*C*(O)OH), 161.2 (q, J_{C-F} = 34.7 Hz, $C(O)CF_3$), 159.2 (Ar-C_q-OMe), 133.4 (Ar-C_q-C_q), 127.6 (2 × Ar-CH), 116.5 (q, J_{C-F} = 285.4 Hz, $C(O)CF_3$), 113.9 (2 × Ar-CH), 82.9 (CH_2OCH_2), 82.8 (CH_2OCH_2), 54.4 ($ArOCH_3$), 52.5 (SCH_2CH), 52.1 (C_q), 30.0 (SCH_2); FTMS (+p NSI) *m/z* calcd for $C_{13}H_{17}NO_4SNa^+$ $[M+Na]^+$: 306.0769, Found: 306.0770.

Synthesis of Thioester 21 and Benzylic Sulfide 22

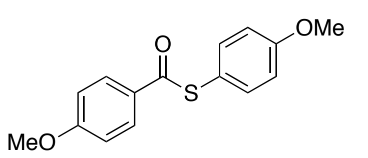

***S*-(4-Methoxyphenyl) 4-methoxybenzothioate (21)**

4-Methoxybenzoyl chloride (256 mg, 1.5 mmol) in MeCN (2 mL) then pyridine (0.24 mL, 3 mmol) were added dropwise to a solution of 4-methoxythiophenol (0.18 mL, 1.5 mmol) in MeCN (3.0 mL) at 0 °C. The reaction mixture was stirred at 25 °C for 2 h then concentrated *in vacuo*. Dichloromethane (3 × 5 mL) was added and the layers separated. Water (10 mL) was added and the product was extracted with EtOAc (2 × 10 mL). The organic extracts were combined, washed with aq. NaOH (1 M, 10 mL), water (10 mL) and brine (10 mL) then dried over Na_2SO_4, filtered and concentrated *in vacuo*. Purification by flash column chromatography (30% Et_2O/pentane) afforded thioester **21** (342 mg, 83%) as a white solid. R_f = 0.30 (20% Et_2O/pentane); mp = 133–135 °C; IR (film)/cm^{-1} 2969, 1662 (C=O), 1593, 1251, 1213, 1166, 1023, 905, 824, 792; ^1H NMR (400 MHz, $CDCl_3$) δ 8.03–7.98 (m, 2 H, 2 × Ar-CH), 7.44–7.39 (m, 2 H, 2 × Ar-CH), 7.03–6.94 (m, 4 H, 4 × Ar-CH), 3.89 (s, 3 H, OCH_3), 3.86 (s, 3 H, OCH_3); ^{13}C NMR (101 MHz, $CDCl_3$) δ 188.9 (C=O), 163.9 (Ar-C_q-C(O)S), 160.6 (2 × Ar-C_q-OMe), 136.7 (2 × $Ar_{(p\text{-OMe})}$-CH), 129.7 (2 × $Ar_{(p\text{-OMe})}$-CH), 129.5 (Ar-C_q-S), 114.9 (2 × $Ar_{(p\text{-OMe})}$-CH), 113.9 (2 × $Ar_{(p\text{-OMe})}$-CH), 55.5 (OCH_3), 55.4 (OCH_3); HRMS (TOF-ASAP+) *m/z* calcd for $C_{15}H_{15}SO_3^+$ $[M+H]^+$: 275.0742, Found: 275.0742.

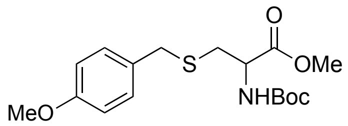

Methyl *N*-(*tert*-butoxycarbonyl)-*S*-(4-methoxybenzyl)cysteinate (S6)

Methyl 2-((*tert*-butoxycarbonyl)amino)acrylate (604 mg, 3.0 mmol) was added to a stirring solution of 4-Methoxybenzyl mercaptan (0.35 mL, 2.5 mmol) and triethylamine (1.0 mL, 7.5 mmol) in methanol (10 mL). The reaction mixture was stirred at 25 °C for 90 min then quenched with sat. aq. NH_4Cl (50 mL). The aqueous portion was extracted with EtOAc (3 × 50 mL). The organic portions were combined, washed with water (50 mL), dried over Na_2SO_4, filtered and concentrated *in vacuo*. Purification by flash column chromatography (40% Et_2O/pentane) afforded methyl *N*-(*tert*-butoxycarbonyl)-*S*-(4-methoxybenzyl)cysteinate **S6** (660 mg, 74%) as a white solid; R_f = 0.30 (40% Et_2O/pentane); mp = 81–83 °C; IR (film)/cm^{-1} 3366 (NH), 2971, 1732 (C=O), 1680 (C=O), 1512, 1234, 1293, 1249, 1163, 1031, 1011, 832, 824; ^1H NMR (400 MHz, $CDCl_3$) δ 7.26–7.20 (m, 2 H, 2 × Ar-CH), 6.89–6.83 (m, 2 H, 2 × Ar-CH), 5.30 (d, *J* = 8.1 Hz, 1 H, NH), 4.56 (m, 1 H, C*H*NH), 3.82 (s, 3 H, $ArOCH_3$), 3.77 (s, 3 H, C(O)OCH_3), 3.70 (Ar-CH_2S), 2.70 (m, 2 H, SCH_2CH), 1.48 (s, 9 H, C(CH_3)$_3$); ^{13}C NMR (101 MHz, $CDCl_3$) δ 171.6 (*C*(O)OMe), 158.8 (Ar-C_q-OMe), 155.0 (*C*(O)OC(CH_3)$_3$), 130.0 (2 × Ar-CH) 129.6 (Ar-C_q-C_q), 114.0 (2 × Ar-CH), 80.1 (*C*(CH_3)$_3$), 55.3 ($ArOCH_3$), 53.1 (SCH_2*C*H), 52.5 (C(O)O*C*H_3), 36.0 (Ar-CH_2S), 33.5 (S*C*H_2CH), 28.3 (C(*C*H_3)$_3$); FTMS (+p NSI) *m/z* calcd for $C_{17}H_{26}NO_5S^+$ $[M+H]^+$: 356.1526, Found: 356.1530.

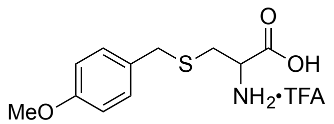

***S*-(4-Methoxybenzyl)cysteine trifluoroacetate salt (22)**

Methyl *N*-(*tert*-butoxycarbonyl)-*S*-(4-methoxybenzyl)cysteinate (356 mg, 1.0 mmol) and lithium hydroxide monohydrate (46 mg, 1.1 mmol) in MeCN (5 mL) and water (5 mL) was stirred at 25 ºC for 16 h. Water (15 mL) was added and the aqueous layer washed with EtOAc (2 × 15 mL). The aqueous layer was acidified to pH 2 with 4 M HCl and the product was extracted with EtOAc (3 × 15 mL). The organic extracts were combined then dried over Na_2SO_4, filtered and concentrated *in vacuo*. The crude product was then dissolved in dichloromethane (4 mL) and trifluoracetic acid (0.77 mL, 10 mmol) was added. The reaction mixture was stirred at 25 ºC for 2 h then concentrated *in vacuo* to afford cysteine derivative **22** (305 mg, 86%) as a white solid. IR (film)/cm^{-1} 2906 (br. OH), 1744 (C=O), 1654, 1612, 1512, 1431, 1135, 1243, 1032, 819; ^1H NMR (400 MHz, CD_3OD) δ 7.32–7.28 (m, 2 H, 2 × Ar-CH), 6.92–6.88 (m, 2 H, 2 × Ar-CH), 4.07 (dd, *J* = 8.1, 4.2 Hz, 1 H, SCH_2C*H*), 3.82–3.78 (m, 5 H, $ArCH_2$ + OCH_3), 3.06 (dd, *J* = 14.9, 4.2 Hz, 1 H, SC*H*H), 2.91 (dd, *J* = 14.9, 8.1 Hz, 1 H, SCH*H*); ^{13}C NMR (101 MHz, CD_3OD) δ 169.3 (*C*(O)OH), 159.1 (Ar-C_q-OMe), 129.9 (2 × Ar-CH), 129.1 (Ar-C_q-C_q), 113.7 (2 × Ar-CH), 54.3 ($ArOCH_3$), 52.0 (SCH_2*C*H), 34.9 ($ArCH_2$), 30.9 (SCH_2); FTMS (+p NSI) *m/z* calcd for $C_{11}H_{15}NO_3SNa^+$ $[M+Na]^+$: 264.0665, Found: 264.0667.

References

[1] R. A. Croft, J. J. Mousseau, C. Choi, J. A. Bull, *Chem. Eur. J.* **2016**, *22*, 16271–16276.

[2] K. Nemoto, S. Tanaka, M. Konno, S. Onozawa, M. Chiba, Y. Tanaka, Y. Sasaki, R. Okubo, T. Hattori, *Tetrahedron*, **2016**, *72*, 734–745.

[3] J. Hutton, W. A. Waters, *J. Chem. Soc.,* **1965**, 4253–4256.

[4] S. Xu, X. Huang, X. Hong, B. Xu, *Org. Lett.* **2012**, *14*, 4614–4617.

[5] J. Yan, T. Ni, F. Yan, *Tetrahedron Letters*, **2015**, *56*, 1096–1098.

[6] G. Li, E. Wang, H. Chen, H. Li, Y. Liu, P. G. Wang, *Tetrahedron*, **2008**, *64*, 9033–9043.

[7] A. M. Tickner, G. K. Huang, K. Gombatz, R. J. Mills, V. Novack, K. S. Webb, *Synthetic Communications*, **1995** , *25*, 2497–2505

[8] Tran, K.; Berlin, K. D.; Holt, E. M.; Hallford, R.; Eastman, M. A.; Yu, V. K.; Praliev, K. D, *Phosphorus, Sulfur Silicon Relat. Elem.*, **2005**, *180*, 53-66.

[9] Sabatier, M. C*omptes Rendus Hebdomadaires des Seances de l'Academie des Sciences*, **1904**, *138*, 1322.

[10] (a) Guijarro, A.; Ramon, D. J.; Yus, M. *Tetrahedron*, **1993**, *49*, 469-482. (b) Korenaga, T.; Ko, A.; Uotani, K.; Tanaka, Y.; Sakai, T. *Angew. Chem. Int. Ed.,* **2011**, *50*, 10703-10707.

[11] Wilt, J. W.; Roberts, D. D. *J. Org. Chem.*, **1962**, *27*, 3430–3434.

[12] (a) Smith, A. *Canadian Journal of Chemistry*, **1968**, *46*, 1561. (b) Holland, H. L.; Kindermann, M.; Kumaresan, S.; Stefanac, T. *Tetrahedron: Asymmetry*, **1993**, *4*, 1353-1364.

[13] (a) Trachtenberg, E. N.; Carver, J. R. *J. Org. Chem*. **1970**, *35*, 1646–1653. (b) Hamon, D. P. G.; Tuck, K. L. *Tetrahedron*, **2000**, *56*, 4829–4835.

[14] (a) Kornblum, N.; Ackermann, P.; Manthey, J. W.; Musser, M. T.; Pinnick, H. W.; Saraswathi, S.; Wade, P.A. *J. Org. Chem.*, **1988**, *53*, 1475–1481. (b) Engel, P.; Ying, A. *J. Am. Chem. Soc.*, **2011**, *123*, 3706–3715. (c) Girijavallabhan, V.; Alvarez, C.; Njoroge, F. G. *J. Org. Chem.*, **2011**, *76*, 6442–6446.

[15] (a) Yoon, N. M.; Choi, J.; Ahn, J. H. *J. Org. Chem.*, **1994**, *59*, 3490–3493. (b) Sakai, N.; Moritaka, K.; Konakahara, T. *Chem. Eur. J.*, **2009**, *24*, 4123–4127.

[16] Inada, Y.; Nishibayashi, Y.; Hidai, M.; Uemura, S. *J. Am. Chem. Soc.*, **2002**, *124*, 15172–15173.

[17] Katritzky, A. R.; Yang, Z.; Lam, J. N.; Cundy, D. J. *Heterocycles*, **1993**, *36*, 1367–1373.

[18] (a) Hopkins, P. B.; Fuchs, P. L. *J. Org. Chem.*; **1978**, *43*, 1208–1217. (b) Du, J.; Zheng, R.; Li, X. *J. Chem. Res.*, **2005**, *3*, 180–181. (c) Banerjee, S.; Adak, L.; Ranu, B. C. *Tetrahedron Lett.*, **2012**, *53*, 2149–2152.

[19] R. Ramesh, K. De, S. Chandrasekaran, *Tetrahedron*, **2007**, *63*, 10534–10542.

^{1}H and ^{13}C NMR spectra of selected compounds

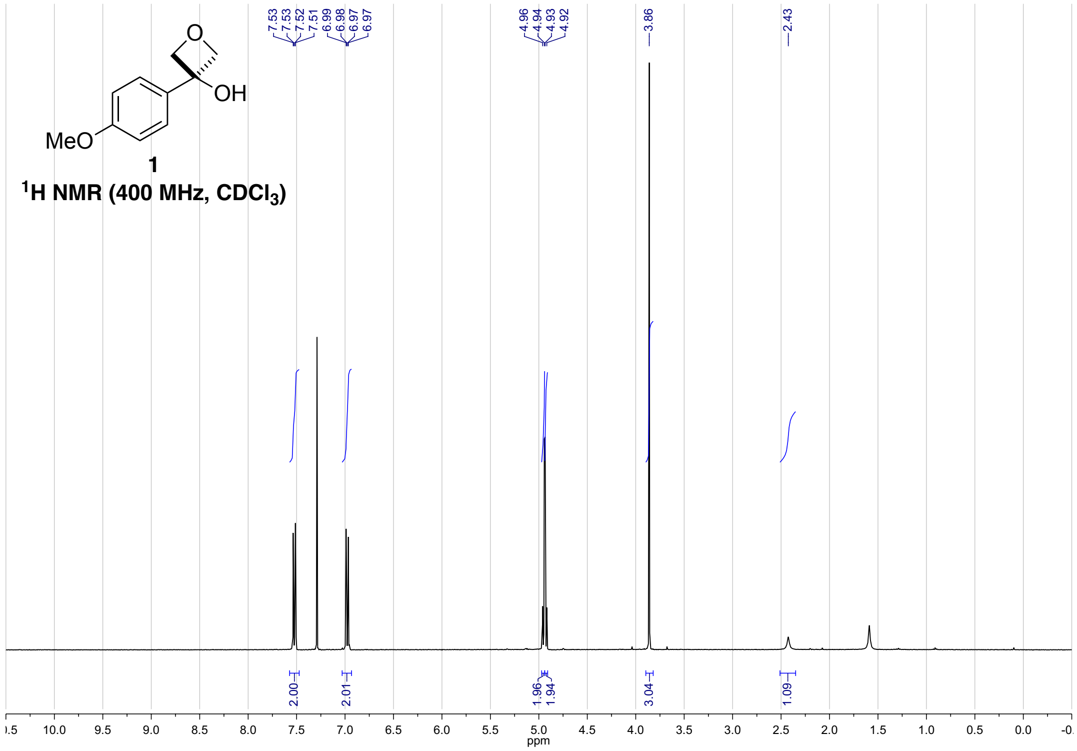
7.53
7.53
7.52
7.51
6.99
6.98
6.97
6.97
4.96
4.94
4.93
4.92
3.86
2.43
MeO
OH
O
1
1H NMR (400 MHz, CDCl3)
2.00
2.01
1.96
1.94
3.04
1.09
ppm

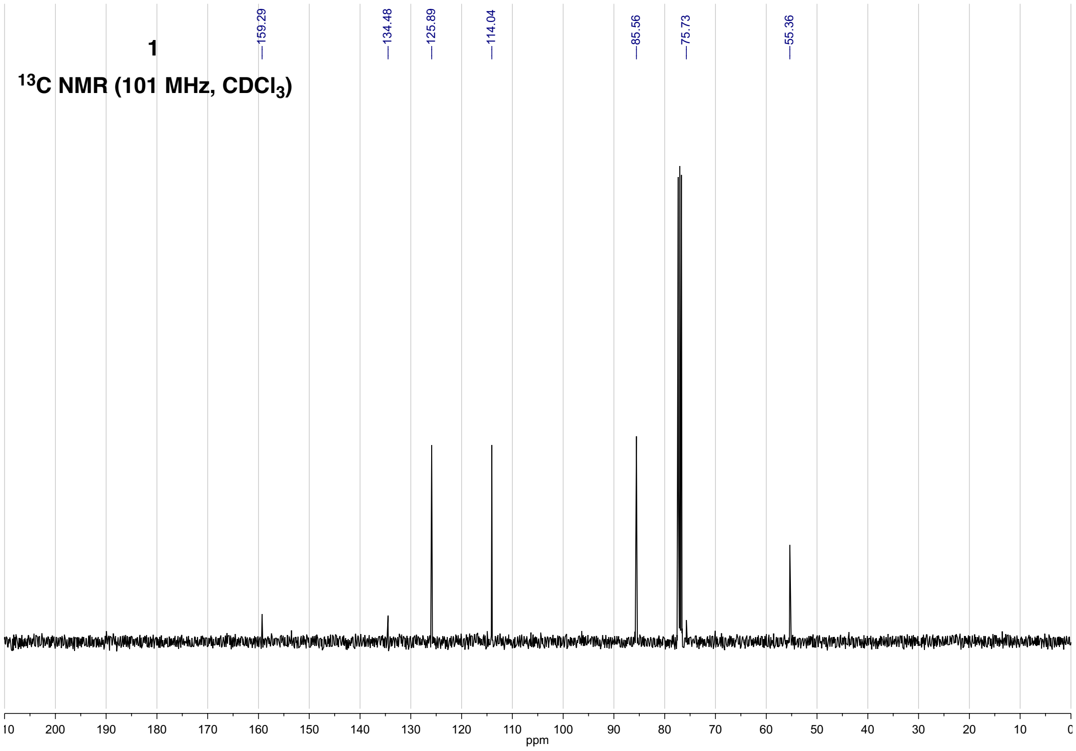
159.29
134.48
125.89
114.04
85.56
75.73
55.36
1
13C NMR (101 MHz, CDCl3)
ppm

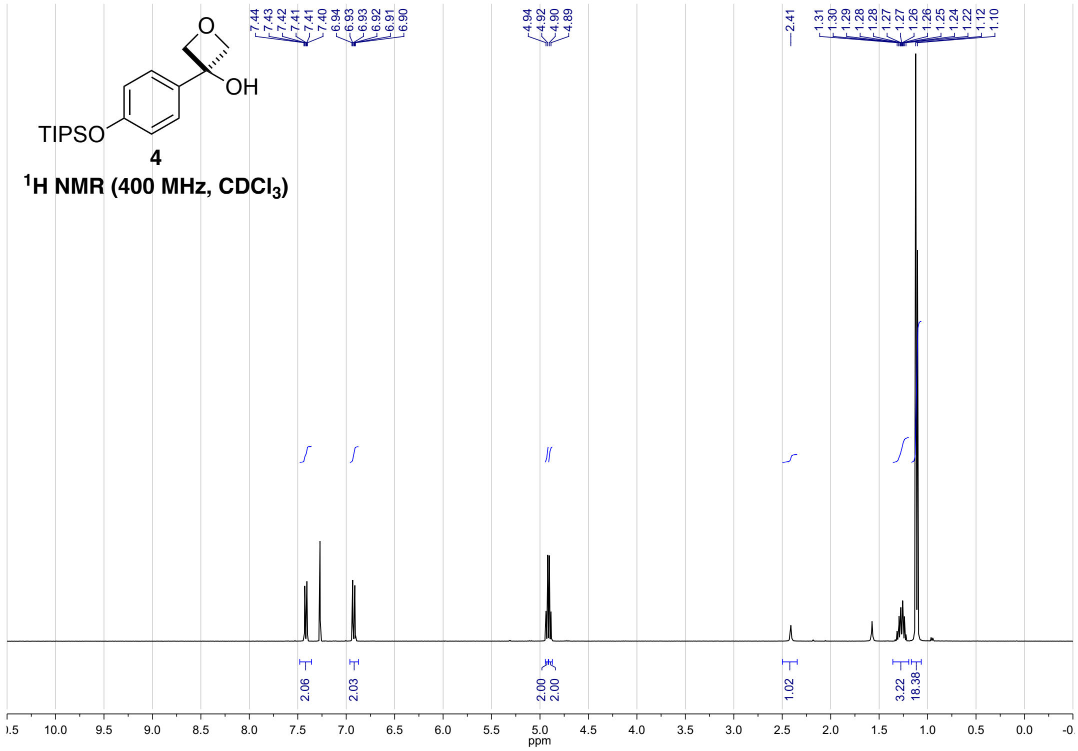
TIPSO
OH
O
4
1H NMR (400 MHz, CDCl3)
ppm

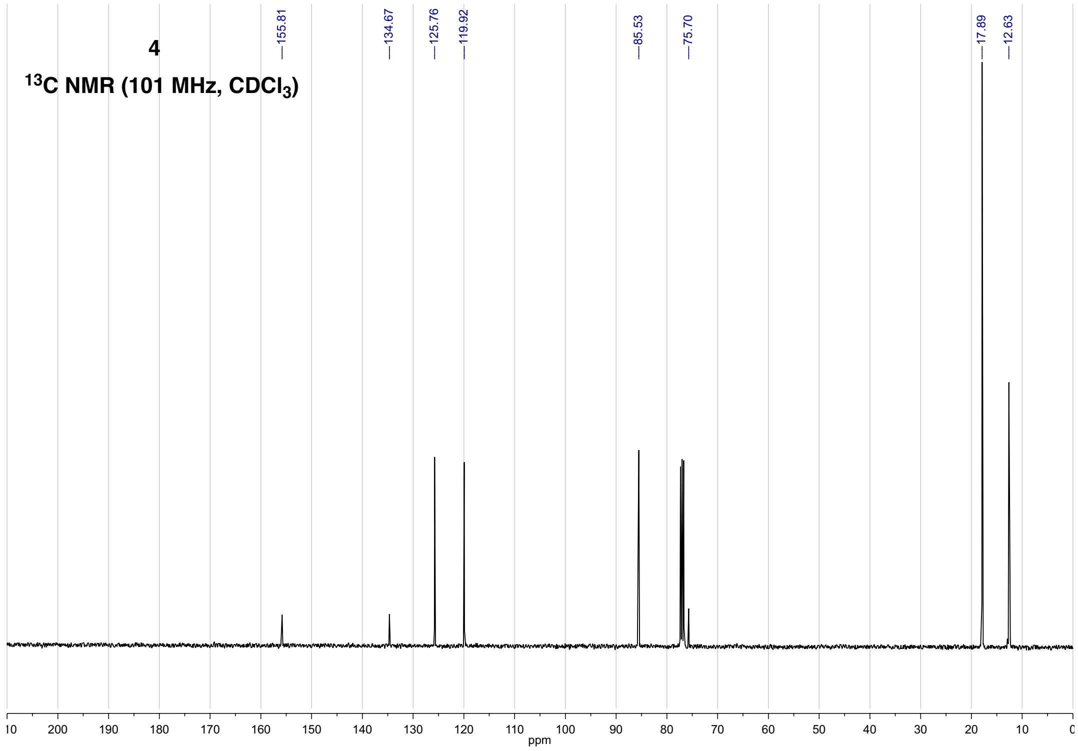
4
13C NMR (101 MHz, CDCl3)
155.81
134.67
125.76
119.92
85.53
75.70
17.89
12.63
ppm

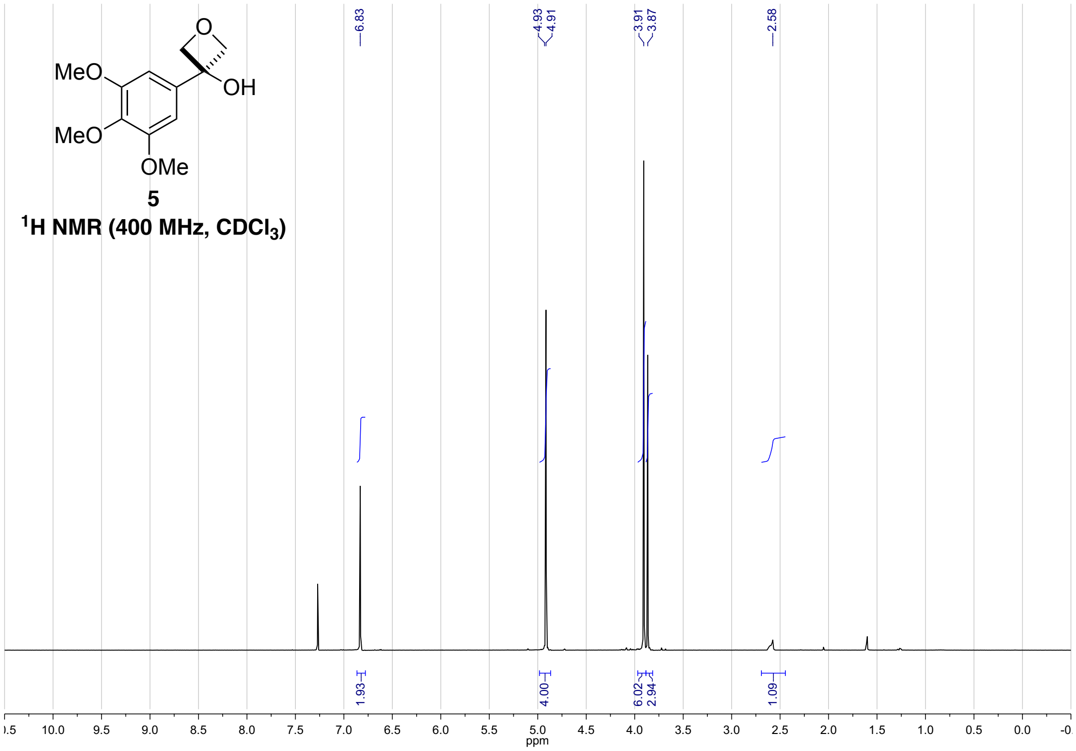

5

^{1}H NMR (400 MHz, $CDCl_3$)

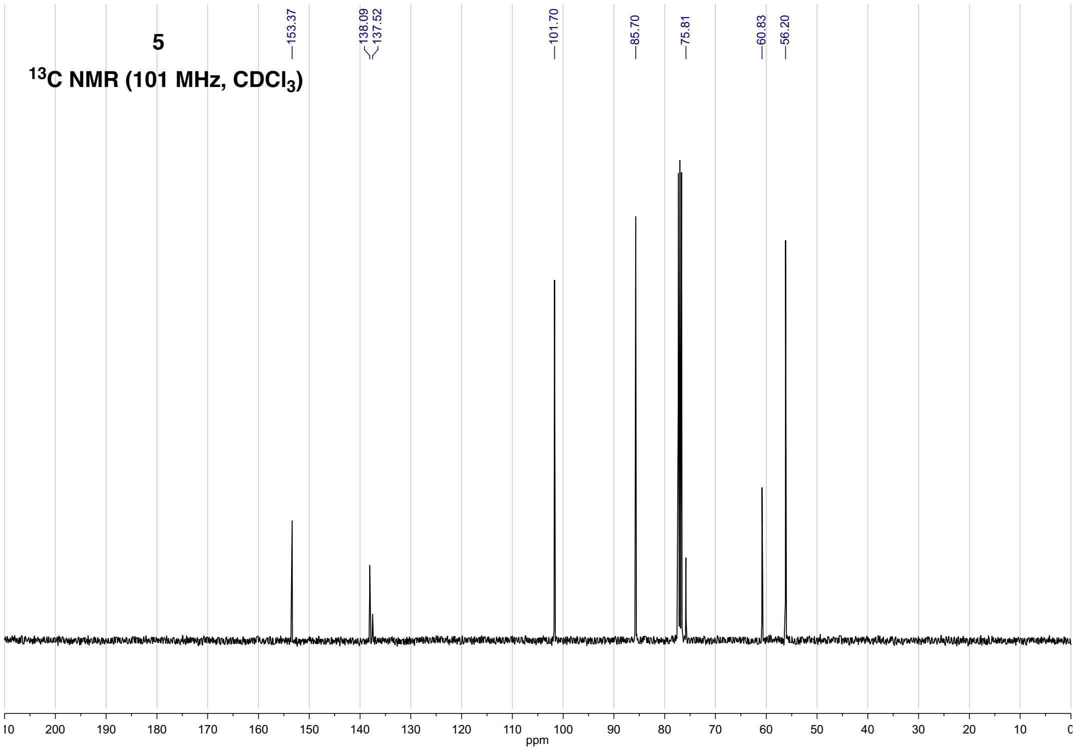

5

^{13}C NMR (101 MHz, $CDCl_3$)

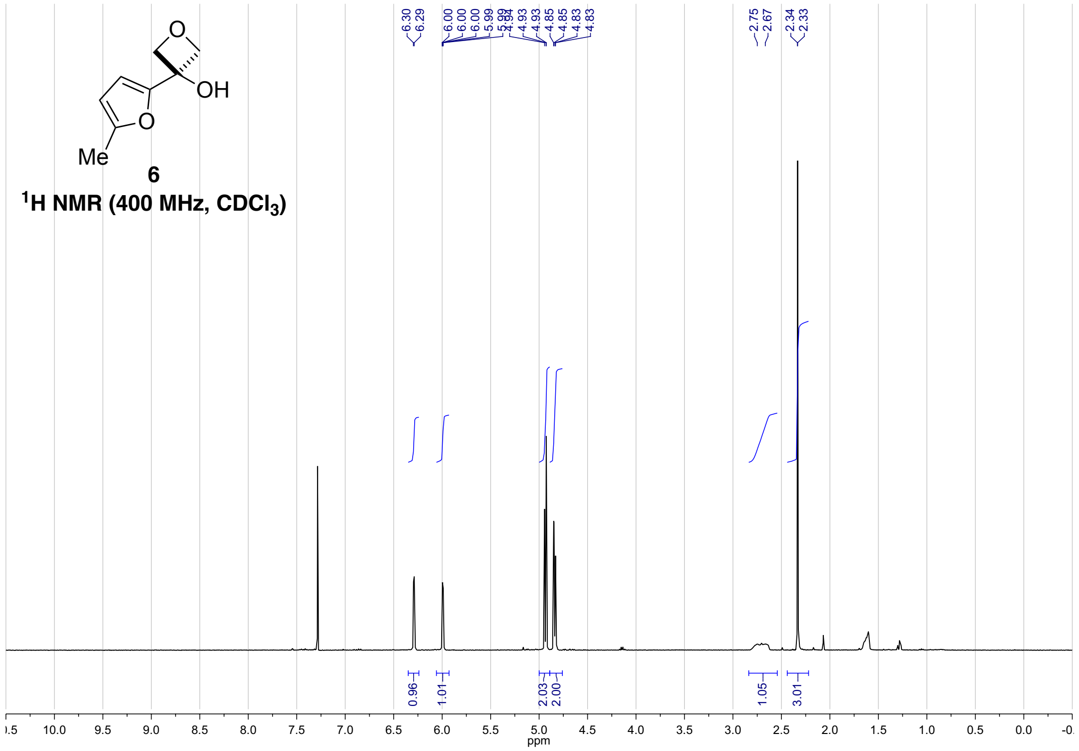
OH
O
Me
6
1H NMR (400 MHz, CDCl3)
6.30
6.29
6.00
6.00
6.00
5.99
5.99
4.94
4.93
4.93
4.85
4.85
4.83
4.83
2.75
2.67
2.34
2.33
0.96
1.01
2.03
2.00
1.05
3.01
ppm

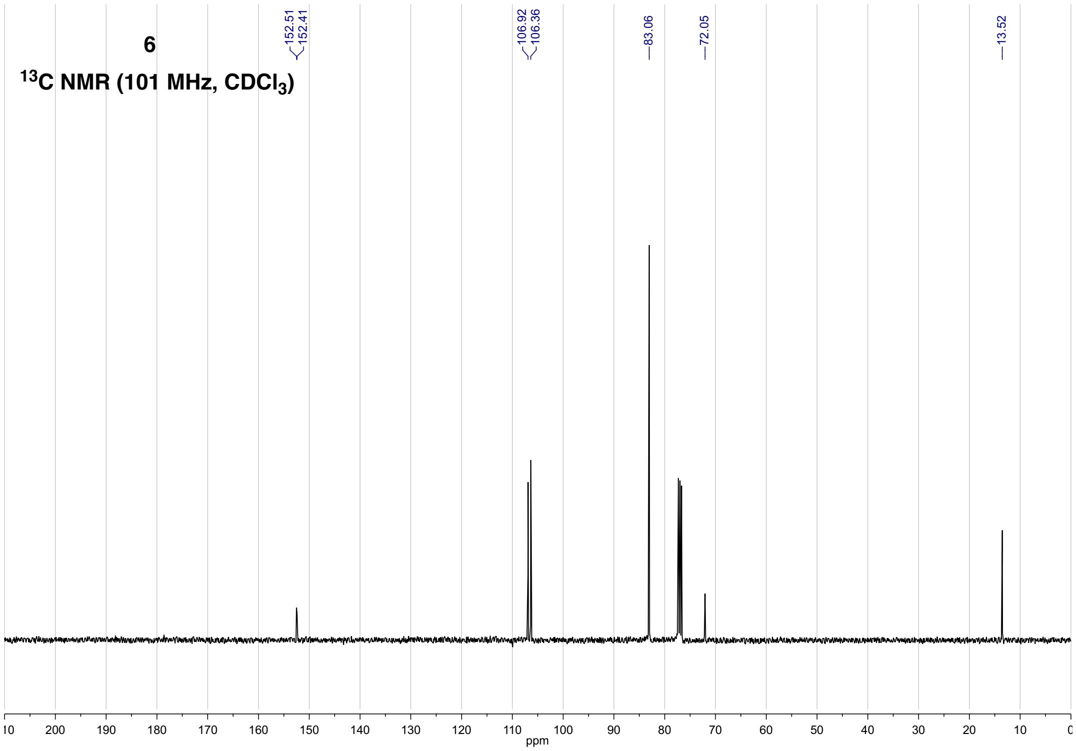
6
13C NMR (101 MHz, CDCl3)
152.51
152.41
106.92
106.36
83.06
72.05
13.52
ppm

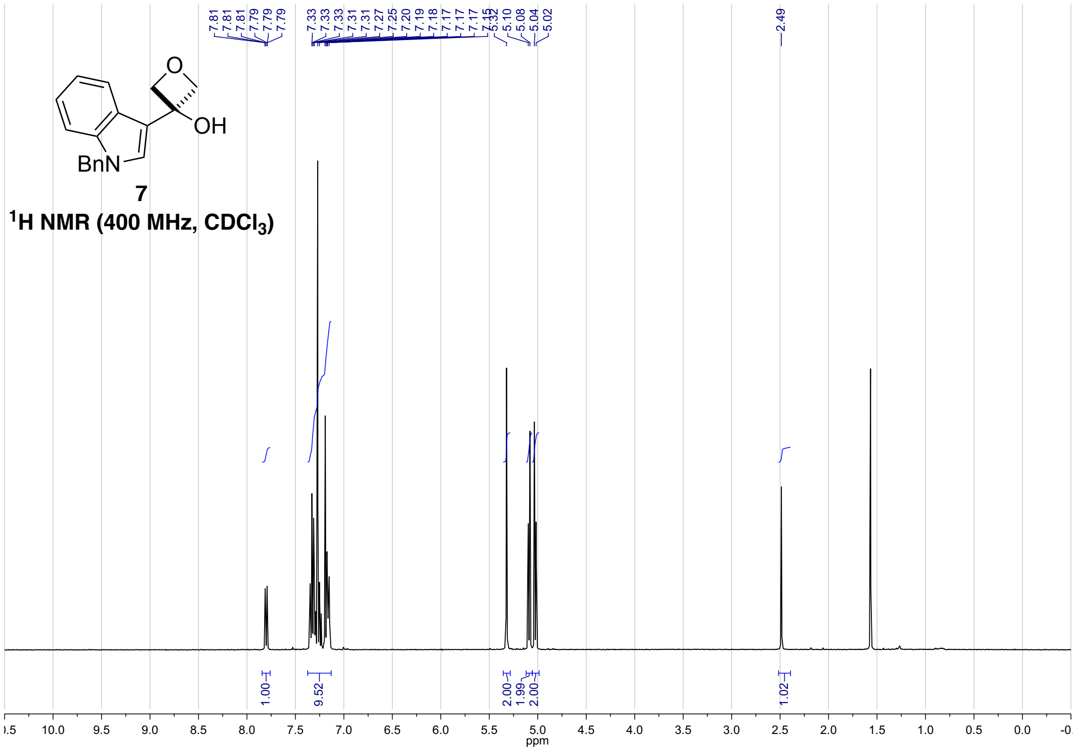
O
OH
BnN
7
^{1}H NMR (400 MHz, CDCl$_3$)
ppm

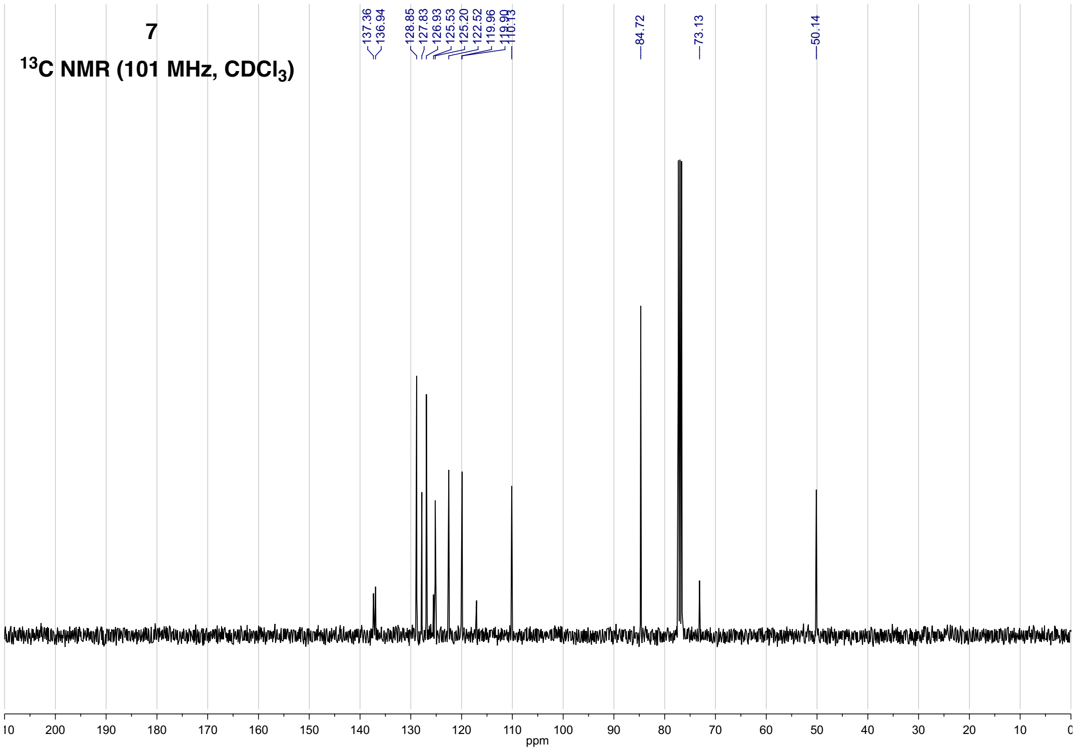
7
^{13}C NMR (101 MHz, CDCl$_3$)
ppm

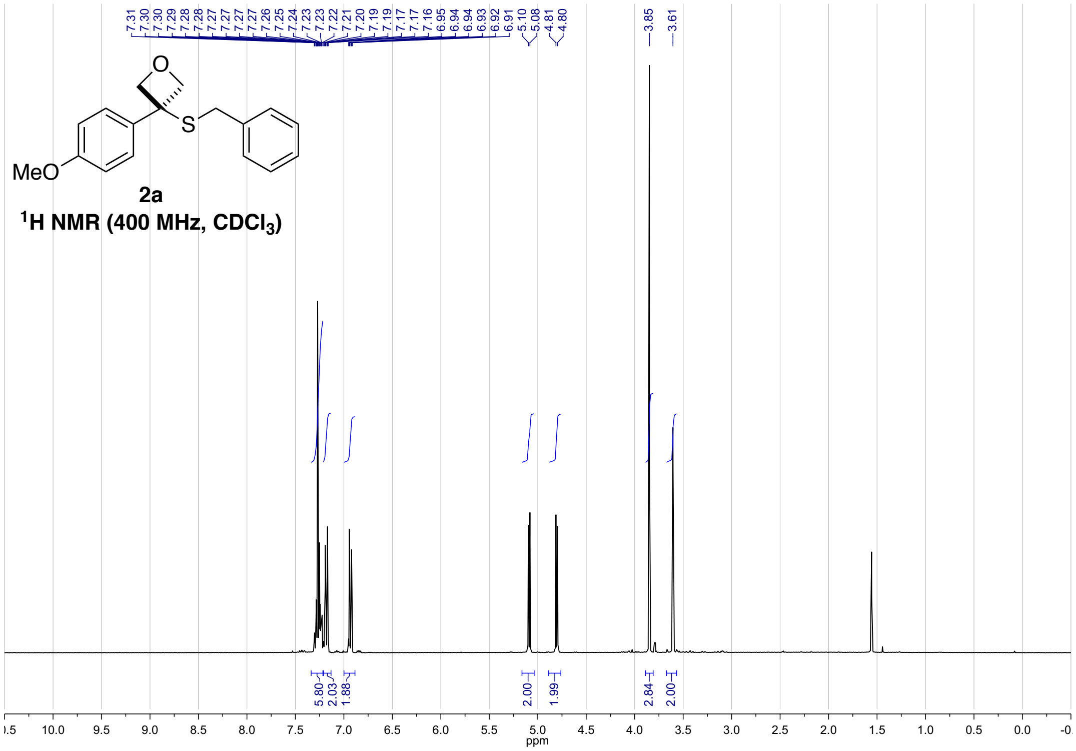
7.31 7.30 7.30 7.29 7.28 7.28 7.27 7.27 7.27 7.27 7.26 7.25 7.24 7.23 7.23 7.22 7.21 7.20 7.19 7.19 7.17 7.17 7.16 6.95 6.94 6.94 6.93 6.92 6.91 5.10 5.08 4.81 4.80
3.85
3.61
MeO
S
O
2a
^{1}H NMR (400 MHz, $CDCl_3$)
5.80 2.03 1.88 2.00 1.99 2.84 2.00
ppm

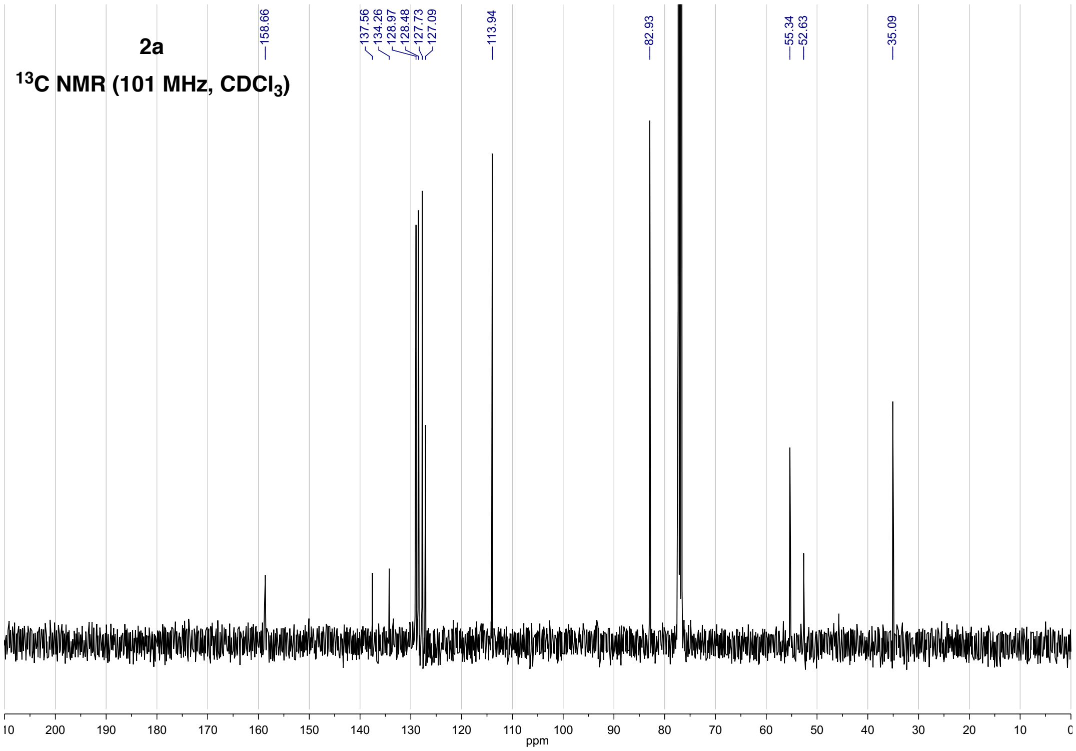
158.66
137.56 134.26 128.97 128.48 127.73 127.09
113.94
82.93
55.34 52.63
35.09
2a
^{13}C NMR (101 MHz, $CDCl_3$)
ppm

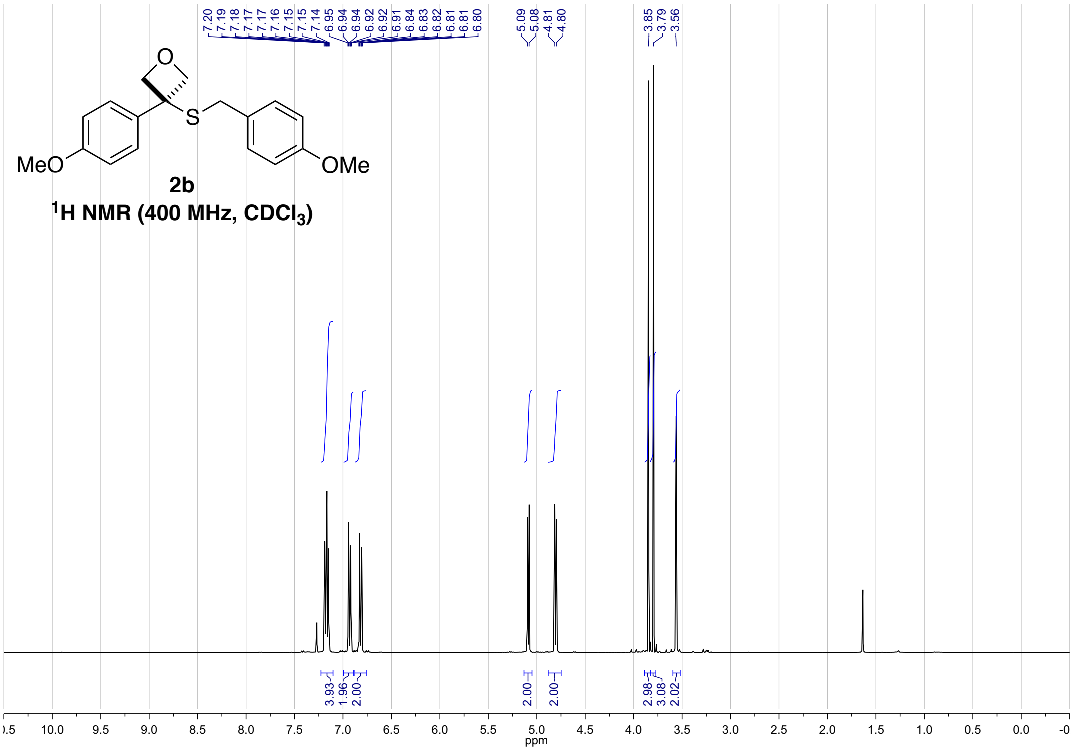

7.20 7.19 7.18 7.17 7.17 7.16 7.15 7.15 7.14 6.95 6.94 6.94 6.92 6.92 6.91 6.84 6.83 6.82 6.81 6.81 6.80
5.09 5.08 4.81 4.80
3.85 3.79 3.56
MeO
OMe
O
S
2b
1H NMR (400 MHz, CDCl3)
3.93 1.96 2.00 2.00 2.00 2.98 3.08 2.02
ppm

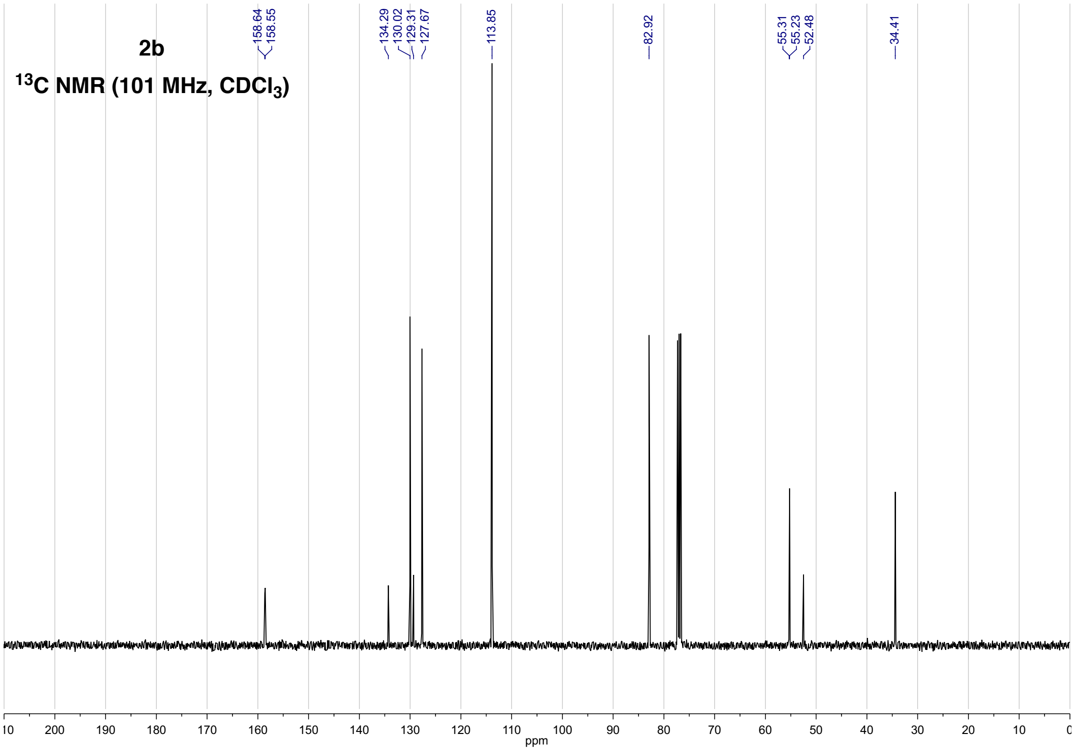

158.64 158.55
134.29 130.02 129.31 127.67
113.85
82.92
55.31 55.23 52.48
34.41
2b
13C NMR (101 MHz, CDCl3)
ppm

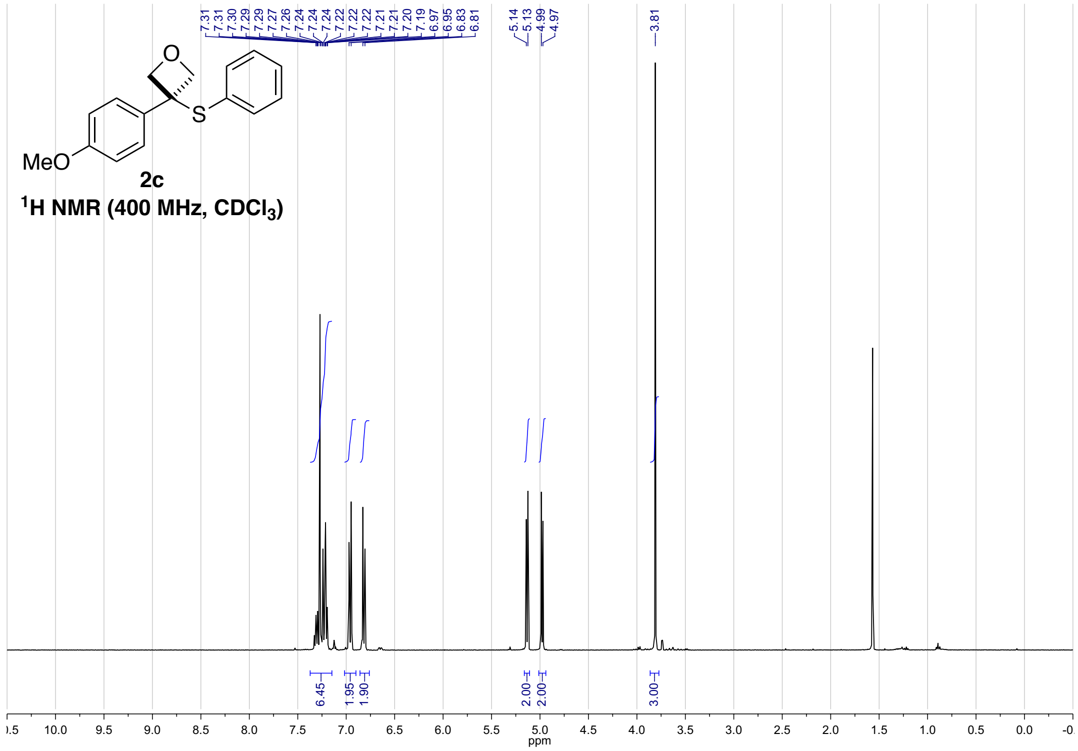
MeO
O
S
2c
^{1}H NMR (400 MHz, $CDCl_3$)
7.31 7.31 7.30 7.29 7.29 7.27 7.26 7.24 7.24 7.24 7.22 7.22 7.22 7.21 7.21 7.20 7.19 6.97 6.95 6.83 6.81
5.14 5.13 4.99 4.97
3.81
6.45 1.95 1.90 2.00 2.00 3.00
ppm

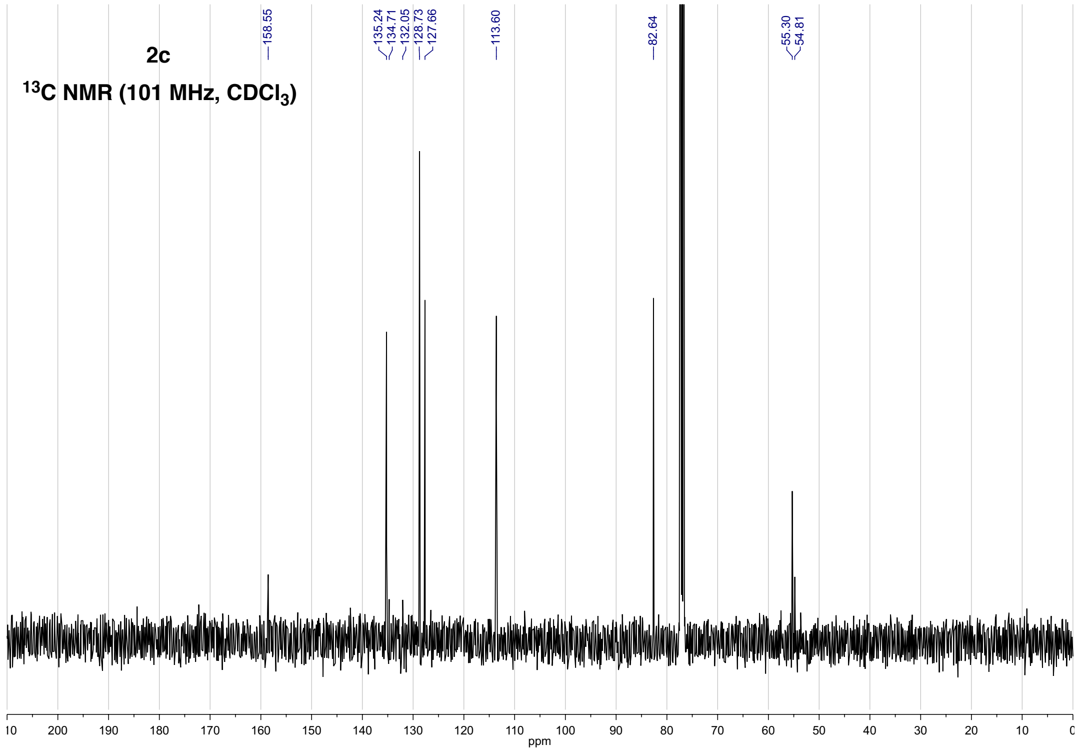
2c
^{13}C NMR (101 MHz, $CDCl_3$)
158.55
135.24 134.71 132.05 128.73 127.66
113.60
82.64
55.30 54.81
ppm

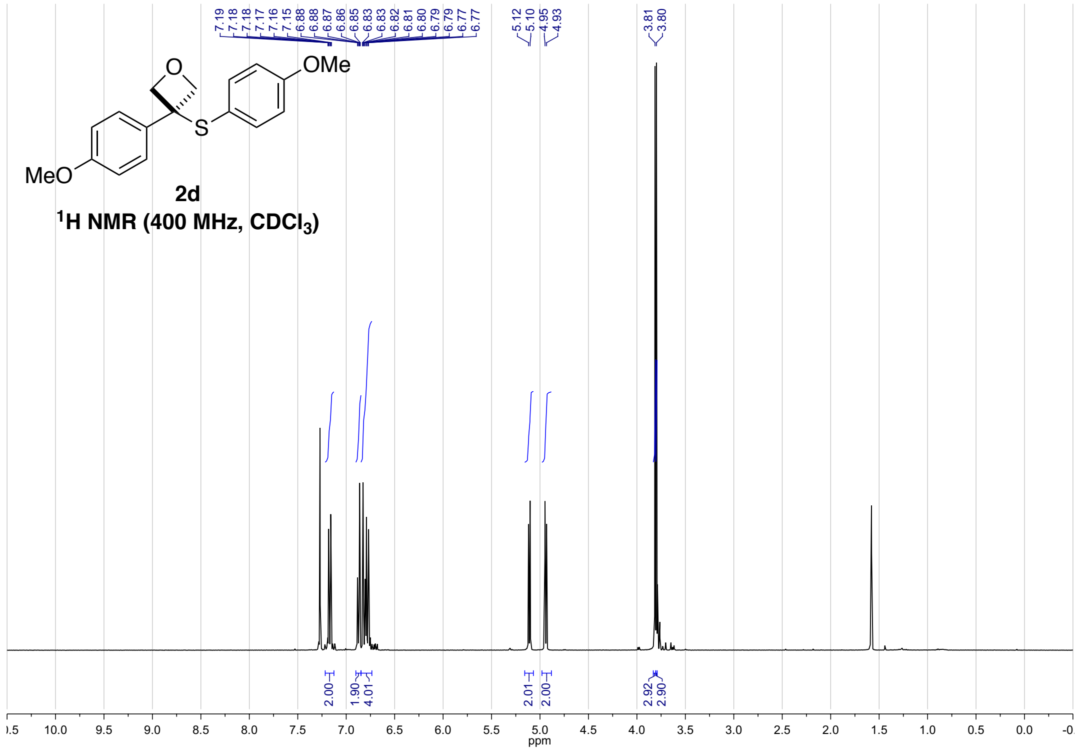
OMe
MeO
2d
^{1}H NMR (400 MHz, CDCl$_3$)
ppm

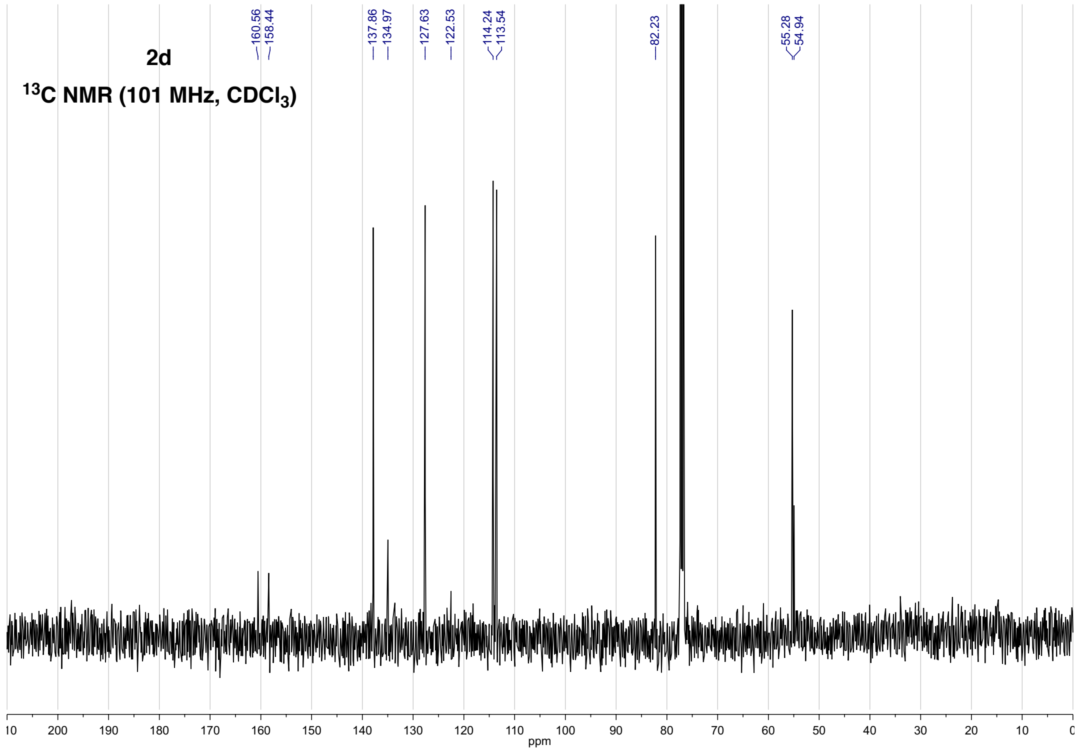
2d
^{13}C NMR (101 MHz, CDCl$_3$)
ppm

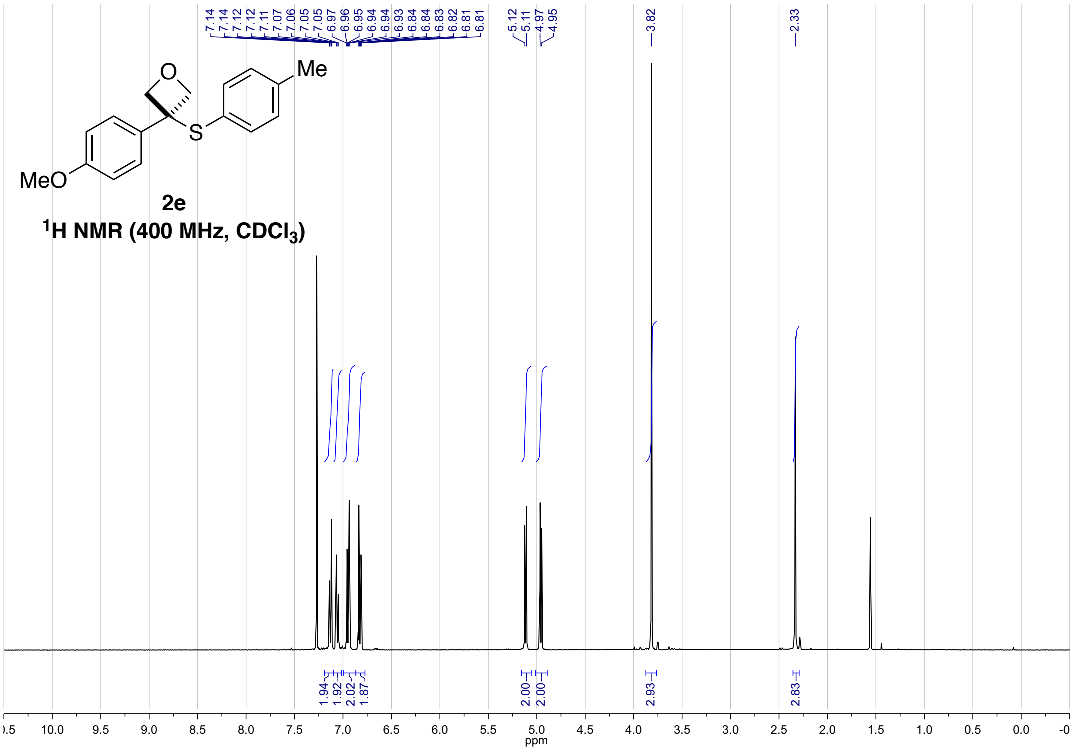
7.14
7.14
7.12
7.12
7.11
7.07
7.06
7.05
7.05
6.97
6.96
6.95
6.94
6.94
6.93
6.84
6.84
6.83
6.82
6.81
6.81
5.12
5.11
4.97
4.95
3.82
2.33
Me
S
O
MeO
2e
1H NMR (400 MHz, CDCl3)
1.94
1.92
2.02
1.87
2.00
2.00
2.93
2.83
ppm

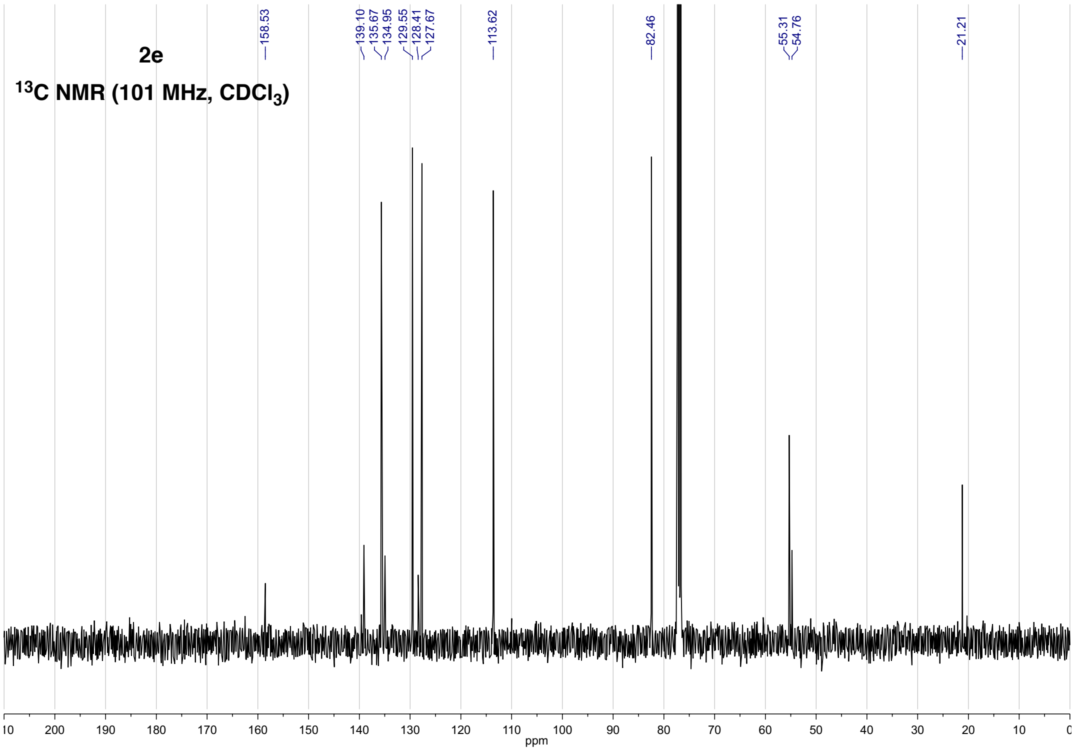
158.53
139.10
135.67
134.95
129.55
128.41
127.67
113.62
82.46
55.31
54.76
21.21
2e
13C NMR (101 MHz, CDCl3)
ppm

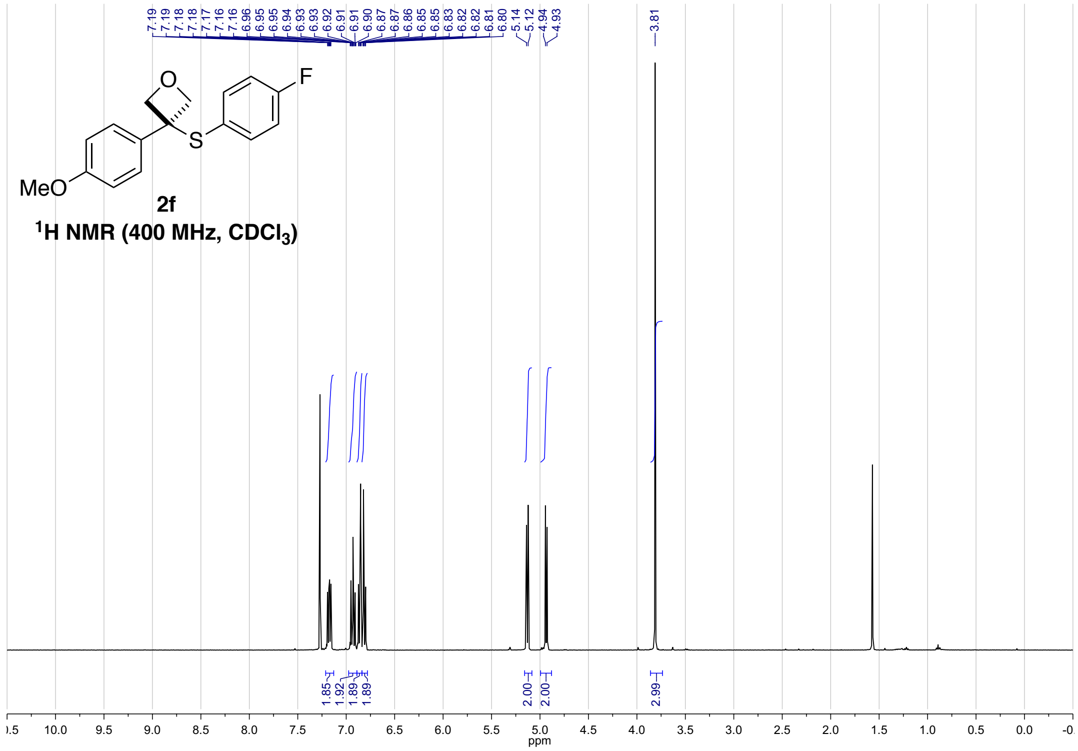
MeO
O
S
F
2f
^{1}H NMR (400 MHz, CDCl$_3$)
ppm

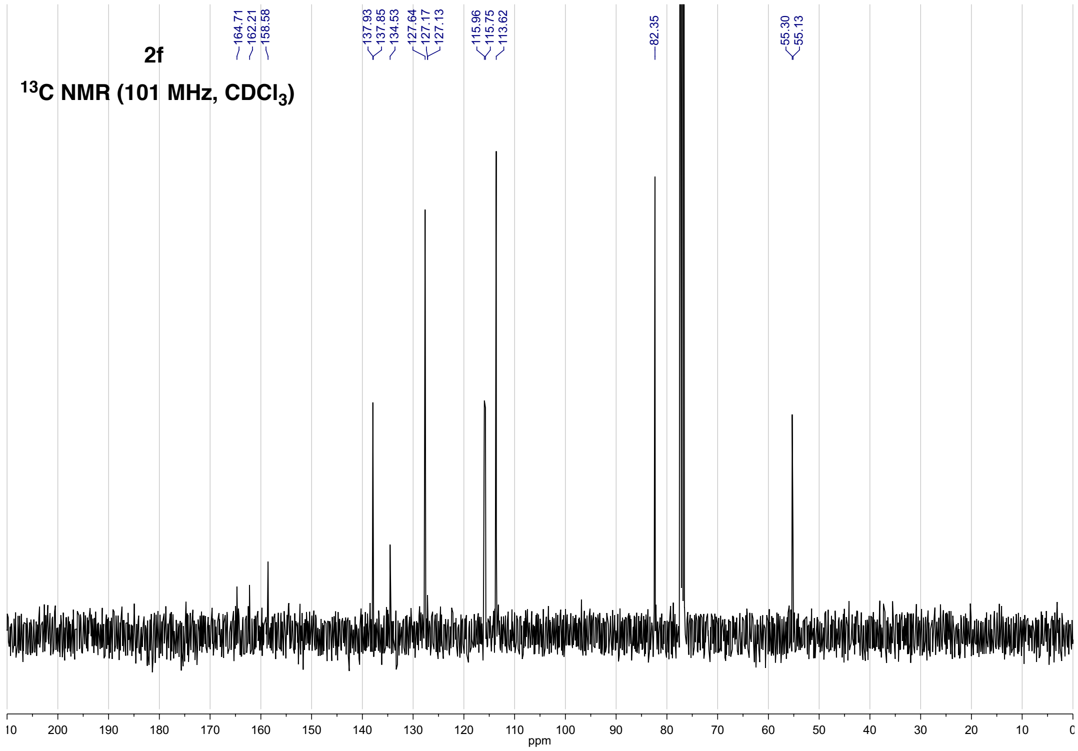
2f
^{13}C NMR (101 MHz, CDCl$_3$)
ppm

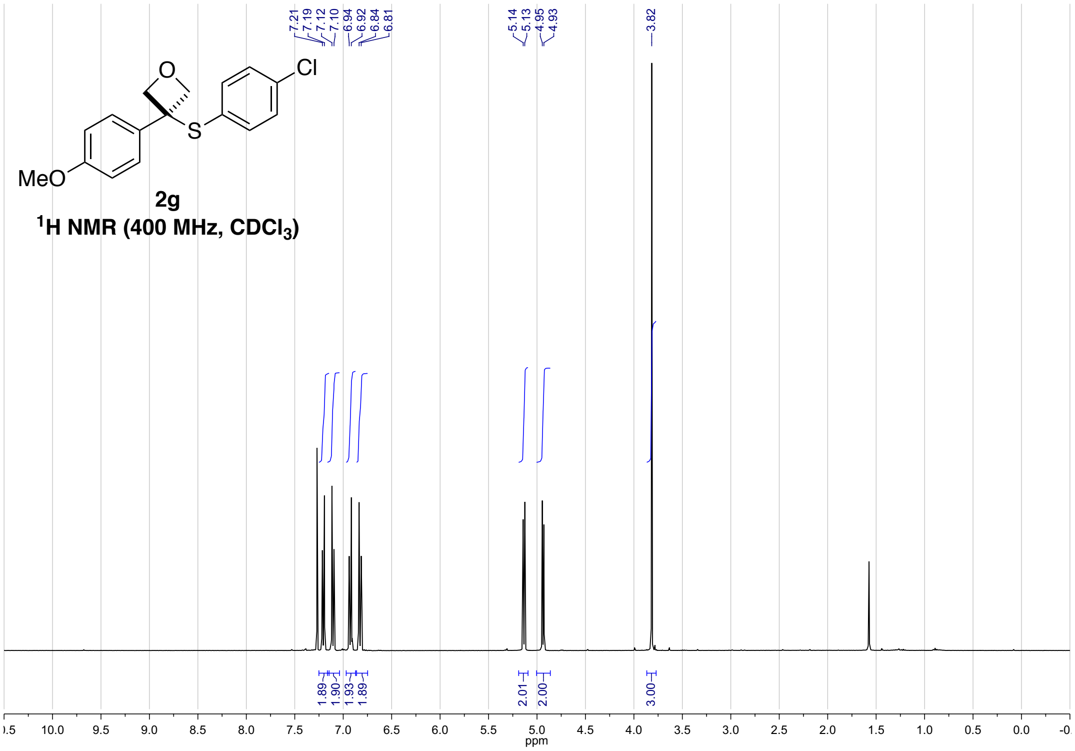

2g
^{1}H NMR (400 MHz, CDCl$_3$)
MeO
O
S
Cl
7.21 7.19 7.12 7.10 6.94 6.92 6.84 6.81
5.14 5.13 4.95 4.93
3.82
1.89 1.90 1.93 1.89
2.01 2.00
3.00
ppm

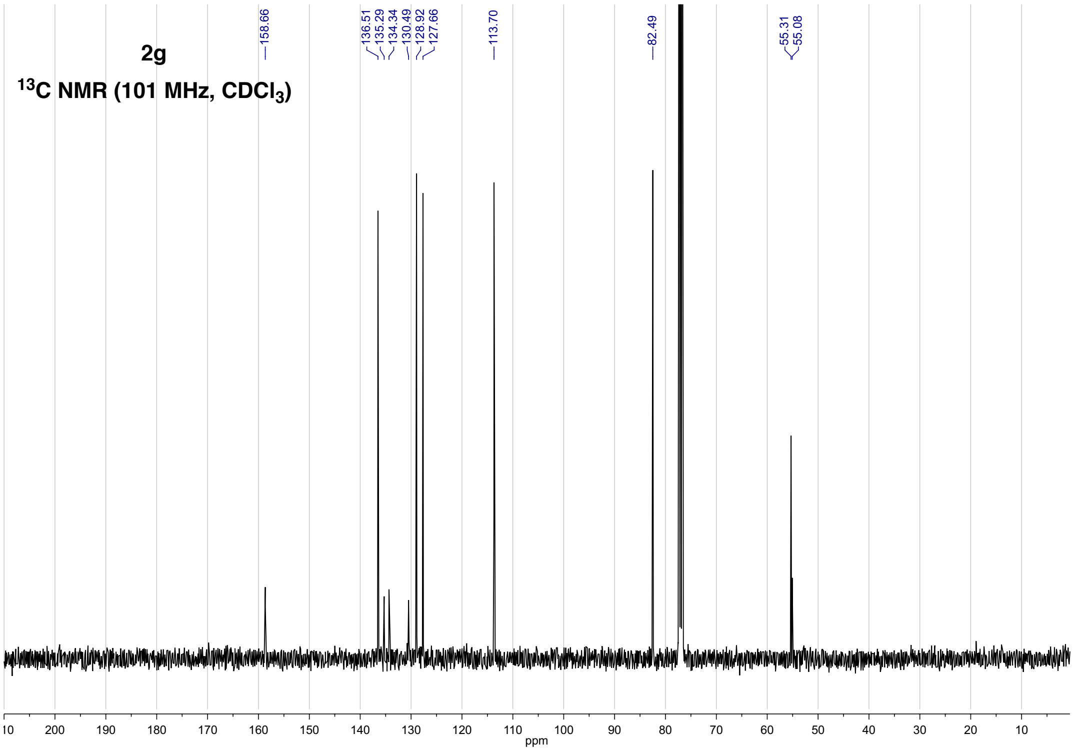

2g
^{13}C NMR (101 MHz, CDCl$_3$)
158.66
136.51 135.29 134.34 130.49 128.92 127.66
113.70
82.49
55.31 55.08
ppm

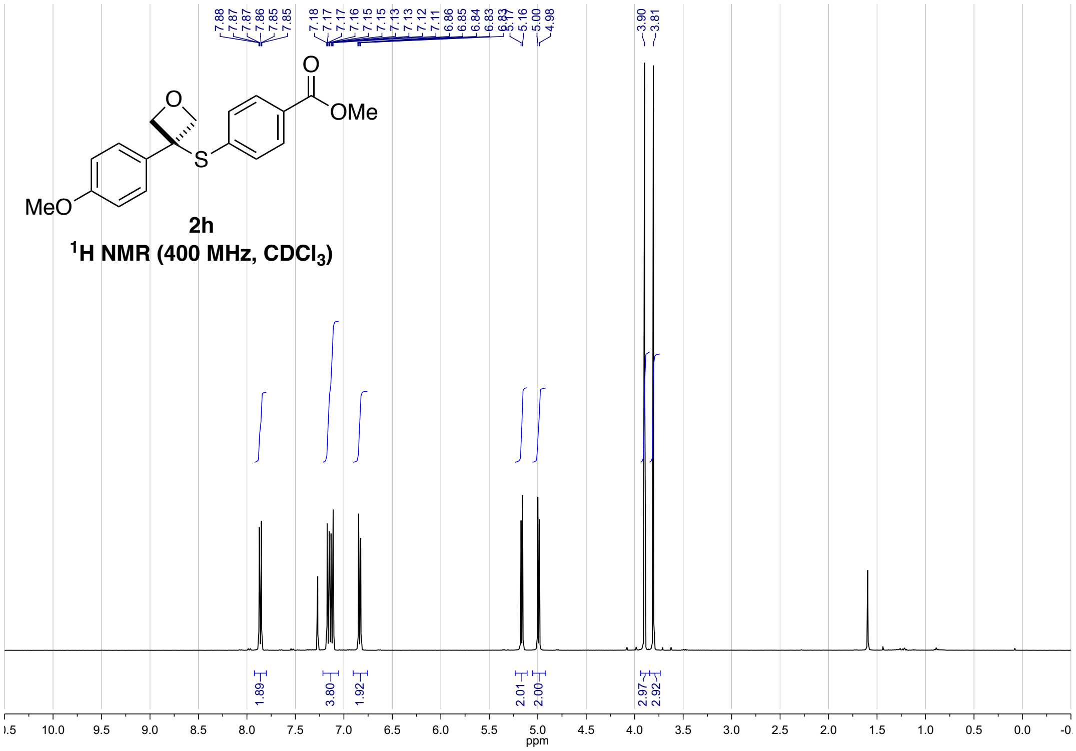
2h
^{1}H NMR (400 MHz, $CDCl_3$)
ppm

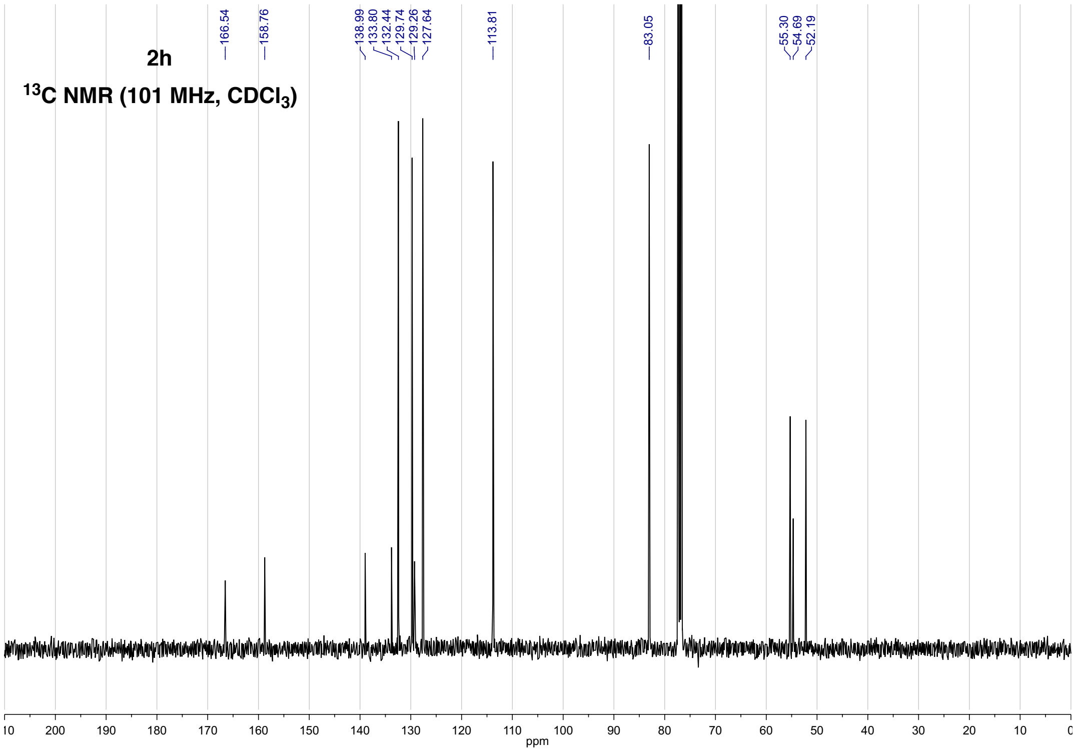
2h
^{13}C NMR (101 MHz, $CDCl_3$)
ppm

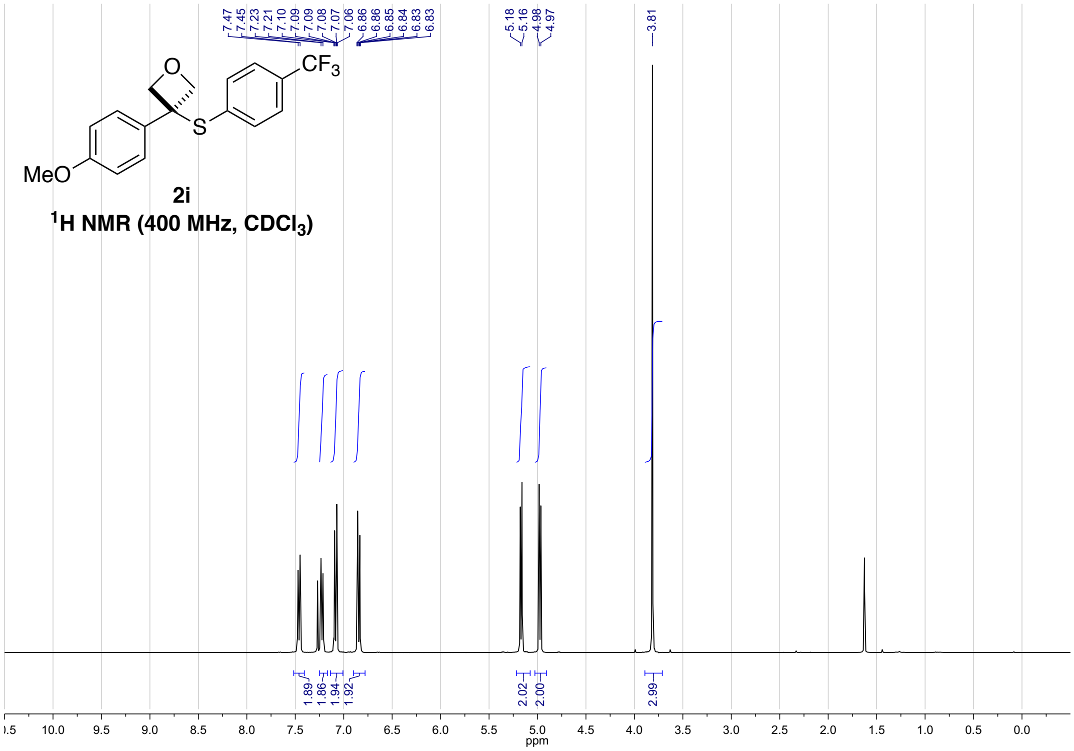

2i
^{1}H NMR (400 MHz, CDCl$_3$)
7.47 7.45 7.23 7.21 7.10 7.09 7.09 7.08 7.07 7.06 6.86 6.86 6.85 6.84 6.83 6.83
5.18 5.16 4.98 4.97
3.81
1.89 1.86 1.94 1.92 2.02 2.00 2.99
ppm

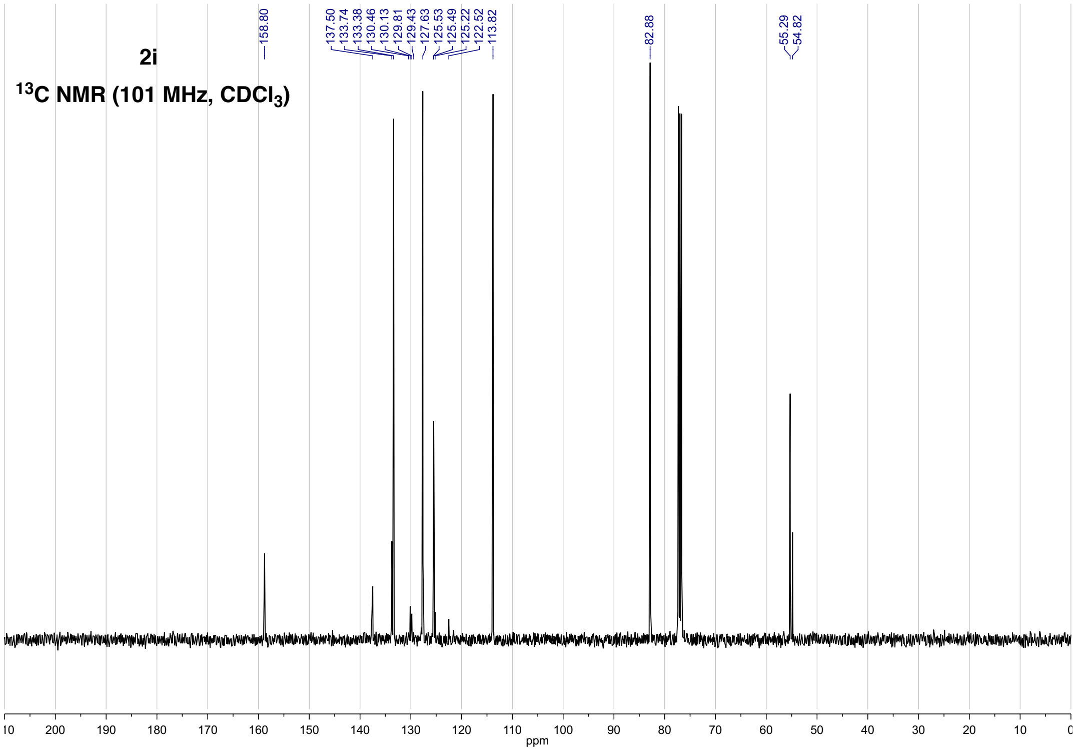

2i
^{13}C NMR (101 MHz, CDCl$_3$)
158.80
137.50 133.74 133.38 130.46 130.13 129.81 129.43 127.63 125.53 125.49 125.22 122.52 113.82
82.88
55.29 54.82
ppm

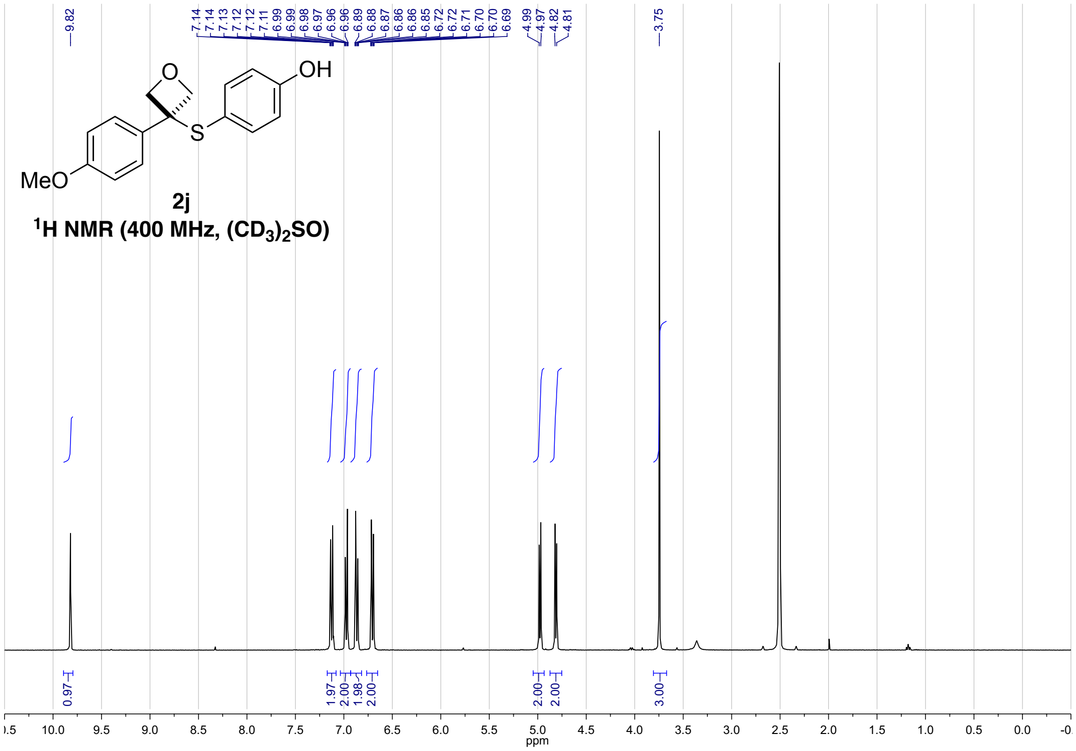
OH
O
S
MeO
2j
1H NMR (400 MHz, (CD3)2SO)
ppm

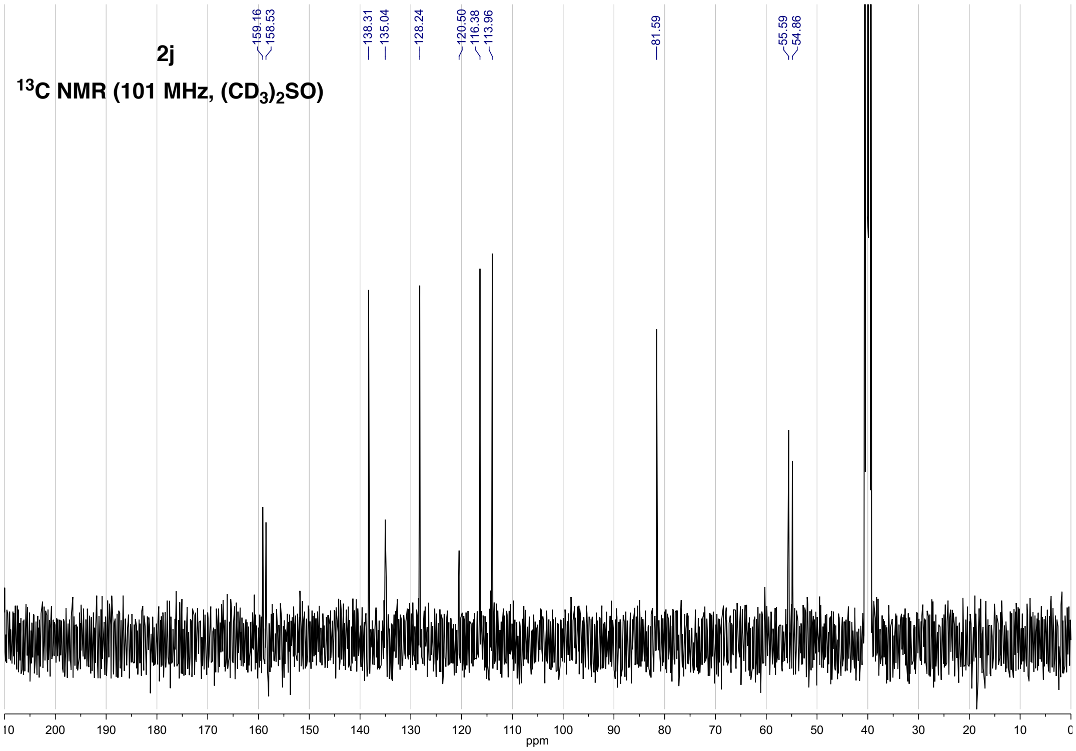
2j
13C NMR (101 MHz, (CD3)2SO)
ppm

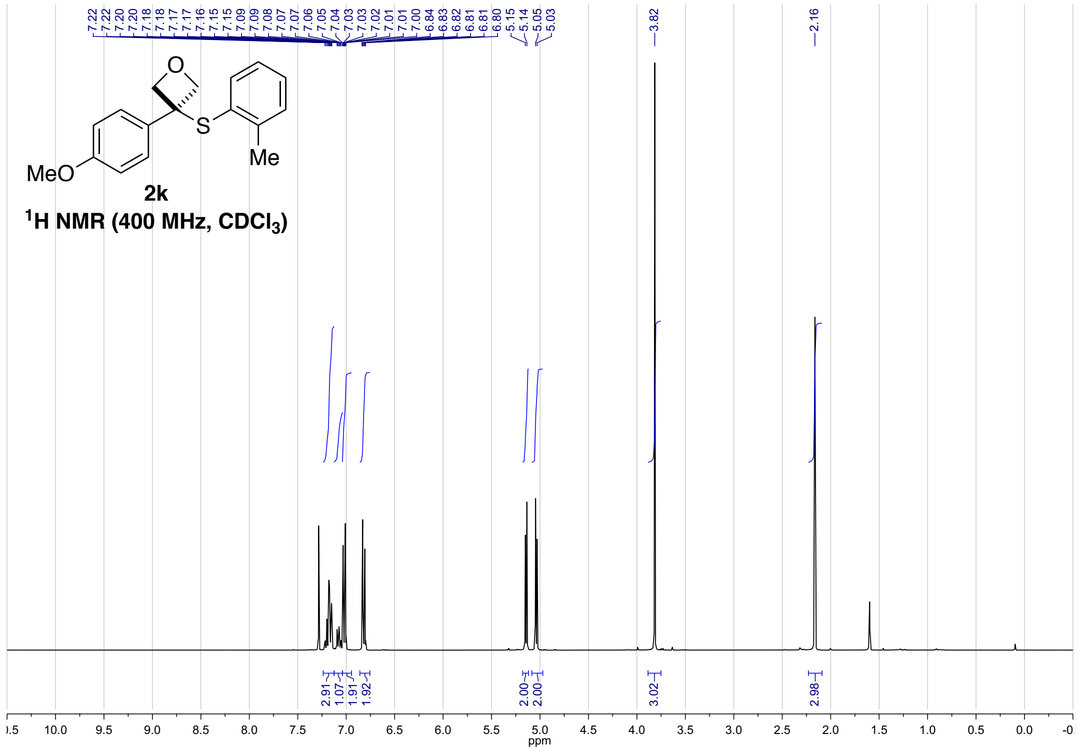
MeO
S
O
Me
2k
^{1}H NMR (400 MHz, CDCl$_3$)
ppm

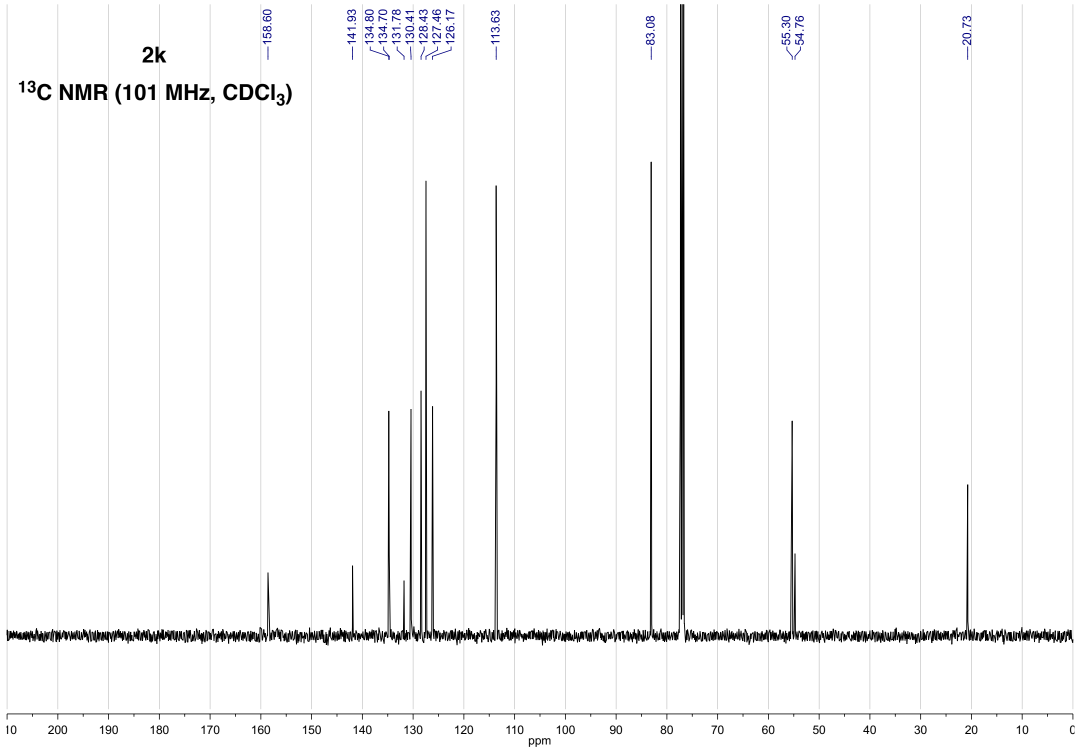
2k
^{13}C NMR (101 MHz, CDCl$_3$)
158.60
141.93
134.80
134.70
131.78
130.41
128.43
127.46
126.17
113.63
83.08
55.30
54.76
20.73
ppm

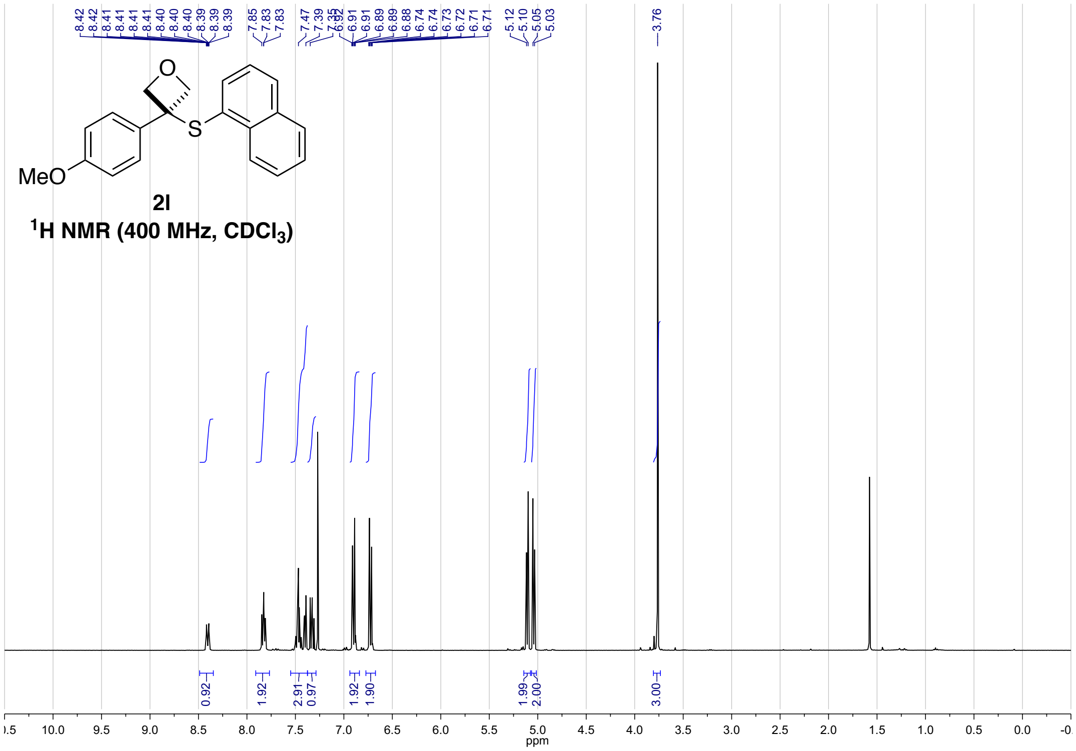
MeO
S
O
2l
1H NMR (400 MHz, CDCl3)
ppm

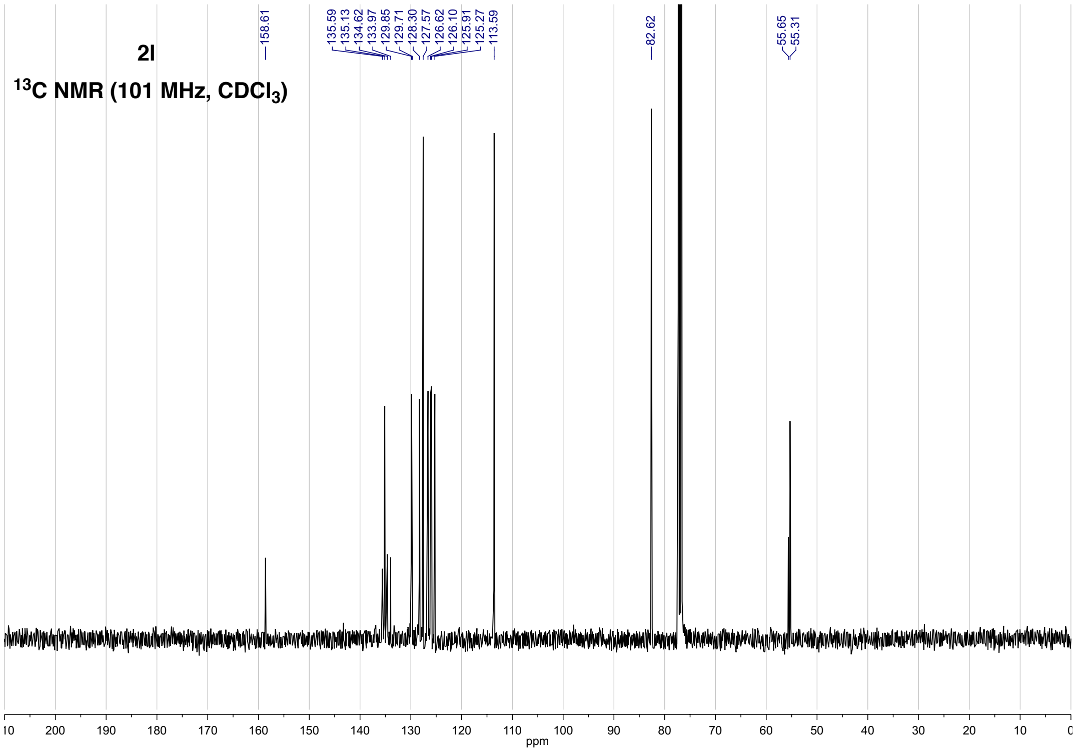
2l
13C NMR (101 MHz, CDCl3)
ppm

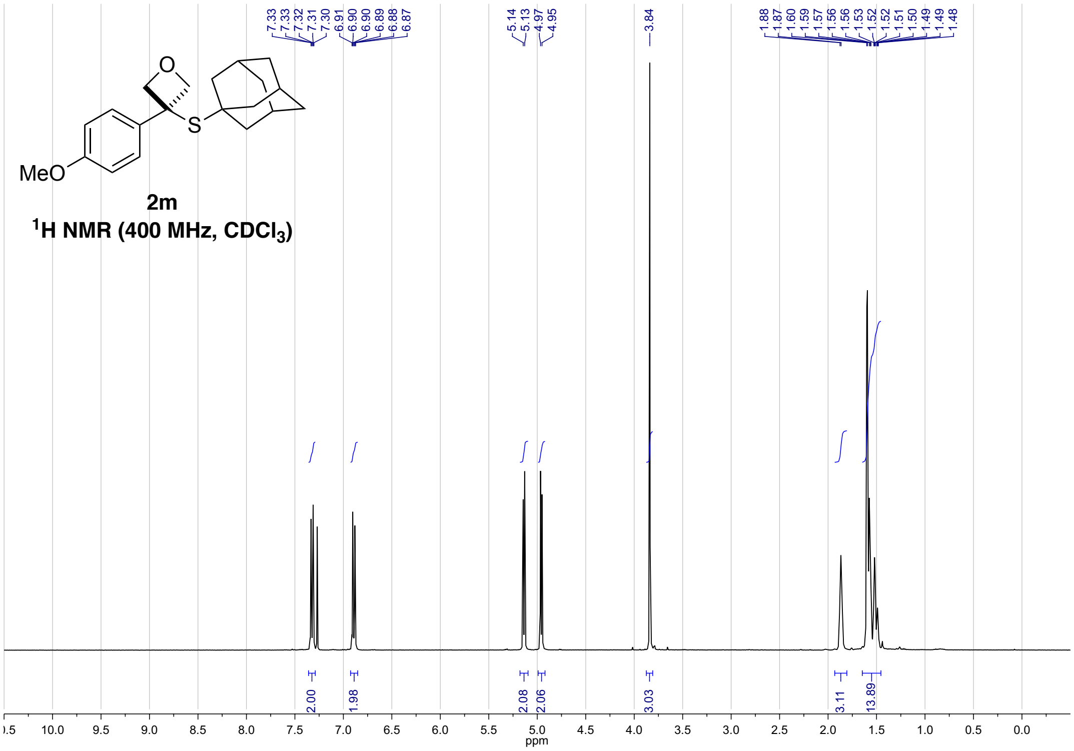
MeO
O
S
2m
1H NMR (400 MHz, CDCl3)
7.33
7.33
7.32
7.31
7.30
6.91
6.90
6.90
6.89
6.88
6.87
5.14
5.13
4.97
4.95
3.84
1.88
1.87
1.60
1.59
1.57
1.56
1.56
1.53
1.52
1.52
1.51
1.50
1.49
1.49
1.48
2.00
1.98
2.08
2.06
3.03
3.11
13.89
ppm

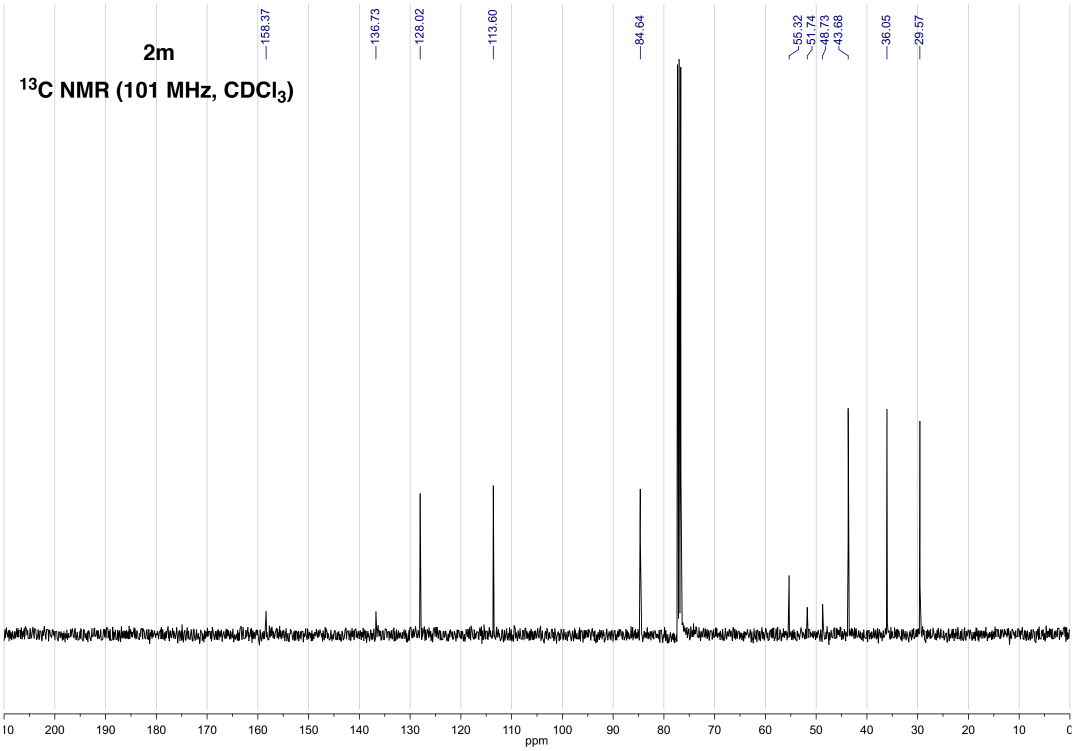
2m
13C NMR (101 MHz, CDCl3)
158.37
136.73
128.02
113.60
84.64
55.32
51.74
48.73
43.68
36.05
29.57
ppm

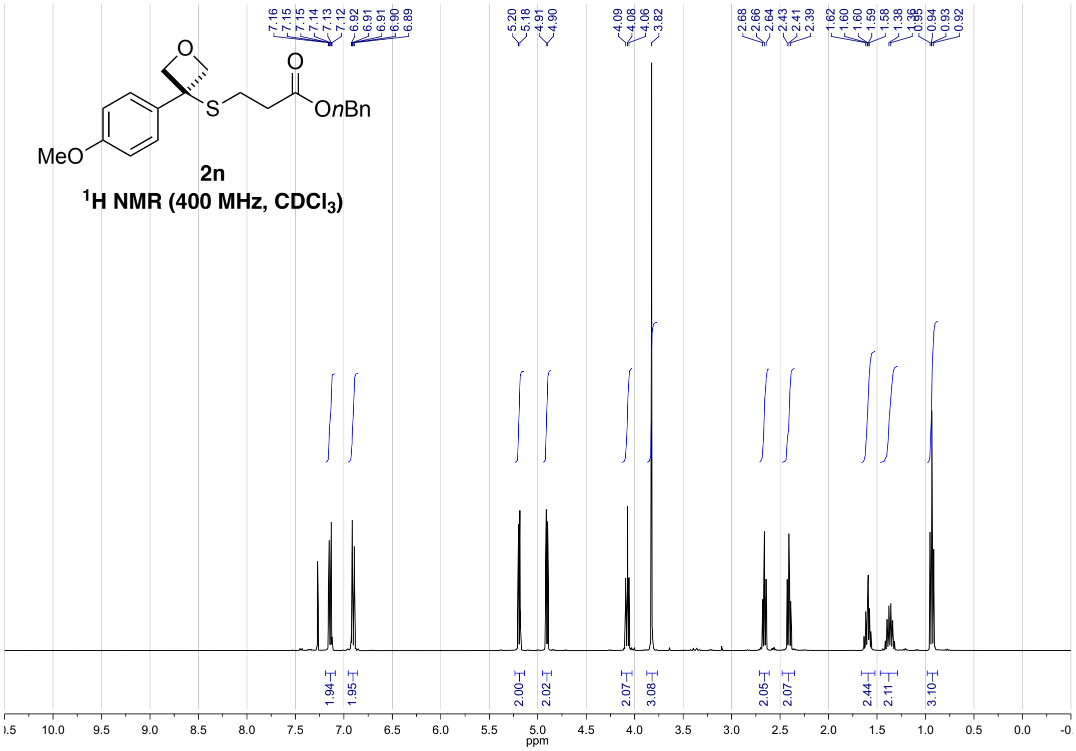
MeO
OnBn
2n
^{1}H NMR (400 MHz, $CDCl_3$)
ppm

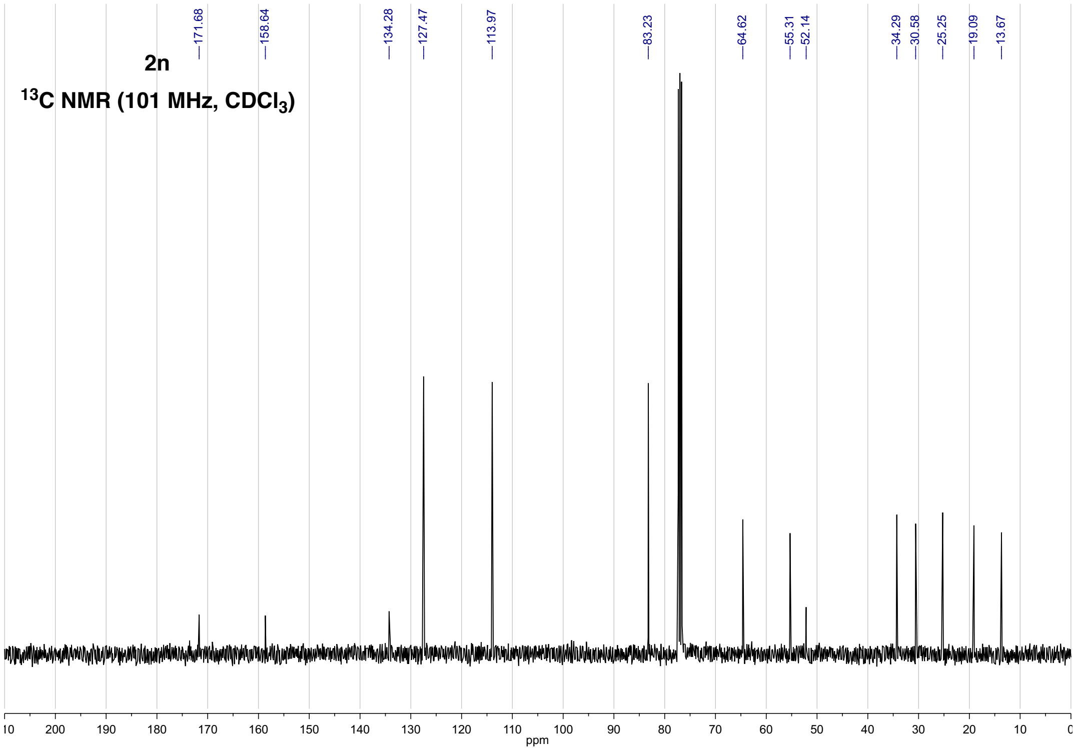
2n
^{13}C NMR (101 MHz, $CDCl_3$)
ppm

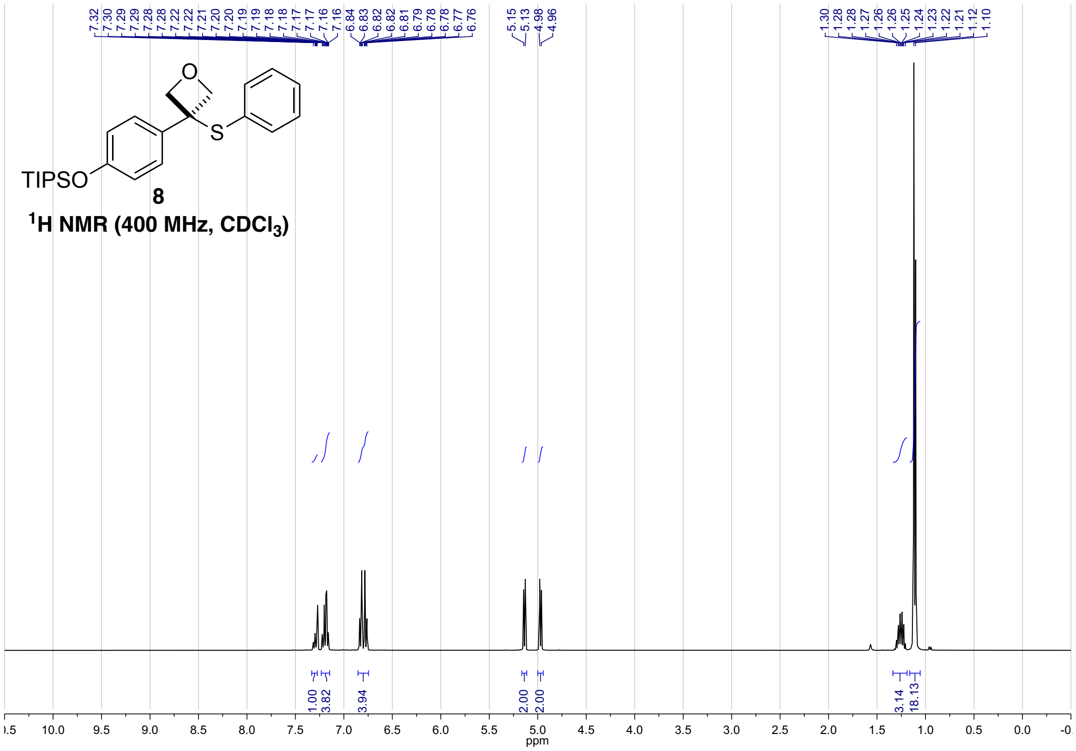
TIPSO
S
O
8
1H NMR (400 MHz, CDCl3)
ppm

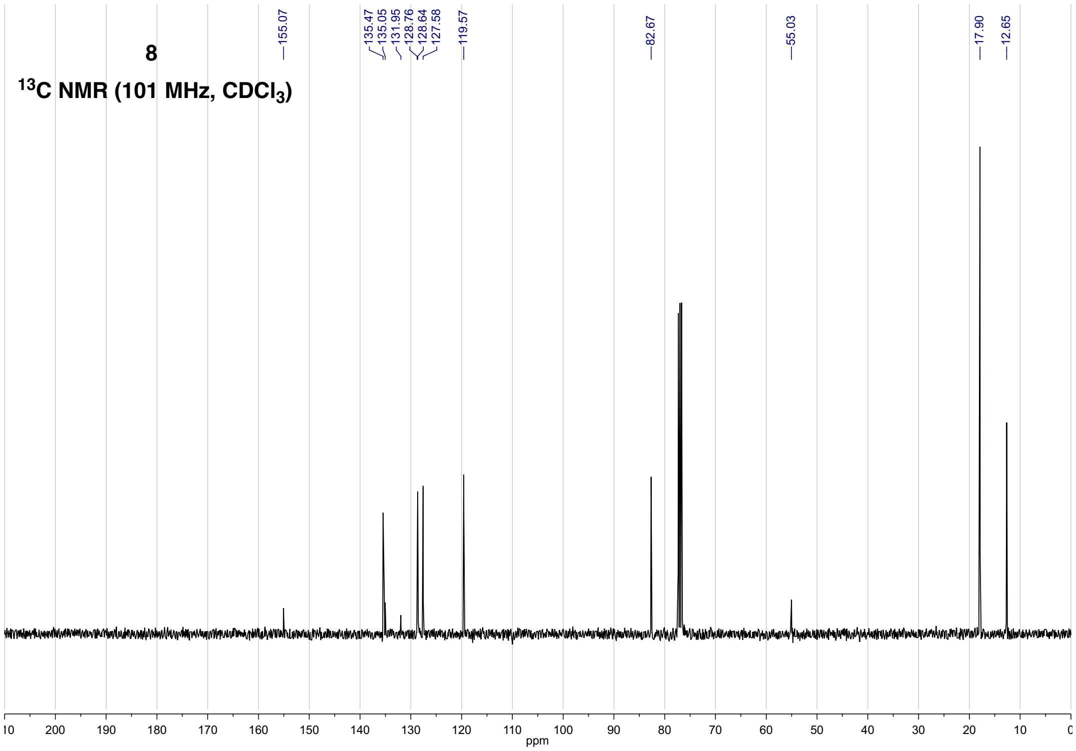
8
13C NMR (101 MHz, CDCl3)
155.07
135.47
135.05
131.95
128.76
128.64
127.58
119.57
82.67
55.03
17.90
12.65
ppm

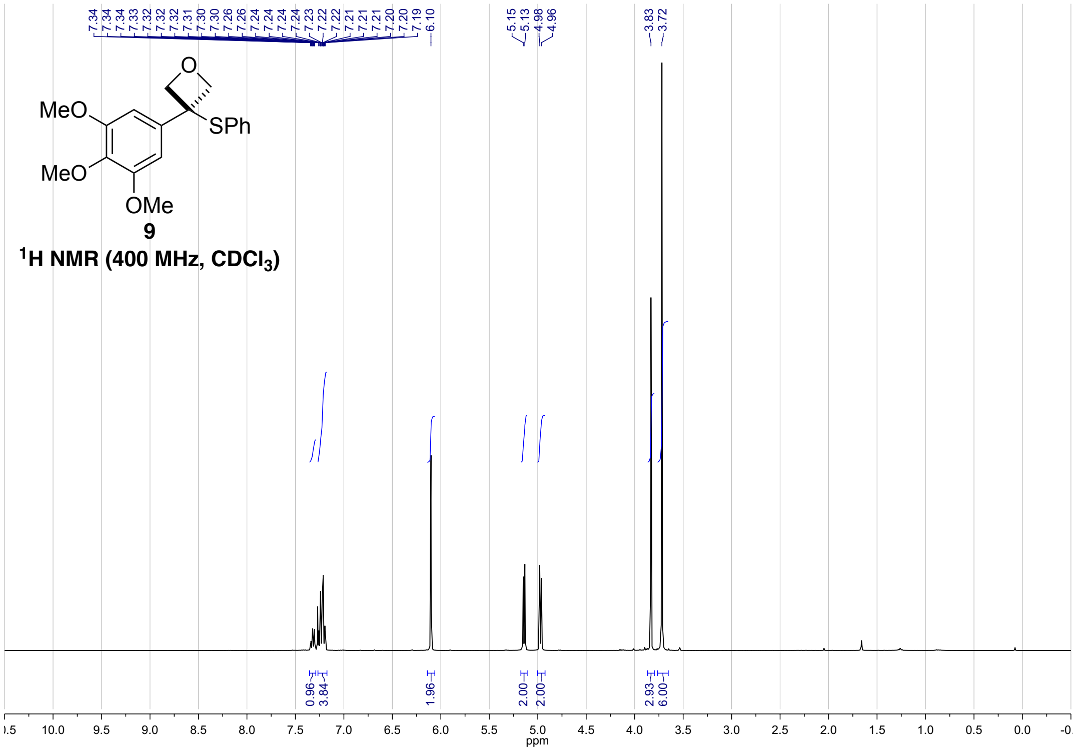
MeO
MeO
OMe
SPh
O
9
^{1}H NMR (400 MHz, $CDCl_3$)
ppm

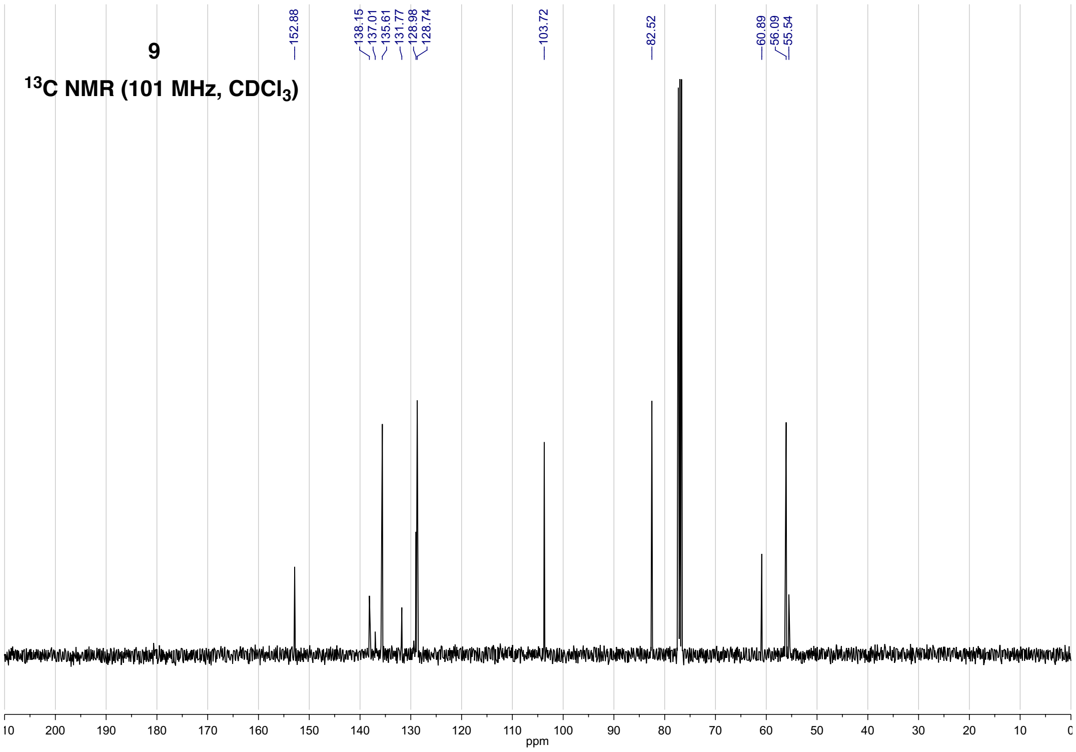
9
^{13}C NMR (101 MHz, $CDCl_3$)
ppm

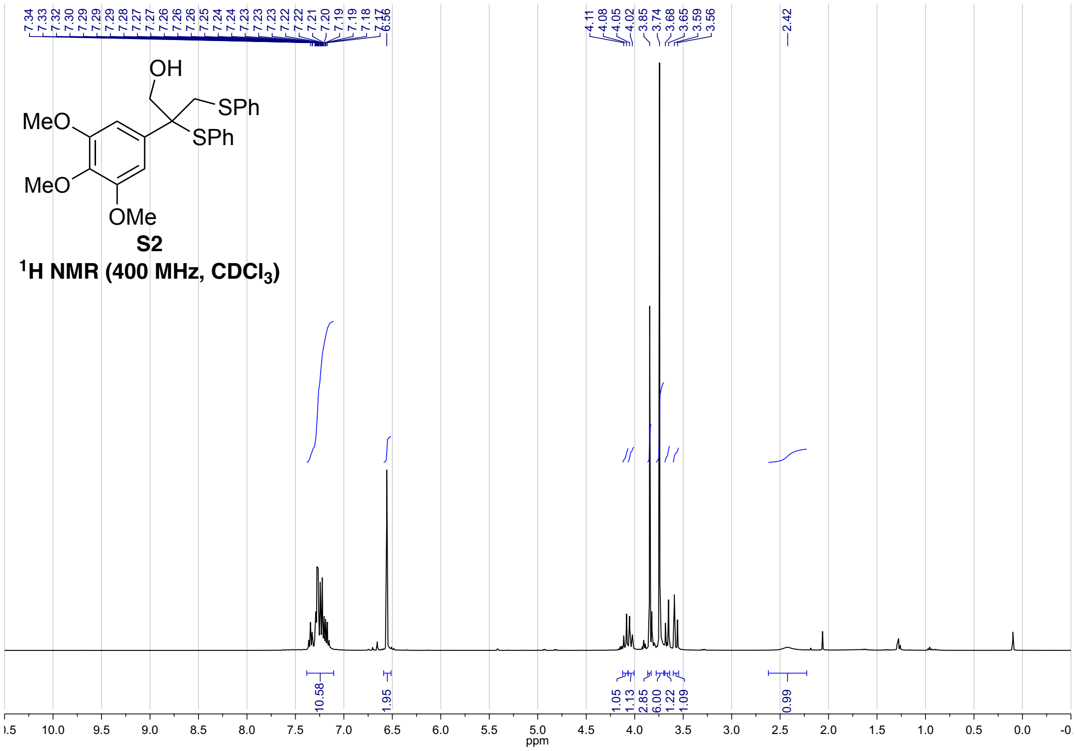
OH
SPh
SPh
MeO
MeO
OMe
S2
1H NMR (400 MHz, CDCl3)
7.34 7.33 7.32 7.30 7.29 7.29 7.28 7.27 7.27 7.26 7.26 7.25 7.24 7.24 7.23 7.23 7.23 7.22 7.22 7.21 7.20 7.19 7.19 7.18 7.17 6.56
4.11 4.08 4.05 4.02 3.85 3.74 3.68 3.65 3.59 3.56
2.42
10.58 1.95 1.05 1.13 2.85 6.00 1.22 1.09 0.99
ppm

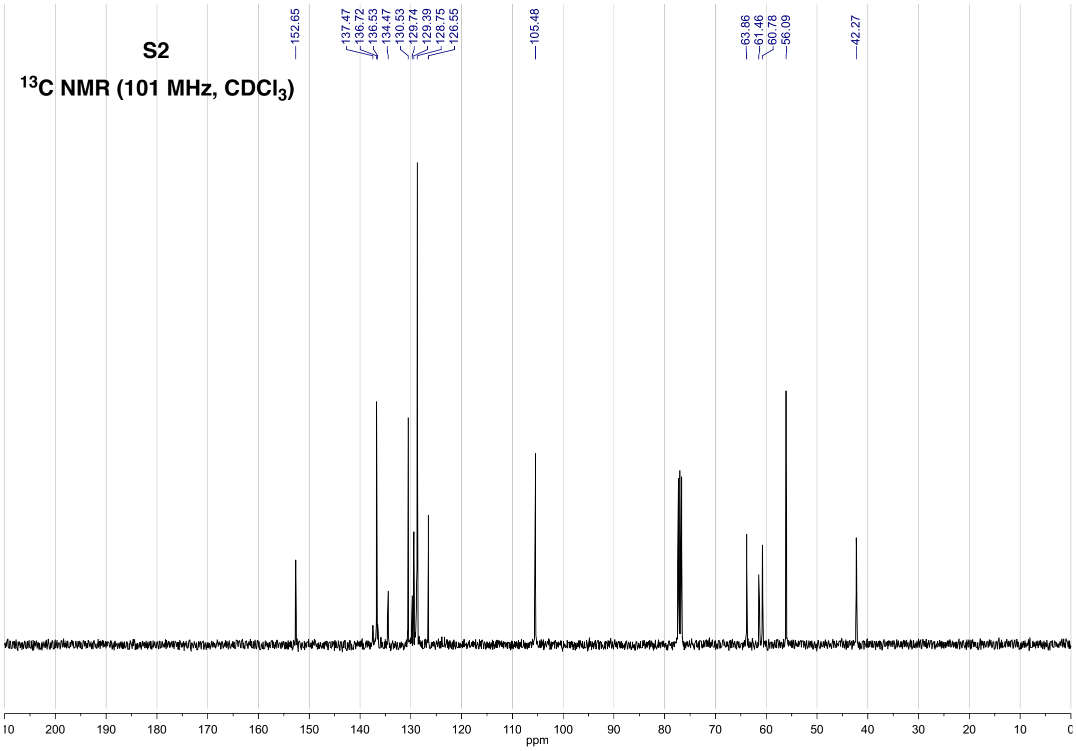
S2
13C NMR (101 MHz, CDCl3)
152.65
137.47 136.72 136.53 134.47 130.53 129.74 129.39 128.75 126.55
105.48
63.86 61.46 60.78 56.09
42.27
ppm

10

^{1}H NMR (400 MHz, $CDCl_3$)

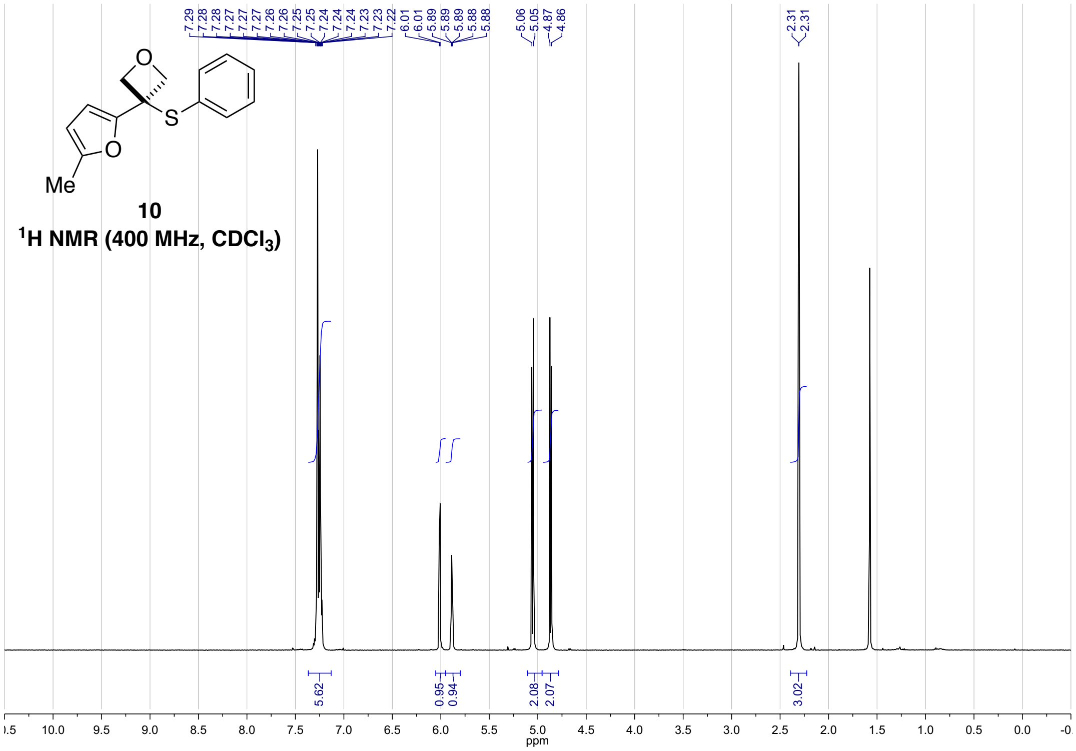

10

^{13}C NMR (101 MHz, $CDCl_3$)

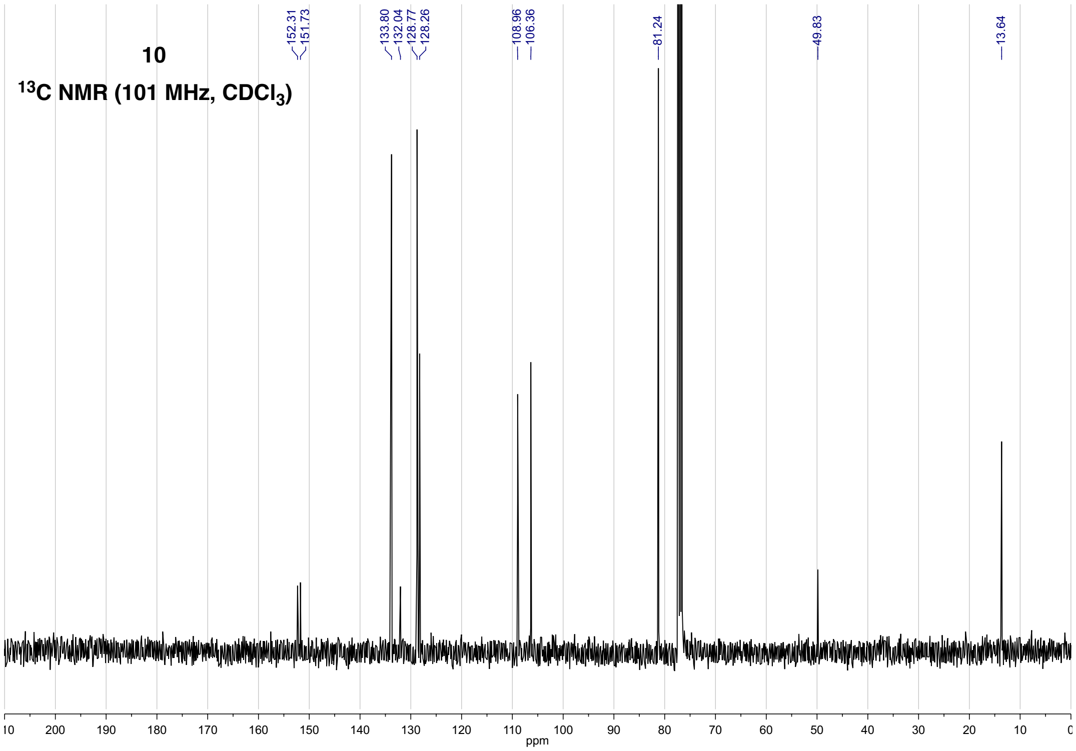

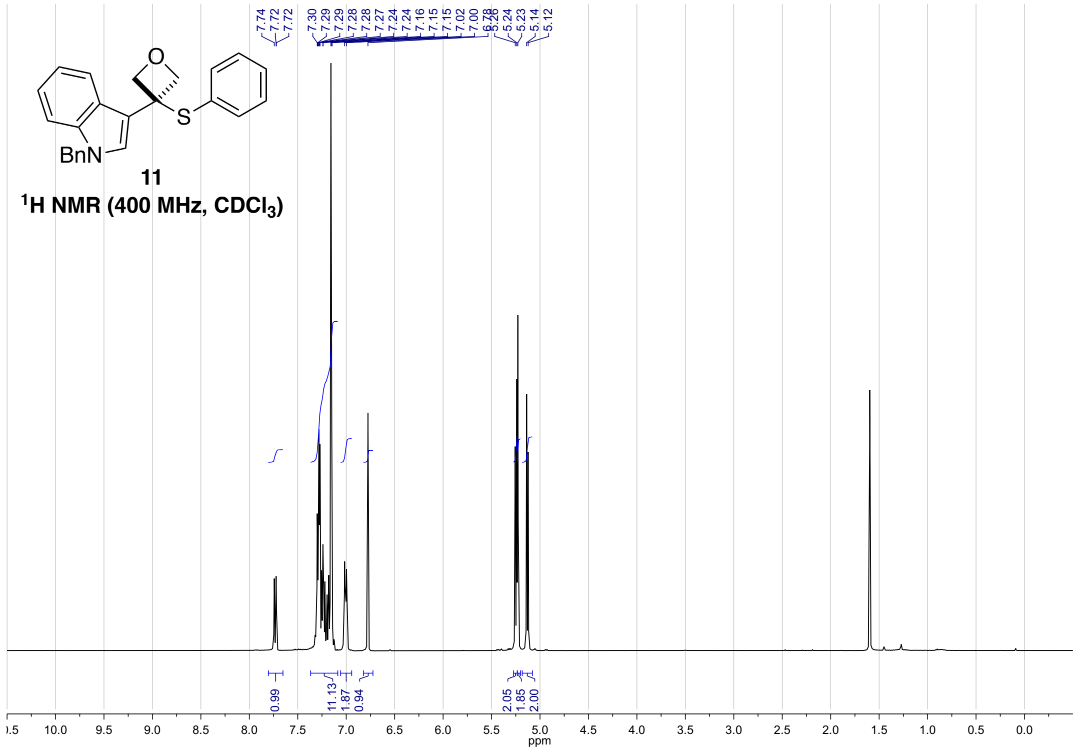
BnN
O
S
11
^{1}H NMR (400 MHz, $CDCl_3$)
7.74 7.72 7.72
7.30 7.29 7.29 7.28 7.28 7.27 7.24 7.24 7.16 7.15 7.15 7.02 7.00 6.78 5.26 5.24 5.23 5.14 5.12
0.99 11.13 1.87 0.94 2.05 1.85 2.00
ppm

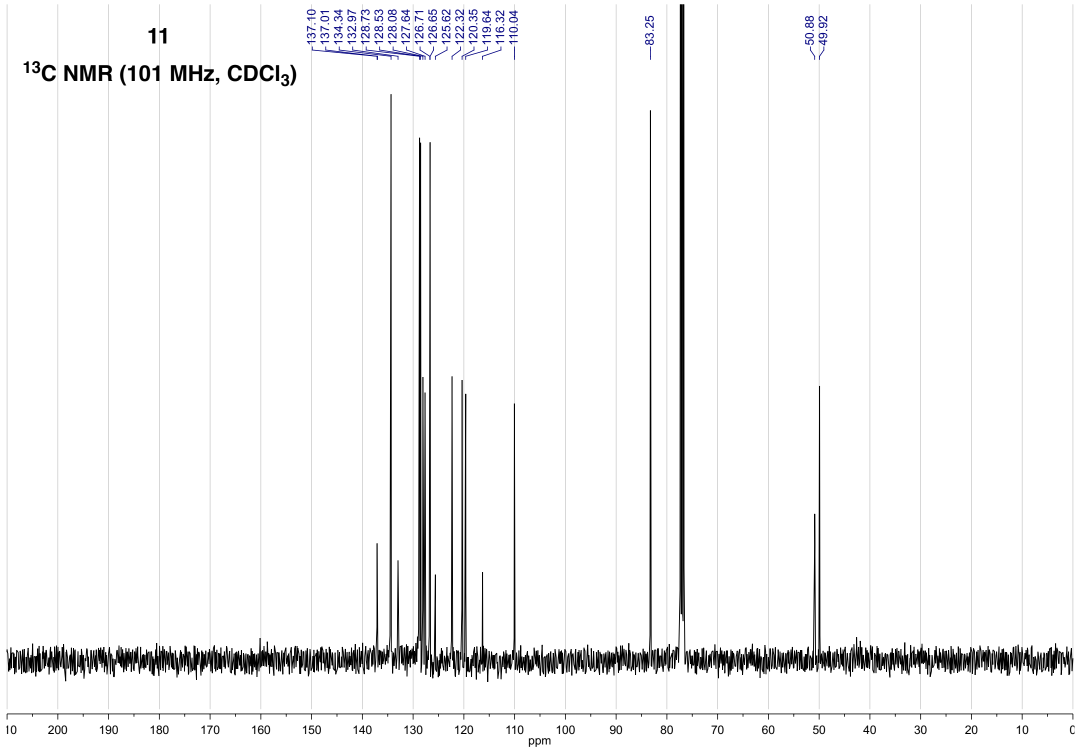
11
^{13}C NMR (101 MHz, $CDCl_3$)
137.10 137.01 134.34 132.97 128.73 128.53 128.08 127.64 126.71 126.65 125.62 122.32 120.35 119.64 116.32 110.04
83.25
50.88 49.92
ppm

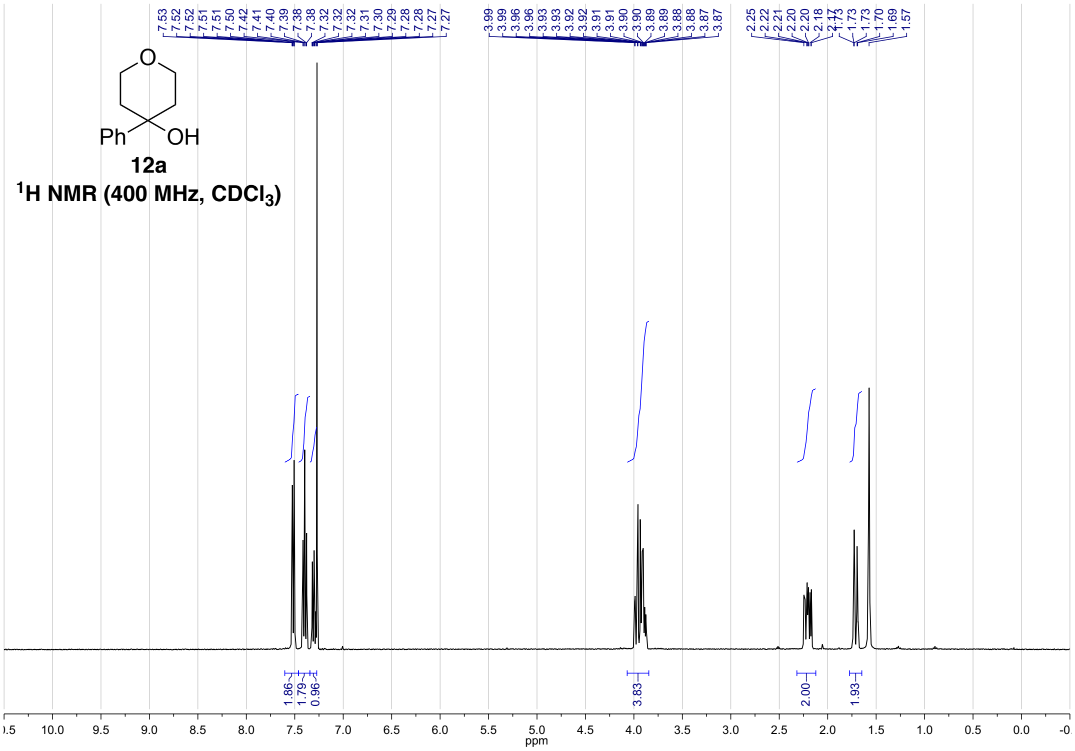
Ph
OH
O
12a
1H NMR (400 MHz, CDCl3)
ppm

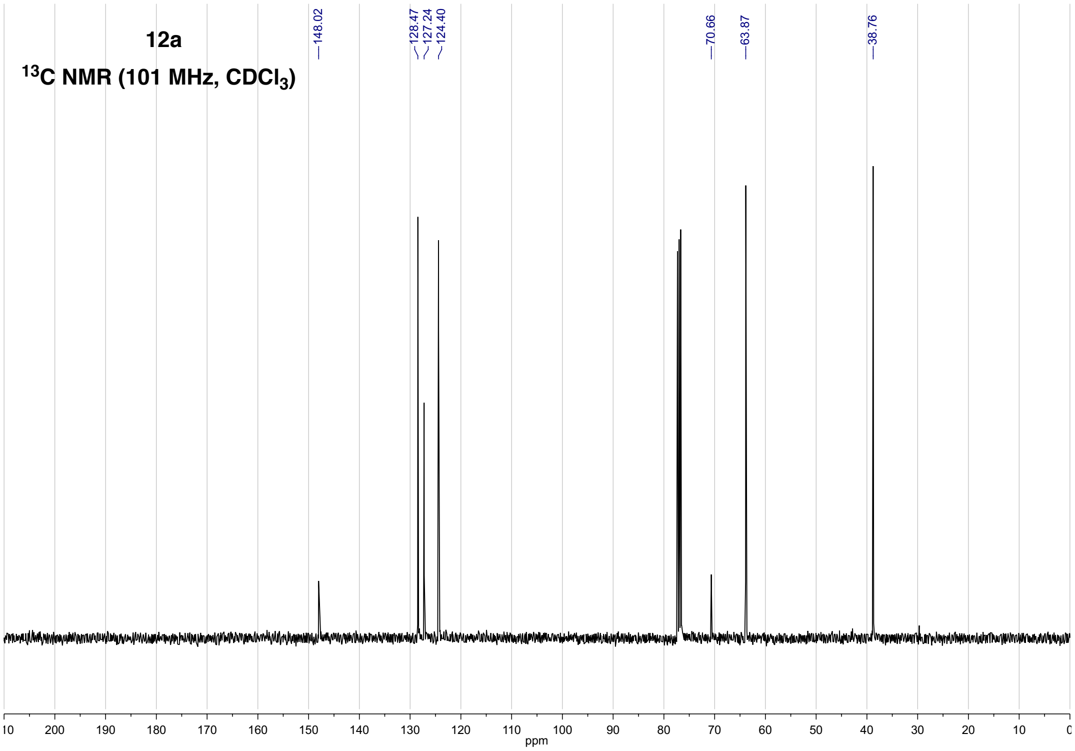
12a
13C NMR (101 MHz, CDCl3)
148.02
128.47
127.24
124.40
70.66
63.87
38.76
ppm

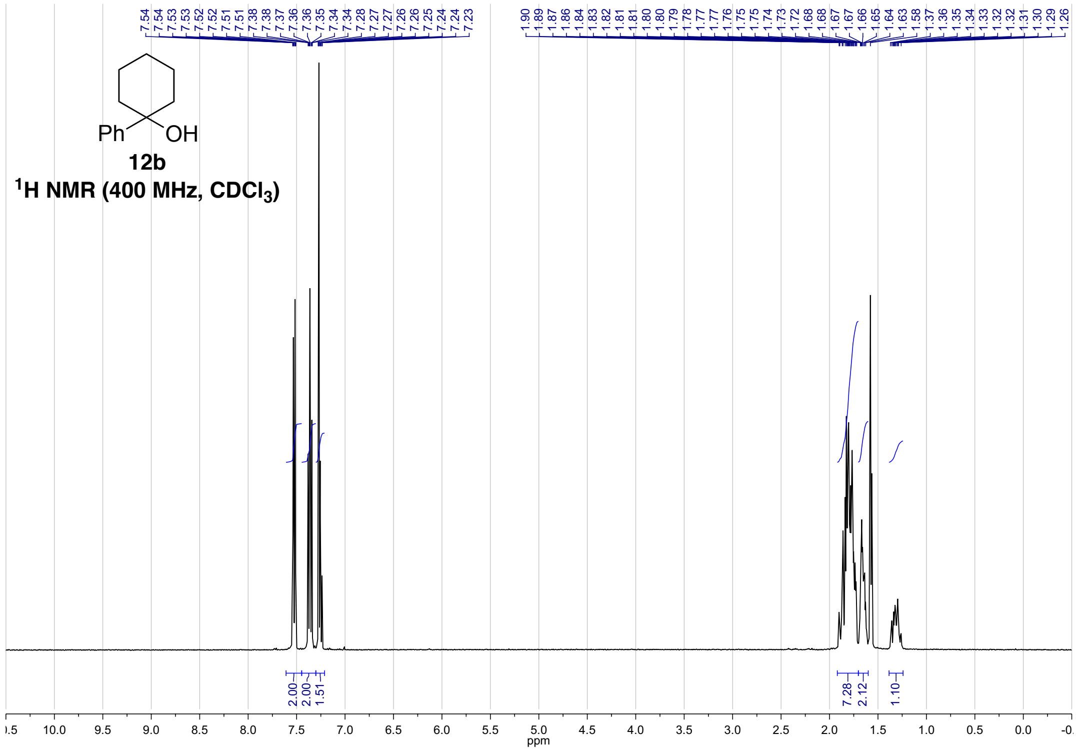
Ph OH
12b
1H NMR (400 MHz, CDCl3)
ppm

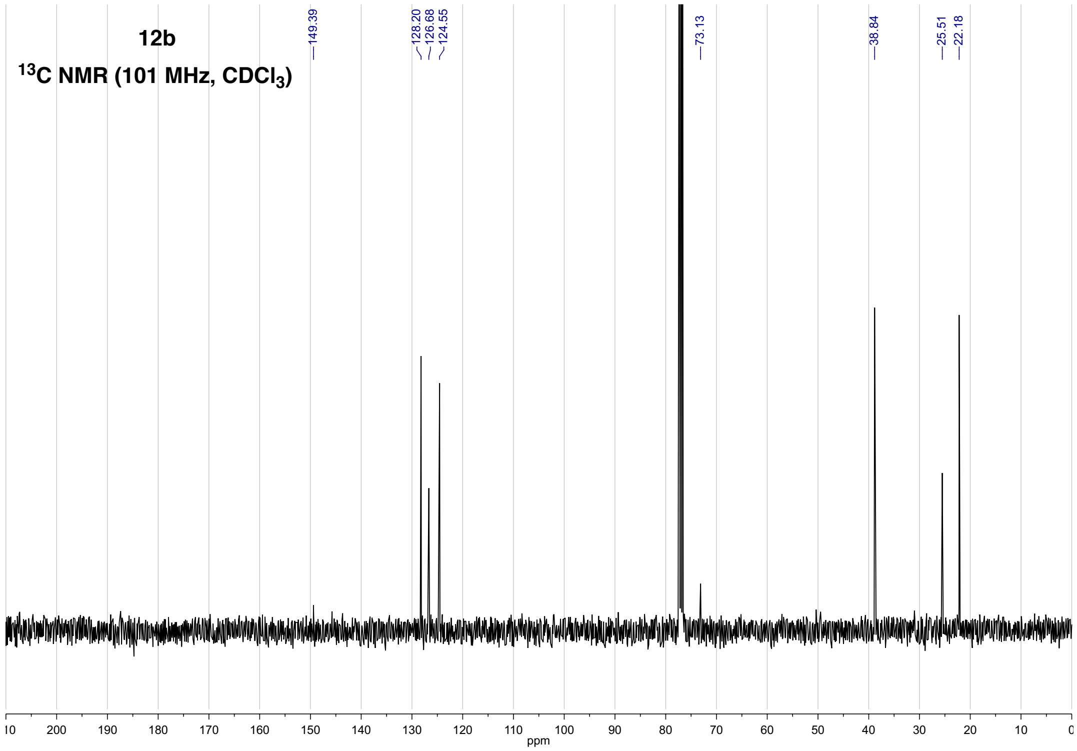
12b
13C NMR (101 MHz, CDCl3)
149.39
128.20
126.68
124.55
73.13
38.84
25.51
22.18
ppm

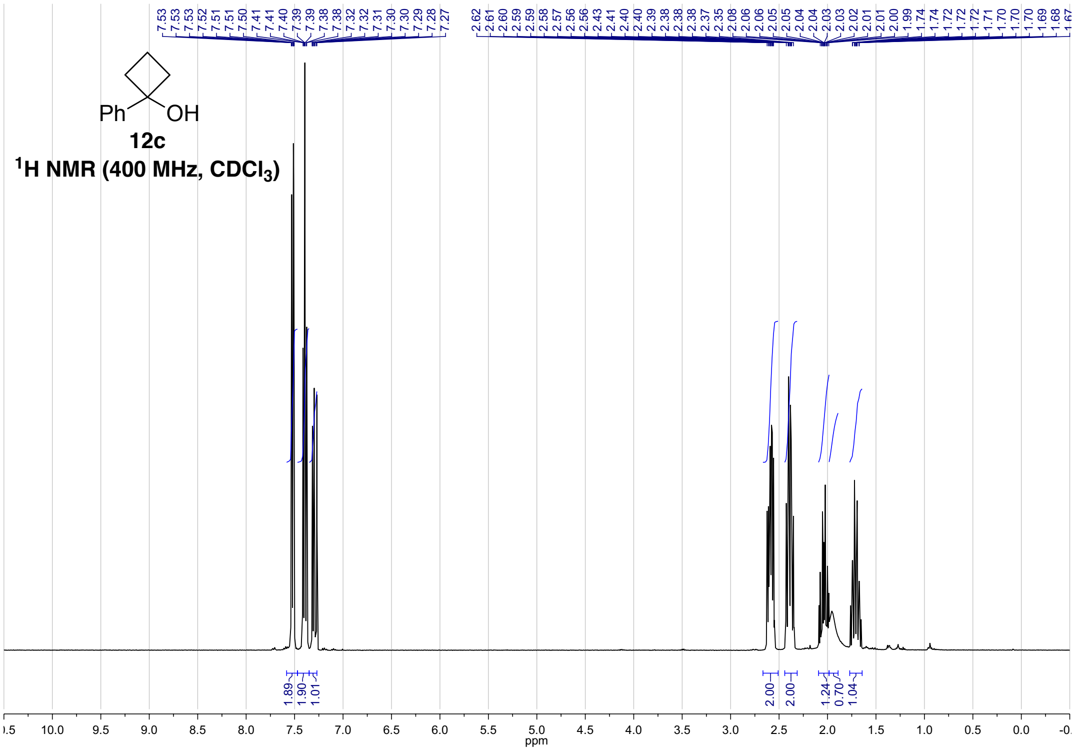
Ph OH
12c
1H NMR (400 MHz, CDCl3)
7.53 7.53 7.52 7.51 7.51 7.50 7.41 7.40 7.39 7.39 7.38 7.38 7.32 7.32 7.31 7.30 7.30 7.29 7.28 7.27
2.62 2.61 2.60 2.59 2.59 2.58 2.57 2.56 2.56 2.43 2.41 2.40 2.40 2.39 2.38 2.38 2.38 2.37 2.35 2.08 2.06 2.06 2.05 2.05 2.04 2.04 2.03 2.03 2.02 2.01 2.00 1.99 1.74 1.74 1.72 1.72 1.72 1.71 1.70 1.70 1.69 1.68 1.67
1.89 1.90 1.01 2.00 2.00 1.24 0.70 1.04
ppm

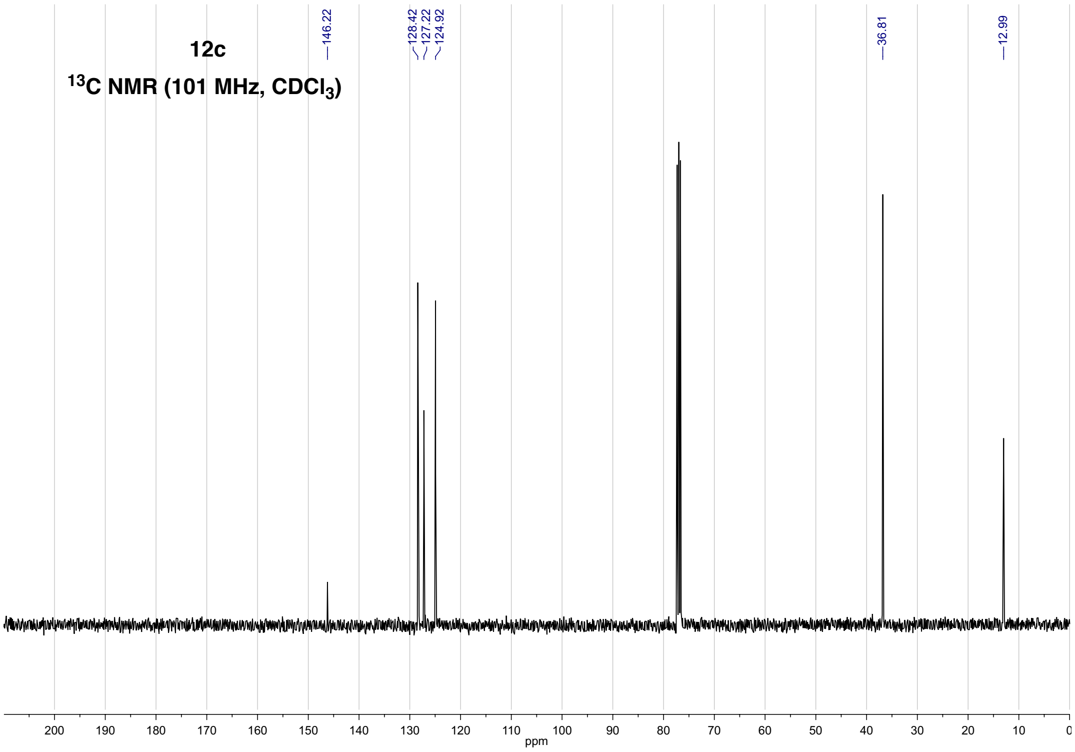
12c
13C NMR (101 MHz, CDCl3)
146.22
128.42
127.22
124.92
36.81
12.99
ppm

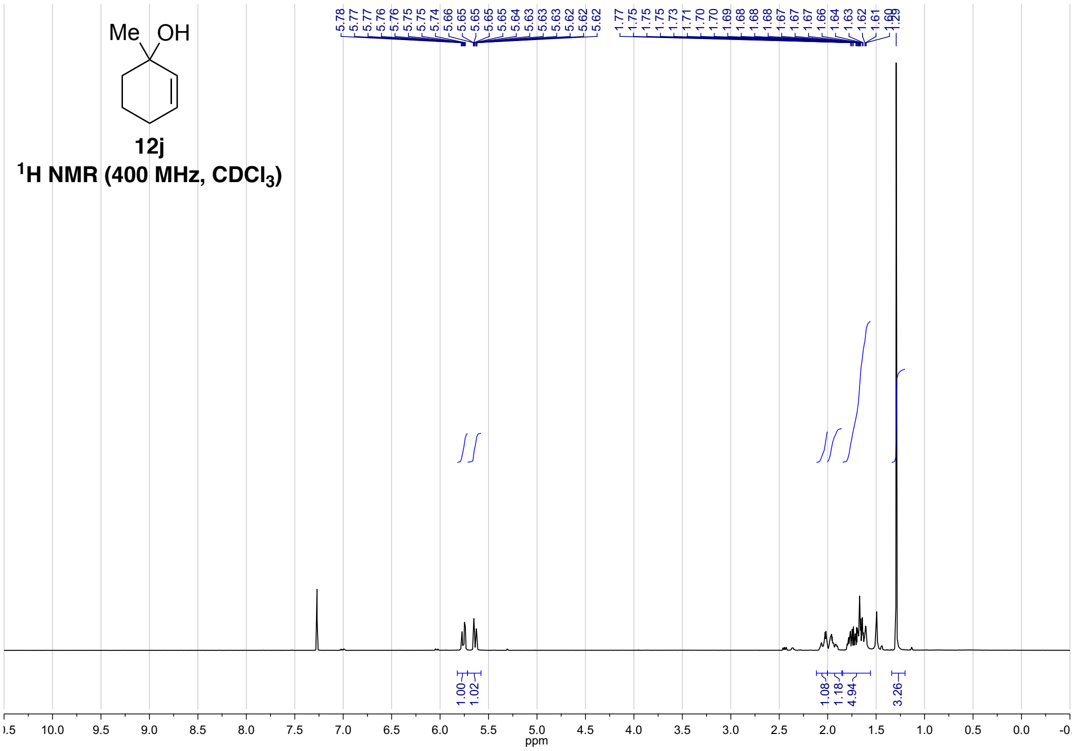
Me
OH
12j
¹H NMR (400 MHz, CDCl₃)
ppm

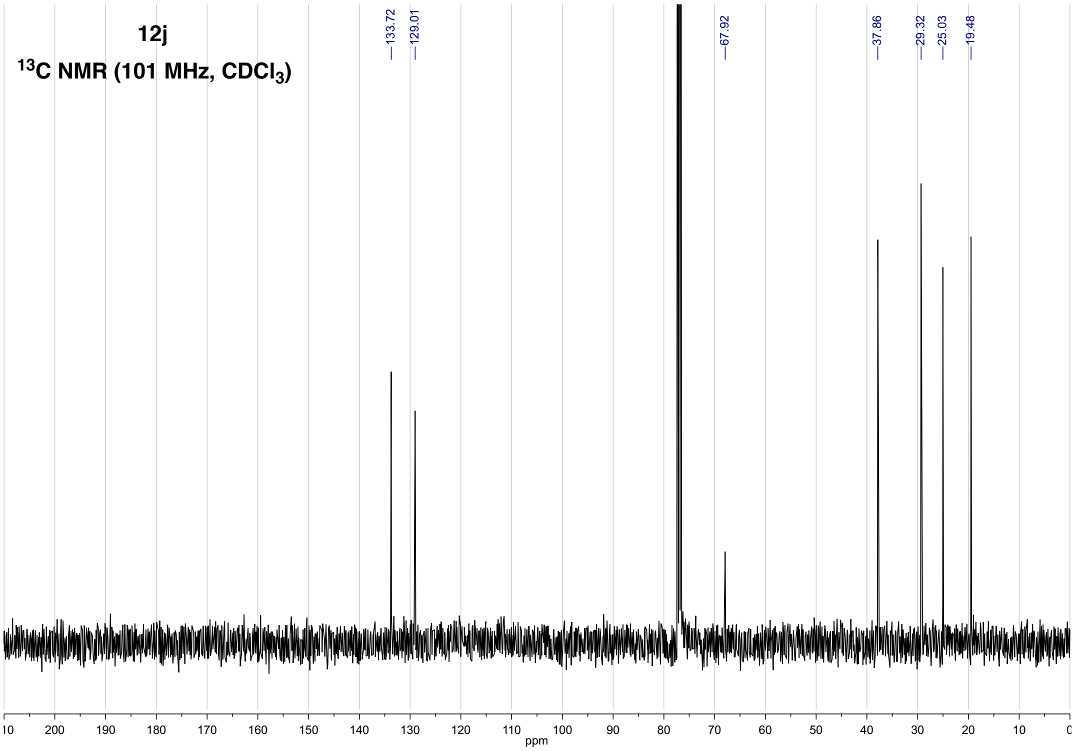
12j
¹³C NMR (101 MHz, CDCl₃)
133.72
129.01
67.92
37.86
29.32
25.03
19.48
ppm

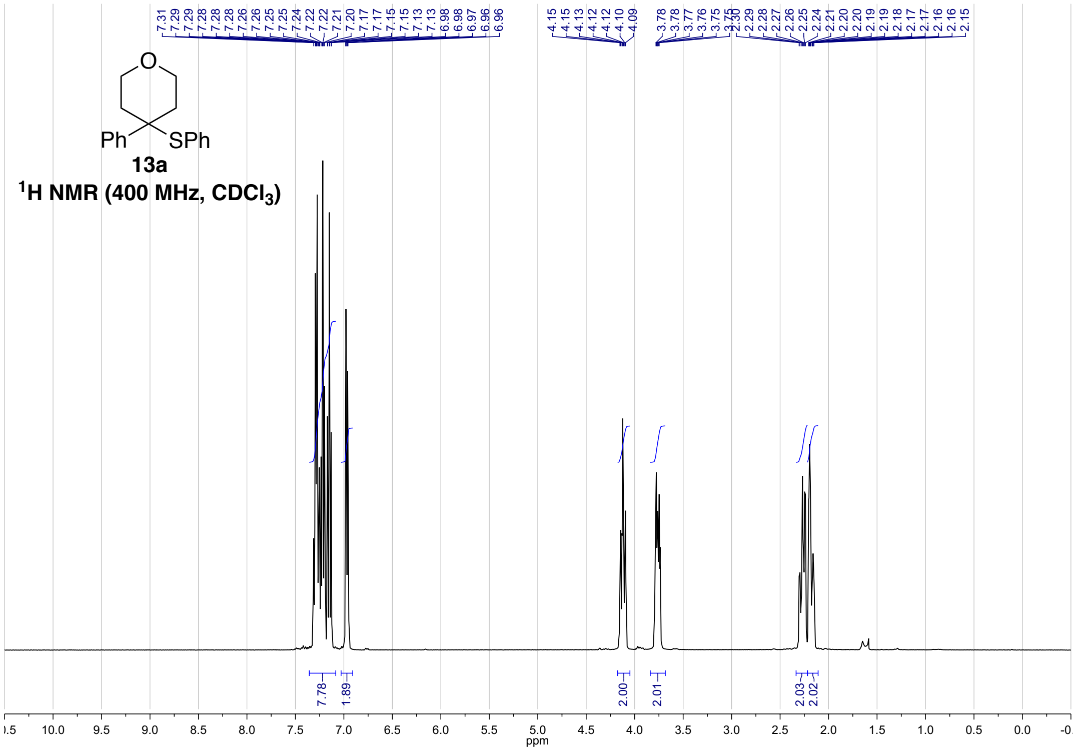

13a

^{1}H NMR (400 MHz, $CDCl_3$)

13a

^{13}C NMR (101 MHz, $CDCl_3$)

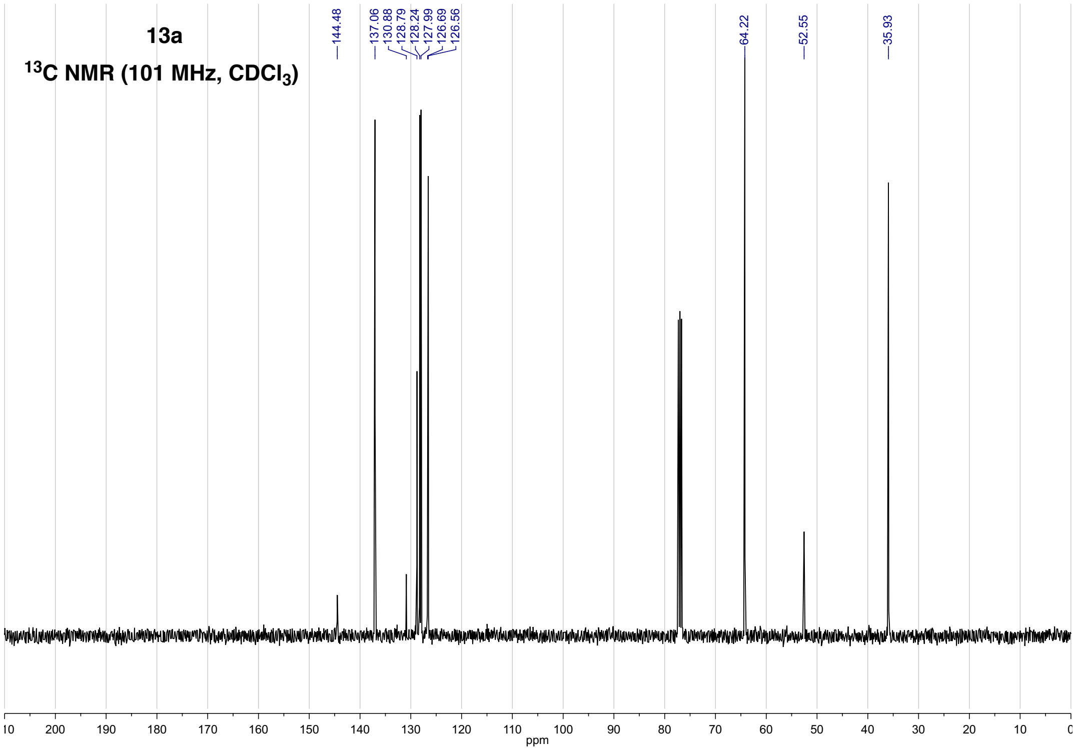

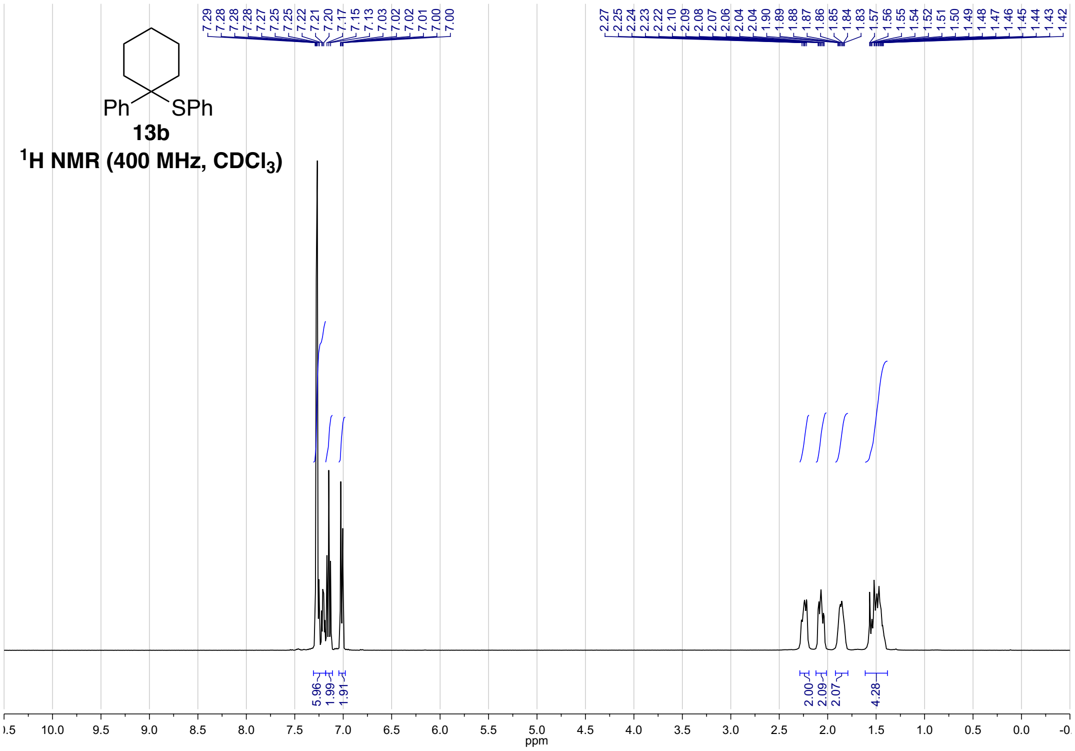
Ph SPh
13b
1H NMR (400 MHz, CDCl3)
7.29 7.28 7.28 7.28 7.27 7.25 7.25 7.22 7.21 7.20 7.17 7.15 7.13 7.03 7.02 7.01 7.00 7.00
2.27 2.25 2.24 2.23 2.22 2.10 2.09 2.08 2.07 2.06 2.04 2.04 1.90 1.89 1.88 1.87 1.86 1.85 1.84 1.83 1.57 1.56 1.55 1.54 1.52 1.51 1.50 1.49 1.48 1.47 1.46 1.45 1.44 1.43 1.42
5.96 1.99 1.91
2.00 2.09 2.07
4.28
ppm

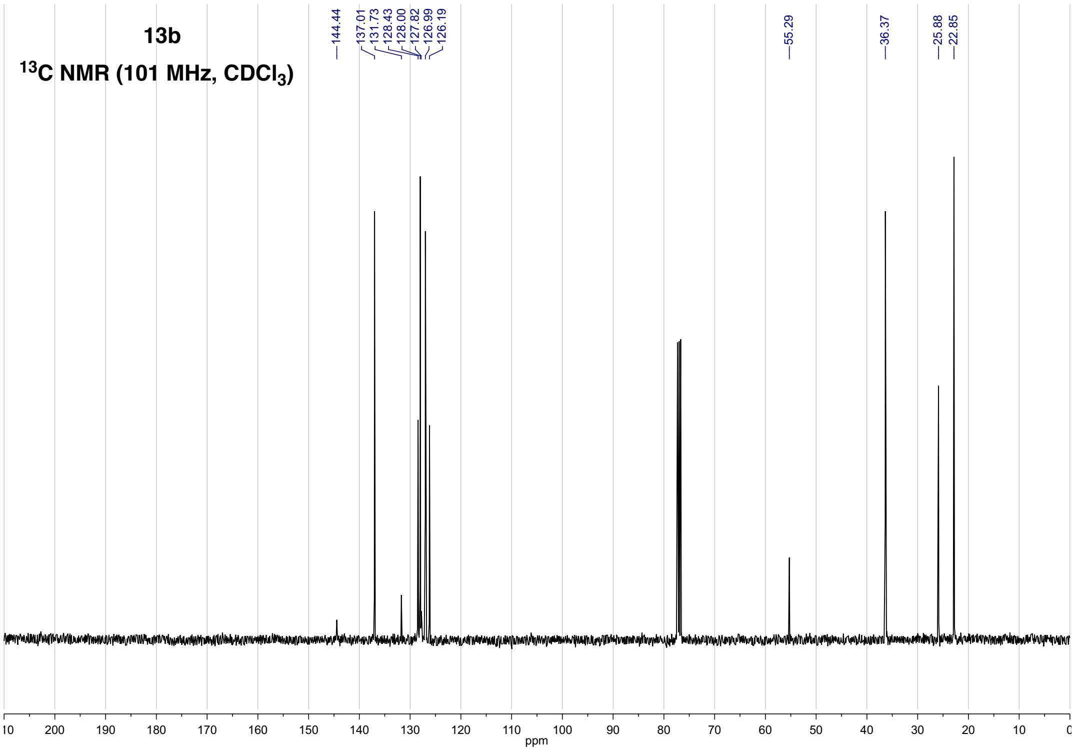
13b
13C NMR (101 MHz, CDCl3)
144.44
137.01 131.73 128.43 128.00 127.82 126.99 126.19
55.29
36.37
25.88 22.85
ppm

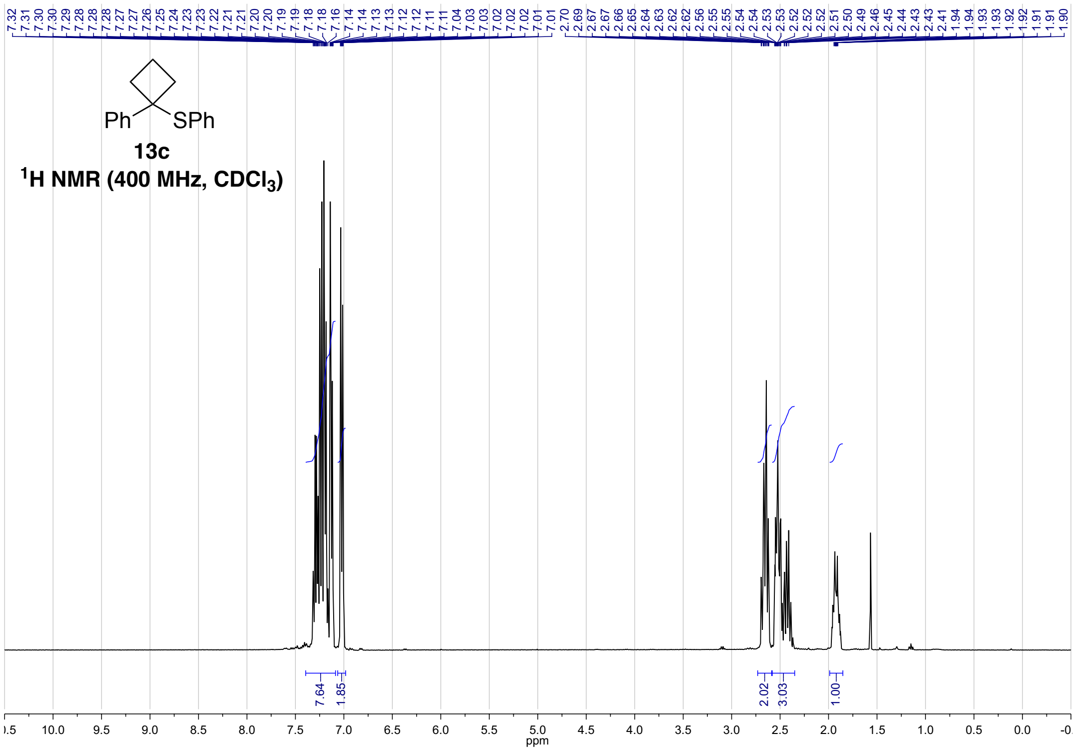
13c
1H NMR (400 MHz, CDCl3)
7.64
1.85
2.02
3.03
1.00
ppm

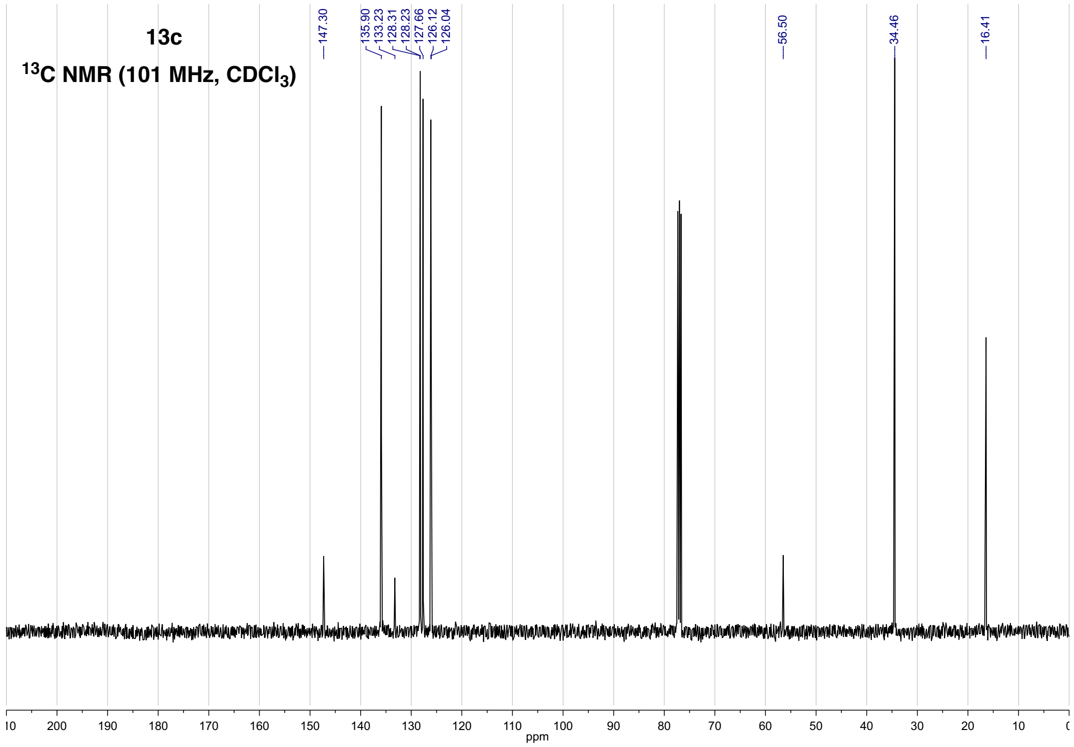
13c
13C NMR (101 MHz, CDCl3)
147.30
135.90
133.23
128.31
128.23
127.66
126.12
126.04
56.50
34.46
16.41
ppm

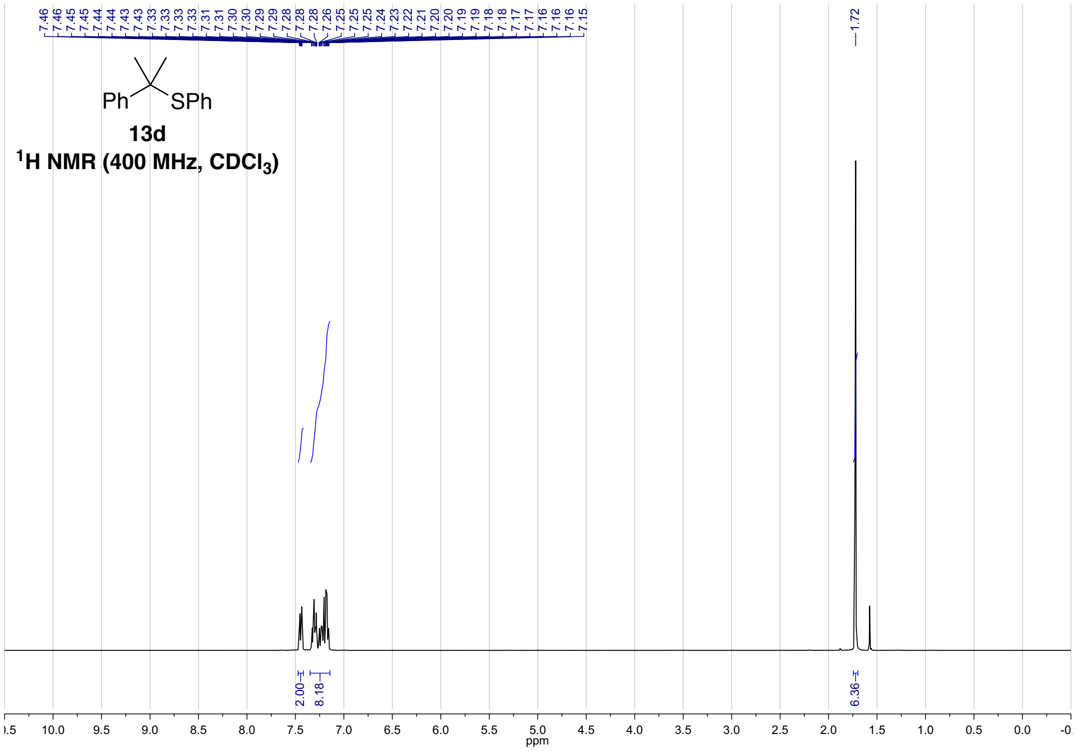
Ph SPh
13d
^{1}H NMR (400 MHz, $CDCl_3$)
7.46 7.46 7.45 7.45 7.44 7.44 7.43 7.43 7.33 7.33 7.33 7.33 7.31 7.31 7.30 7.30 7.29 7.29 7.28 7.28 7.28 7.26 7.25 7.25 7.25 7.25 7.24 7.23 7.22 7.21 7.20 7.20 7.19 7.19 7.18 7.18 7.17 7.17 7.16 7.16 7.16 7.15
1.72
2.00
8.18
6.36
ppm

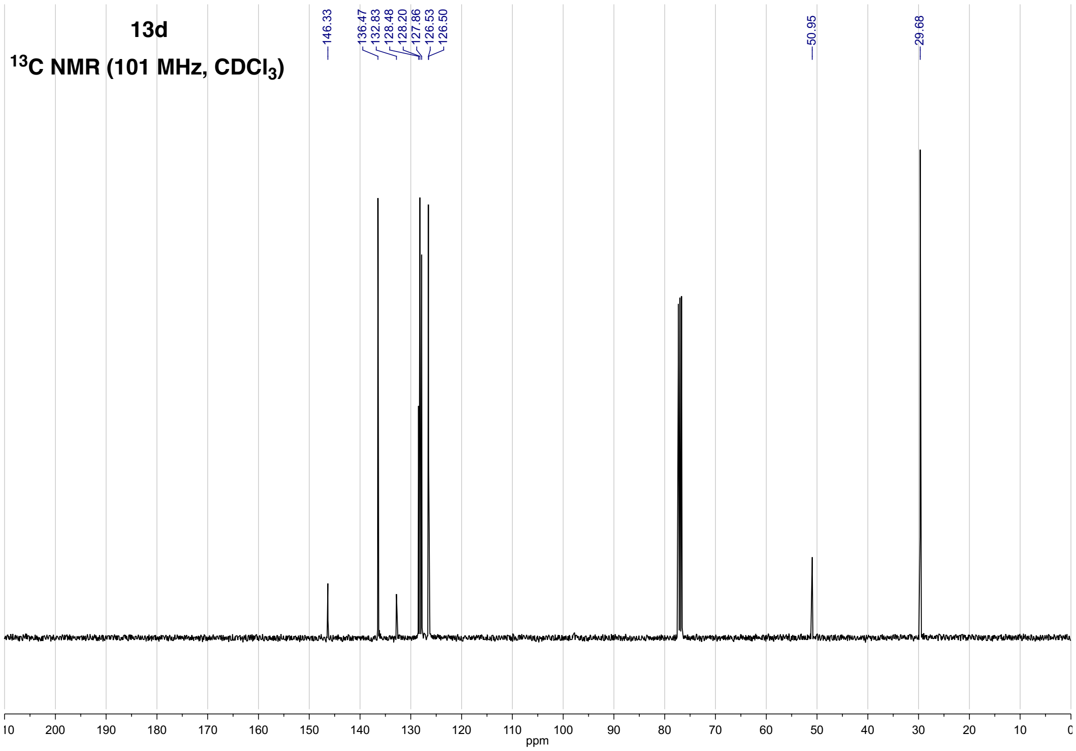
13d
^{13}C NMR (101 MHz, $CDCl_3$)
146.33
136.47
132.83
128.48
128.20
127.86
126.53
126.50
50.95
29.68
ppm

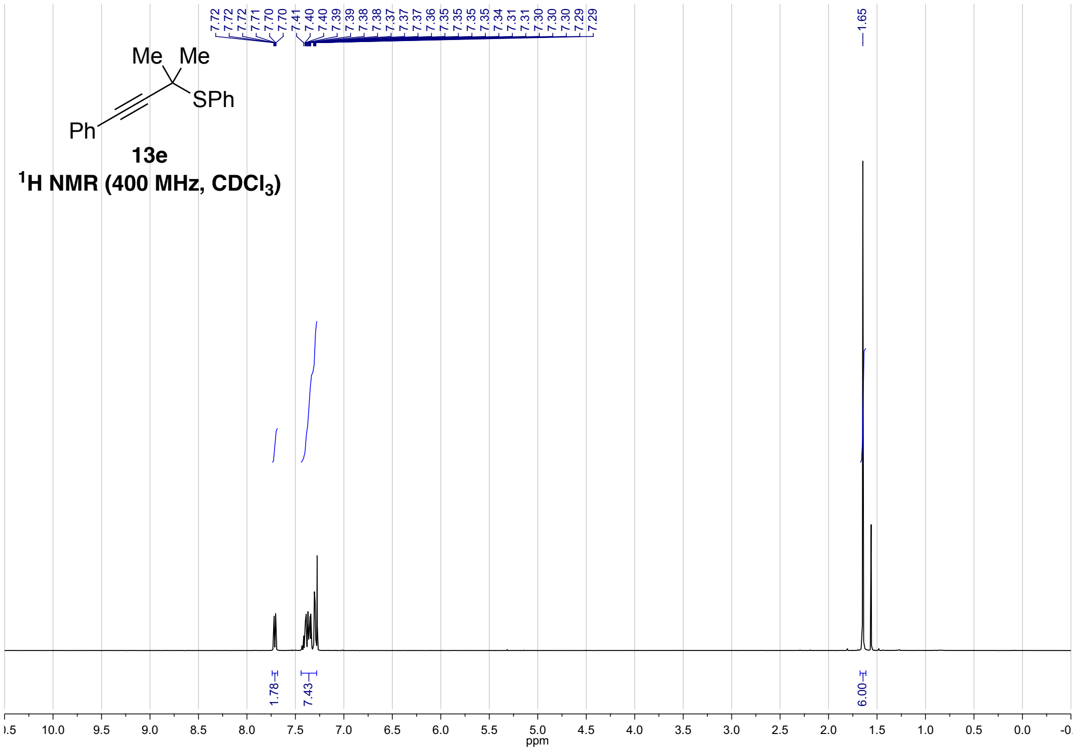

Me Me
SPh
Ph
13e
1H NMR (400 MHz, CDCl3)
7.72 7.72 7.72 7.71 7.70 7.70 7.41 7.40 7.40 7.39 7.39 7.38 7.38 7.37 7.37 7.37 7.36 7.35 7.35 7.35 7.35 7.34 7.31 7.31 7.30 7.30 7.30 7.29 7.29
1.65
1.78
7.43
6.00
ppm

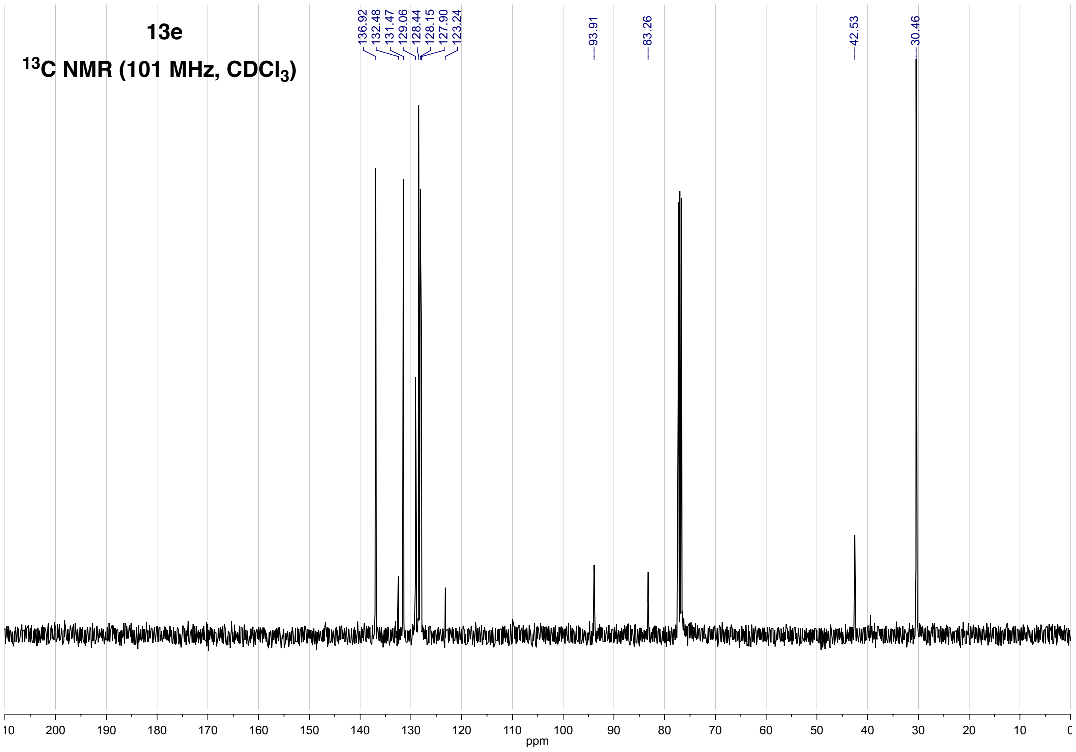

13e
13C NMR (101 MHz, CDCl3)
136.92 132.48 131.47 129.06 128.44 128.15 127.90 123.24
93.91
83.26
42.53
30.46
ppm

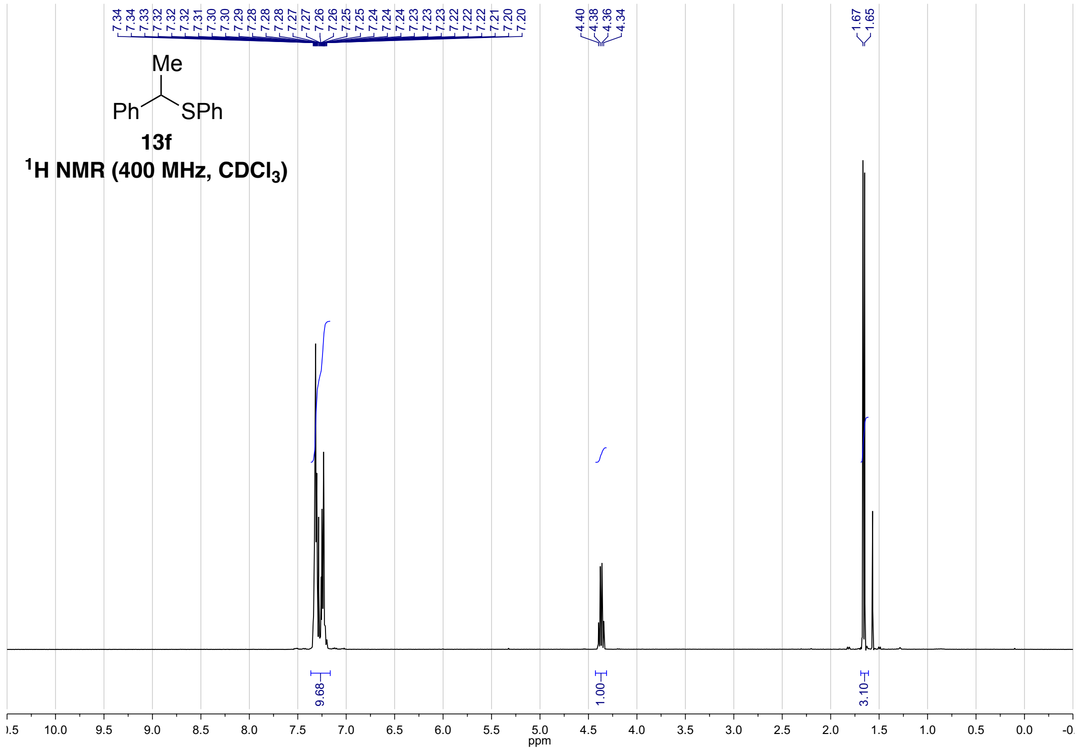
Me
Ph
SPh
13f
^{1}H NMR (400 MHz, CDCl$_3$)
9.68
1.00
3.10
ppm

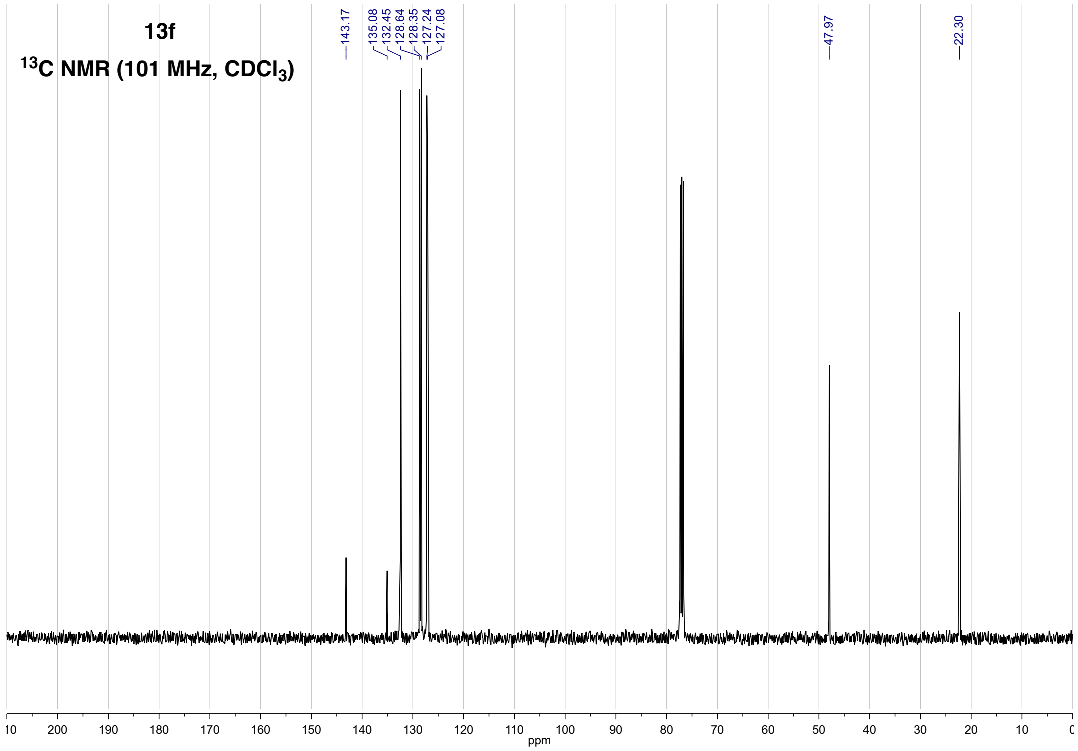
13f
^{13}C NMR (101 MHz, CDCl$_3$)
143.17
135.08
132.45
128.64
128.35
127.24
127.08
47.97
22.30
ppm

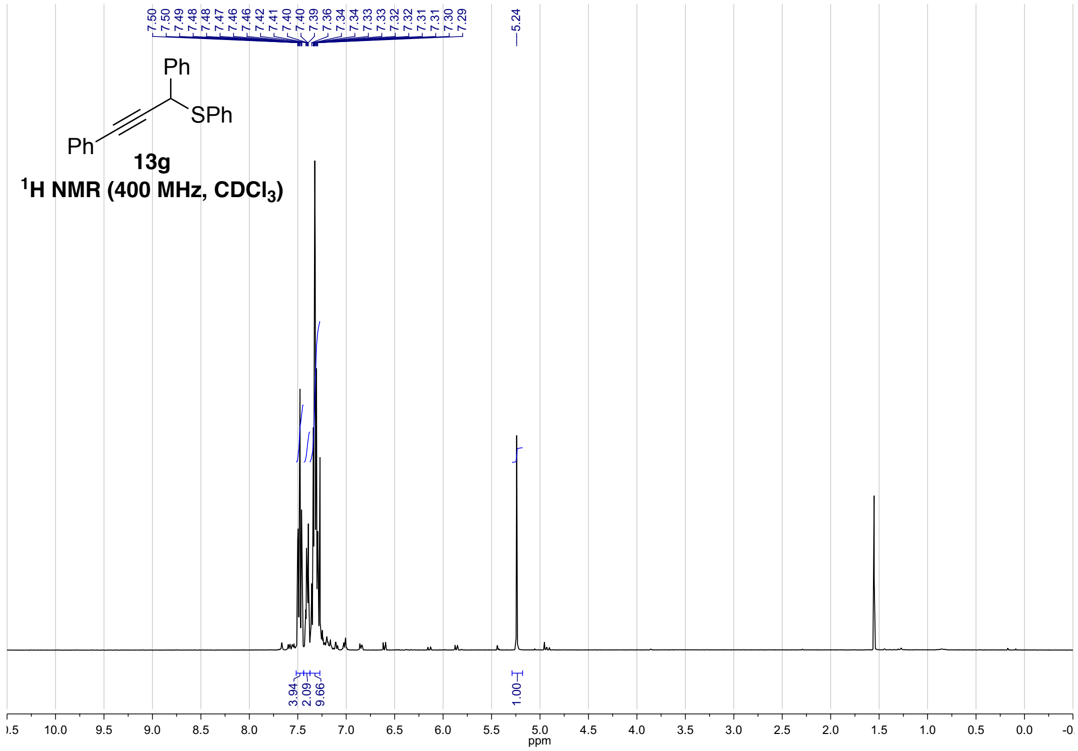
Ph
SPh
Ph
13g
^{1}H NMR (400 MHz, CDCl$_3$)
ppm

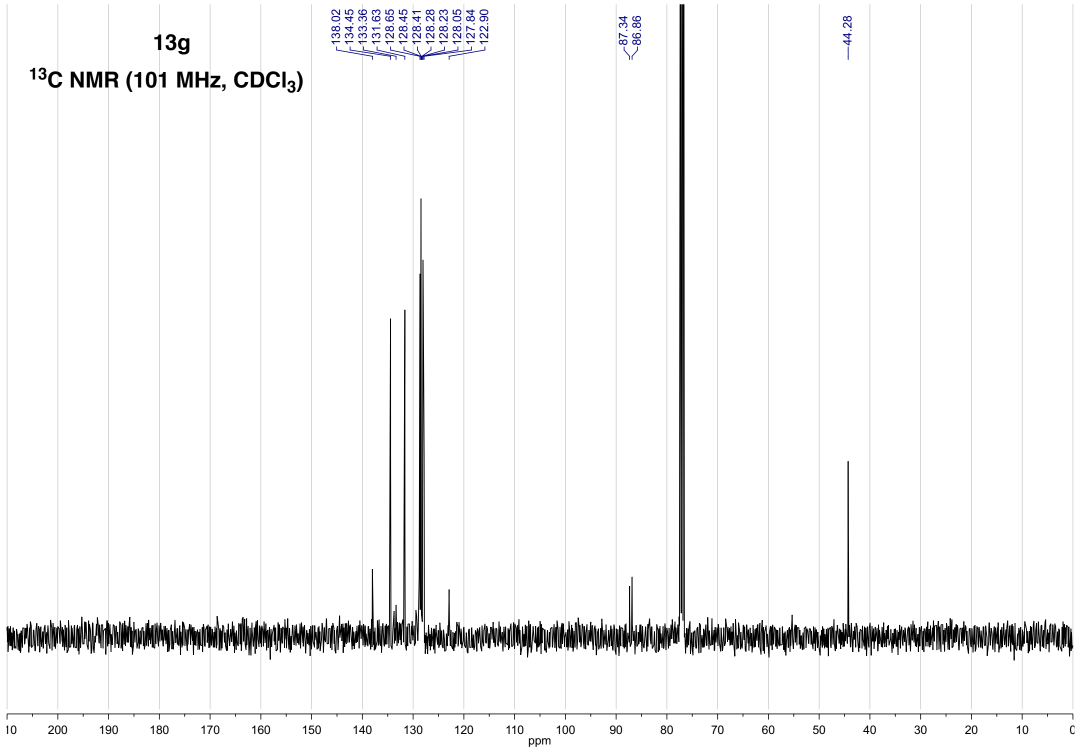
13g
^{13}C NMR (101 MHz, CDCl$_3$)
ppm

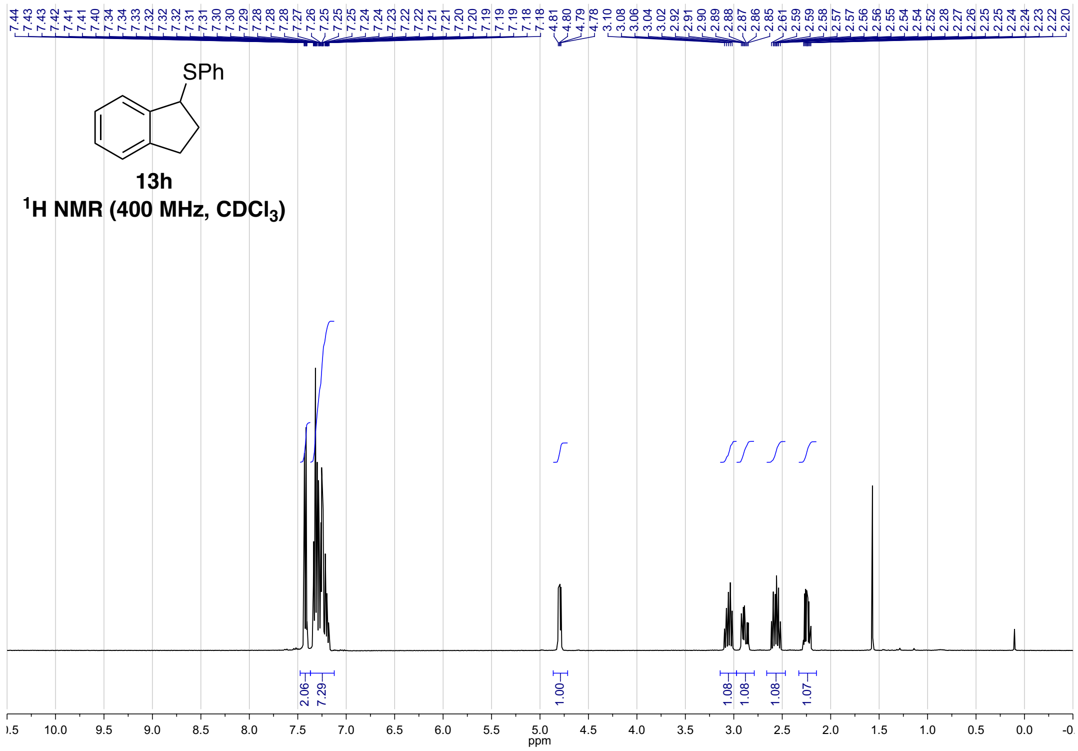
SPh
13h
^{1}H NMR (400 MHz, CDCl$_3$)
7.44 7.43 7.43 7.42 7.41 7.41 7.40 7.34 7.34 7.33 7.32 7.32 7.32 7.31 7.31 7.30 7.30 7.29 7.28 7.28 7.28 7.27 7.26 7.25 7.25 7.24 7.24 7.23 7.22 7.22 7.21 7.21 7.20 7.20 7.19 7.19 7.18 7.18
4.81 4.80 4.79 4.78
3.10 3.08 3.06 3.04 3.02 2.92 2.91 2.90 2.89 2.88 2.87 2.86 2.85
2.61 2.59 2.59 2.58 2.57 2.57 2.56 2.55 2.54 2.54 2.52 2.28 2.27 2.26 2.25 2.25 2.24 2.24 2.23 2.22 2.22 2.20
2.06 7.29 1.00 1.08 1.08 1.08 1.07
ppm

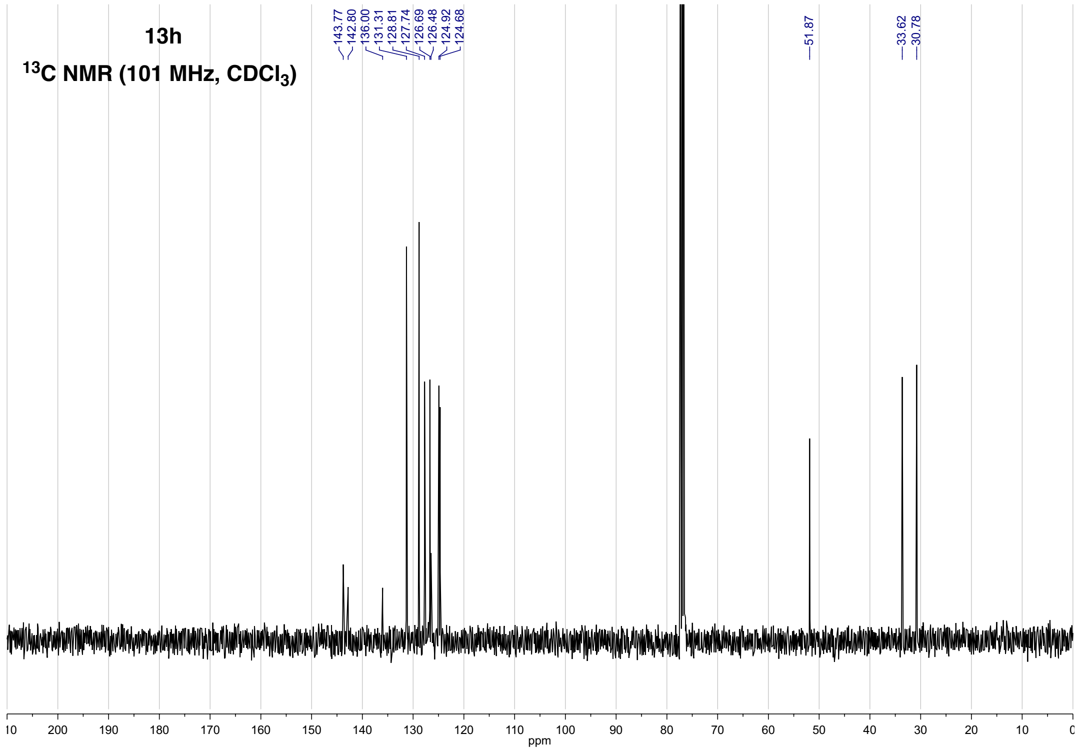
13h
^{13}C NMR (101 MHz, CDCl$_3$)
143.77 142.80 136.00 131.31 128.81 127.74 126.69 126.48 124.92 124.68
51.87
33.62 30.78
ppm

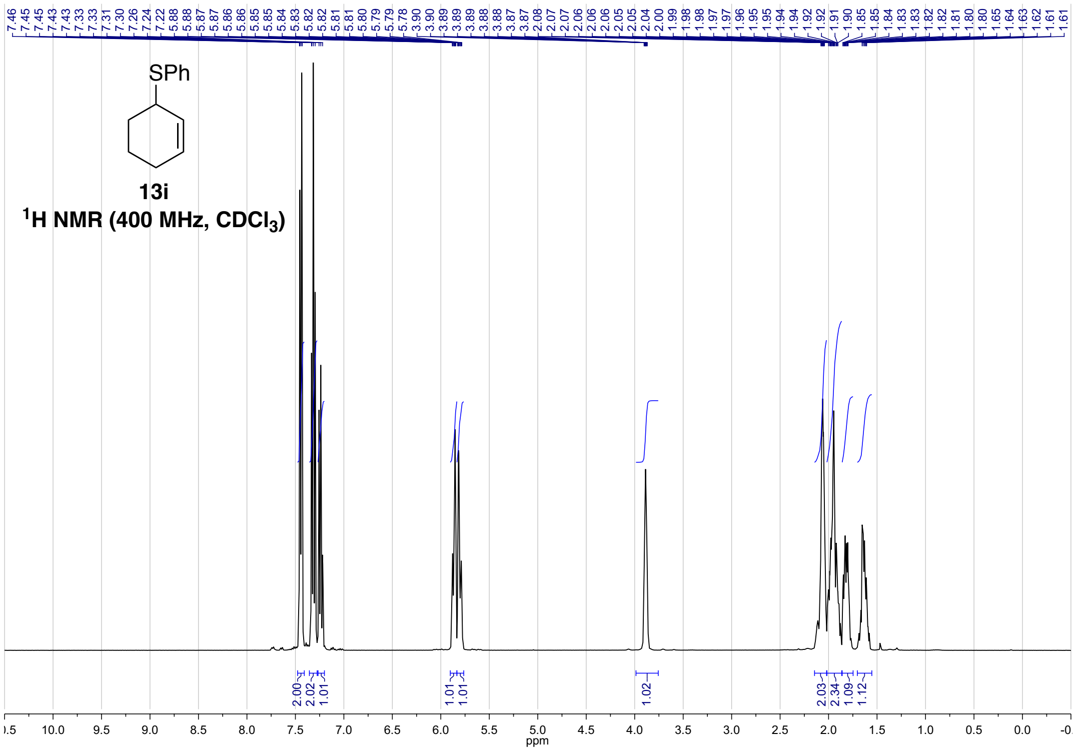
7.46 7.45 7.45 7.43 7.43 7.33 7.33 7.31 7.30 7.26 7.24 7.22 5.88 5.88 5.87 5.87 5.86 5.86 5.85 5.85 5.84 5.83 5.82 5.82 5.81 5.81 5.80 5.79 5.79 5.78 3.90 3.89 3.89 3.89 3.88 3.88 3.87 2.08 2.07 2.07 2.06 2.06 2.06 2.05 2.04 2.00 1.99 1.98 1.98 1.97 1.97 1.96 1.95 1.95 1.94 1.94 1.92 1.92 1.91 1.90 1.85 1.84 1.83 1.83 1.82 1.82 1.81 1.80 1.80 1.65 1.64 1.63 1.62 1.61 1.61
SPh
13i
^{1}H NMR (400 MHz, $CDCl_3$)
2.00 2.02 1.01 1.01 1.01 1.02 2.03 2.34 1.09 1.12
ppm

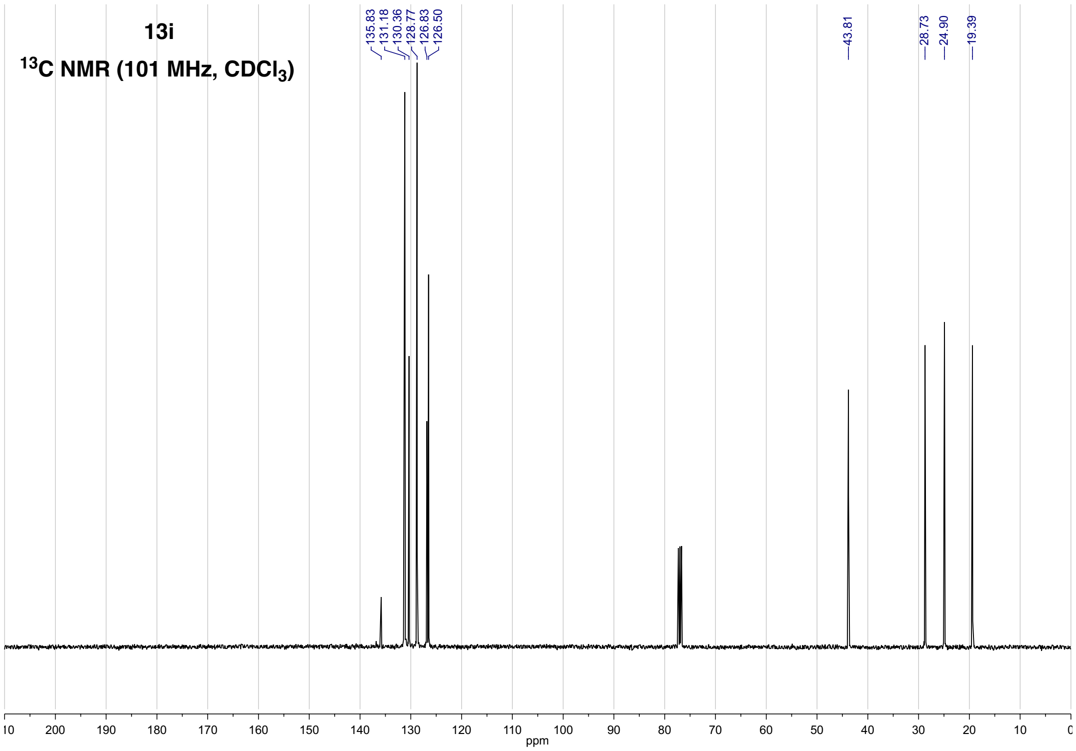
13i
^{13}C NMR (101 MHz, $CDCl_3$)
135.83 131.18 130.36 128.77 126.83 126.50
43.81
28.73 24.90 19.39
ppm

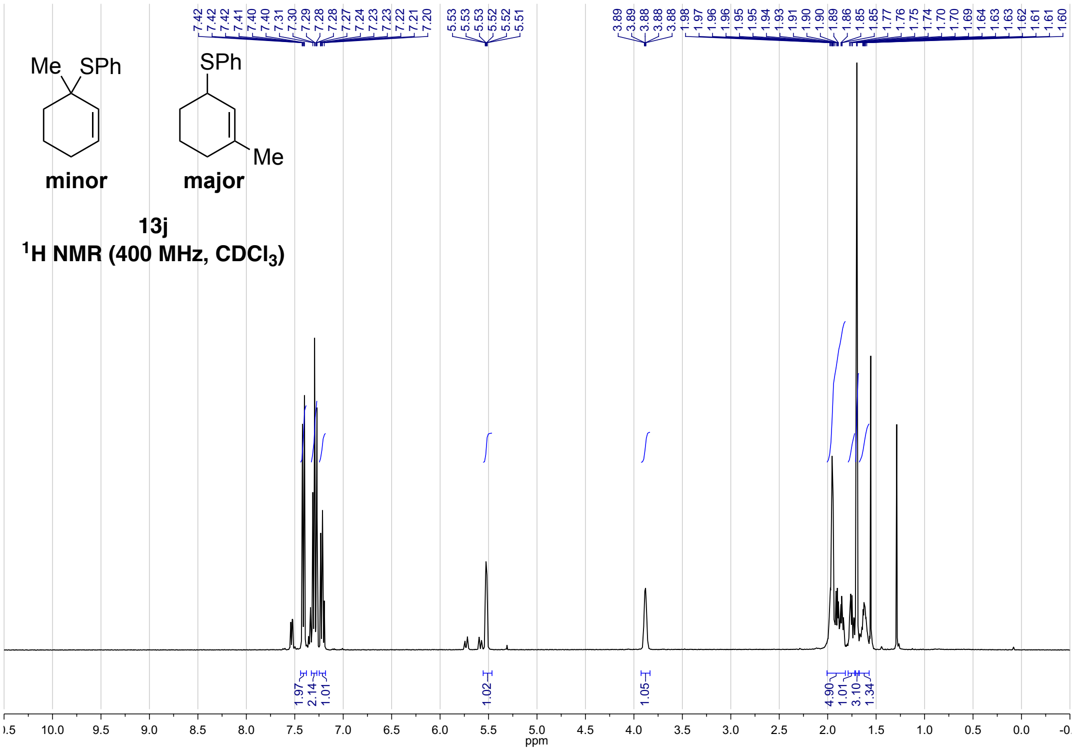
Me SPh
minor
SPh
Me
major
13j
1H NMR (400 MHz, CDCl3)
7.42 7.42 7.42 7.41 7.40 7.40 7.31 7.30 7.29 7.28 7.28 7.27 7.24 7.23 7.23 7.22 7.21 7.20
5.53 5.53 5.53 5.52 5.52 5.51
3.89 3.89 3.88 3.88 3.88
1.98 1.97 1.96 1.96 1.95 1.94 1.93 1.91 1.90 1.90 1.89 1.86 1.85 1.85 1.77 1.76 1.75 1.74 1.70 1.70 1.69 1.64 1.63 1.63 1.62 1.61 1.61 1.60
1.97 2.14 1.01 1.02 1.05 4.90 1.01 3.10 1.34
ppm

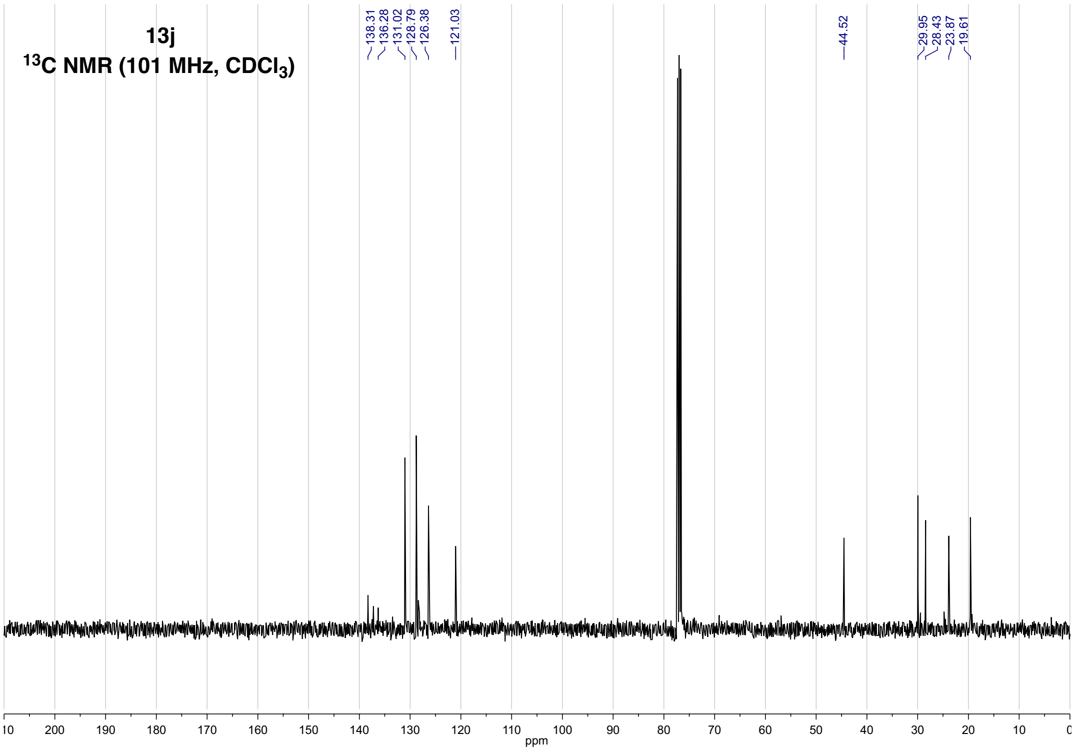
13j
13C NMR (101 MHz, CDCl3)
138.31 136.28 131.02 128.79 126.38 121.03
44.52
29.95 28.43 23.87 19.61
ppm

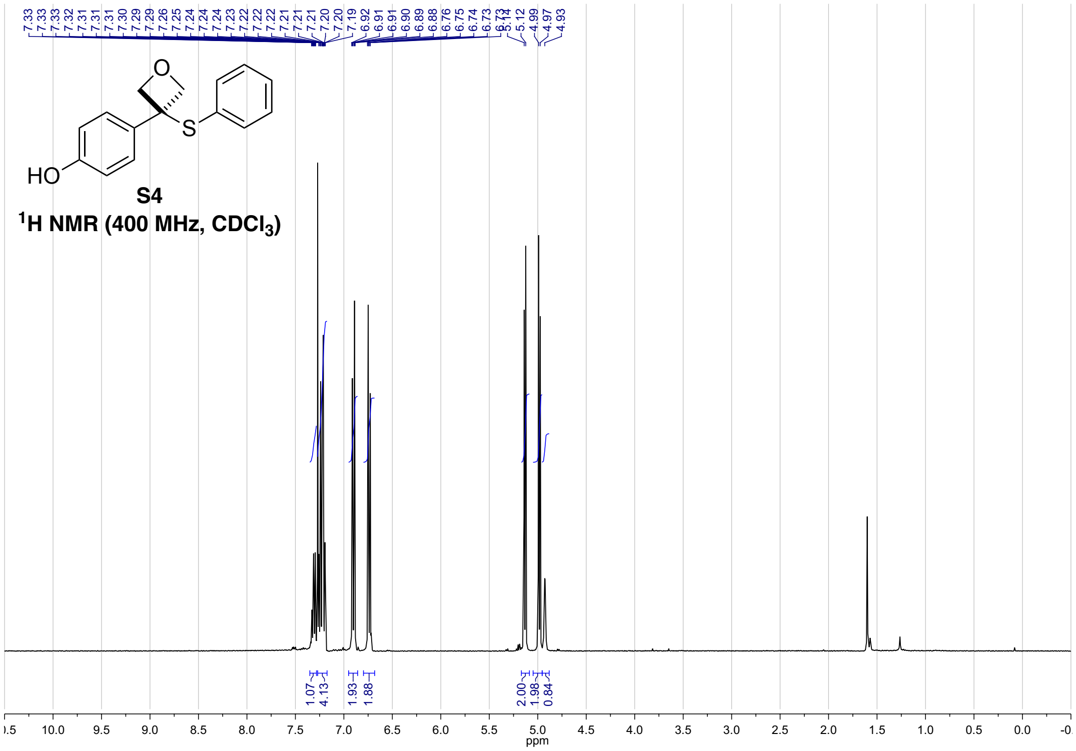
HO
O
S
S4
^{1}H NMR (400 MHz, $CDCl_3$)
1.07
4.13
1.93
1.88
2.00
1.98
0.84
ppm

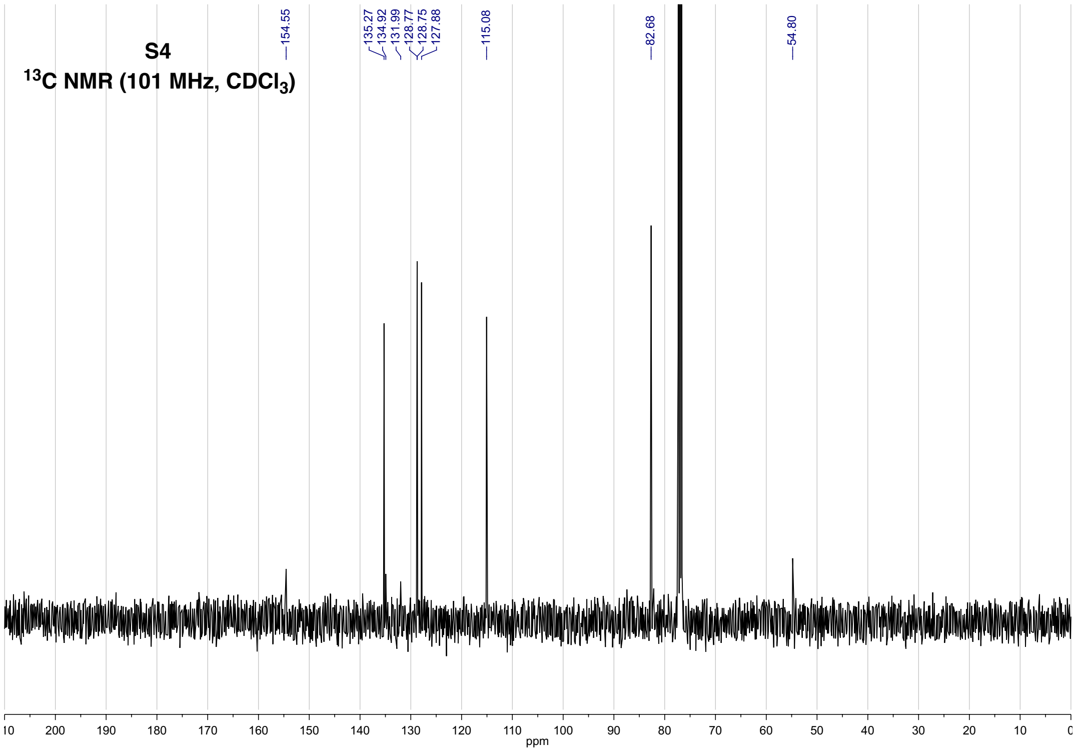
S4
^{13}C NMR (101 MHz, $CDCl_3$)
154.55
135.27
134.92
131.99
128.77
128.75
127.88
115.08
82.68
54.80
ppm

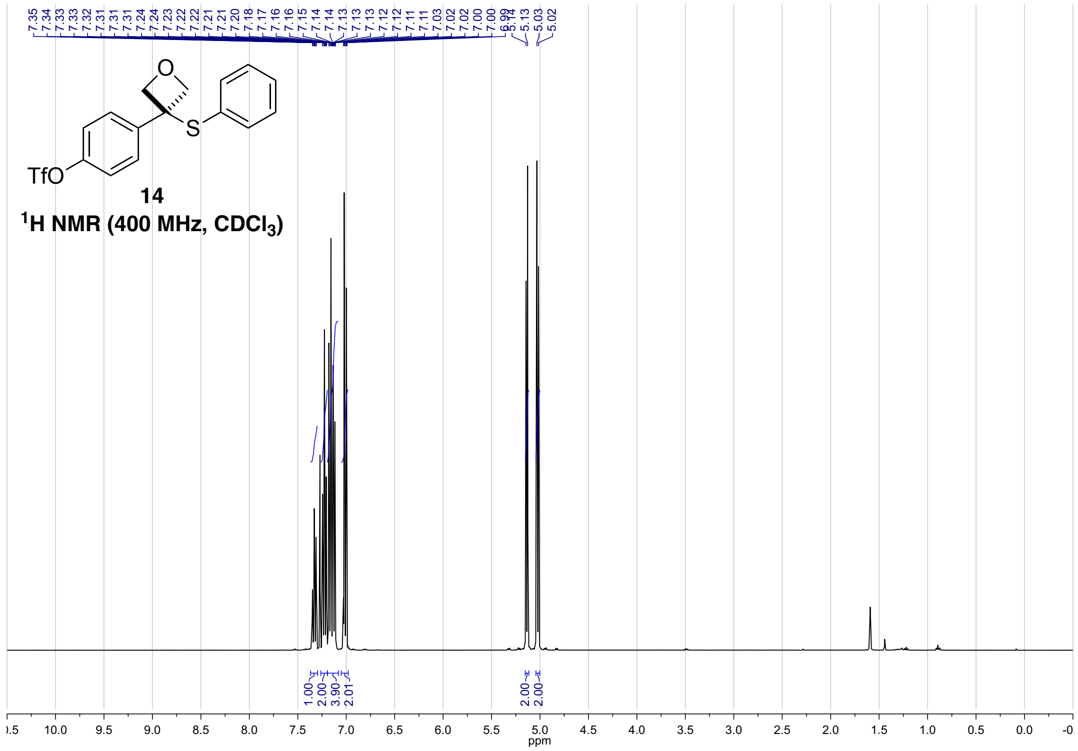
7.35 7.34 7.33 7.32 7.31 7.31 7.31 7.24 7.24 7.23 7.22 7.22 7.21 7.21 7.20 7.17 7.16 7.15 7.14 7.14 7.13 7.13 7.13 7.12 7.12 7.11 7.11 7.03 7.02 7.02 7.00 7.00 6.99 5.14 5.13 5.03 5.02
TfO
O
S
14
1H NMR (400 MHz, CDCl3)
1.00 2.00 3.90 2.01 2.00 2.00
ppm

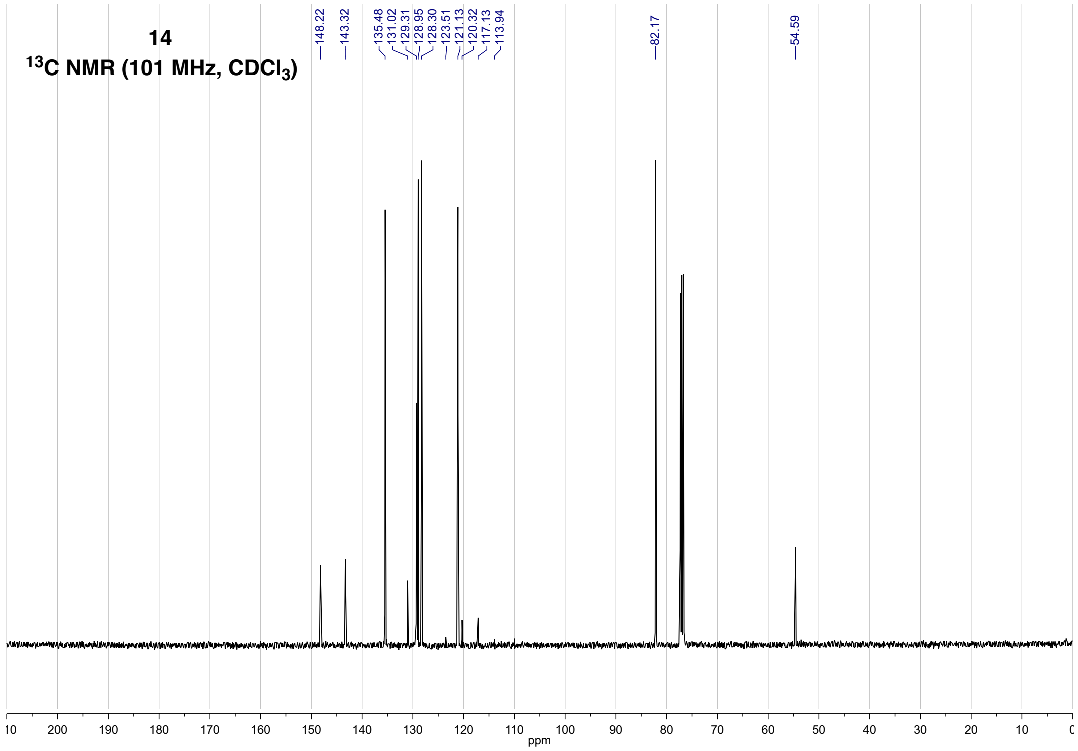
14
13C NMR (101 MHz, CDCl3)
148.22 143.32 135.48 131.02 129.31 128.95 128.30 123.51 121.13 120.32 117.13 113.94 82.17 54.59
ppm

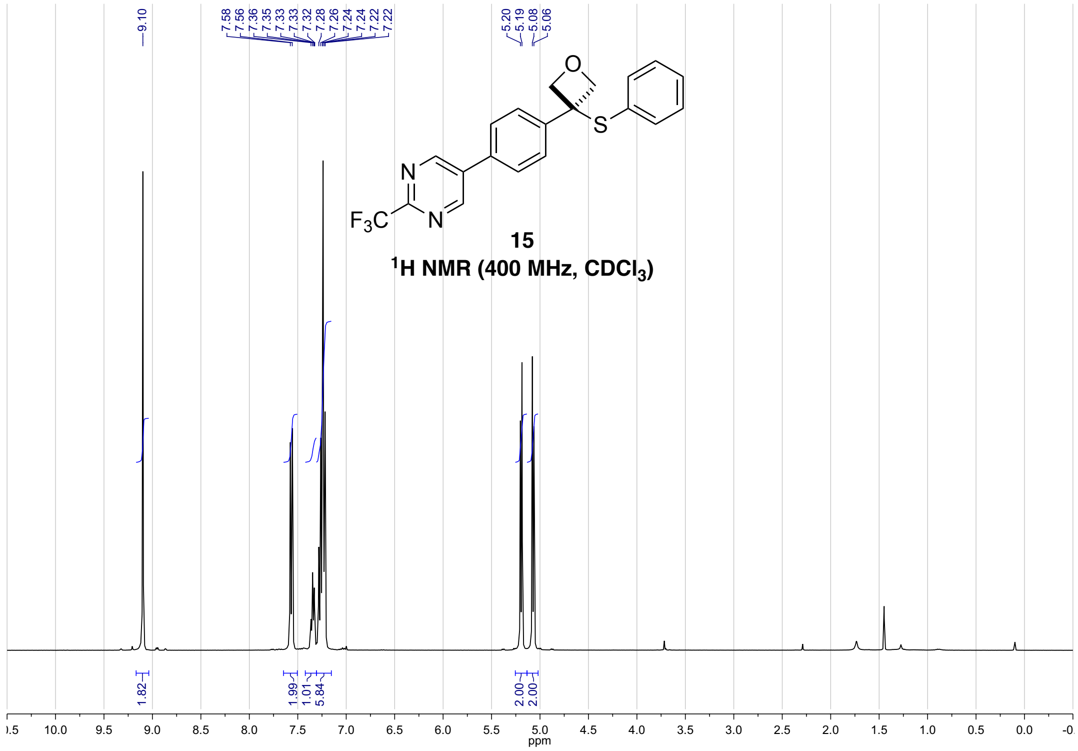
9.10
7.58
7.56
7.36
7.35
7.33
7.33
7.32
7.28
7.26
7.24
7.24
7.22
7.22
5.20
5.19
5.08
5.06
O
S
N
N
F3C
15
1H NMR (400 MHz, CDCl3)
1.82
1.99
1.01
5.84
2.00
2.00
ppm

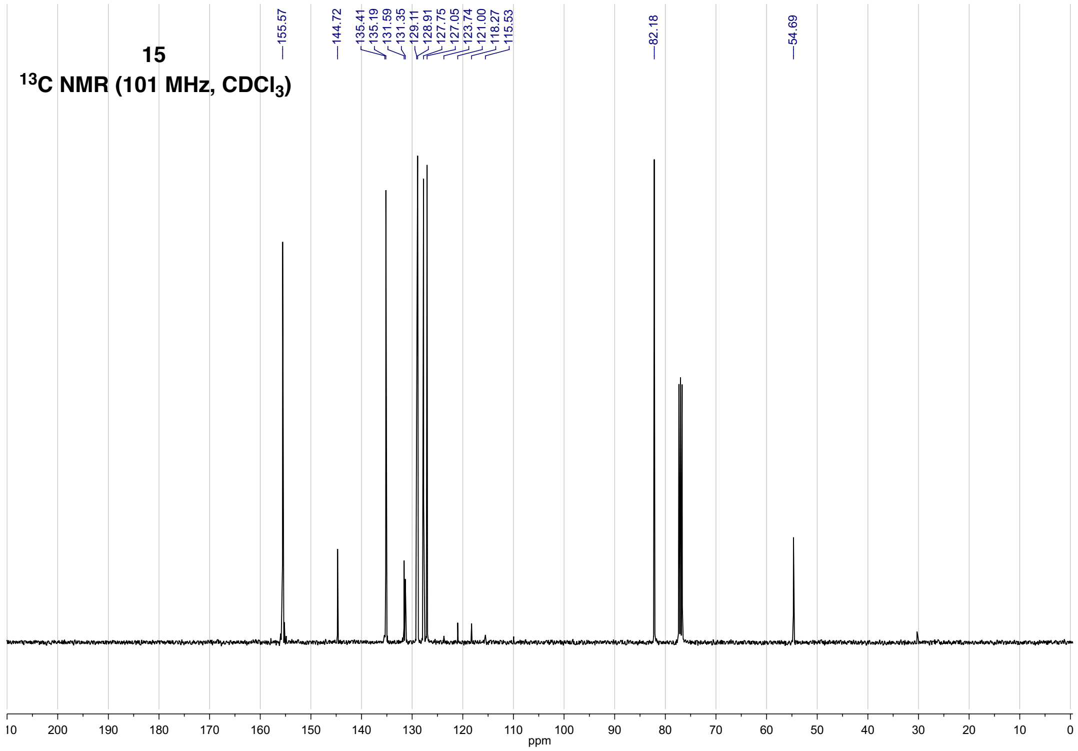
155.57
144.72
135.41
135.19
131.59
131.35
129.11
128.91
127.75
127.05
123.74
121.00
118.27
115.53
82.18
54.69
15
13C NMR (101 MHz, CDCl3)
ppm

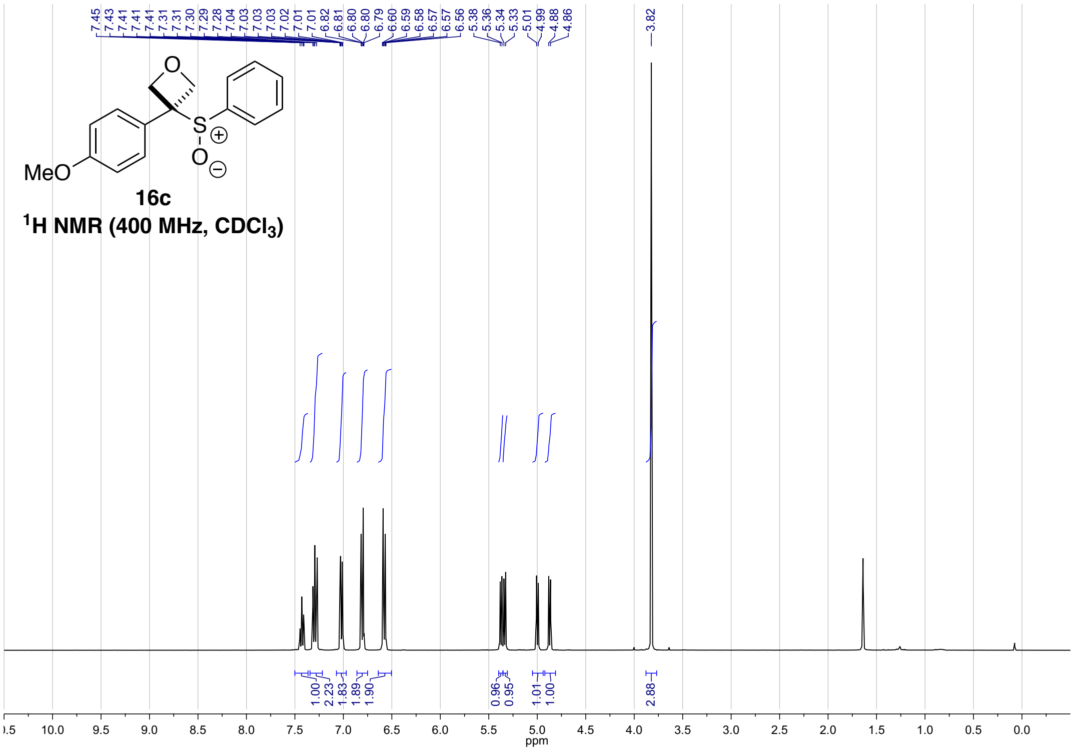
16c
1H NMR (400 MHz, CDCl3)
ppm

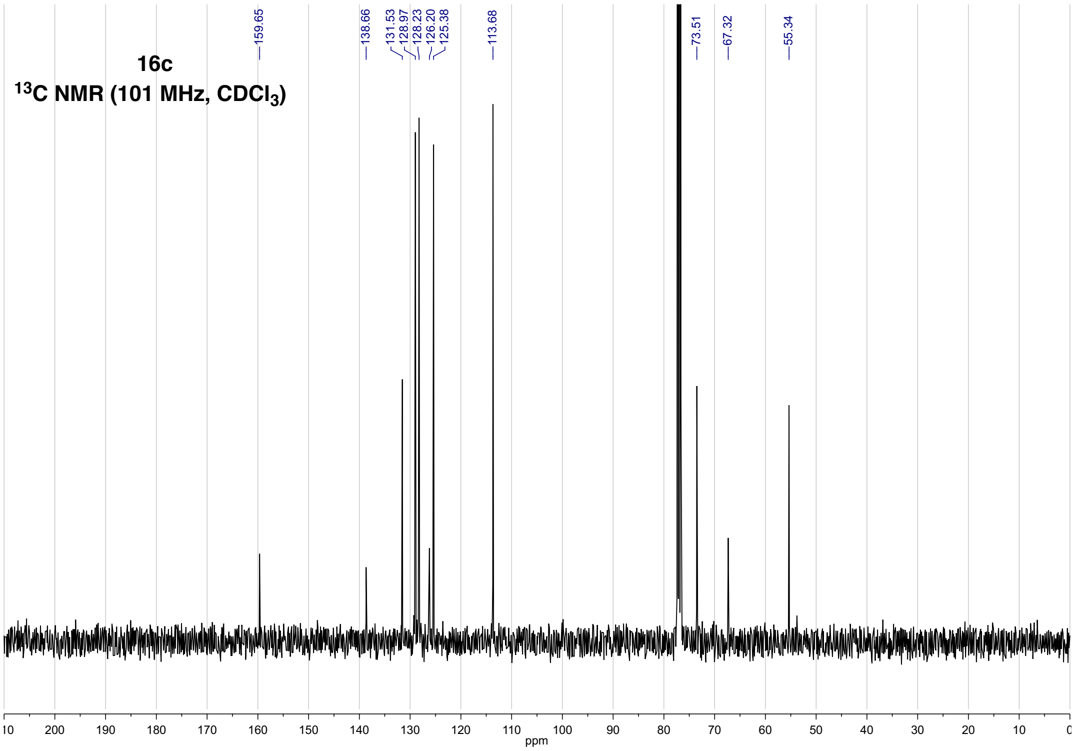
16c
13C NMR (101 MHz, CDCl3)
159.65
138.66
131.53
128.97
128.23
126.20
125.38
113.68
73.51
67.32
55.34
ppm

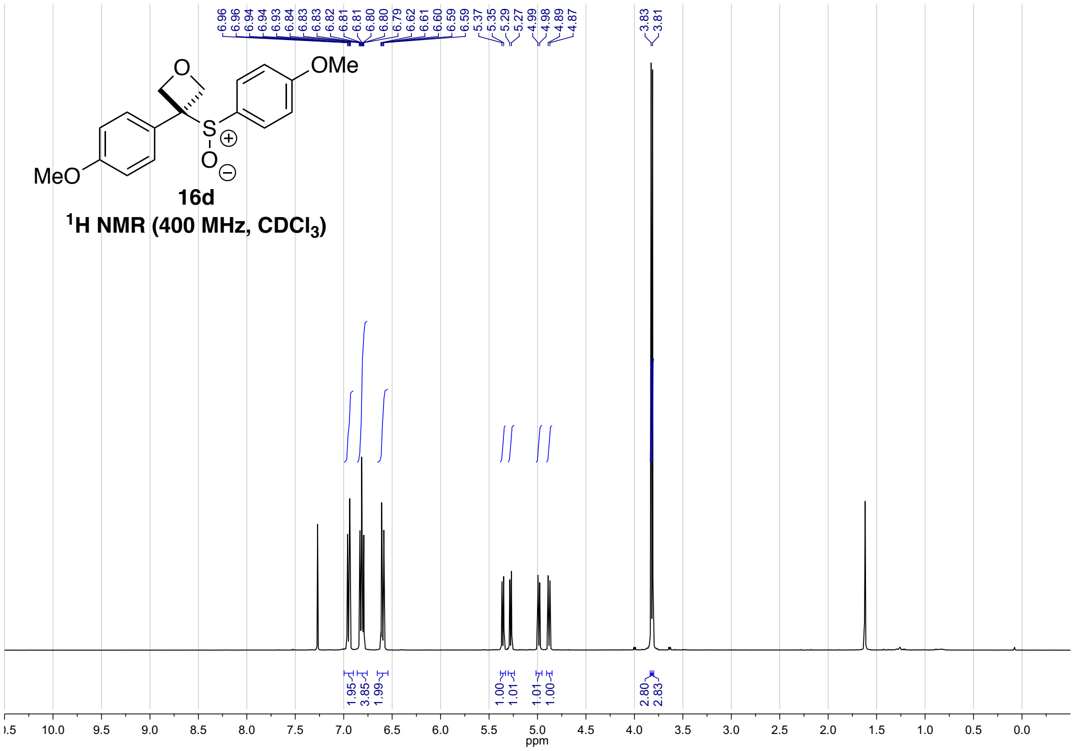
OMe
MeO
16d
^{1}H NMR (400 MHz, CDCl$_3$)
6.96 6.96 6.94 6.94 6.93 6.84 6.83 6.83 6.82 6.81 6.81 6.80 6.80 6.79 6.62 6.61 6.60 6.59 5.37 5.35 5.29 5.27 4.99 4.98 4.89 4.87 3.83 3.81
1.95 3.85 1.99 1.00 1.01 1.01 1.00 2.80 2.83
ppm

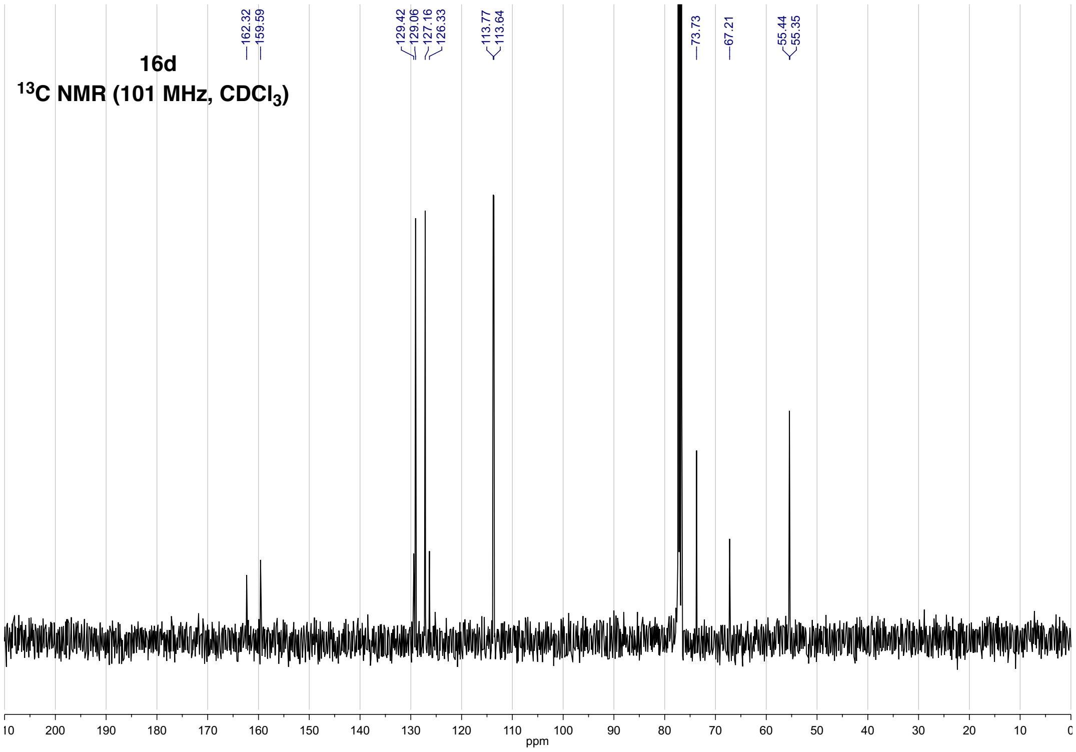
16d
^{13}C NMR (101 MHz, CDCl$_3$)
162.32 159.59 129.42 129.06 127.16 126.33 113.77 113.64 73.73 67.21 55.44 55.35
ppm

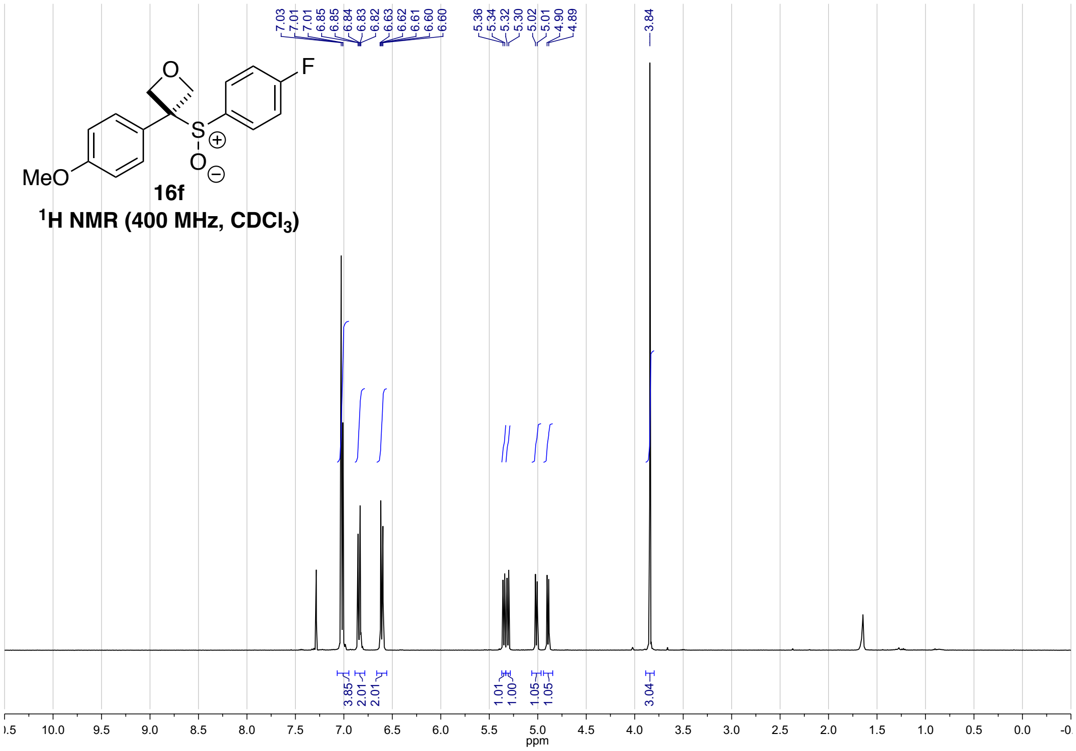
7.03
7.01
7.01
6.85
6.85
6.84
6.83
6.82
6.63
6.62
6.61
6.60
6.60
5.36
5.34
5.32
5.30
5.02
5.01
4.90
4.89
3.84
MeO
O
S
F
16f
^{1}H NMR (400 MHz, $CDCl_3$)
3.85
2.01
2.01
1.01
1.00
1.05
1.05
3.04
ppm

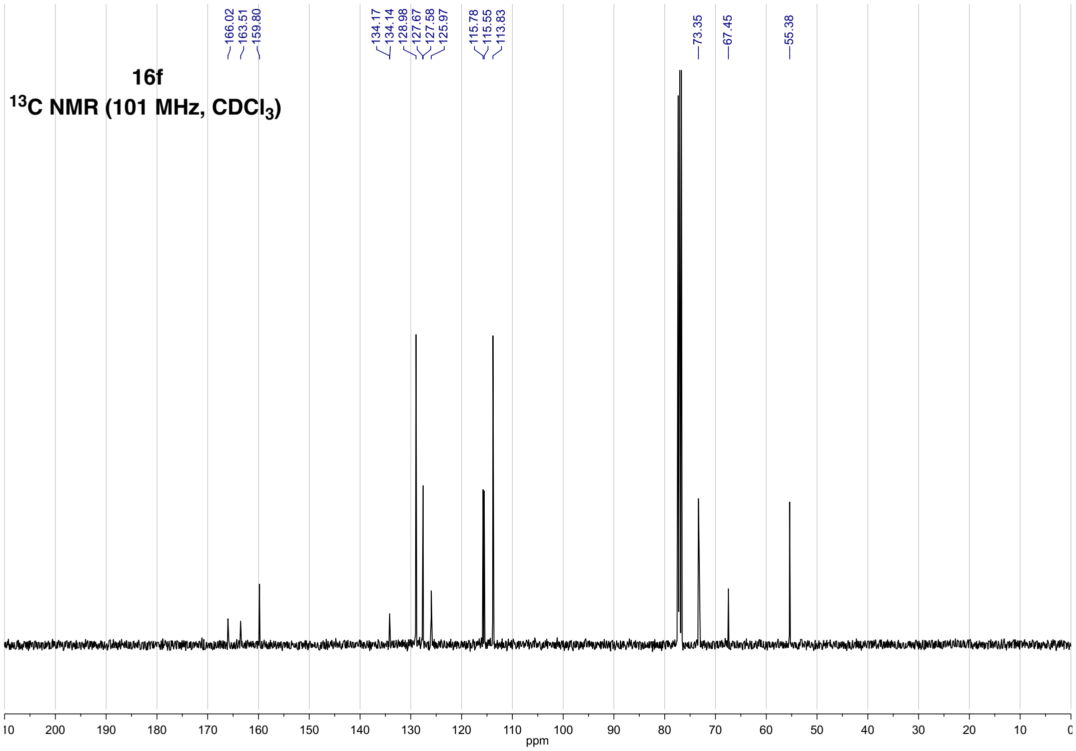
166.02
163.51
159.80
134.17
134.14
128.98
127.67
127.58
125.97
115.78
115.55
113.83
73.35
67.45
55.38
16f
^{13}C NMR (101 MHz, $CDCl_3$)
ppm

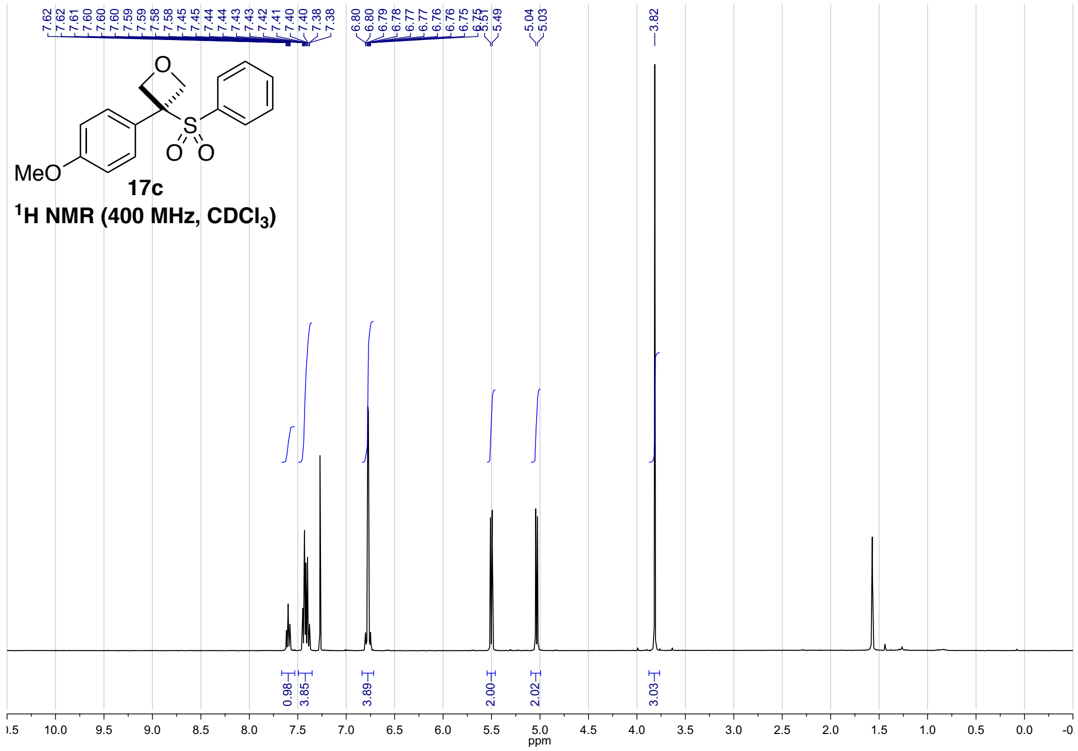
MeO
O
S
O
O
17c
1H NMR (400 MHz, CDCl3)
ppm

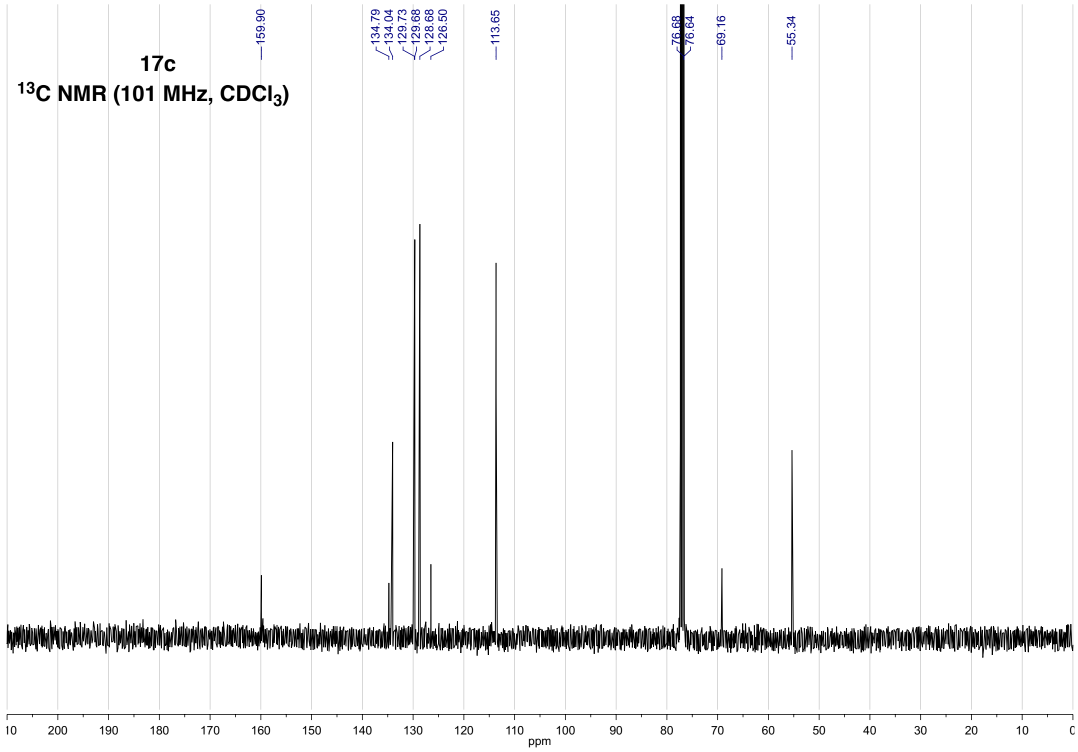
17c
13C NMR (101 MHz, CDCl3)
ppm

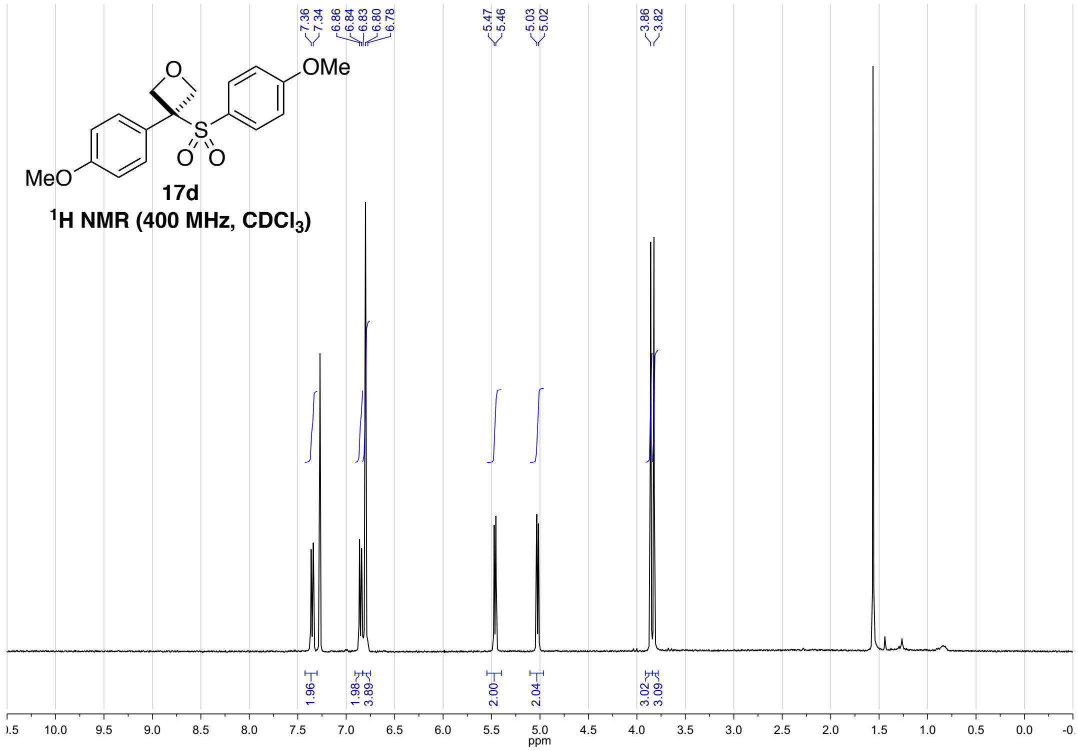
7.36
7.34
6.86
6.84
6.83
6.80
6.78
5.47
5.46
5.03
5.02
3.86
3.82
OMe
MeO
O
S
O O
17d
¹H NMR (400 MHz, CDCl₃)
1.96
1.98
3.89
2.00
2.04
3.02
3.09
ppm

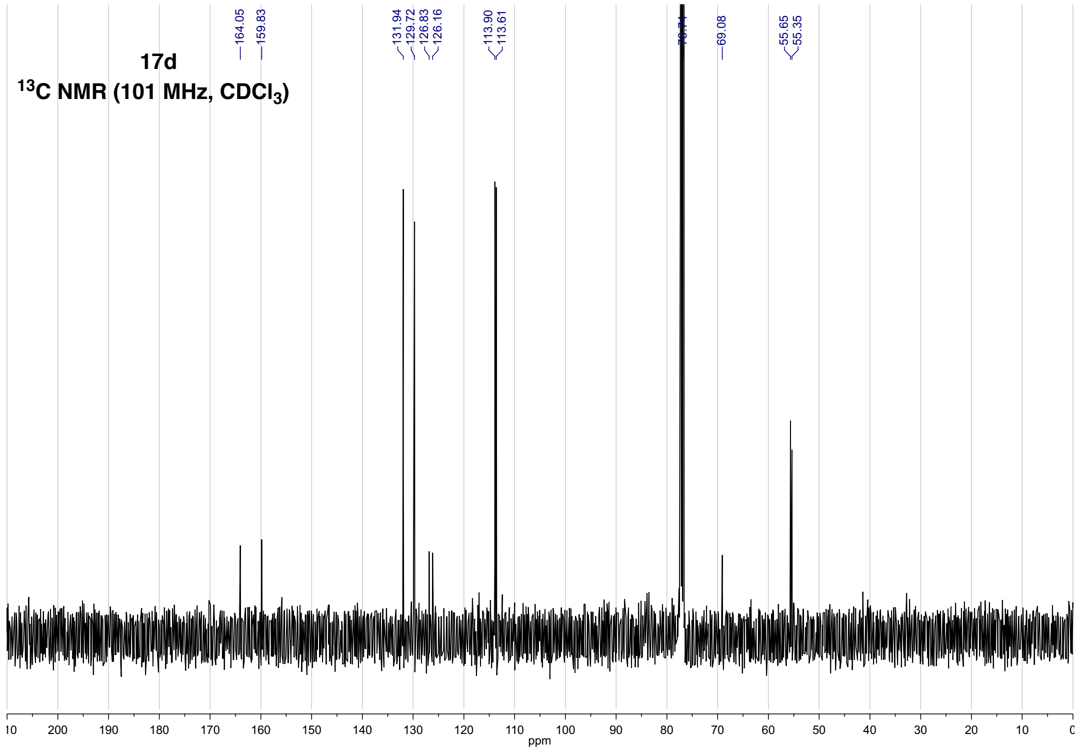
164.05
159.83
131.94
129.72
126.83
126.16
113.90
113.61
69.08
55.65
55.35
17d
¹³C NMR (101 MHz, CDCl₃)
ppm

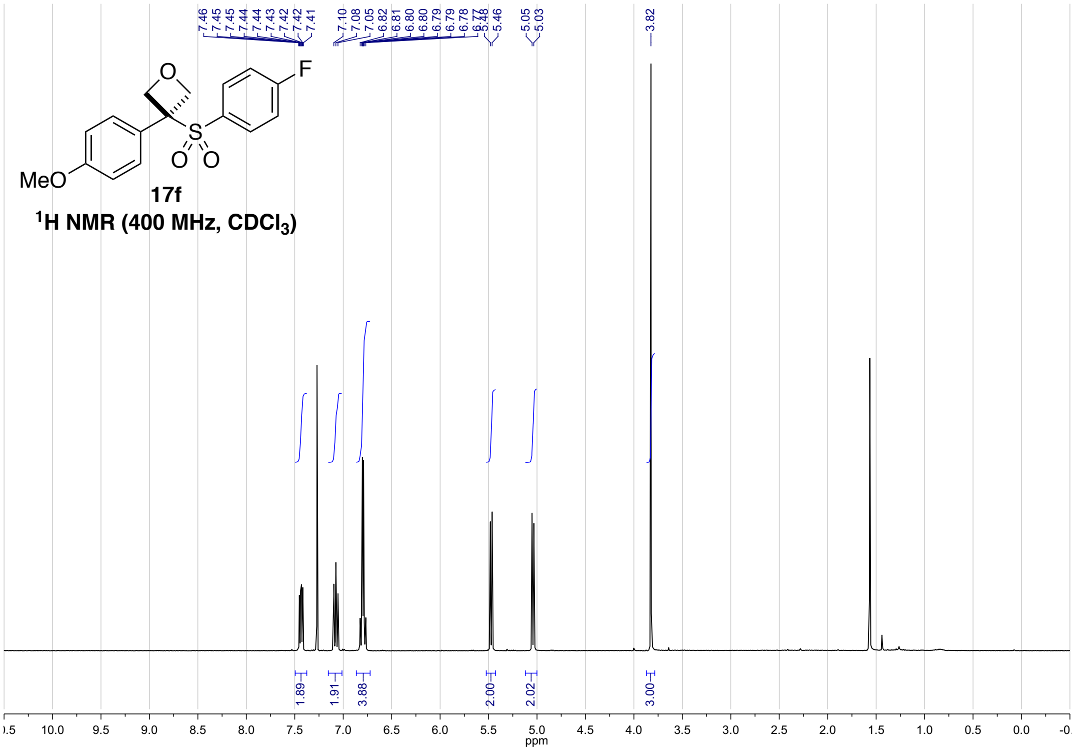
MeO
17f
^{1}H NMR (400 MHz, CDCl3)
ppm

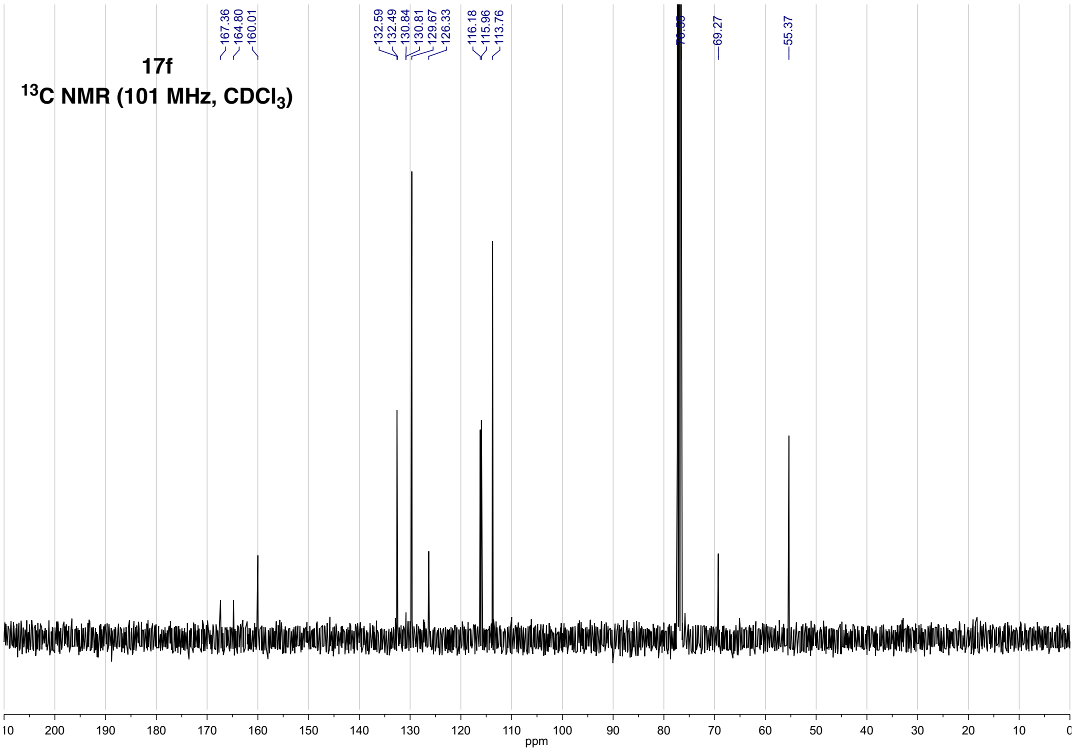
17f
^{13}C NMR (101 MHz, CDCl3)
ppm

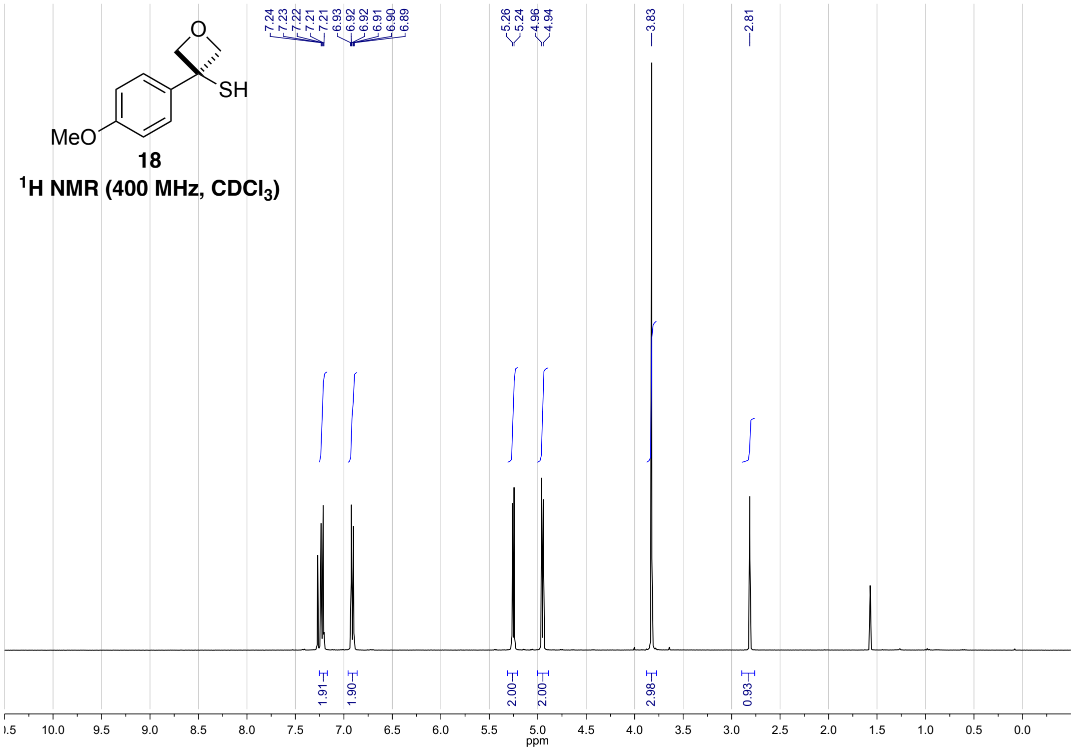
MeO
SH
O
18
1H NMR (400 MHz, CDCl3)
7.24
7.23
7.22
7.21
7.21
6.93
6.92
6.92
6.91
6.90
6.89
5.26
5.24
4.96
4.94
3.83
2.81
1.91
1.90
2.00
2.00
2.98
0.93
ppm

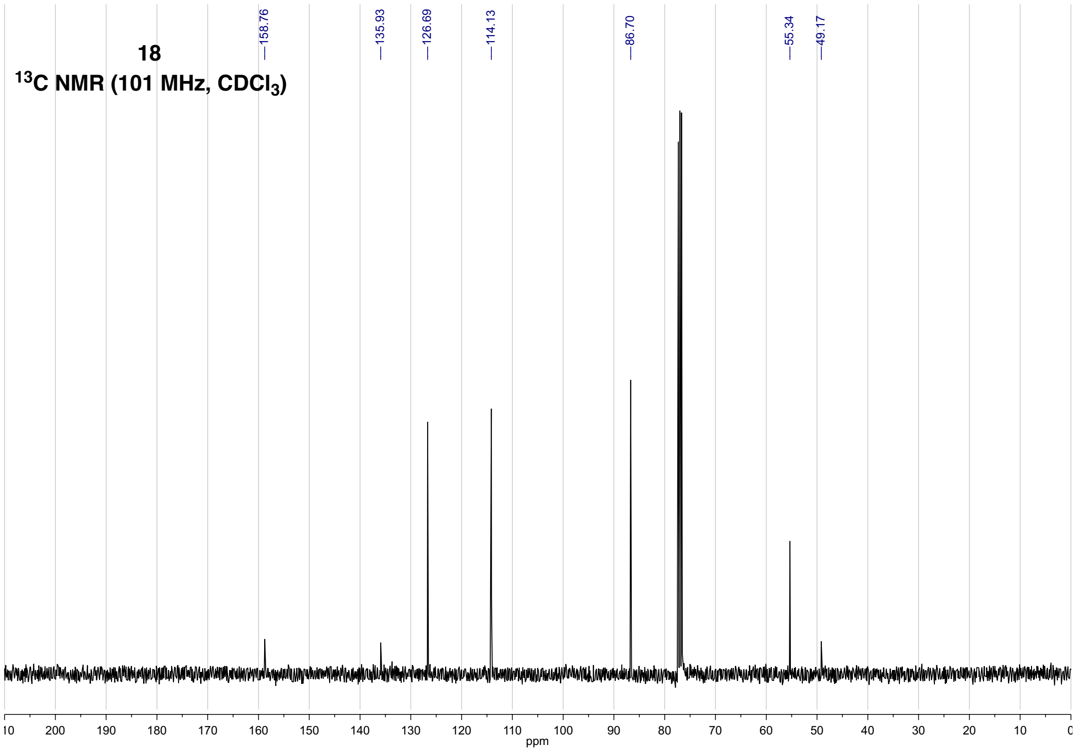
18
13C NMR (101 MHz, CDCl3)
158.76
135.93
126.69
114.13
86.70
55.34
49.17
ppm

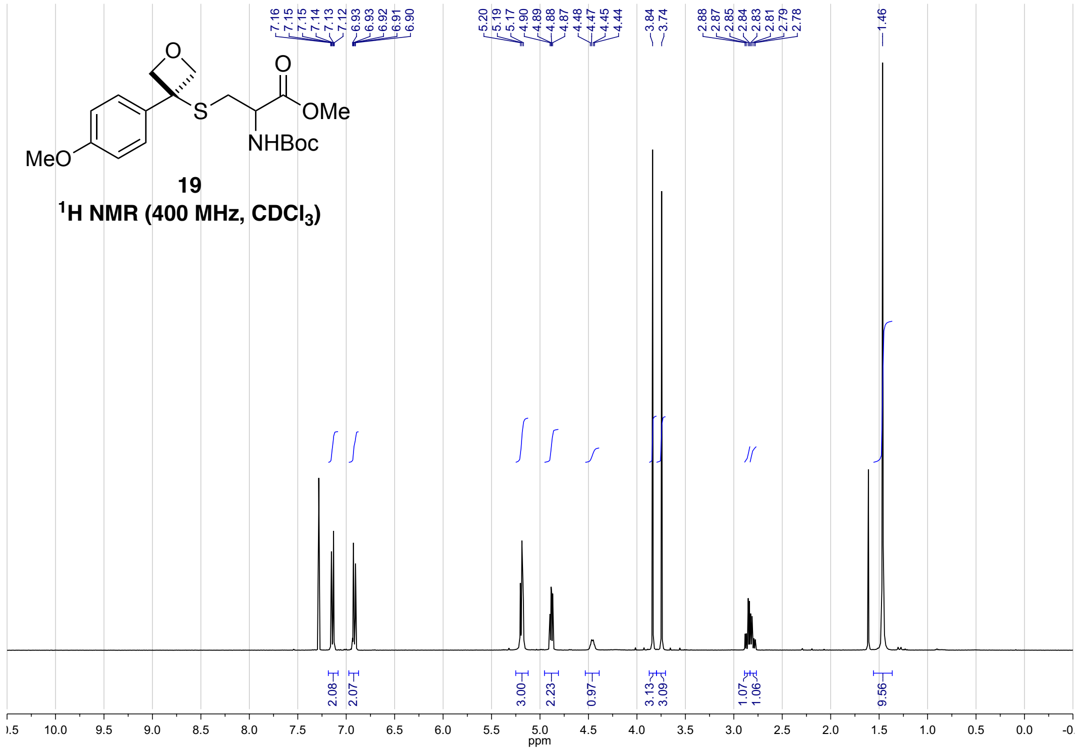
MeO
O
S
O
OMe
NHBoc
19
^{1}H NMR (400 MHz, CDCl$_3$)
ppm

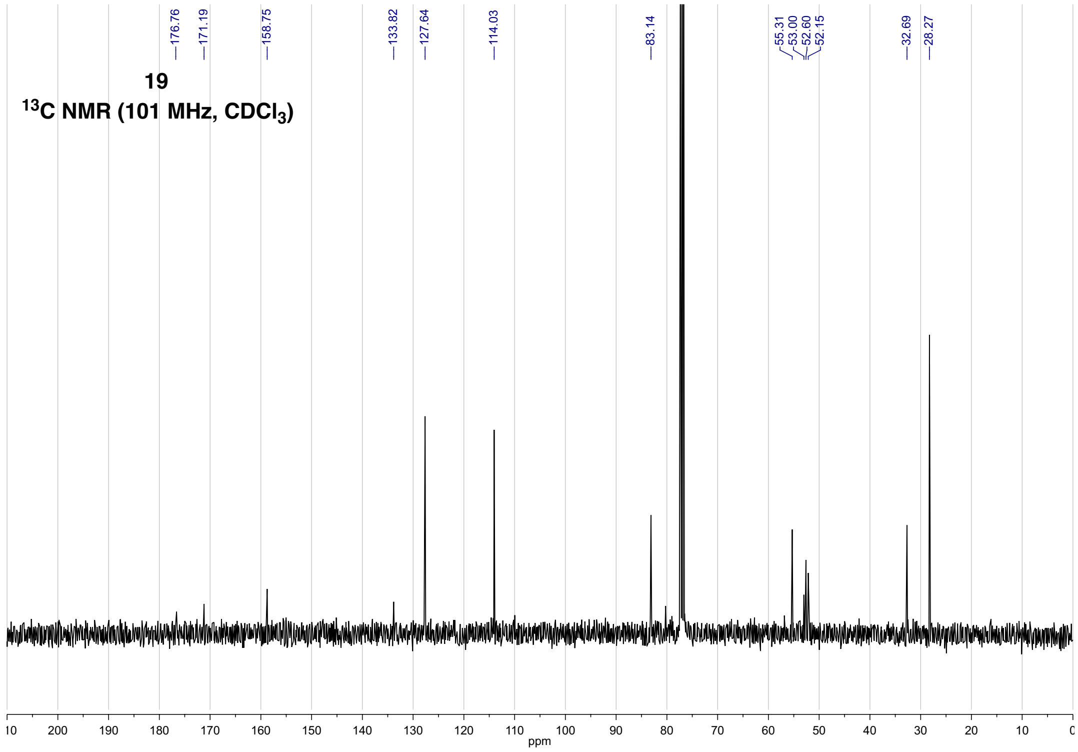
19
^{13}C NMR (101 MHz, CDCl$_3$)
ppm

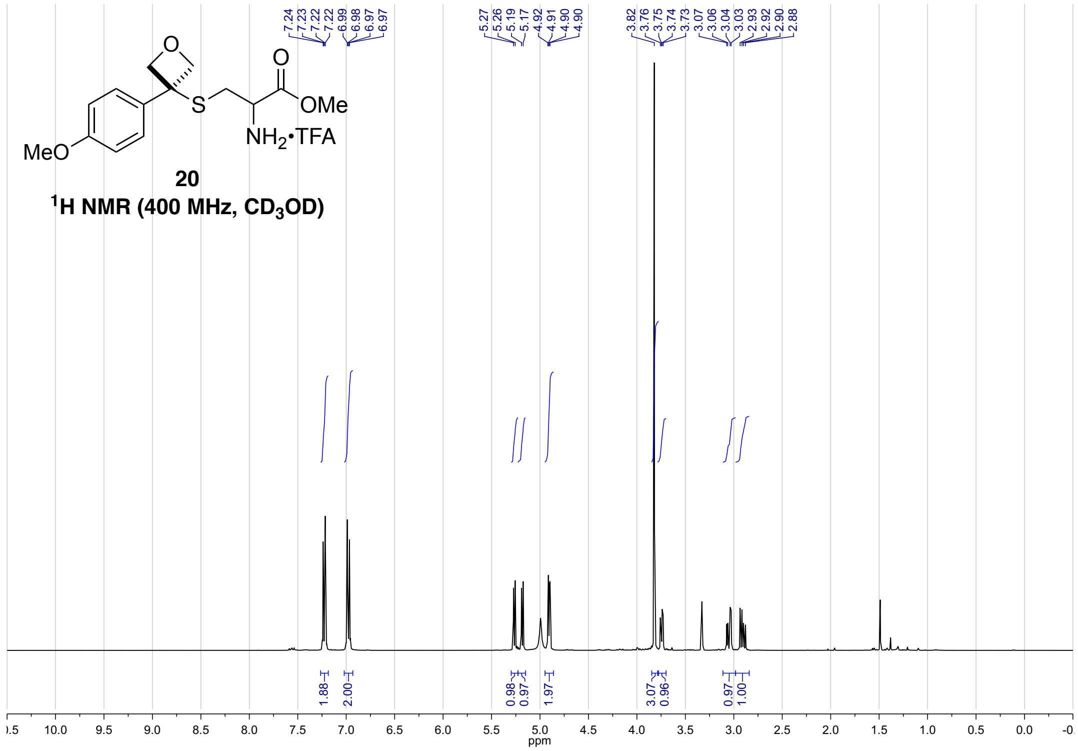

MeO
S
O
OMe
NH_2•TFA
20
^{1}H NMR (400 MHz, CD_3OD)
7.24
7.23
7.22
7.22
6.99
6.98
6.97
6.97
5.27
5.26
5.19
5.17
4.92
4.91
4.90
4.90
3.82
3.76
3.75
3.74
3.73
3.07
3.06
3.04
3.03
2.93
2.92
2.90
2.88
1.88
2.00
0.98
0.97
1.97
3.07
0.96
0.97
1.00
ppm

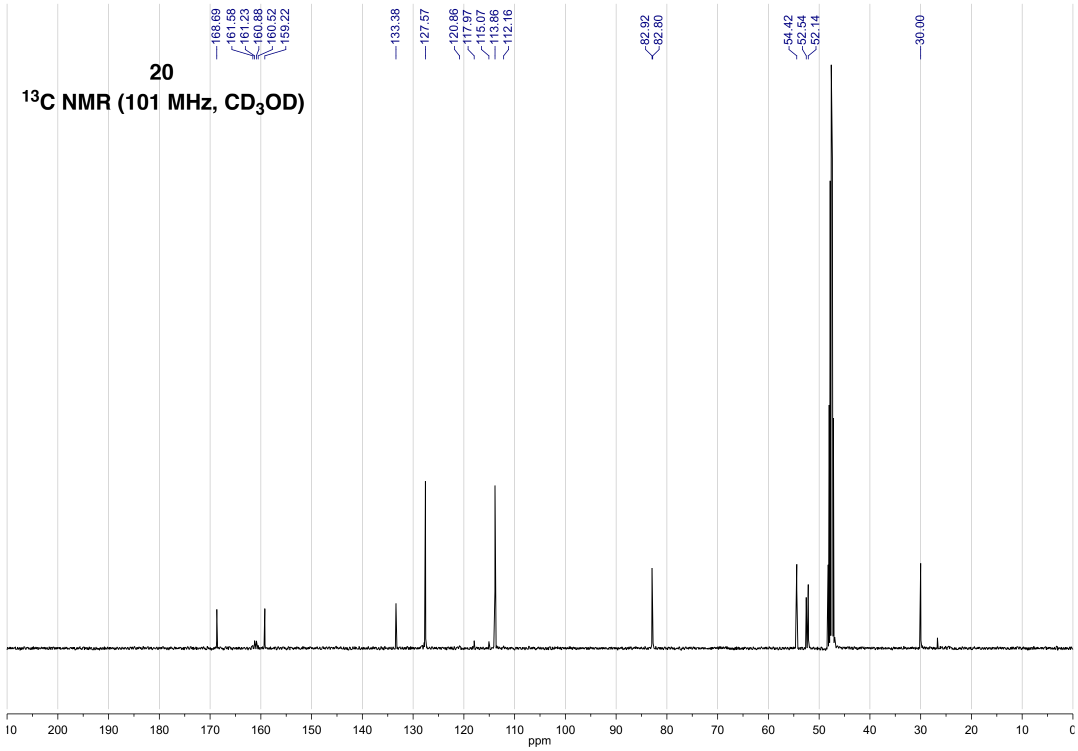

20
^{13}C NMR (101 MHz, CD_3OD)
168.69
161.58
161.23
160.88
160.52
159.22
133.38
127.57
120.86
117.97
115.07
113.86
112.16
82.92
82.80
54.42
52.54
52.14
30.00
ppm

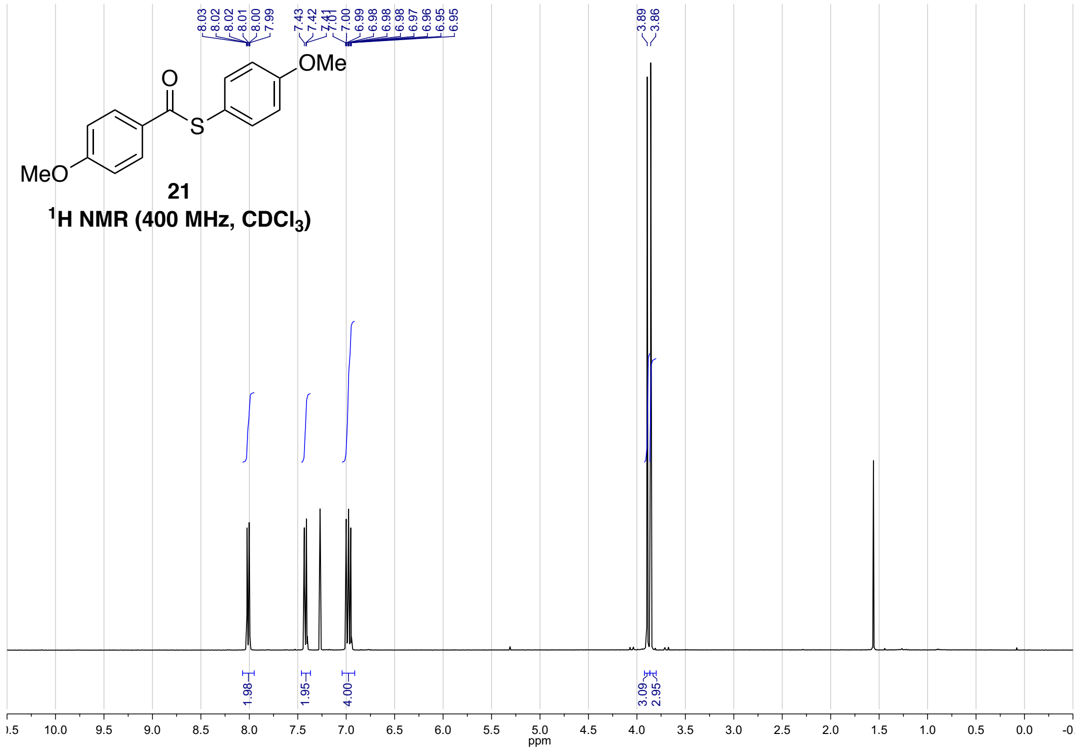
OMe
O
S
MeO
21
^{1}H NMR (400 MHz, CDCl$_3$)
8.03
8.02
8.02
8.01
8.00
7.99
7.43
7.42
7.41
7.01
7.00
6.99
6.98
6.98
6.98
6.97
6.96
6.95
6.95
3.89
3.86
1.98
1.95
4.00
3.09
2.95
ppm

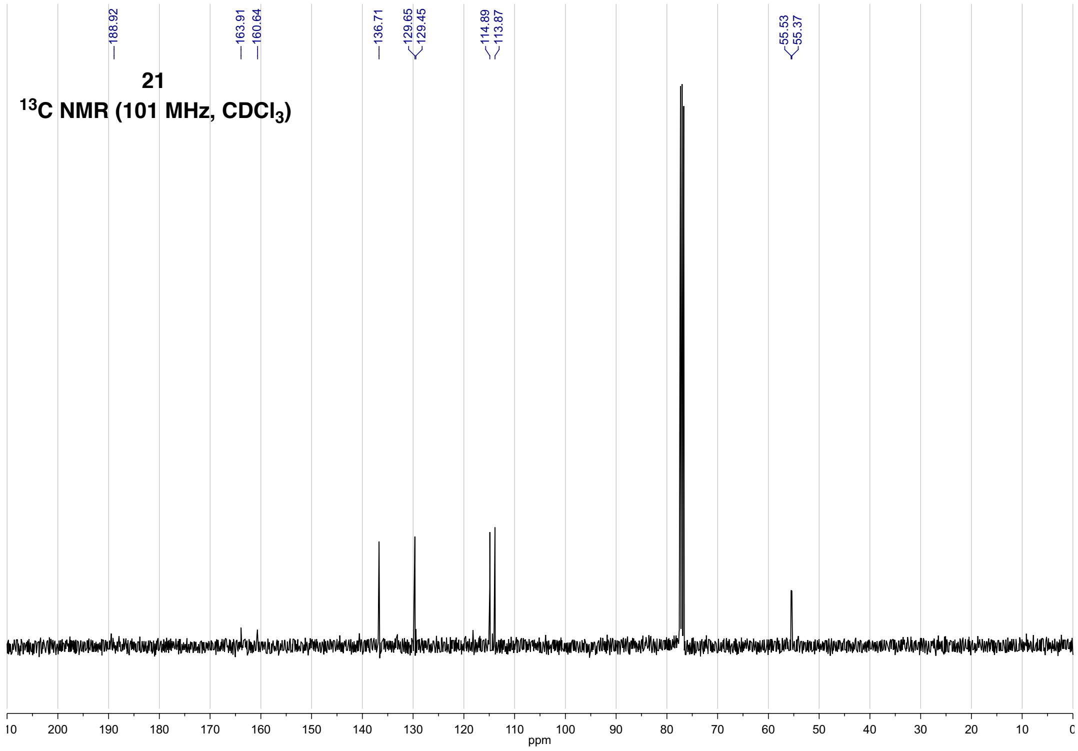
21
^{13}C NMR (101 MHz, CDCl$_3$)
188.92
163.91
160.64
136.71
129.65
129.45
114.89
113.87
55.53
55.37
ppm

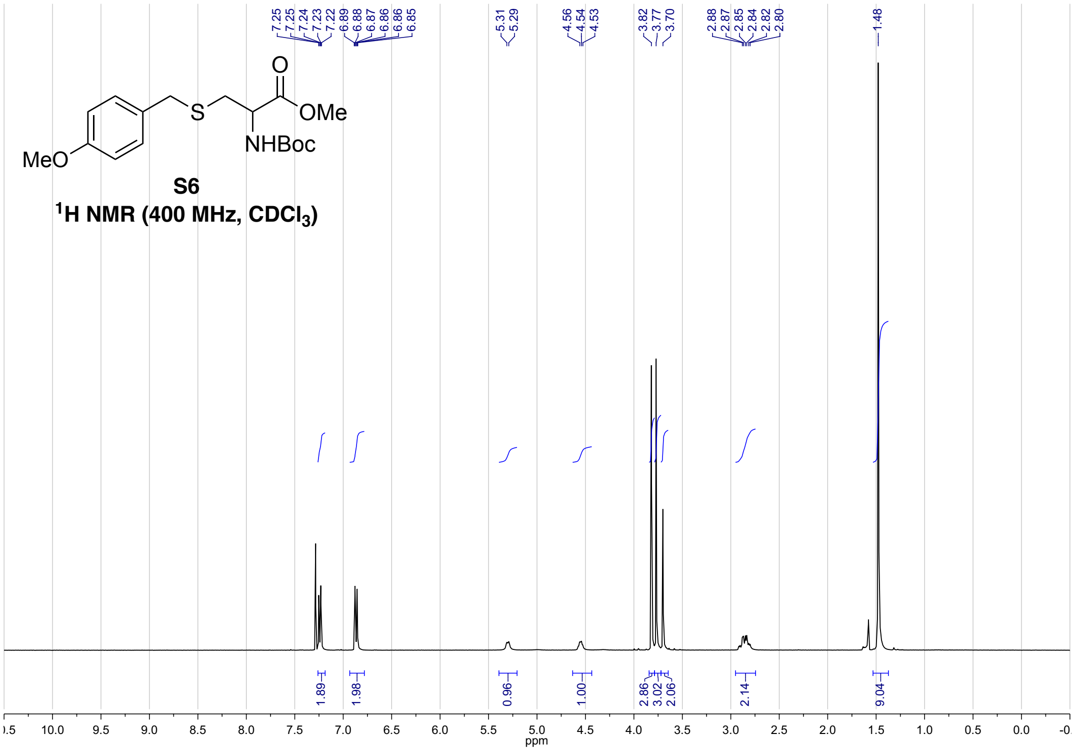
7.25
7.25
7.24
7.23
7.22
6.89
6.88
6.87
6.86
6.86
6.85
5.31
5.29
4.56
4.54
4.53
3.82
3.77
3.70
2.88
2.87
2.85
2.84
2.82
2.80
1.48
O
S
OMe
NHBoc
MeO
S6
1H NMR (400 MHz, CDCl3)
1.89
1.98
0.96
1.00
2.86
3.02
2.06
2.14
9.04
ppm

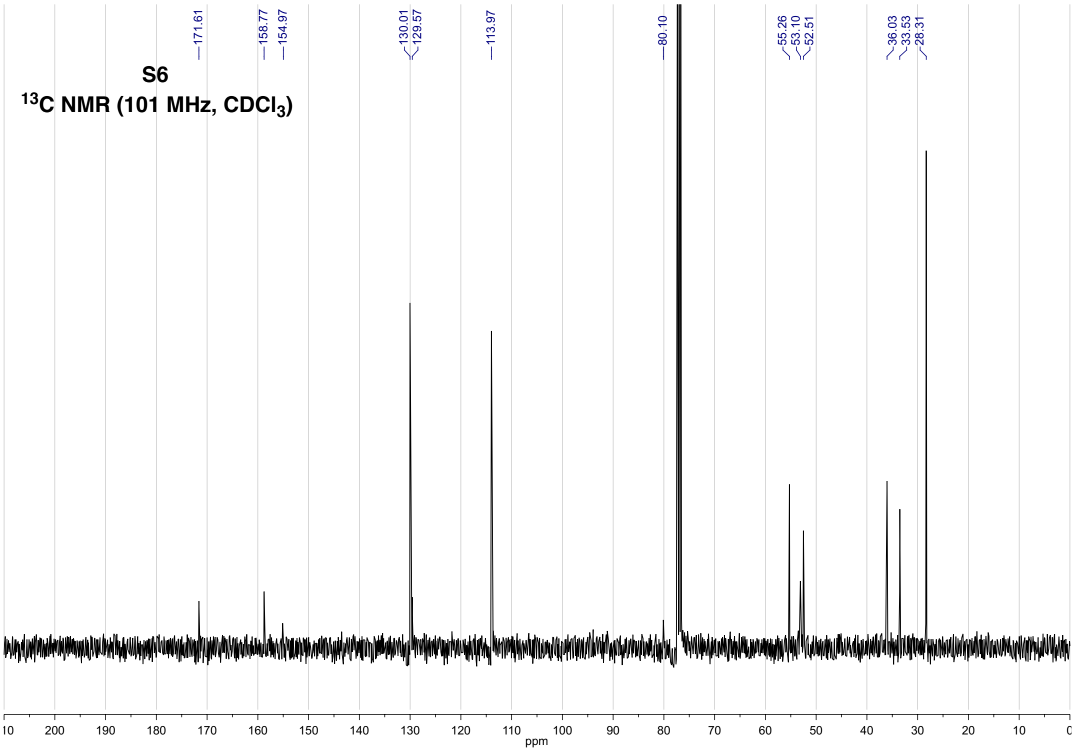
171.61
158.77
154.97
130.01
129.57
113.97
80.10
55.26
53.10
52.51
36.03
33.53
28.31
S6
13C NMR (101 MHz, CDCl3)
ppm

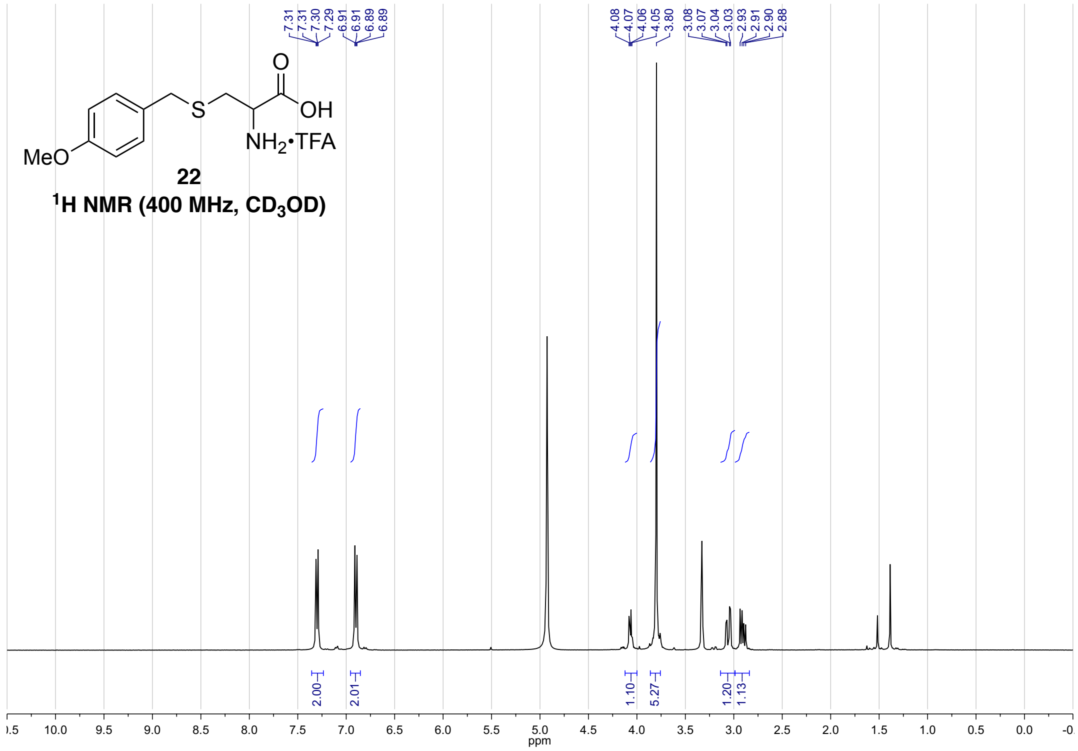

22

^{1}H NMR (400 MHz, CD_3OD)

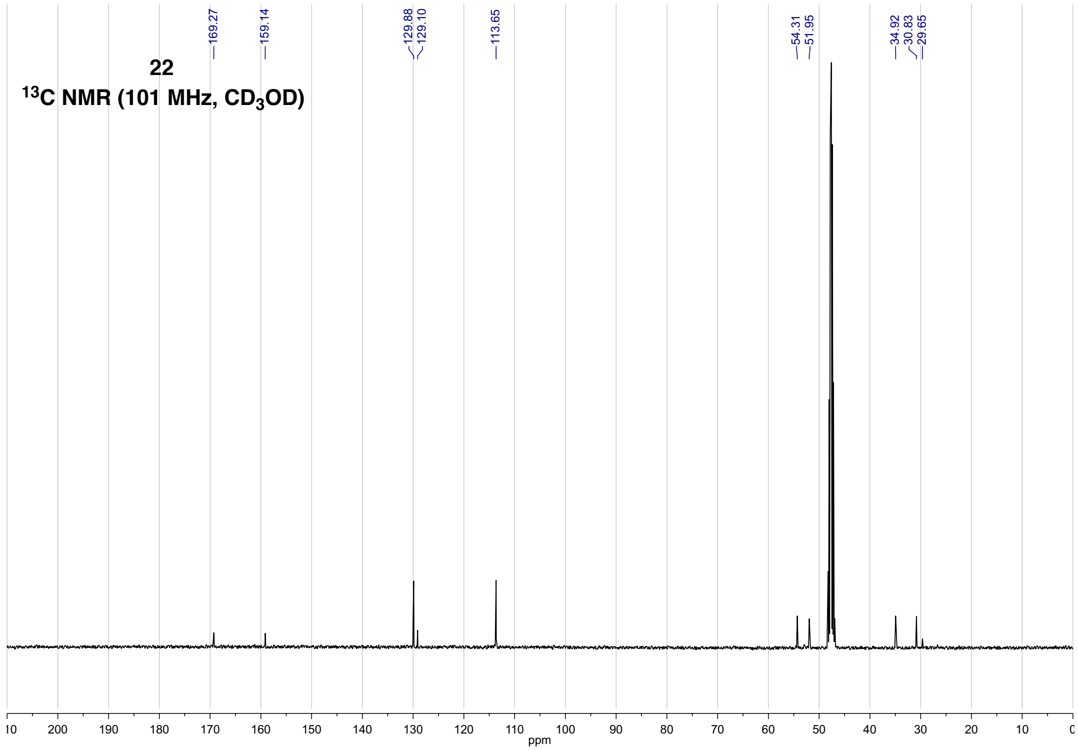

22

^{13}C NMR (101 MHz, CD_3OD)
